# Comparative multiomics analysis of cell physiological state after culture in a basket bioreactor

**DOI:** 10.1038/s41598-022-24687-4

**Published:** 2022-11-23

**Authors:** Shouzhi Yu, Miaomiao Guo, Yadan Zhang, Cunpei Bo, Hongyang Liang, Hui Wang, Xiaoming Yang

**Affiliations:** 1 Beijing Institute of Biological Products Company Limited, Beijing, China; 2 China National Biotec Group Company Limited, Beijing, China

**Keywords:** Biological techniques, Biotechnology, Computational biology and bioinformatics

## Abstract

Bioreactors are one of the most important, basic pieces of equipment in the biopharmaceutical industry. Understanding the effects of mechanical damage and other factors on the physiological state of cells during cell matrix culture is the basis for continuously achieving greater efficiency and higher product quality. In this study, Vero cells were used as a model and apoptosis, senescence, transcriptomics, proteomics, and metabolomics were carried out for analysis at the cellular and molecular levels. The results showed that compared with cells cultured in the simulated natural state, the cells cultured in the basket bioreactor displayed no obvious senescence. Additionally, the proportion of early apoptotic cells increased, but the proportions of damaged, late apoptotic and dead cells did not change significantly. The transcription levels of aminoacyl-tRNA synthetase and cyclin D1 and the expression levels of DNA replication licensing factor, methenyltetrahydrofolate cyclohydrolase, arachidonic acid and other metabolites of cells cultured in the basket bioreactor were significantly increased. These results suggest that DNA replication, protein translation and the metabolic activities in cells cultured in basket bioreactors are more active, which is more conducive to cell amplification and target product production. In this study, the growth and physiological state of cells in a basket bioreactor were characterized at the molecular level for the first time. Additionally, a tool to evaluate the physiological state of cells in a bioreactor was established, which can be used to guide the development and optimization of cell matrix culture conditions in industrial production and improve the production efficiency of the target products.

## Introduction

In the biopharmaceutical industry, biological products are mainly produced by culturing mammalian cells^1^. Researchers in industry and elsewhere continue to explore and improve cell culture density and production efficiency, striving to achieve the efficient production of target products.

Bioreactors have always been the core equipment for the production of vaccines or monoclonal antibodies using animal cells and play a very important role in the large-scale culture of animal cells^2^. The theory that animal cells are highly sensitive to induced effects such as fluid shear in the bioreactor has been broad and lasting, and cell growth and product quality are directly related to mechanical damage such as fluid shear in bioreactors^2^. Larger inverted conical shaker bioreactors generate relatively lower shear stress and higher cell density and antibody expression levels in BHK21 and CHO cells^3^. Hybridoma cell concentrations, respiratory activity, and monoclonal antibody concentrations decrease significantly in agitate bioreactors operated at high agitation rates, while lactate dehydrogenase release, the specific glucose consumption rate, and the cell death rate constant increase^4^. For efficient production, it is very important to develop bioreactors with low shear force and effective substance delivery. A basket bioreactor is a kind of fixed bed reactor. Existing studies have shown that basket bioreactors can produce high-density cell cultures with low (or no) shear force fluid characteristics. Their applications are becoming increasingly extensive, and the technology is becoming increasingly mature, especially for the industrial production of vaccines for human use^5,6^. A basket bioreactor has a special structure allowing cells to establish stationary culture, while fluid dynamic feeding nearly mimics the culture mode of natural growth^6,7^. However, there is still a lack of detailed studies on the physiological and metabolic characteristics of cells cultured in basket bioreactors.

The Vero cell line is considered the most commonly used continuous cell line for the production of vaccines and other biological products. Historically, it was the first cell line approved by the World Health Organization for the production of human vaccines. Comprehensive experimental data on the production of multiple viruses using the Vero cell line can be found in the literature^8^. It is worth mentioning that the Beijing Institute of Biological Products Co., Ltd. used a basket bioreactor to culture Vero cells to complete the large-scale production of a SARS-CoV-2 inactivated vaccine, providing a more effective solution for meeting the global vaccine supply during the current COVID-19 pandemic period. The rapid development of omics has provided us with convenient tools to systematically understand cellular state at the molecular level^9^. In previous studies, HeLa cells were used to pioneer omics approaches^10^, such as microarray-based gene expression profiling^11,12^ and responses to genetic perturbations^13^. In this study, Vero cells as model cells were cultured in basket bioreactors with the lowest potential mechanical damage, and the physiological state of Vero cells was explored with the aid of omics as a research method.

The growth and physiological status of basket bioreactor-cultured cells were systematically analysed for the first time, and the state and mechanism of basket bioreactor high-density cultured cells were characterized to provide new ideas for the establishment of tools to evaluate cell matrix culture status in industrial production, development and optimization of the cell matrix culture process, and improvement of cell yield and target product production efficiency.

## Results

### Physiological state of cells cultured in a basket bioreactor

**Figure 1:**
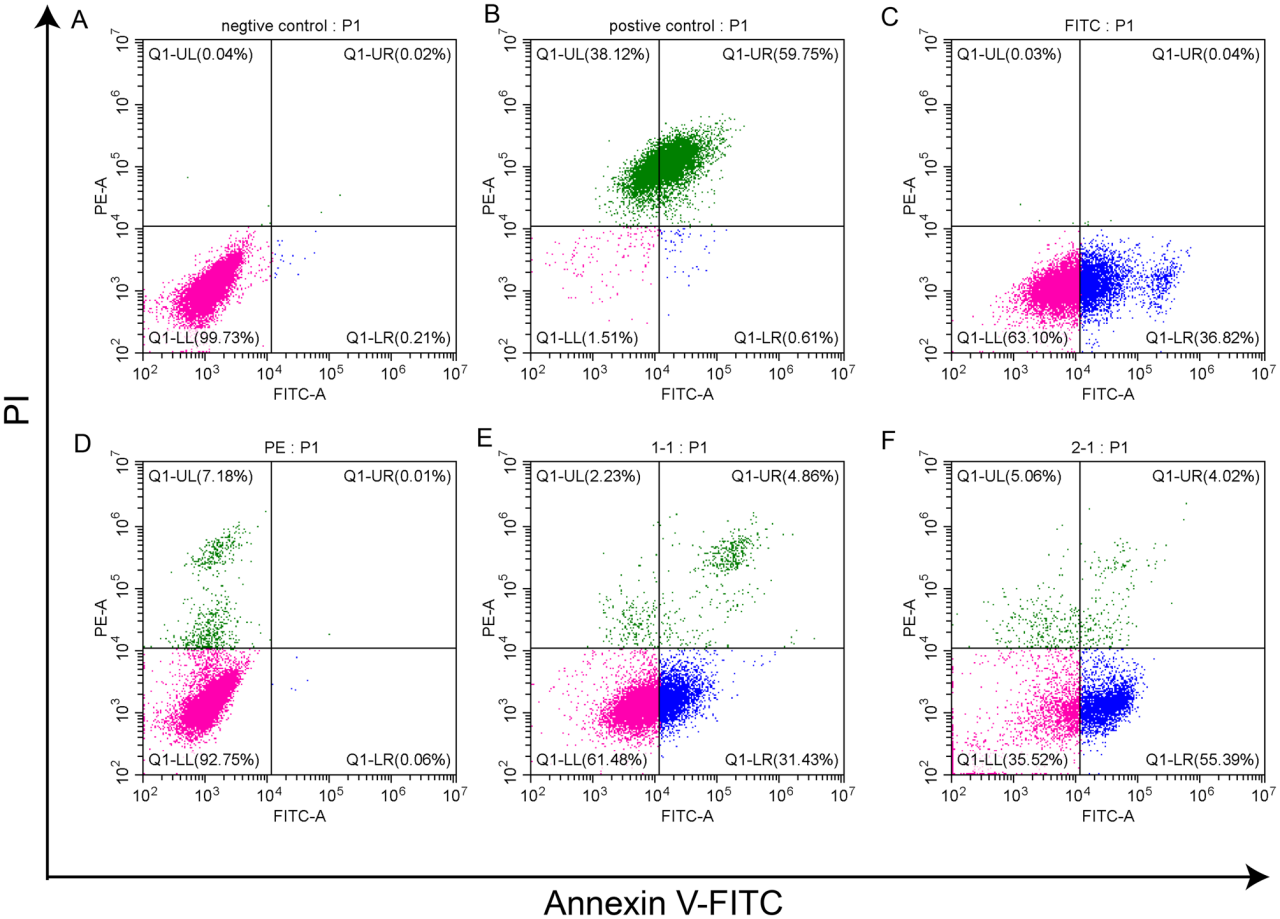
Apoptosis of cells cultured by two culture methods. (**A**) The gating strategy for flow cytometry analysis. (**B**) Apoptosis of cells treated with 4% paraformaldehyde (positive control). (**C**) Annexin V-FITC single staining control. (**D**) PI single staining control. (**E**) Apoptosis of cells in the natural growth state (Annexin V-FITC/PI double staining). (**F**) Apoptosis of cells in basket bioreactor culture (Annexin V-FITC/PI double staining).

We evaluated cell senescence and apoptosis to compare cell status at the cellular level between culture in a basket bioreactor and under conditions simulating the natural state using square flask culture. The results showed that the cells cultured in the bioreactor did not display significant cell senescence compared with those in the natural state (Fig. S1). The apoptosis assay was performed with Annexin V-FITC/propidium iodide (Annexin V/PI) double staining. The gating strategy for flow cytometry analysis was showed in Fig. 1A and the positive control was showed in Fig. 1B. The Fig. 1C,D showed the Annexin V-FITC single staining and Propidium iodide (PI) single staining control, respectively. The proportion of early apoptotic cells (Annexin V^+^/PI^−^) increased, but the numbers of damaged (Annexin V^−^/PI^+^), late apoptotic and dead cells (Annexin V^+^/ PI^+^) did not change significantly, indicating that although some of the cells cultured in the basket bioreactor experienced early apoptosis, the overall state of the cells did not change significantly, and the proportion of cell death did not increase (Fig. 1E,F).

### Transcriptomic analysis

**Figure 2:**
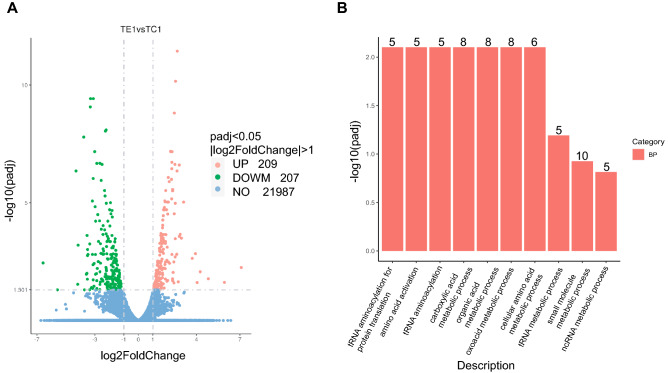
Transcriptome analysis of cells cultured in a basket bioreactor. (**A**) Differentially expressed genes in cells cultured in the basket bioreactor. (**B**) GO analysis of differentially expressed genes in cells cultured in the basket bioreactor. *TE1* samples from the basket bioreactor, *TC1* control samples.

To analyse the transcription level of cells cultured in a basket bioreactor at the molecular level, we performed a transcriptomic study (Fig. 2A). GO enrichment analysis of the differentially expressed genes showed that the transcription levels of multiple aminoacyl-tRNA synthetases were increased (Fig. 2B). These genes are involved in tRNA aminoacylation for protein translation, amino acid activation, tRNA aminoacylation, carboxylic acid metabolic processes, organic acid metabolic processes, oxoacid metabolic processes, and cellular amino acid metabolic processes among biological process (BP) terms, mainly including glycyl-tRNA synthetase 1 (GARS1), isoleucyl-tRNA synthetase 1 (IARS1), tryptophanyl-tRNA synthetase (WARS1), cysteinyl-tRNA synthetase 1 (CARS1), and methionyl-tRNA synthetase 1 (MARS1) (Table S1). Aminoacyl-tRNA synthetases are essential enzymes involved in protein translation that catalyse the aminoacylation of tRNAs through cognate homologous amino acids to nucleotide triplets contained in tRNAs^14^. The increased transcription levels of these genes suggest that the protein translation process in cells cultured in the basket bioreactor is more active.

KEGG analysis showed that the transcription levels of several genes involved in cell growth were increased, such as amphiregulin (AREG), CCND1, and EREG (Table S2)^15–17^. Studies have found that AREG is a ligand for epidermal growth factor receptor (EGFR), which activates major intracellular signalling cascades that control cell survival, proliferation, and motility through EGFR binding^18^. The cell cycle and cell proliferation/division are driven by cyclins and cyclin-dependent kinases (CDKs). Cyclin D1 binds to CDK4/6 and acts as a mitotic sensor to regulate the phase transition from G1 to S and initiate DNA replication^19^. Epiregulin is a novel growth factor that plays an important role in the reproductive process^20^. MYC is a transcription factor that regulates translation initiation factors and plays an important role in regulating cell growth and cell division^21^.

### Proteomic analysis

**Figure 3:**
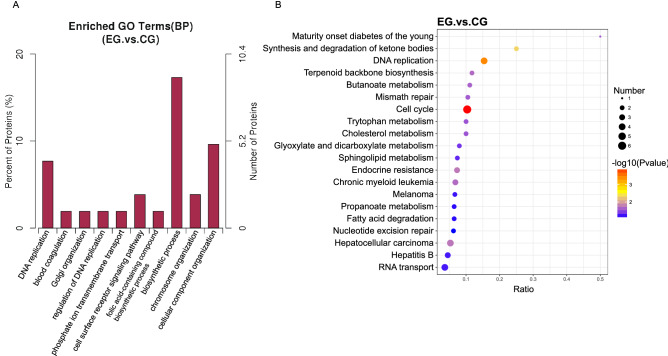
Differential protein enrichment analysis. (**A**) Analysis of differential protein GO enrichment of BP terms in cells cultured in basket bioreactors. (**B**) Upregulated protein bubble map by KEGG analysis of the differential proteins in cells cultured in basket bioreactor. *EG* samples from the basket bioreactor, *CG* control samples^15–17^.

To clarify the difference in protein expression between the two groups, we conducted protein difference analysis and enrichment analysis based on differential proteins with fold-change (FC) ≥ 2.0 and P value ≤ 0.05 or FC ≤ 0.50 and P value ≤ 0.05 (Table e S3). The results showed that the top three terms significantly enriched in BP had many terms associated with cell proliferation, including biosynthetic process, cellular component organization, and DNA replication (Fig. 3A).

The expression of a variety of proteins belonging to biosynthetic process terms was significantly increased, such as DNA replication licensing factor MCM3, DNA replication licensing factor MCM4, proliferating cell nuclear antigen (PCNA), methenyltetrahydrofolate cyclohydrolase (MTHFD2), and replication factor C subunit 1 (RFC) (Table S4). Minichromosome maintenance complex 3/4 (MCM3 and MCM4) is essential for regulating DNA replication and cell cycle progression. MCM3 precisely regulates DNA replication by controlling the number of DNA replication initiations in each cell cycle^22^. PCNA can interact with many components involved with cell replication and signal transduction mechanisms and can facilitate the exchange of DNA repair enzymes that recognize common DNA intermediates^23^. MTHFD2 is involved in supporting cytosolic purine synthesis in rapidly growing cells^24^, and human replication factor C (hRFC) is a multisubunit protein complex that supports PCNA-dependent DNA synthesis via DNA polymerases δ and ε (Fig. 3A).

The expression of proteins belonging to cell component organization terms, such as structural maintenance of chromosomes protein (SMC2), structural maintenance of chromosomes protein 1A (SMC1A), and BTB domain-containing protein, was also significantly increased (Table S5). Structural maintenance chromosome (SMC) proteins (SMC1 and SMC3) and auxiliary subunits constitute cohesin, which is involved in binding sister chromosomes to maintain the integrity of the chromosome structure during mitosis and meiosis^25^ (Fig. 3A).

Among the top 20 terms from KEGG enrichment analysis, the number of genes belonging to cell cycle and DNA replication was the largest, and these genes also showed the most significant changes, including SMC1A, MCM3, MCM4, PCNA, and RFC (Fig. 3B). The increased expression of these proteins suggested that the DNA replication process in the cells cultured in the basket bioreactor was more active and these cells may grow rapidly.

**Figure 4:**
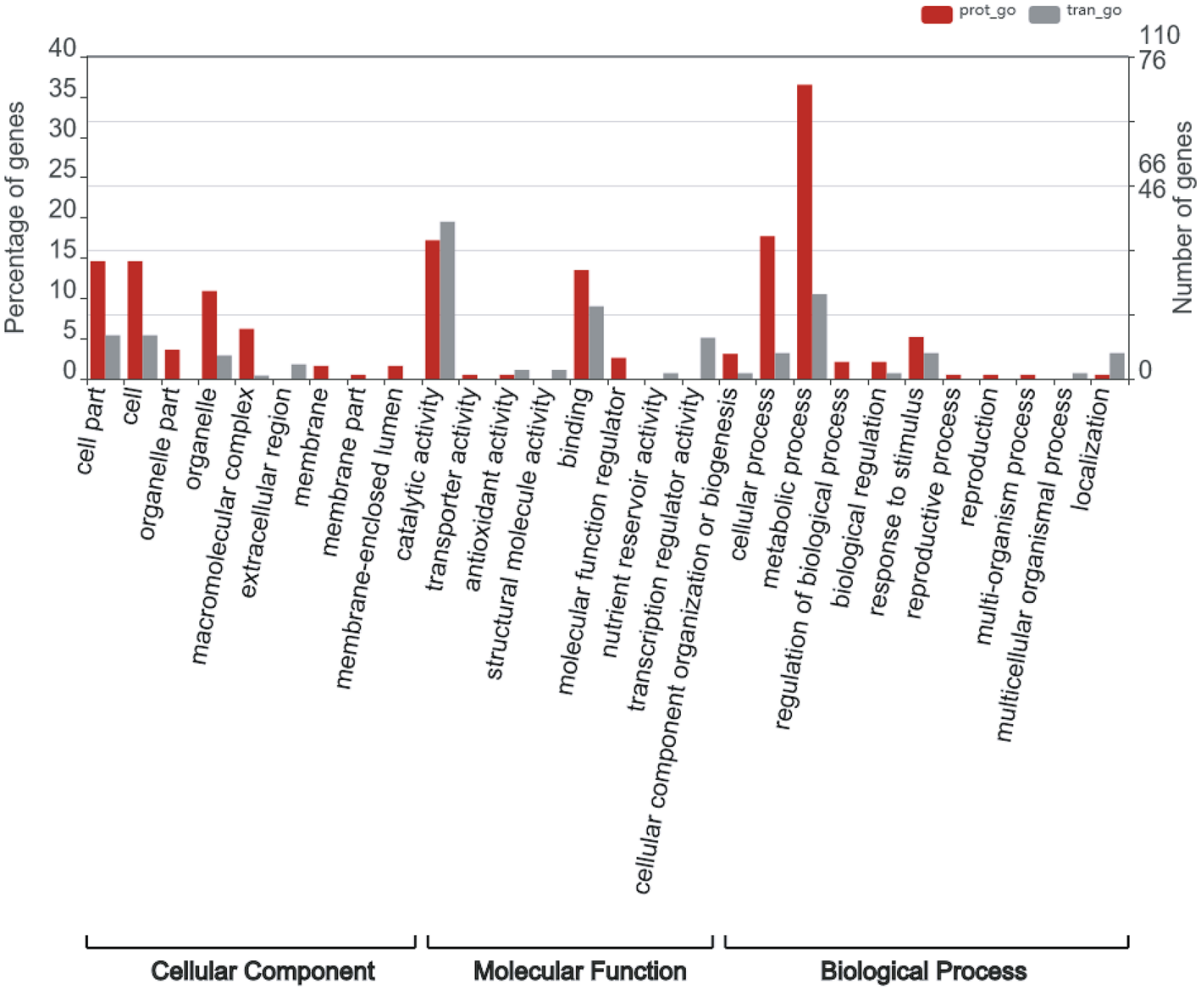
Association analysis of proteomics and transcriptomics. The red column represents the GO enrichment of the proteome, and the green column represents the GO enrichment of the transcriptome. The abscissa displays the enriched GO items, and the ordinate displays the number of enriched proteins (or genes) of the proteome and the transcriptome. The corresponding GO entries of the differential proteins and differential genes were extracted, and the graph was drawn using WEGO (http://wego.genomics.org.cn/).

The transcriptomics and proteomics association analysis showed that a large proportion of genes and proteins were involved in catalytic activity, metabolic processes, and cellular processes (Fig. 4). Further analysis revealed that these genes were mainly involved in sphingolipid metabolism, nitrogen metabolism, glutathione metabolism, cholesterol metabolism and other metabolic processes, indicating that the metabolic processes of the cells cultured in the basket bioreactor were more active.

### Metabolomics analysis

**Figure 5:**
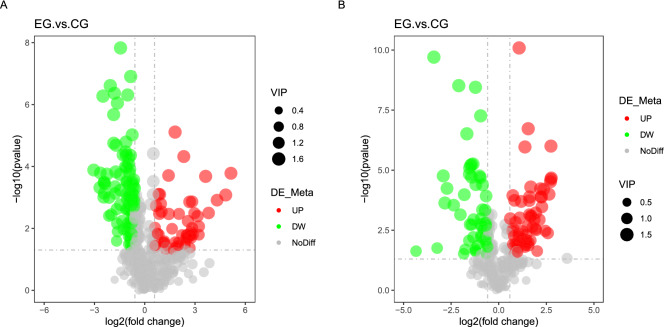
Analysis of the differential metabolites in cells cultured in a basket bioreactor. (**A**) Volcano diagram of the differential metabolites in positive ion mode. (**B**) Volcano diagram of the differential metabolites in negative ion mode. The abscissa displays the expression fold change (log_2_FC) of metabolites in different groups, and the ordinate displays the level of significant difference (− log_10_(p value)). Each point in the graph represents a metabolite, and the size of the point represents the VIP value. The significantly upregulated metabolites are represented by red points, and the significantly downregulated metabolites are represented by green points. *VIP* variable importance in projection reflecting the contribution of the quantitative value of each sample to the difference, generally set to VIP > 1, *DE_Meta* differential expression of metabolites.

To systematically investigate the metabolic status of the cells cultured in a basket bioreactor, we conducted metabolomics analysis. The results identified 509 and 285 metabolites in the cells cultured in basket bioreactors in positive and negative ion modes, respectively, of which 164 and 119 metabolites were significantly different (56 and 60 metabolites were upregulated, and 108 and 59 metabolites were downregulated, respectively) (Table S6)^6,26,27^. In positive ion mode, the levels of cytidine, cholecalciferol, uridine 5′-monophosphate and guanosine monophosphate belonging to metabolic pathways were significantly increased. The levels of psychosine, a metabolite of sphingolipid metabolism, palmitoleic acid, a metabolite of the fatty acid biosynthesis pathway, and adenosine 5′-monophosphate, a metabolite of the cAMP signalling pathway, were also significantly increased (Fig. 5A). The detail information of some differential metabolites showed in Table S7. The negative ion mode experiment revealed that the content of the metabolite arachidonic acid was significantly increased (Fig. 5B). Arachidonic acid is present in all mammalian cells and is usually esterified in membrane phospholipids. It is one of the most abundant polyunsaturated fatty acids that is important for normal membrane fluidity and a substrate for many enzymes that form bioactive lipid mediators^28^.

**Figure 6:**
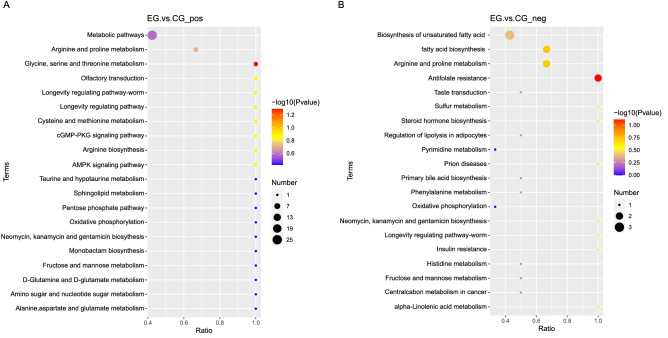
KEGG analysis of the differential metabolites in cells cultured in a basket bioreactor. KEGG analysis of the differential metabolites analysed in (**A**) positive ion mode and (**B**) negative ion mode^15–17^.

The KEGG enrichment analysis bubble plot shows that the metabolites with the greatest difference in positive ion mode were concentrated in glycine, serine and threonine metabolism, and the pathways with the most differential metabolites were metabolic pathways (Fig. 6A). In negative ion mode, more metabolites were involved in the biosynthesis of unsaturated fatty acids, while the metabolites with the greatest changes were involved in antifolate resistance (Fig. 6B).

Metabolomics and transcriptomics association analysis was also performed, and the main biochemical pathway and signal transduction pathway involved in differential metabolites and differential genes were aldosterone synthesis and secretion and amino acid biosynthesis. Comprehensive analysis of the transcriptome, proteome and metabolome showed that the differences in cells cultured in basket bioreactors mainly focused on protein synthesis and amino acid metabolism compared with those in the natural state, suggesting that Vero cells cultured in basket bioreactors may grow faster and proliferate rapidly.

## Discussion

There are many types of bioreactors. Mechanical damage in bioreactors, such as fluid shear force, has a great impact on the cell state, and shear stress is also one of the key factors that affects the large-scale culture of mammalian cells. At present, research on the effect of mechanical factors such as bioreactor shear force on cells has mostly focused on cell density, cell viability and product expression, and there is a lack of systematic research on the effects of such mechanical factors on the physiological state of the cells. In particular, systematic research can optimize and improve the efficacy of cell matrix culture based on the mechanism. The combination of a new carrier bed and stirring paddle in a basket bioreactor can significantly reduce mechanical damage such as fluid shear force, provide a large specific surface area using carriers, and ensure the static culture of cells. Moreover, mechanical stirring can also maintain the effective transmission of nutrients, and it is therefore widely used in the large-scale production of biological products. In this study, the physiological state of model cells (Vero cells) was analysed after culture in a potential shear-free basket bioreactor at both the cellular level (cellular senescence and apoptosis) and molecular level (transcriptomics, proteomics, and metabolomics).

First, compared with the square flask culture simulating the natural state, the proportion of early apoptotic cells cultured in the basket bioreactor increased, but the proportions of damaged, late apoptotic and dead cells did not increase significantly. The cells cultured in the basket bioreactor did not show senescence, indicating that the physiological state of the cells was not greatly affected at the cellular level. Macroscopically, cell detachment is proportional to the flow rate in flow-perfused bioreactors^29^. Foetal hepatocytes cultured in packed bed reactors are very sensitive to medium flow, inhibiting their growth and albumin secretion activity^30^. Compared with these previous reports, the macroscopic characterization data are more optimistic.

Then, we performed a systematic analysis of the molecular-level changes in cells cultured in a basket bioreactor. The transcriptomic results showed that the transcription levels of several aminoacyl-tRNA synthetases involved in protein translation were significantly increased in cells cultured in a basket bioreactor. Aminoacyl-tRNA synthetase (aaRS) directly controls the speed and fidelity of mRNA translation and regulates protein homeostasis in cells. Human TARS1 (cytosolic threonyl-tRNA synthetase, ThrRS) interacts with eIF4E homologous proteins and recruits other translation initiation complexes to form an eIF4F-like complex to mediate translation initiation^31^. These results indicate that the protein translation process is more active in cells cultured in basket bioreactors. The proteomic results revealed that the expression of proteins involved in DNA replication and cell cycle pathways was significantly increased in cells cultured in basket bioreactors, such as DNA replication licensing factor, proliferating cell nuclear antigen, methylenetetrahydrofolate cyclohydrolase, etc., suggesting that the DNA replication process is more active in cells cultured in basket bioreactors and may present a trend towards rapid growth. The metabolomic results showed that the metabolites involved in metabolic pathways, sphingolipid metabolism and fatty acid biosynthesis changed significantly in cells cultured in the basket bioreactor.

In summary, our study found that from the perspective of the culture cycle, the characteristics of low shear force in basket bioreactors did not significantly affect the overall physiological state of cultured cells. Moreover, compared with the natural state, DNA replication, protein translation, metabolic pathways and other processes of cell growth and proliferation are more active in basket bioreactor culture, indicating that this culture mode is more conducive to the proliferation of the mammalian cell matrix to meet the needs of target product production. This study revealed that the basket bioreactors produced no obvious effect from mechanical damage on cultured mammalian cells at the cellular and molecular levels. Combined with the characteristics of static cell culture and dynamic nutrients feeding, a basket bioreactor ensures a good physiological state of the cells and sufficient nutrient supply. In addition, the high specific surface area of the carrier is more conducive to obtaining more cell matrix and improving the production efficiency in the extremely clean environment of biopharmaceutical facilities. This study provides a systematic evaluation method for continuously improving the yield of the cell matrix and directionally regulating the cell state and gives an important tool for the selection of technical routes and equipment for cell matrix culture and amplification during biopharmaceutical manufacturing processes.

## Materials and methods

### Cell culture

**Figure 7:**
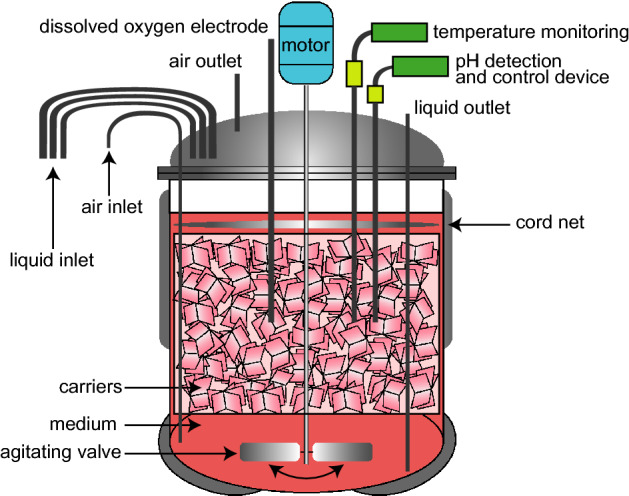
Schematic of the basket reactor.

The original Vero cell lines used in this study were obtained directly from the UK Health Authority HPACC. The 147-generation cells were cultured in 199 medium (Gibco, 31100019) at 37 ± 0.5 °C, pH 7.2–7.4. The cells were inoculated into a square bottle (Corning, T225) or a basket bioreactor at a ratio of 1:6. The parameters of the basket bioreactor were set as follows: stirring speed 70–90 rpm, dissolved oxygen 40–80%, 4-gas mode (air, oxygen, carbon dioxide and nitrogen), and continuous perfusion culture. The attached carrier (Chukun Biology, TQ1000W) was used for cell growth (Fig. 7). Then, the cells were digested and collected after 5 days of culture.

### Cell senescence assay

The cells cultured in the square flask (T225 cm^2^) and basket bioreactor (10 L) were washed twice with sterile PBS to remove serum. The cells were digested with 0.25% trypsin until they became round, during which time they were gently shaken to ensure that the trypsin had come in full contact with the cells. Each cell suspension was taken for cell counting after blowing to mix the cells. The cells were inoculated into a 6-well plate at 2 × 10^5^ cells/well with three replicate wells for each group. After the cells were cultured in a 37 °C cell incubator overnight, a cell senescence assay was performed according to a β-galactosidase staining kit (Yuanye Biology, R20438-100T). The cells were washed once with PBS before being fixed at room temperature for 15 min with β-galactosidase staining fixative. Then, the fixative was removed, and PBS (Procell, PB180327) was used to wash the cells three times. After the staining working solution was added, the cells were incubated overnight at 37 °C. The number of positively stained and unstained cells was counted under an ordinary light microscope.

### Apoptosis assay

The cells cultured in the square flask (T225 cm^2^) and basket bioreactor (10 L) were washed twice with sterile PBS. The digestion of the cells was carried out according to the same procedure described for the cell senescence assay. The cell suspension was taken for cell counting after blowing to mix the cells. The apoptosis assay was performed following the manufacturer’s instructions of the Annexin V-FITC/PI Apoptosis Kit (KeyGEN BioTECH, KGA108). After the cells were centrifuged at 1100 rpm for 3 min, the cells were washed twice with PBS. Binding buffer was added to resuspend the cells, and Annexin V-FITC and PI were used to stain the cells. Equal amounts of Vero cells cultured in square flasks were fixed in 4% paraformaldehyde for 15 min before Annexin V-FITC/PI staining as the positive control. The cells were analysed by flow cytometry.

### Transcriptomics detection

#### Sample collection and preparation

Total amounts and integrity of RNA were assessed using the RNA Nano 6000 Assay Kit of the Bioanalyzer 2100 system (Agilent Technologies, CA, USA). The extracted RNA was analysed by agarose gel electrophoresis for RNA integrity and DNA contamination. A NanoPhotometer spectrophotometer was used to determine RNA purity and an Agilent 2100 bioanalyzer was used for precise RNA integrity analysis. Complete high-purity RNA was detected for library construction with a total amount of initial RNA of greater than 1 μg. Sequencing library construction was carried out using Illumina's NEBNext^®^ Ultra™ RNA Library Prep Kit. The mRNA with a polyA tail was enriched by Oligo (dT) magnetic beads, and then the obtained mRNA was randomly interrupted with divalent cations in NEB Fragmentation Buffer. The first strand of cDNA was synthesized in the M-MuLV reverse transcriptase system using fragmented mRNA as a template and random oligonucleotides as primers. Then, the RNA strand was degraded by RNaseH, and the second strand of cDNA was synthesized from dNTPs in the DNA polymerase I system. The purified double-stranded cDNA was subjected to end-repair, A-tail and linker ligation. AMPure XP beads were used to screen approximately 250–300 bp cDNA for PCR amplification, and the PCR products were purified by using AMPure XP beads again. After library construction was completed, a Qubit 2.0 fluorometer was used for preliminary quantification, the library was diluted to 1.5 ng/µl, and the library fragment size was detected using an Agilent 2100 bioanalyser. qRT‒PCR was used to accurately quantify the effective concentration of the library (the effective concentration of the library was higher than 2 nM) to ensure the quality of the library. After the quality inspection of the sequencing library was completed, sequencing was performed, with PE150 being selected for sequencing.

#### Data analysis

TO ensure the quality and reliability of the data analysis, the original data were subjected to quality control. This protocol mainly included the removal of adaptor reads, N-containing reads (N indicates that the base information cannot be determined), and low-quality reads (the number of bases with Qphred < = 20 accounting for more than 50% of the entire read length). Then, Q20, Q30 and GC content calculations were performed on the clean data. All subsequent analyses were high-quality analyses based on clean data. After quality control, the sequence data were analysed, and the reference genome and gene model annotation files were downloaded from the genome website. We used HISAT2 v2.0.5 to construct an index of the reference genome and used HISAT2 v2.0.5 to align the paired terminal clean reads with the reference genome. Novel genes were predicted using StringTie. StringTie applies a network flow algorithm and optional de novo assembly to splice transcripts. Gene expression level quantification and differential expression analysis, differential gene enrichment analysis, differential gene protein network interaction analysis and SNP analysis were performed.

### Proteomic analysis

#### Sample collection and preparation

The sample was removed from the − 80 °C freezer and transferred to a 1.5 mL centrifuge tube. An appropriate amount of DB protein solution (8 M urea, 100 mM TEAB, pH = 8.5) was added, mixed well by shaking, and ultrasonicated for 5 min in an ice water bath. After centrifugation at 4 °C and 12,000×*g* for 15 min, the supernatant was added to 10 mM DTT and reacted at 56 °C for 1 h. Then, a sufficient amount of IAM was added for reaction at room temperature in the dark for 1 h. The extracted total protein was detected by a Bradford protein quantification kit. Each 20 μg protein sample was subjected to 12% SDS-PAGE. The concentrated gel electrophoresis conditions were 80 V for 20 min, and the separation gel electrophoresis conditions were 120 V for 90 min. After electrophoresis, Coomassie brilliant blue R-250 staining was performed, and the bands were clear. Protein samples were taken, and DB protein solution (8 M urea, 100 mM TEAB, pH = 8.5) was added to supplement the volume to 100 μL. Trypsin and 100 mM TEAB buffer were added, and the solution was mixed. After digestion at 37 °C for 4 h, trypsin and CaCl_2_ were added for digestion overnight. The pH was adjusted to less than 3 by adding formic acid. After mixing, the mixture was centrifuged at room temperature and 12,000×*g* for 5 min. The supernatant was slowly passed through a C18 desalination column, and then cleaning solution (0.1% formic acid, 3% acetonitrile) was used 3 times for continuous cleaning. Then, an appropriate amount of eluent (0.1% formic acid, 70% acetonitrile) was added. The filtrate was collected and lyophilized. Mobile phases A (100% water, 0.1% formic acid) and B (80% acetonitrile, 0.1% formic acid) were prepared. A total of 4 μg of supernatant from each sample was added to 0.8 μL of iRT reagent, and then half the volume of the sample was added to the instrument. An EASY-nLCTM1200 nano-upgrade UHPLC system was used for detection. A Q ExactiveTM HF-X mass spectrometer with a Nanospray Flex TM (ESI) ion source was used. The ion spray voltage was set at 2.1 kV and the ion transport tube temperature was 320 °C, and mass spectrometry was performed in data-independent acquisition (DIA) mode. The full scan range was m/z 350–1500.

#### Data analysis

Protein profiling data analysis was performed for protein identification and quantification, differential protein analysis, functional analysis of proteins and differentially expressed proteins (DEPs), and GO and KEGG pathway enrichment analysis of the DEPs.

### Metabolomics analysis

#### Sample collection and preparation

The cell sample was placed in an EP tube, and 300 μL of 80% methanol aqueous solution was added. The samples were frozen in liquid nitrogen for 5 min; after melting on ice, they were vortexed for 30 s, ultrasonicated for 6 min, and centrifuged at 5000 rpm and 4 °C for 1 min. The supernatant was transferred to a new centrifuge tube and freeze-dried into a dry powder. According to the volume of the sample, an appropriate amount of 10% methanol solution was added to dissolve the sample for injection into the LC‒MS system for analysis. The chromatographic conditions were as follows. Chromatographic column: Hypersil GOLD (C18); column temperature: 40 °C; flow rate: 0.2 mL/min; positive mode: mobile phase A: 0.1% formic acid, mobile phase B: methanol; negative mode: mobile phase A: 5 mM ammonium acetate, pH 9.0, mobile phase B: methanol; and a gradient elution program was utilized for sample separation. The mass spectrometry conditions were as follows. Scanning range m/z 100–1500; ESI source settings: spray voltage: 3.5 kV; sheath gas flow rate: 35 psi; aux gas flow rate: 10 L/min; capillary temperature: 320 °C; s-lens RF level: 60; aux gas heater temperature: 350 °C; and polarity: positive or negative. MS/MS secondary scans were data-dependent.

#### Data analysis

The KEGG database, HMDB database and LIPID Maps database were used to annotate the identified metabolites. For univariate analysis, the statistical significance (P value) of each metabolite between the two groups was calculated based on the t test, and the fold change (FC value) of the metabolites between the two groups was calculated. The default criteria for screening differential metabolites were VIP > 1, P < 0.05 and FC ≥ 2 or FC ≤ 0.5. Volcano diagram analysis and correlation analysis between the differential metabolites were performed.

## Supplementary Information

Supplementary Information 1.

Supplementary Information 2.

## Data Availability

All data generated or analysed during this study are included in its supplementary information files.

## References

[1] Shi S (2014). Biologics: An update and challenge of their pharmacokinetics. Curr. Drug Metab..

[2] Ding N (2020). Numerical simulation and optimization of impeller combination used in stirred bioreactor. Sheng Wu Gong Cheng Xue Bao.

[3] Ding N (2019). Numerical simulation of scaling-up an inverted frusto-conical shaking bioreactor with low shear stress for mammalian cell suspension culture. Cytotechnology.

[4] Abu-Reesh I, Kargi F (1991). Biological responses of hybridoma cells to hydrodynamic shear in an agitated bioreactor. Enzyme Microb. Technol..

[5] Wang H (2020). Development of an inactivated vaccine candidate, BBIBP-CorV, with potent protection against SARS-CoV-2. Cell.

[6] Lesch HP, Valonen P, Karhinen M (2021). Evaluation of the single-use fixed-bed bioreactors in scalable virus production. Biotechnol. J..

[7] Lazar A (1991). Immobilization of animal cells in fixed bed bioreactors. Biotechnol. Adv..

[8] Kiesslich S, Kamen AA (2020). Vero cell upstream bioprocess development for the production of viral vectors and vaccines. Biotechnol. Adv..

[9] Yang M, Soga T, Pollard PJ (2013). Oncometabolites: Linking altered metabolism with cancer. J. Clin. Investig..

[10] Landry JJ (2013). The genomic and transcriptomic landscape of a HeLa cell line. G3 (Bethesda).

[11] Chaudhry MA (2002). Gene expression profiling of HeLa cells in G1 or G2 phases. Oncogene.

[12] Murray JI (2004). Diverse and specific gene expression responses to stresses in cultured human cells. Mol. Biol. Cell.

[13] Jaluria P (2007). Enhancement of cell proliferation in various mammalian cell lines by gene insertion of a cyclin-dependent kinase homolog. BMC Biotechnol..

[14] Cho S (2020). Endogenous TLR2 ligand embedded in the catalytic region of human cysteinyl-tRNA synthetase 1. J. Immunother. Cancer.

[15] Kanehisa M, Goto S (2000). KEGG: Kyoto encyclopedia of genes and genomes. Nucleic Acids Res..

[16] Kanehisa M (2019). Toward understanding the origin and evolution of cellular organisms. Protein Sci..

[17] Kanehisa M, Furumichi M, Sato Y, Ishiguro-Watanabe M, Tanabe M (2021). KEGG: Integrating viruses and cellular organisms. Nucleic Acids Res..

[18] Berasain C, Avila MA (2014). Amphiregulin. Semin. Cell Dev. Biol..

[19] Tchakarska G, Sola B (2020). The double dealing of cyclin D1. Cell Cycle.

[20] Cui X (2020). Epiregulin promotes trophoblast epithelial-mesenchymal transition through poFUT1 and O-fucosylation by poFUT1 on uPA. Cell Prolif..

[21] Schmidt EV (2004). The role of c-myc in regulation of translation initiation. Oncogene.

[22] Ma H (2021). Bioinformatics analysis reveals MCM3 as an important prognostic marker in cervical cancer. Comput. Math. Methods Med..

[23] Ivanov I (2006). Proliferating cell nuclear antigen loaded onto double-stranded DNA: Dynamics, minor groove interactions and functional implications. Nucleic Acids Res..

[24] Christensen KE, Mackenzie RE (2008). Mitochondrial methylenetetrahydrofolate dehydrogenase, methenyltetrahydrofolate cyclohydrolase, and formyltetrahydrofolate synthetases. Vitam. Horm..

[25] Yoshinaga M, Inagaki Y (2021). Ubiquity and origins of structural maintenance of chromosomes (SMC) proteins in eukaryotes. Genome Biol. Evol..

[26] Berrie DM (2020). Development of a high-yield live-virus vaccine production platform using a novel fixed-bed bioreactor. Vaccine.

[27] Tapia F (2016). Bioreactors for high cell density and continuous multi-stage cultivations: Options for process intensification in cell culture-based viral vaccine production. Appl. Microbiol. Biotechnol..

[28] Martin SA, Brash AR, Murphy RC (2016). The discovery and early structural studies of arachidonic acid. J. Lipid Res..

[29] McCoy RJ, Jungreuthmayer C, O'Brien FJ (2012). Influence of flow rate and scaffold pore size on cell behavior during mechanical stimulation in a flow perfusion bioreactor. Biotechnol. Bioeng..

[30] Miyoshi H (2010). Three-dimensional perfusion cultures of mouse and pig fetal liver cells in a packed-bed reactor: Effect of medium flow rate on cell numbers and hepatic functions. J. Biotechnol..

[31] Jeong SJ (2019). A threonyl-tRNA synthetase-mediated translation initiation machinery. Nat. Commun..

