# Supplementary material for: Comparative multiomics analysis of cell physiological state after culture in a basket bioreactor
Source: Sci Rep. 2022 Nov 23;12:20161. doi: 10.1038/s41598-022-24687-4 (PMC9686226; doi:10.1038/s41598-022-24687-4)
Supplement: Supplementary file 1 — Supplementary Information 1. [file 41598_2022_24687_MOESM1_ESM.zip › raw data/Metabolomics raw data/1.MetQuant-QC/1-MetQuant-QC-readme.pdf]

MetQuant-QC Readme

|-- 1.MetQuant-QC 【代谢物定性定量结果目录】
| |-- meta_intensity_{pos,neg,all}.xls 【所有代谢物定性定量列表】
| |-- sam_qc_infor_{pos,neg,all}.xls 【样本信息及总 PCA 图中样本编号信息】
| |-- Samples_QC_{pos,neg,all}-PCA[.3D].{png,pdf} 【QC 及所有代谢样本的总 PCA 图】
| |-- Samples_ QC _pcaloading_{pos,neg,all}.{png,pdf} 【QC 及所有代谢样本总 PCA 图的载荷图】
| |-- Samples_{pos,neg,all}-PCA[.3D].{png,pdf} 【所有代谢样本的总 PCA 图】
| |-- Samples_pcaloading_{pos,neg,all}.{png,pdf} 【所有代谢样本总 PCA 图的载荷图】
| |-- **【TIC】**
| |-- Samples_Specialized_Traces_{pos,neg,all}.xls 【QC 及样本的 TIC 图】
| |-- **【Correlation】**
| |-- cor_pearson_{pos,neg,all}.{png,pdf,xls} 【QC 样本相关性分析结果】

meta_intensity_{pos,neg,all}.xls 【所有代谢物定性定量列表】

第一列：Compound_ID/ID，代谢物 ID （该 ID 是为了方便检索及后续分析而随机添加的编号，无实际意义）；

第二列：Name，代谢物的名称（数据库中代谢物的名称）；

第三列：Formula，代谢物的分子式；

第四列：Molecular Weight，代谢物的相对分子量；

第五列：RT [min]，保留时间；

第六列：m/z，质荷比；

第七列：mzCloud_Results，代谢物与 mzCloud 数据库的匹配结果；

第八列：mzVault_Results，代谢物与 mzVault 数据库的匹配结果；

第九列：MassList_Results，代谢物与 MassList 数据库的匹配结果，匹配结果解释详情，请查阅结题报告名词解释附件；

第十列～：代谢物在各个样本中的相对定量信息（峰面积值）；

sam_infor_{pos,neg,all}.xls

第一列：Sample name ，样本名称；

第二列：Batch，上机批次；

第三列：Group name，组别名称；

第四列：ID num，样本名称顺序（总样本 PCA 图对应的编号）；

cor_pearson_{pos,neg,all}.{png,pdf,xls}

cor_pearson_{pos,neg,all}.xls QC 样本两两相关性 R2 的值

cor_pearson_{pos,neg,all}.{png,pdf }

横坐标、纵坐标分别为 QC 样本，QC 样本两两相关性 R2 的值越接近于 1，表明 QC 的相关性越好，说明整个检测过程稳定性越好。

Samples_QC_{pos,neg,all}-PCA[.3D].{png,pdf}

实验样本+QC 样本的 PCA 分析：横坐标 PC1 和纵坐标 PC2 分别表示排名第一和第二的主成分的得分，不同颜色的散点表示不同实验分组的样本，椭圆为 95%的置信区间，QC 越聚集说明检测过程稳定性越好。

Samples_ QC_pcaloading_{pos,neg,all}.{png,pdf}

样本比较对 PCA loading 分析载荷图：载荷图（loading plot）的横坐标代表每个物质 在第一主成分上的载荷大小（cosα），纵坐标代表每个物质在第二主成分上的载荷大小（cosβ）。载荷图本质上描述的是构成第一主成份和第二主成份的线性方程的系数，载荷的绝对值越大，对于主成份的影响就越大。

Samples_{pos,neg,all}-PCA[.3D].{png,pdf}

实验样本 PCA 分析：横坐标 PC1 和纵坐标 PC2 分别表示排名第一和第二的主成分的得分，不同颜色的散点表示不同实验分组的样本，椭圆为 95%的置信区间，除 QC 样本外，考察实验样本组内聚集及组间的离散情况。

Samples_Specialized_Traces_{pos,neg,all}.xls

为 QC 样本、实验样本的 TIC 图
