# Supplementary material for: Comparative multiomics analysis of cell physiological state after culture in a basket bioreactor
Source: Sci Rep. 2022 Nov 23;12:20161. doi: 10.1038/s41598-022-24687-4 (PMC9686226; doi:10.1038/s41598-022-24687-4)
Supplement: Supplementary file 1 — Supplementary Information 1. [file 41598_2022_24687_MOESM1_ESM.zip › raw data/Metabolomics raw data/2.MetAnnotation/2-MetAnnotation-readme.pdf]

MetAnnotation Readme

|-- 2. MetAnnotation　　【代谢物注释结果目录】
| -- **KEGG**　　【基于 KEGG 数据库注释代谢物通路结果】
| |--meta_{pos,neg,all}_kegg_anno_category.xls　【KEGG 通路注释结果】
| |--meta_{pos,neg,all}.KEGG.Anno. {png,pdf}【KEGG 注释结果统计图】
| |---meta_{pos,neg,all}.KEGG.Anno.xls　【代谢物 KEGG 注释结果列表】
| -- **HMDB**　　【基于 HMDB 数据库注释代谢物分类结果】
| |--meta_{pos,neg,all}_hmdb_anno_category.xls【HMDB 分类注释结果】
| |--meta_{pos,neg,all}.HMDB.Anno.{png,pdf}【HMDB 分类注释结果统计图】
| |--meta_{pos,neg,all}.HMDB.Anno.xls　【代谢物 HMDB 注释结果列表】
| -- **Lipidmaps**　　【基于 Lipidmaps 数据库注释代谢物分类结果】
| |--meta_{pos,neg,all}_lipidmaps_anno_category.xls【Lipidmaps 分类注释结果】
| |--meta_{pos,neg,all}.Lipidmaps.Anno.{png,pdf}【Lipidmaps 分类注释结果统计图】
| |--meta_{pos,neg,all}.Lipidmaps.Anno.xls　【代谢物 Lipidmaps 注释结果列表】
| -- **HMDB_KEGG_Lipidmaps**　【基于 KEGG、HMDB、Lipidmaps 数据库注释整合结果】
| --meta_intensity_{pos,neg,all}_hmdb_kegg_lipidmaps.xls【总 HMDB、KEGG、Lipimaps 数据库注释整合结果】

KEGG

meta_{pos,neg,all}_kegg_anno_category.xls

第一列：KO_Pathway_Level1，KEGG 数据库相应 pathway 的第一层级名称；
第二列：KO_Pathway_Level2，KEGG 数据库相应 pathway 的第二层级名称；
第三列：Meta_Num，对应第二层级中注释到的代谢物数目；
第四列：Metabolites，代谢物 ID

meta_{pos,neg,all}.KEGG.Anno. {png,pdf}

横坐标代表代谢物数目，纵坐标代表注释到的 KEGG 通路；该图展示的是 pathway 一级分类各二级分类对应的代谢物数目。

meta_{pos,neg,all}_kegg_anno.xls

第一列：Compound_ID/ID，代谢物 ID；
第二列：Name，代谢物英文名称；
第三列：Kegg_ID，注释到的 kegg 数据库中对应的 ID；
第四列：Kegg_name，代谢物在 kegg 数据库中对应的名称；
第五列：formula，代谢物分子式；
第六列：Kegg_map，代谢通路对应的 mapID 和名称；

HMDB

meta_{pos,neg,all}_hmdb_anno_category.xls

第一列：SuperClass，HMDB 中的二级分类，如：有机酸、脂类等类别；
第二列：Metabolites_nums，该分类注释到的代谢物数目；
第三列：Metabolites，代谢物 ID；
第四列：Metabolite_Descriptions，代谢物描述信息。

meta_{pos,neg,all}.HMDB.Anno.{png,pdf}

横坐标代表代谢物的数目，纵坐标代表注释到的 HMDB 分类；该图展示 HMDB 中二级分类(SuperClass)对应的代谢物数目。

meta_{pos,neg,all}_hmdb_anno.xls

第一列：Compound_ID/ID，代谢物 ID；
第二列：Name，代谢物英文名称；
第三列：Formula，代谢物分子式；
第四列：HMDB_ID，代谢物在 HMDB 数据库中对应的 ID；
第五列：Kingdom，第一层级分类；
第六列：SuperClass，第二层级分类；
第七列：Class，第三层级分类；
第八列：SubClass，第四级分类；
第九列：DirectParent，化合物的化学类别；
第十列：Source，化合物来源；

Lipidmaps

meta_{pos,neg,all}_lipidmaps_anno_category.xls

第一列：CATEGORY，lipidmaps 分类信息（八大类脂质）；
第二列：MAIN_CLASS，八大类下的主层级分类；
第三列：Metabolites_nums，MAIN_CLASS 层级中注释到的代谢物数目；
第四列：Metabolites，代谢物 ID；
第五列：Metabolite_Descriptions，代谢物描述信息。

meta_{pos,neg,all}.Lipidmaps.Anno.{png,pdf}

横坐标代表代谢物数目，纵坐标代表注释到的 LIPID MAPS 脂质分类；该图展示的是 LIPID MAPS 中 8 大脂质分类（Category）下的主层级分类（Main_Class）对应的（脂质）代谢物数目。

meta_{pos,neg,all}_lipidmaps_anno.xls

第一列：Compound_ID/ID，代谢物 ID；
第二列：Name，代谢物英文名称；
第三列：Formula，代谢物分子式；
第四列：Lipidmaps_ID，Lipidmaps 数据库中对应的 ID；
第五列：COMMON_NAME，常用名；
第五列：SYSTEMATIC_NAME，系统命名；
第六列：SYNONYMS，化合物同义名称；
第七列：CATEGORY，分类信息（八大类脂质）；
第八列：MAIN_CLASS，八大类下的主层级分类；
第九列：SUB_CLASS，主层级分类下的子类；

meta_intensity_{pos,neg,all}_hmdb_kegg_lipidmaps.xls

第一列：Compound_ID/ID，代谢物 ID；
第二列：Name，代谢物英文名称；
第三列：Other_name(Kegg_name)，kegg 中该代谢物的其他命名；
第四列：Formula，代谢物分子式；
第五列：Molecular Weight，代谢物相对分子量；
第六列：RT [min]，代谢物保留时间；

第七列：KEGG ID，注释到的 kegg 数据库中对应的 ID；

第八列：KEGG_pathway，代谢通路对应的 mapID 和名称；

第九列：HMDB_ID，代谢物在 HMDB 数据库中对应的 ID；

第十列：Lipidmaps_ID，Lipidmaps 数据库中对应的 ID；

第十一列：SuperClass(HMDB)，第二层级分类；

第十二列：Class(HMDB)，第三层级分类；

第十三列：SubClass(HMDB)，第四级分类；

第十四列：CATEGORY(Lipidmaps)，分类信息（八大类脂质）；

第十五列：MAIN_CLASS(Lipidmaps)，八大类下的主层级分类；

第十六列：SUB_CLASS(Lipidmaps)，主层级分类下的子类；

第十七列：mzCloud_Results，匹配结果来源于 mzCloud 数据库；

第十八列：mzVault_Results，匹配结果来源于 mzVault 数据库；

第十九列：MassList_Results，匹配结果来源于 MassList 数据库，匹配结果解释详情，请查阅结题报告名词解释附件；

第二十列～：不同实验样品质谱检测的定量值（峰面积值）；
