# Supplementary material for: Comparative multiomics analysis of cell physiological state after culture in a basket bioreactor
Source: Sci Rep. 2022 Nov 23;12:20161. doi: 10.1038/s41598-022-24687-4 (PMC9686226; doi:10.1038/s41598-022-24687-4)
Supplement: Supplementary file 1 — Supplementary Information 1. [file 41598_2022_24687_MOESM1_ESM.zip › raw data/Metabolomics raw data/2.MetAnnotation/HMDB/meta_neg.HMDB.Anno.pdf]

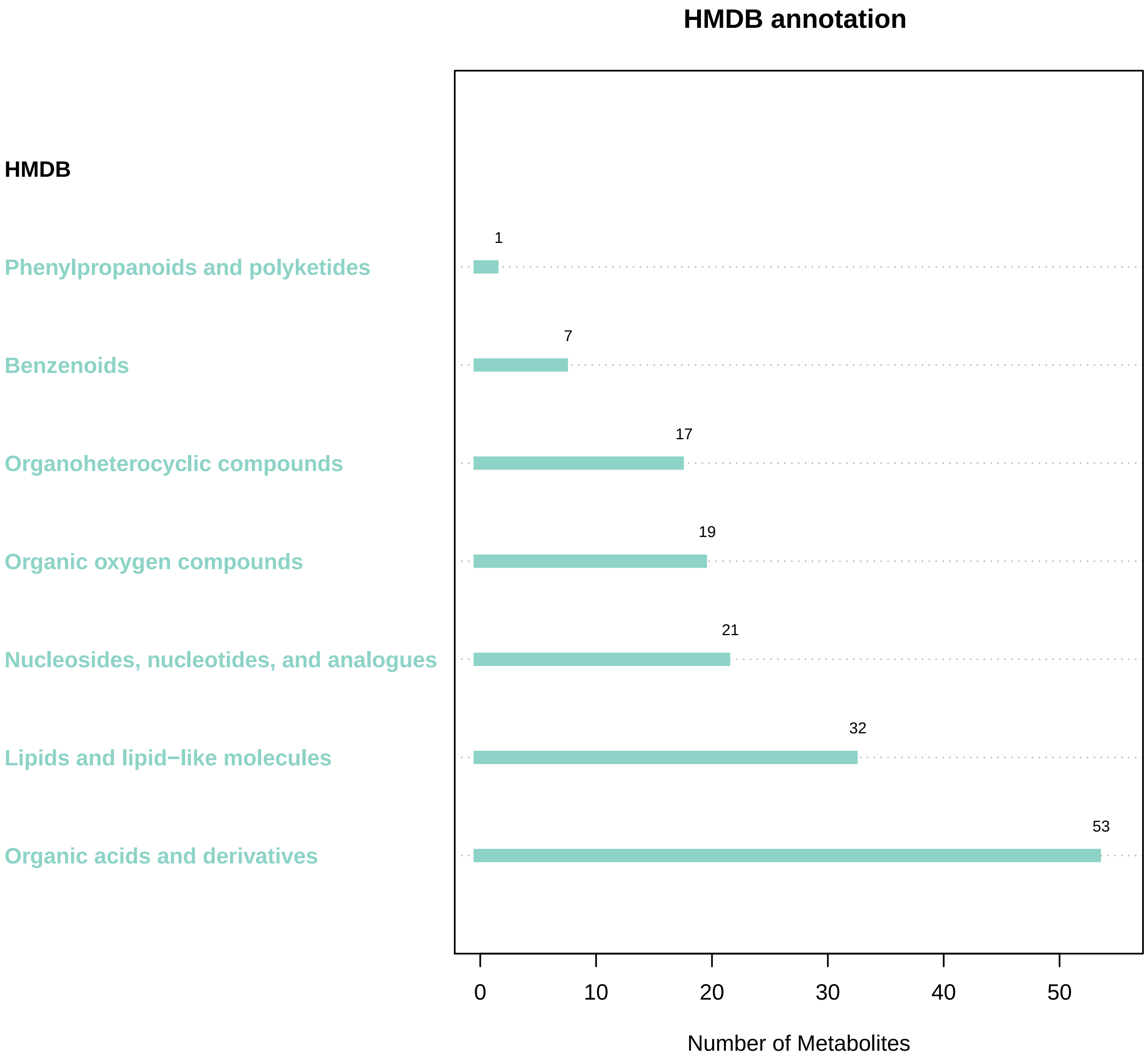
HMDB annotation
HMDB
Phenylpropanoids and polyketides
Benzenoids
Organoheterocyclic compounds
Organic oxygen compounds
Nucleosides, nucleotides, and analogues
Lipids and lipid−like molecules
Organic acids and derivatives
1
7
17
19
21
32
53
0
10
20
30
40
50
Number of Metabolites
