# Supplementary material for: Comparative multiomics analysis of cell physiological state after culture in a basket bioreactor
Source: Sci Rep. 2022 Nov 23;12:20161. doi: 10.1038/s41598-022-24687-4 (PMC9686226; doi:10.1038/s41598-022-24687-4)
Supplement: Supplementary file 1 — Supplementary Information 1. [file 41598_2022_24687_MOESM1_ESM.zip › raw data/Metabolomics raw data/2.MetAnnotation/KEGG/meta_neg.KEGG.Anno.pdf]

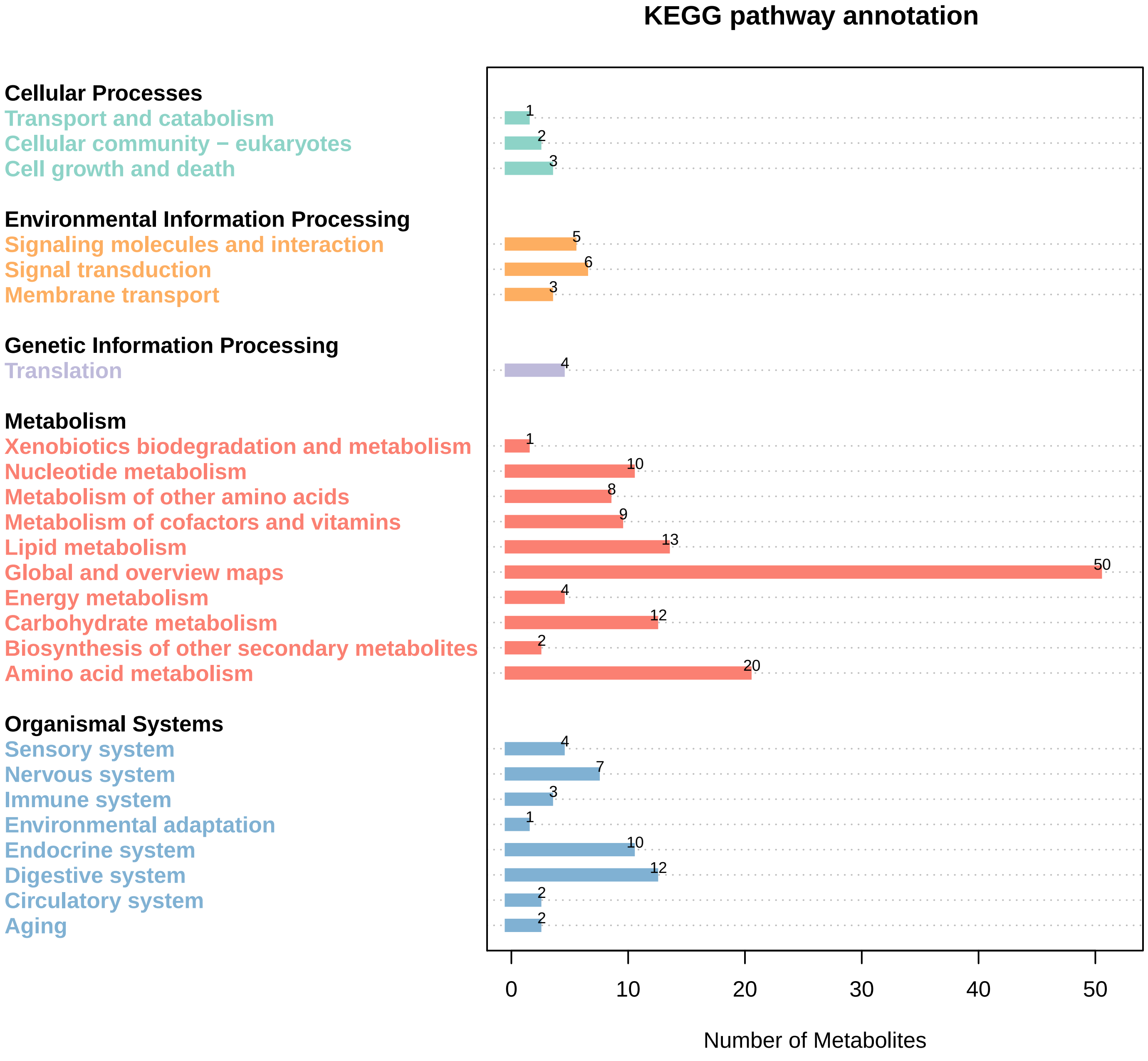

KEGG pathway annotation
Cellular Processes
Transport and catabolism
Cellular community − eukaryotes
Cell growth and death
Environmental Information Processing
Signaling molecules and interaction
Signal transduction
Membrane transport
Genetic Information Processing
Translation
Metabolism
Xenobiotics biodegradation and metabolism
Nucleotide metabolism
Metabolism of other amino acids
Metabolism of cofactors and vitamins
Lipid metabolism
Global and overview maps
Energy metabolism
Carbohydrate metabolism
Biosynthesis of other secondary metabolites
Amino acid metabolism
Organismal Systems
Sensory system
Nervous system
Immune system
Environmental adaptation
Endocrine system
Digestive system
Circulatory system
Aging
1
2
3
5
6
3
4
1
10
8
9
13
50
4
12
2
20
4
7
3
1
10
12
2
2
0
10
20
30
40
50
Number of Metabolites
