# Supplementary material for: Comparative multiomics analysis of cell physiological state after culture in a basket bioreactor
Source: Sci Rep. 2022 Nov 23;12:20161. doi: 10.1038/s41598-022-24687-4 (PMC9686226; doi:10.1038/s41598-022-24687-4)
Supplement: Supplementary file 1 — Supplementary Information 1. [file 41598_2022_24687_MOESM1_ESM.zip › raw data/Metabolomics raw data/2.MetAnnotation/Lipidmaps/meta_neg.Lipidmaps.Anno.pdf]

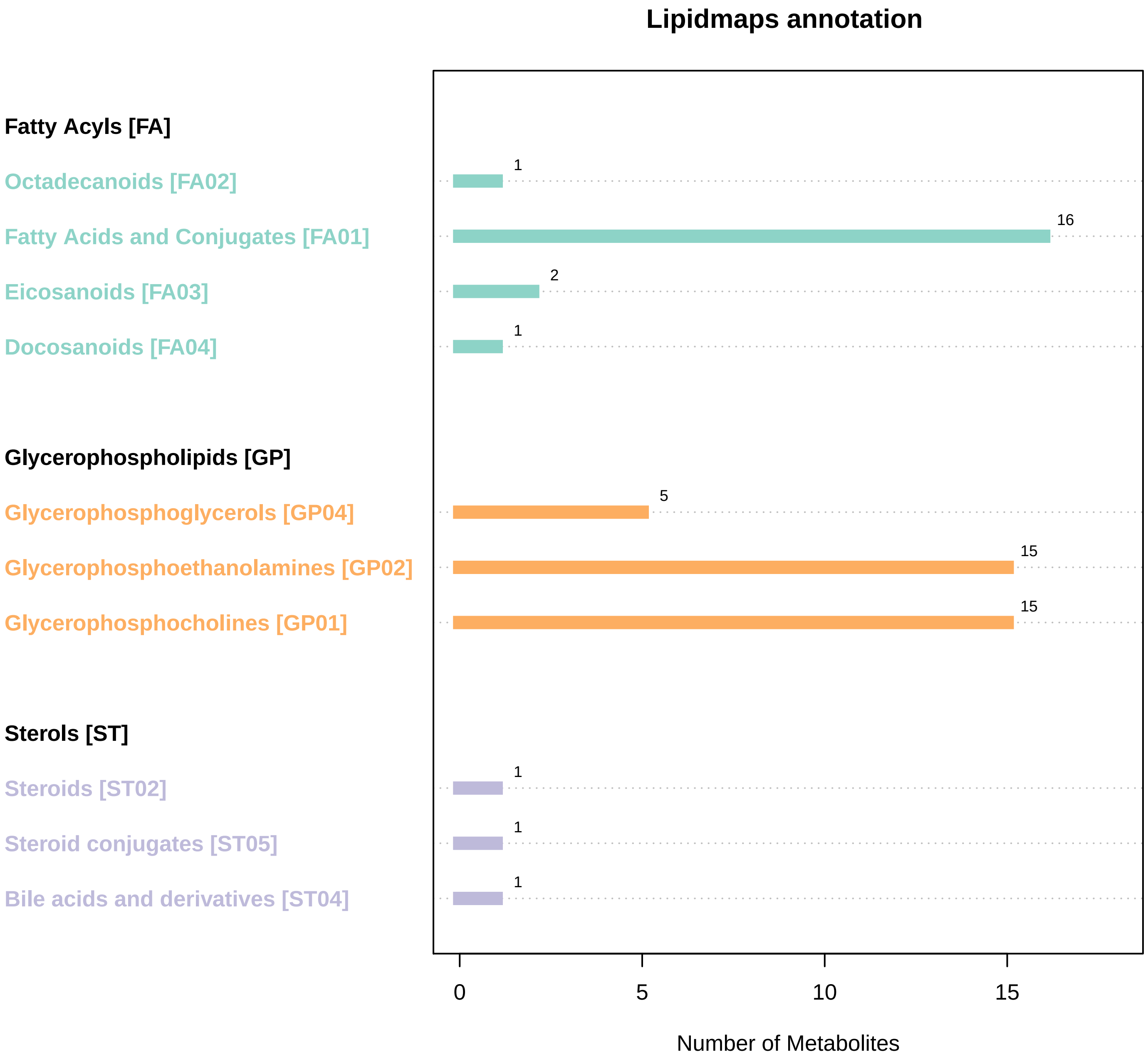
Lipidmaps annotation
Fatty Acyls [FA]
Octadecanoids [FA02]
Fatty Acids and Conjugates [FA01]
Eicosanoids [FA03]
Docosanoids [FA04]
Glycerophospholipids [GP]
Glycerophosphoglycerols [GP04]
Glycerophosphoethanolamines [GP02]
Glycerophosphocholines [GP01]
Sterols [ST]
Steroids [ST02]
Steroid conjugates [ST05]
Bile acids and derivatives [ST04]
1
16
2
1
5
15
15
1
1
1
0
5
10
15
Number of Metabolites
