# Supplementary material for: Comparative multiomics analysis of cell physiological state after culture in a basket bioreactor
Source: Sci Rep. 2022 Nov 23;12:20161. doi: 10.1038/s41598-022-24687-4 (PMC9686226; doi:10.1038/s41598-022-24687-4)
Supplement: Supplementary file 1 — Supplementary Information 1. [file 41598_2022_24687_MOESM1_ESM.zip › raw data/Metabolomics raw data/2.MetAnnotation/Lipidmaps/meta_pos.Lipidmaps.Anno.pdf]

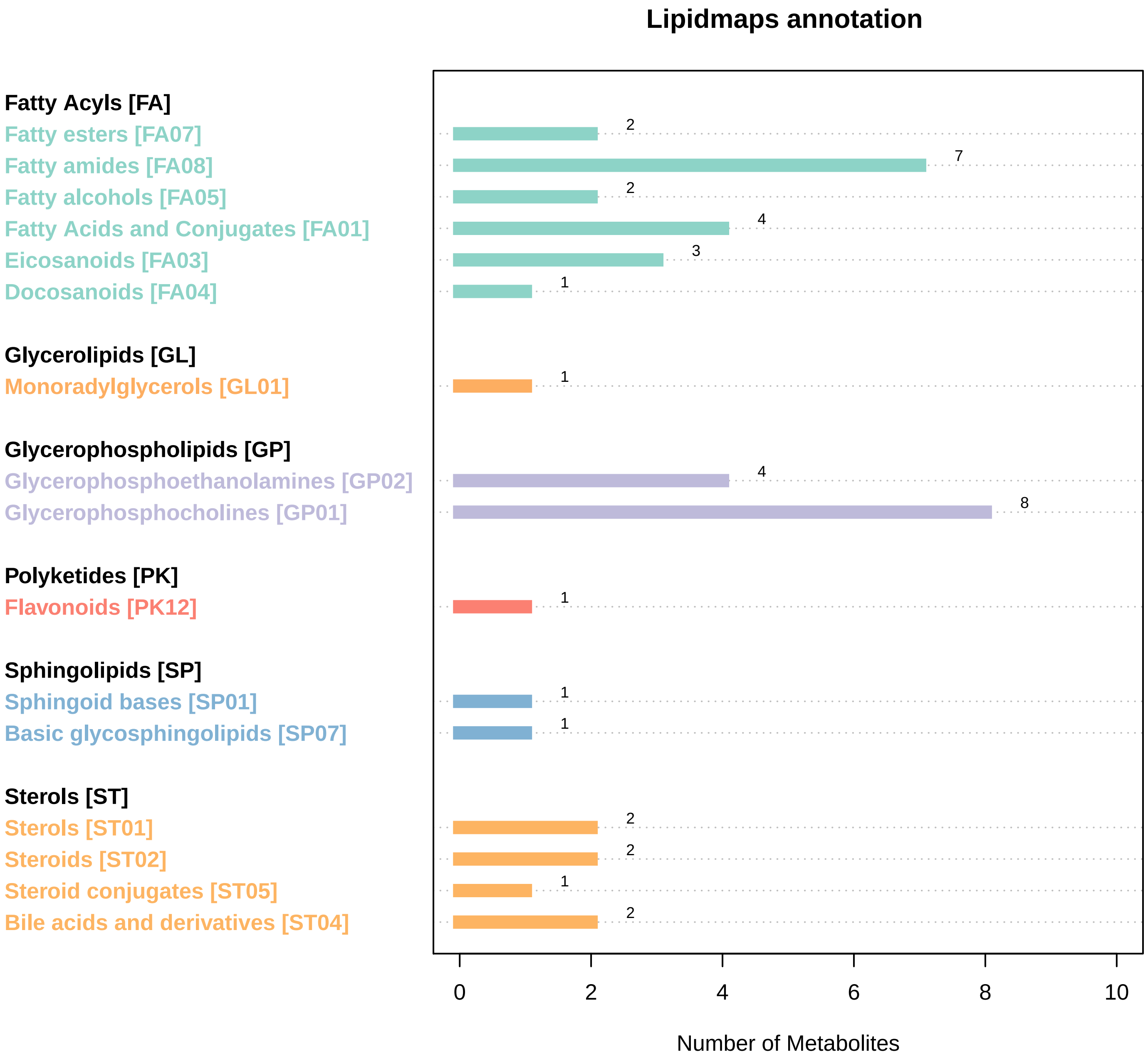

Lipidmaps annotation
Fatty Acyls [FA]
Fatty esters [FA07]
Fatty amides [FA08]
Fatty alcohols [FA05]
Fatty Acids and Conjugates [FA01]
Eicosanoids [FA03]
Docosanoids [FA04]
Glycerolipids [GL]
Monoradylglycerols [GL01]
Glycerophospholipids [GP]
Glycerophosphoethanolamines [GP02]
Glycerophosphocholines [GP01]
Polyketides [PK]
Flavonoids [PK12]
Sphingolipids [SP]
Sphingoid bases [SP01]
Basic glycosphingolipids [SP07]
Sterols [ST]
Sterols [ST01]
Steroids [ST02]
Steroid conjugates [ST05]
Bile acids and derivatives [ST04]
2
7
2
4
3
1
1
4
8
1
1
1
2
2
1
2
0
2
4
6
8
10
Number of Metabolites
