# Supplementary material for: Comparative multiomics analysis of cell physiological state after culture in a basket bioreactor
Source: Sci Rep. 2022 Nov 23;12:20161. doi: 10.1038/s41598-022-24687-4 (PMC9686226; doi:10.1038/s41598-022-24687-4)
Supplement: Supplementary file 1 — Supplementary Information 1. [file 41598_2022_24687_MOESM1_ESM.zip › raw data/Metabolomics raw data/4.MetDiffAnalysis/4-MetDiffAnalysis-readme.pdf]

MetDiffAnalysis Readme

|-- 4.MetDiffAnalysis 【差异代谢物分析结果目录】

|| -- Heatmap_diff 【总差异代谢物热图结果】

| |--Diff_Heatmap_{pos,neg,all}{_cluster,cluster_detail}.{png,pdf} 【总差异代谢物聚类热图】

| |--Diff_Heatmap_{pos,neg,all}{,_detail}.{png,pdf} 【总差异代谢物不聚类热图】

| -- *.vs.* 【比较的样本对目录】

| |-- *.vs.*_{pos,neg,all}.corr.xls 【差异代谢物相关性列表】

| |-- *.vs.*_{pos,neg,all}_Pvalue.xls 【基于相关性分析的 p 值列表】

| |-- *.vs.*_{pos,neg,all}_zscore.xls 【差异代谢物 zscore 分析列表】

| |-- *.vs.*_{pos,neg,all}_cluster_heatmap[,_detail].{png,pdf} 【差异代谢物聚类热图】

| |-- *.vs.*_{pos,neg,all}_heatmap[._detail].{png,pdf} 【差异代谢物不聚类热图】

| |-- *.vs.*_{pos,neg,all}_corr.{png,pdf} 【差异代谢物相关性分析图】

| |-- *.vs.*_{pos,neg,all}_zscore.{png,pdf} 【差异代谢物 z-score 图】

| |-- ROC_{pos,neg,all} 【差异代谢物 ROC 曲线图】

***.vs.*_{pos,neg,all}_corr.xls**

代谢物相关性分析表格；

***.vs.*_{pos,neg,all}_Pvalue.xls**

基于代谢物相关性分析的 P 值；

***.vs.*_{pos,neg,all}_zscore.xls**

代谢物 zscore 值表格；

Diff_Heatmap_{pos,neg,all}{_cluster,cluster_detail}.{png,pdf}

总差异代谢物聚类热图：对各比较对之间的差异代谢物进行层次聚类分析，将差异代谢物相对定量值进行归一化转换并聚类。横向为代谢物的聚类，纵向为样本分组，聚类枝越短代表相似性越高。

Diff_Heatmap_{pos,neg,all}{,_detail}.{png,pdf}

总差异代谢物不聚类热图：将差异代谢物相对定量值进行归一化转换。横向为代谢物的聚类，纵向为样本分组。

***.vs.* _{pos,neg,all}_cluster_heatmap[,_detail].{png,pdf}**

差异代谢物聚类热图：对两组样本获得的差异代谢物进行层次聚类分析，得出同一比较对两组之间和组内代谢表达模式的差异情况。横向为代谢物的聚类，纵向为样本类型，聚类枝越短代表相似性越高。

***.vs.*_ {pos,neg,all}_heatmap[,_detail].{png,pdf}**

差异代谢物不聚类热图：将两组样本获得的差异代谢物相对定量值进行归一化转换，得出同一 比较对两组之间和组内代谢表达模式的差异情况。横向为代谢物的聚类，纵向为样本类型。

***.vs.*_{pos,neg,all}_corr.{png,pdf}**

差异代谢物相关性分析图：通过计算所有差异代谢物两两之间的皮尔逊相关系数，选取显著性水平 P-value 值从小到大排序的 Top20 的差异代谢物进行展示，可以查看

代谢物与代谢物变化趋势的一致性。相关性最高为 1，为完全的正相关（红色），相关性最低为-1，为完全的负相关（蓝色），没有颜色的部分表示 P-value＞0.05。

***.vs.*_{pos,neg,all}_zscore.{png,pdf}**

差异代谢物 z-score 图：z-score（标准分数）是基于代谢物的相对含量转换而来的值，用于衡量同一水平面上代谢物的相对含量的高低。横坐标为 z-score 值，纵坐标为差异代谢物，每个圆圈代表一个样本。图中只展示了 Top30（按 p-value 值从小到大排序）的代谢物 Z-score 值。Z-score 超出 4 或-4 的样本无法展示。

ROC_{pos,neg,all}

差异代谢物 ROC 曲线图：ROC 曲线又叫受试者工作特征曲线或感受性曲线，根据一系列不同的二分类方式（分界值或决定域）绘制的曲线，差异代谢物的 ROC 曲线可用来评判潜在的生物标记物。横坐标为假阳性率（1-特异度），纵坐标为真阳性率（灵敏度）。
