# Supplementary figures and images for: Comparative multiomics analysis of cell physiological state after culture in a basket bioreactor (part 2 of 2)
Source: Sci Rep. 2022 Nov 23;12:20161. doi: 10.1038/s41598-022-24687-4 (PMC9686226; doi:10.1038/s41598-022-24687-4)

# EG.vs.CG

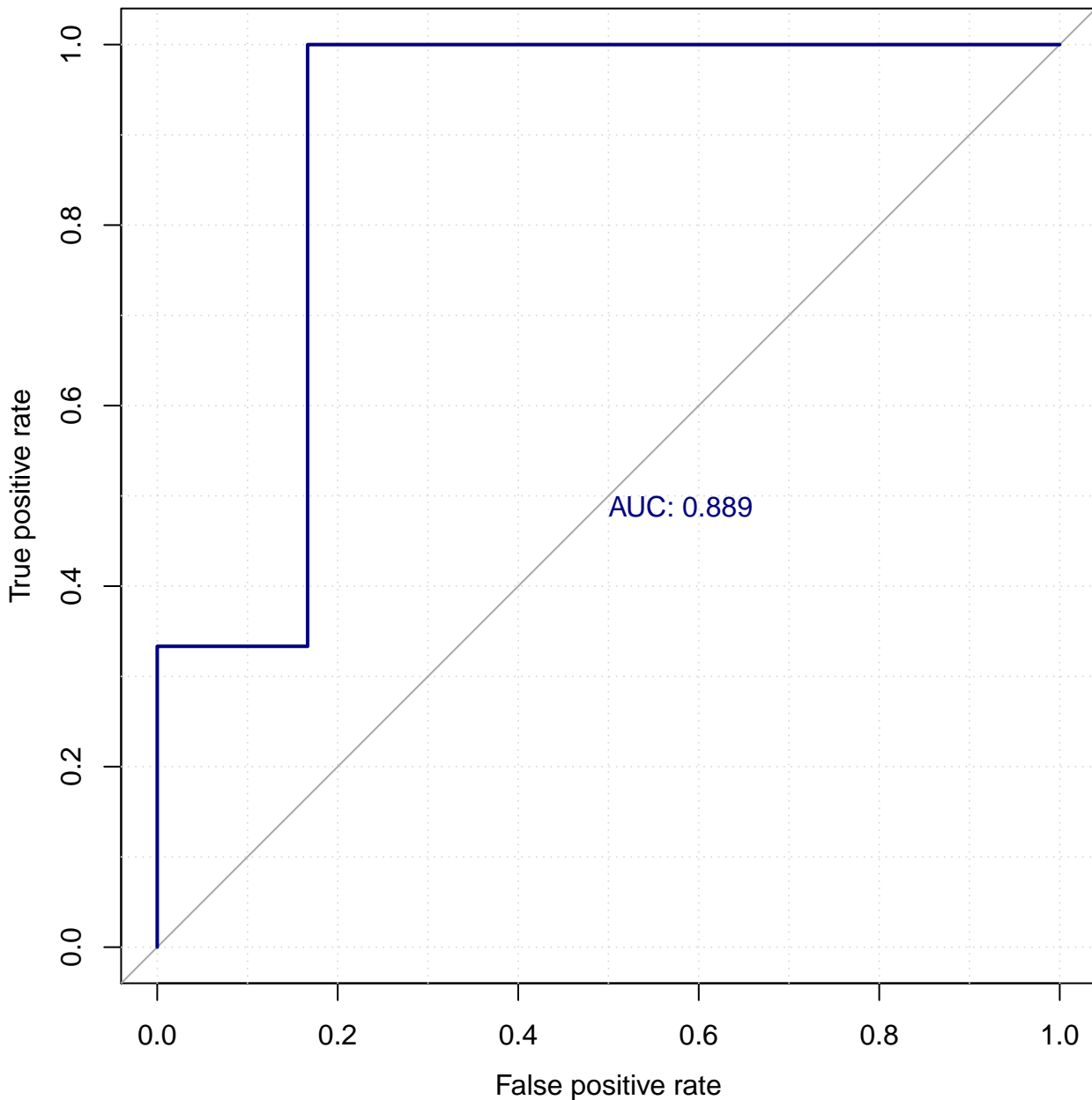

Supplement: Supplementary file 1 — Supplementary Information 1. [file 41598_2022_24687_MOESM1_ESM.zip › raw data/Metabolomics raw data/4.MetDiffAnalysis/EG.vs.CG/ROC_neg/Com_12752_neg_ROC.pdf]

# EG.vs.CG

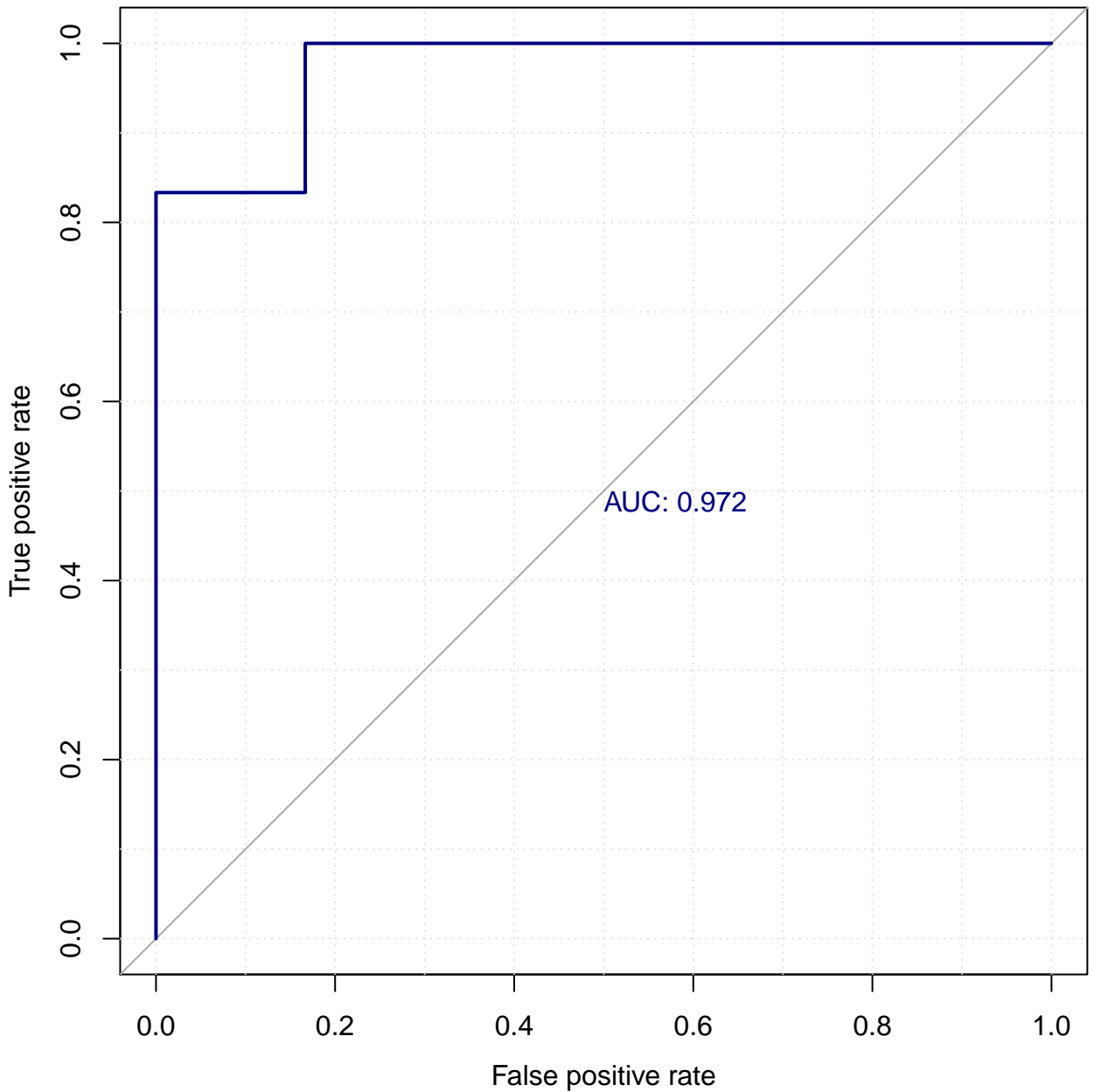

Supplement: Supplementary file 1 — Supplementary Information 1. [file 41598_2022_24687_MOESM1_ESM.zip › raw data/Metabolomics raw data/4.MetDiffAnalysis/EG.vs.CG/ROC_neg/Com_12_neg_ROC.pdf]

# EG.vs.CG

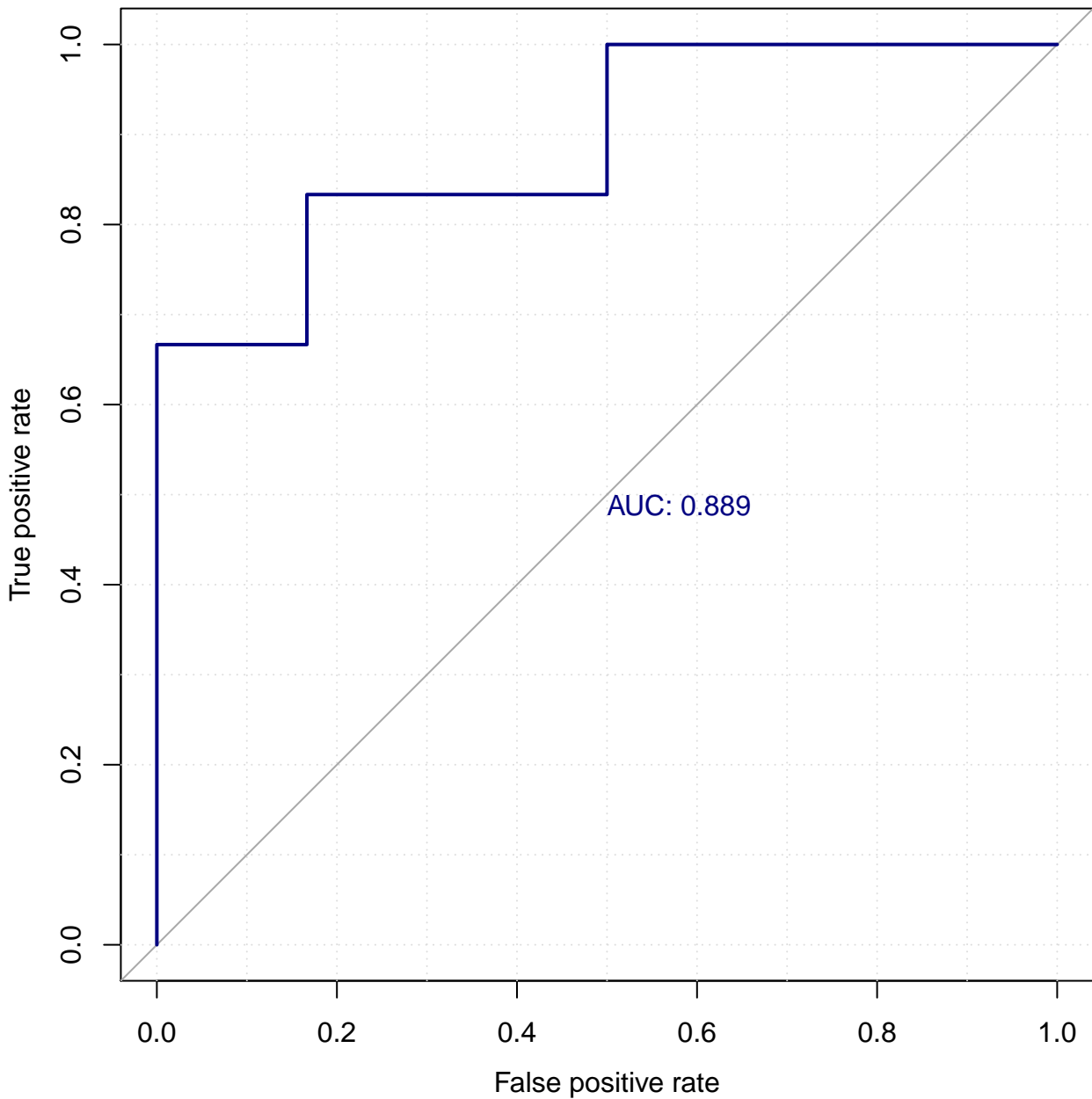

Supplement: Supplementary file 1 — Supplementary Information 1. [file 41598_2022_24687_MOESM1_ESM.zip › raw data/Metabolomics raw data/4.MetDiffAnalysis/EG.vs.CG/ROC_neg/Com_13137_neg_ROC.pdf]

# EG.vs.CG

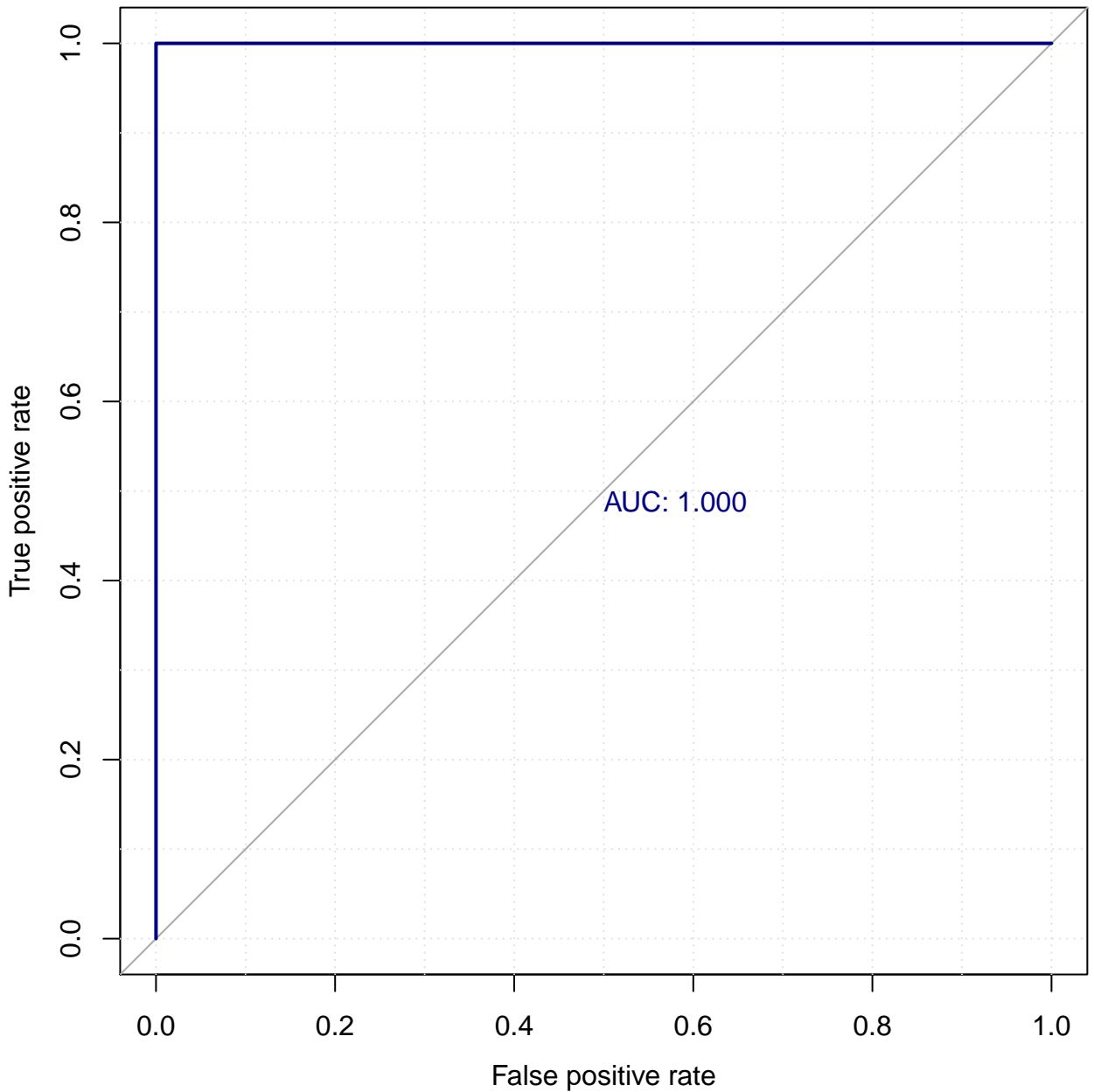

Supplement: Supplementary file 1 — Supplementary Information 1. [file 41598_2022_24687_MOESM1_ESM.zip › raw data/Metabolomics raw data/4.MetDiffAnalysis/EG.vs.CG/ROC_neg/Com_1314_neg_ROC.pdf]

# EG.vs.CG

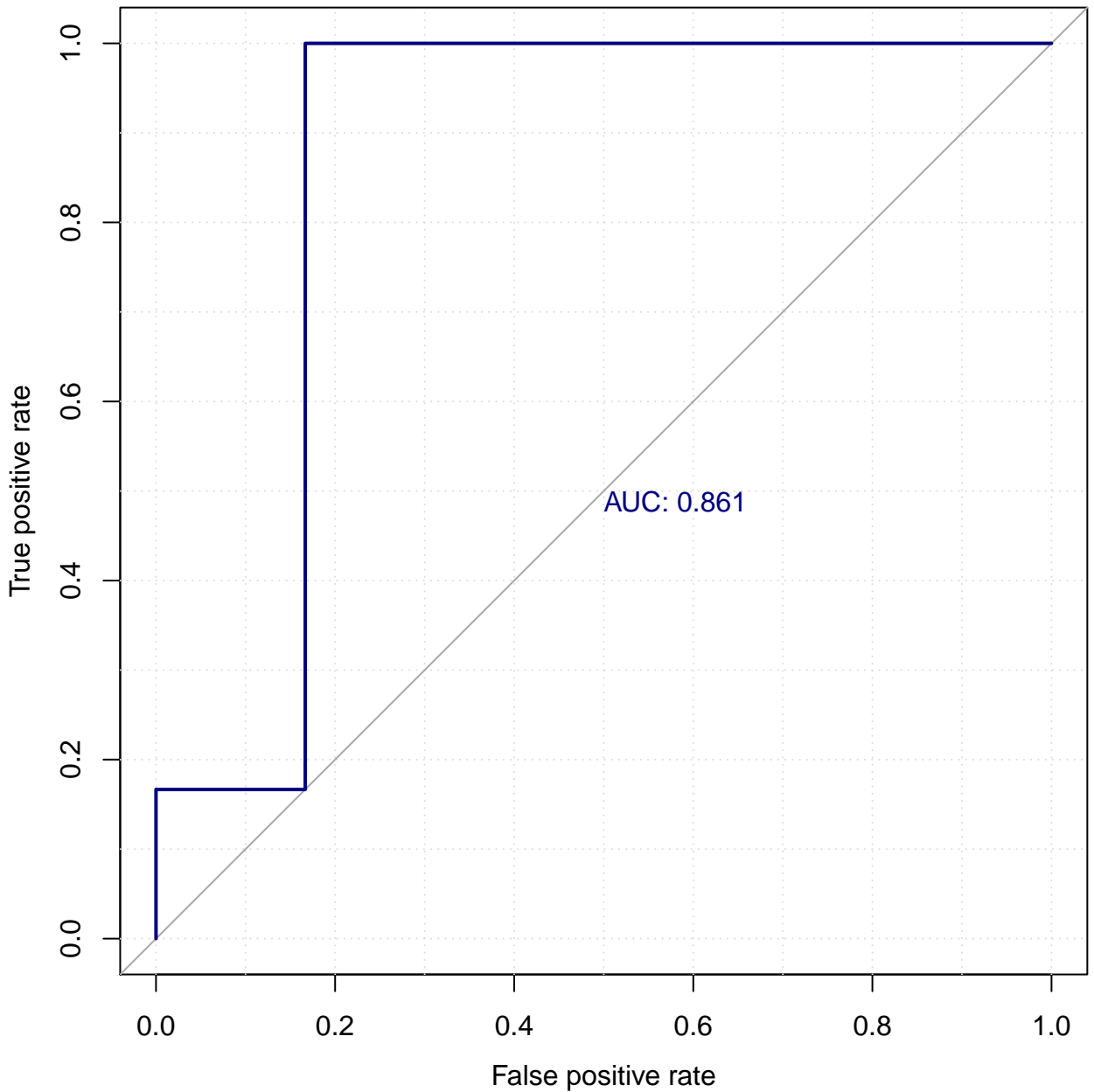

Supplement: Supplementary file 1 — Supplementary Information 1. [file 41598_2022_24687_MOESM1_ESM.zip › raw data/Metabolomics raw data/4.MetDiffAnalysis/EG.vs.CG/ROC_neg/Com_1316_neg_ROC.pdf]

# EG.vs.CG

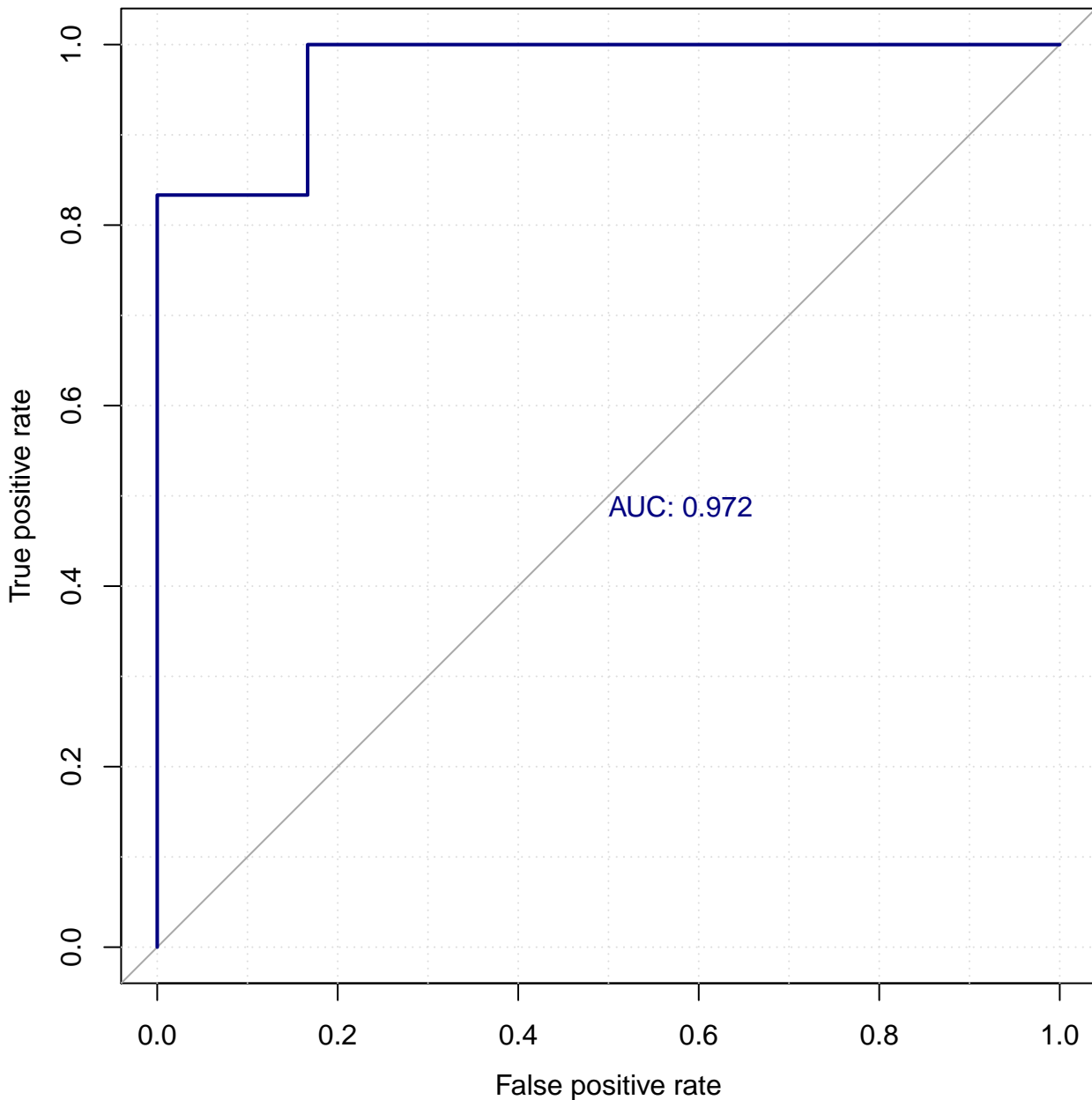

Supplement: Supplementary file 1 — Supplementary Information 1. [file 41598_2022_24687_MOESM1_ESM.zip › raw data/Metabolomics raw data/4.MetDiffAnalysis/EG.vs.CG/ROC_neg/Com_13199_neg_ROC.pdf]

# EG.vs.CG

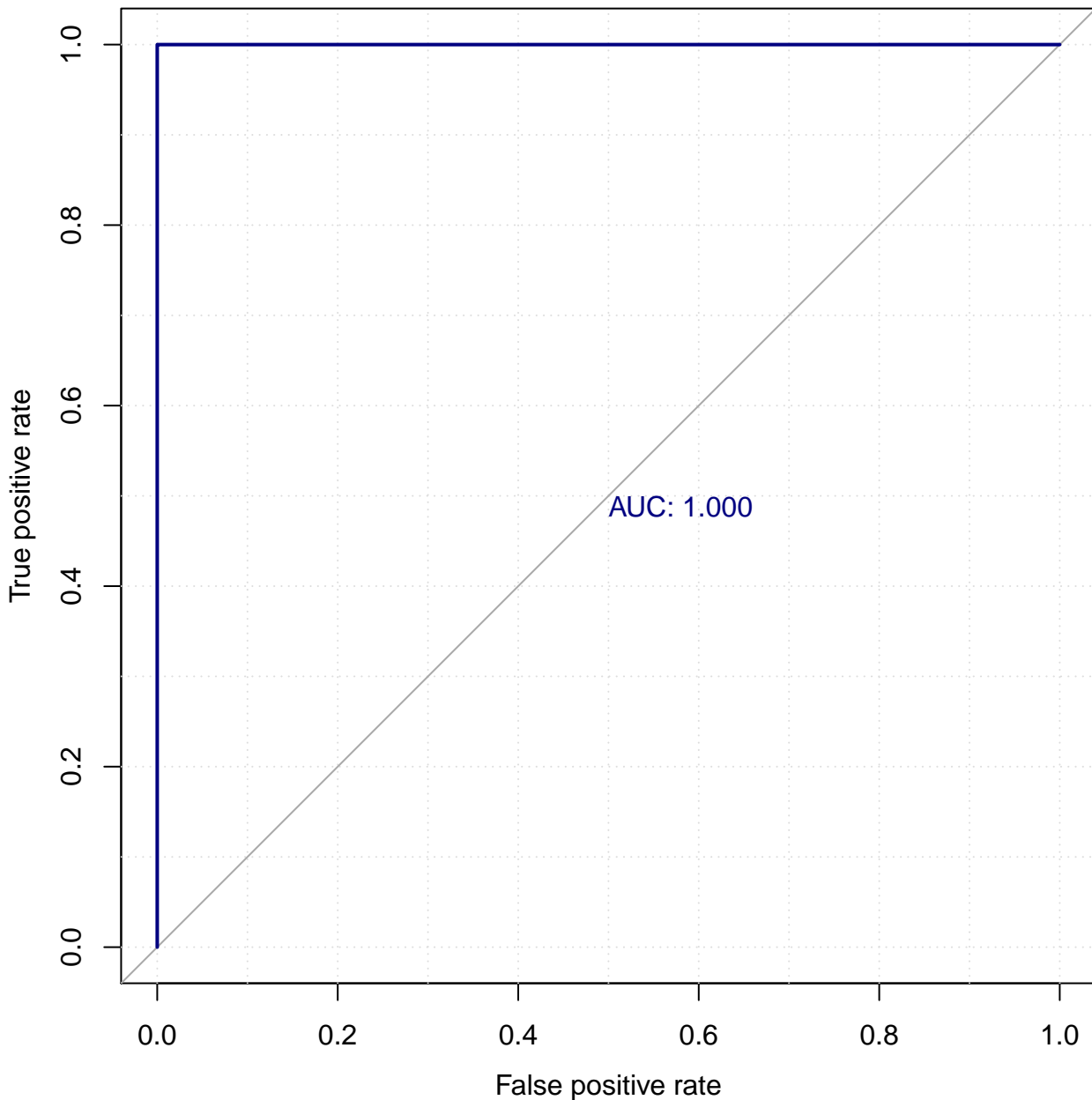

Supplement: Supplementary file 1 — Supplementary Information 1. [file 41598_2022_24687_MOESM1_ESM.zip › raw data/Metabolomics raw data/4.MetDiffAnalysis/EG.vs.CG/ROC_neg/Com_132_neg_ROC.pdf]

# EG.vs.CG

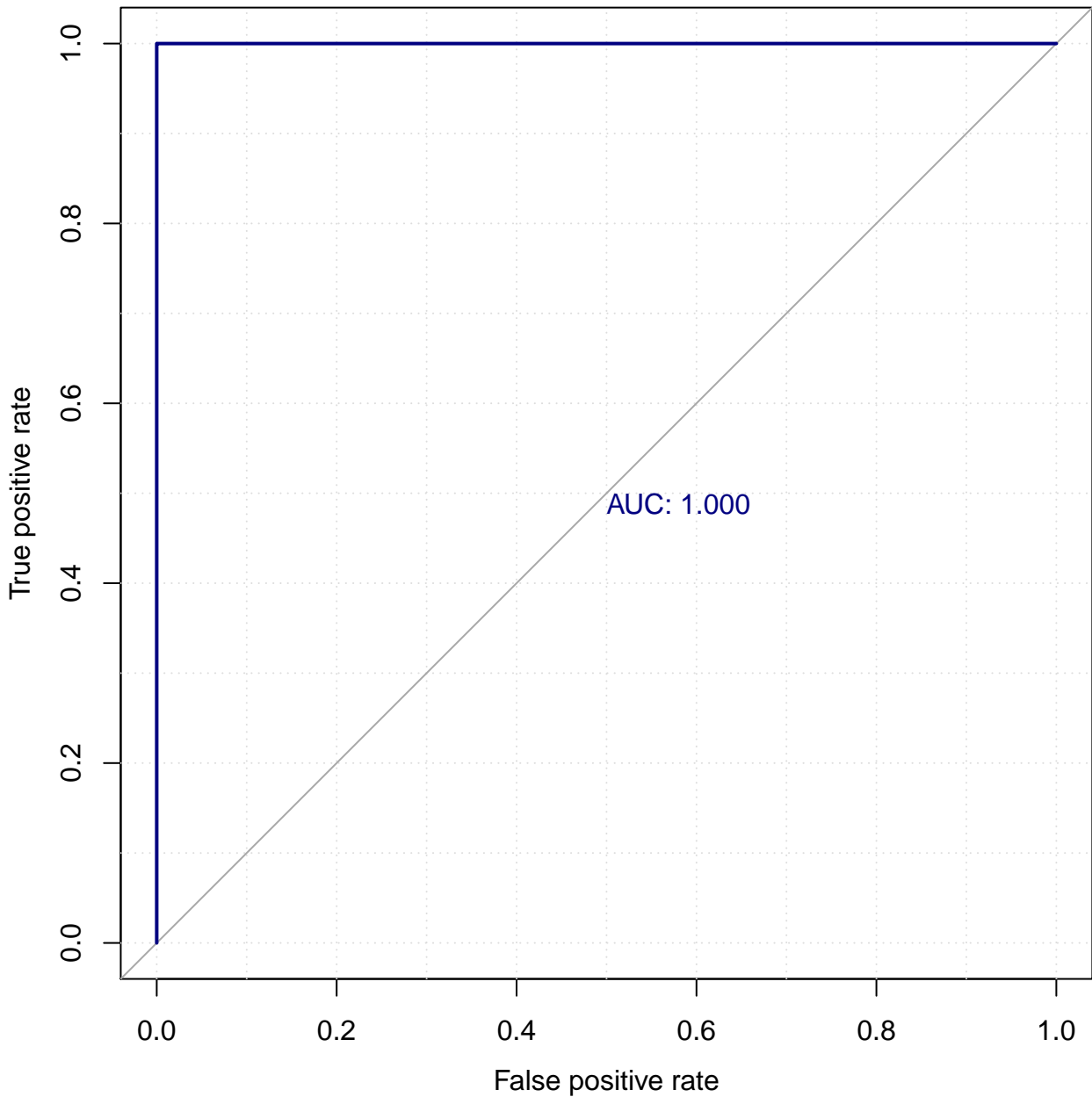

Supplement: Supplementary file 1 — Supplementary Information 1. [file 41598_2022_24687_MOESM1_ESM.zip › raw data/Metabolomics raw data/4.MetDiffAnalysis/EG.vs.CG/ROC_neg/Com_1336_neg_ROC.pdf]

# EG.vs.CG

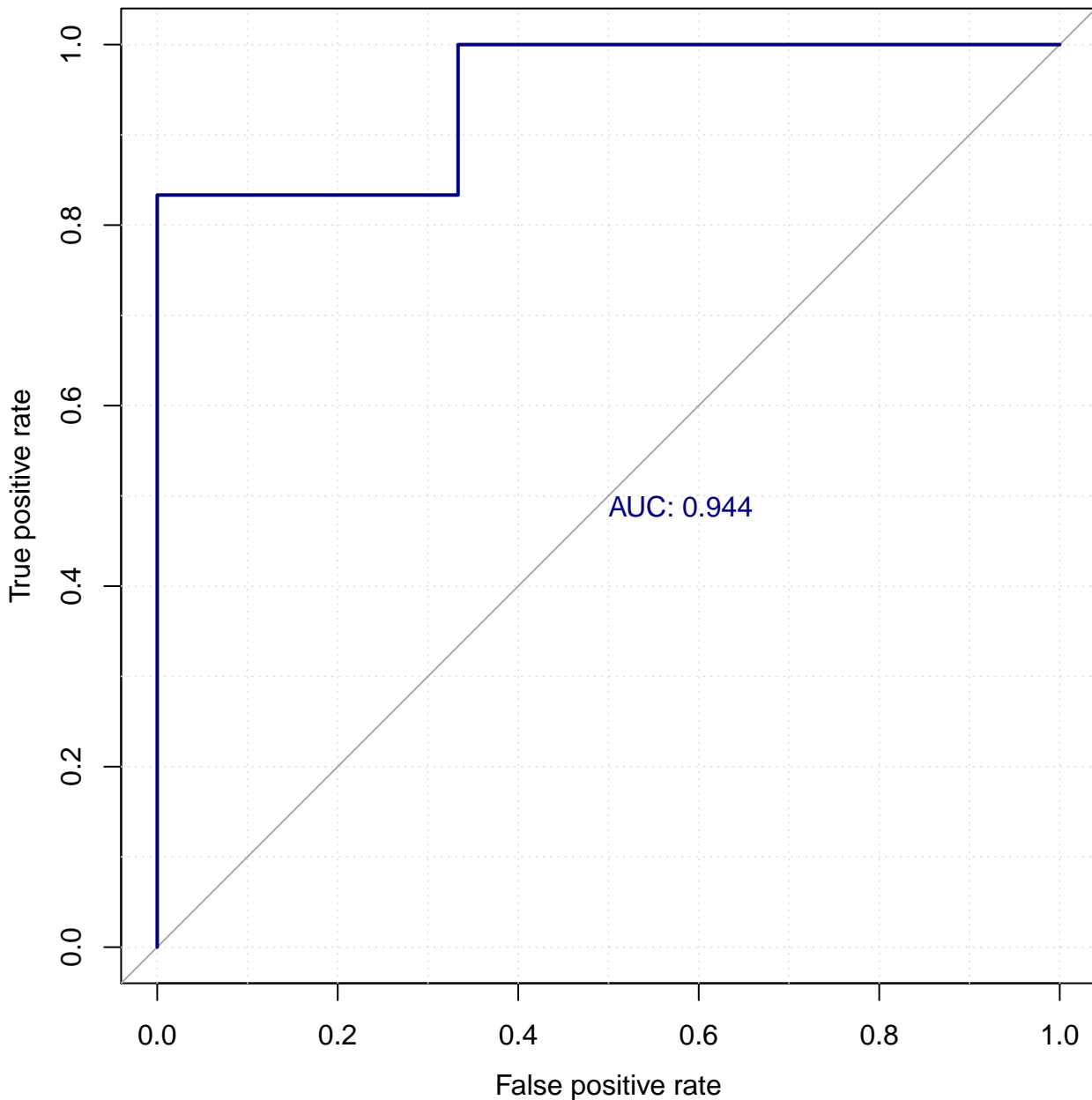

Supplement: Supplementary file 1 — Supplementary Information 1. [file 41598_2022_24687_MOESM1_ESM.zip › raw data/Metabolomics raw data/4.MetDiffAnalysis/EG.vs.CG/ROC_neg/Com_13439_neg_ROC.pdf]

# EG.vs.CG

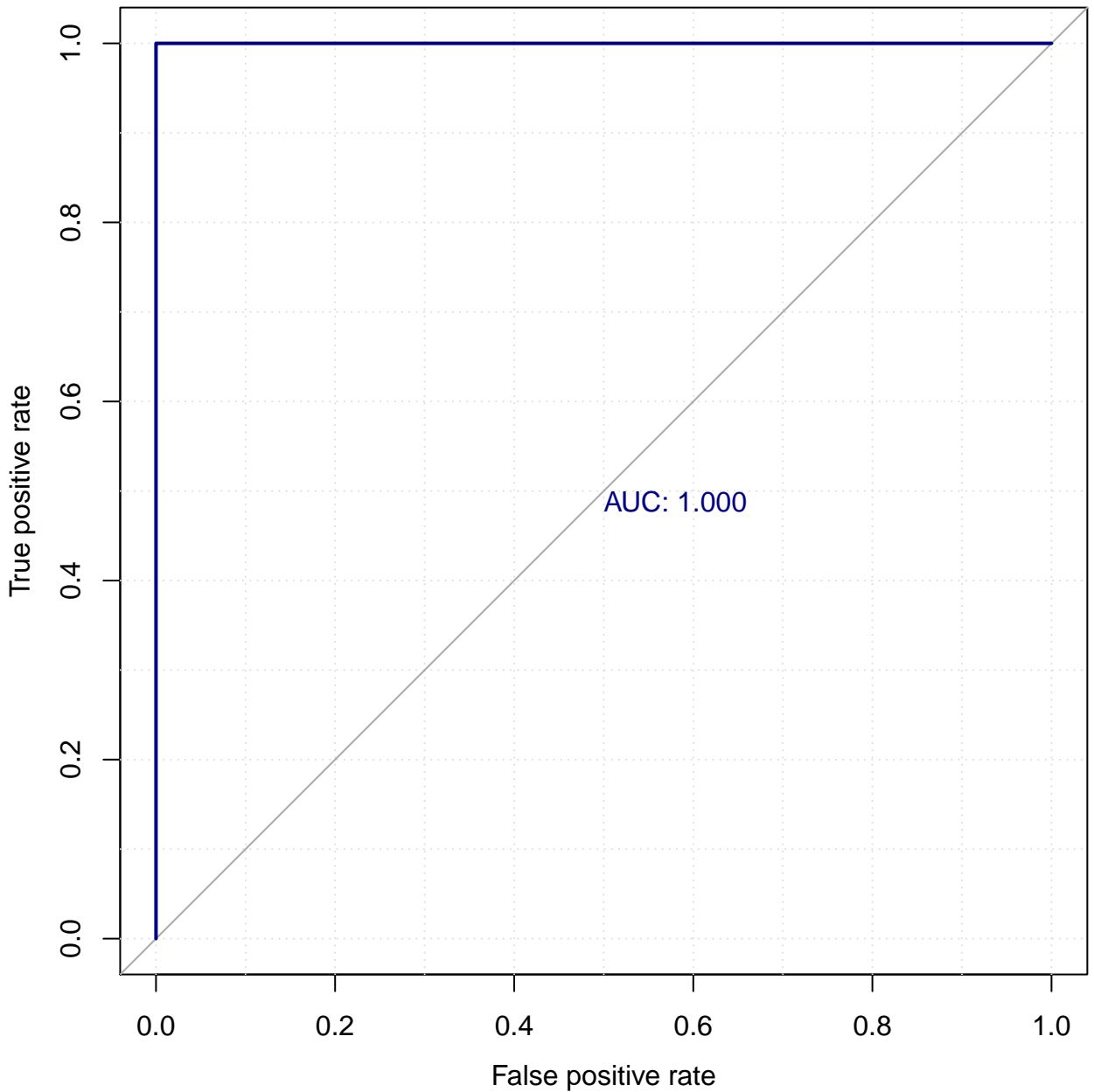

Supplement: Supplementary file 1 — Supplementary Information 1. [file 41598_2022_24687_MOESM1_ESM.zip › raw data/Metabolomics raw data/4.MetDiffAnalysis/EG.vs.CG/ROC_neg/Com_14132_neg_ROC.pdf]

# EG.vs.CG

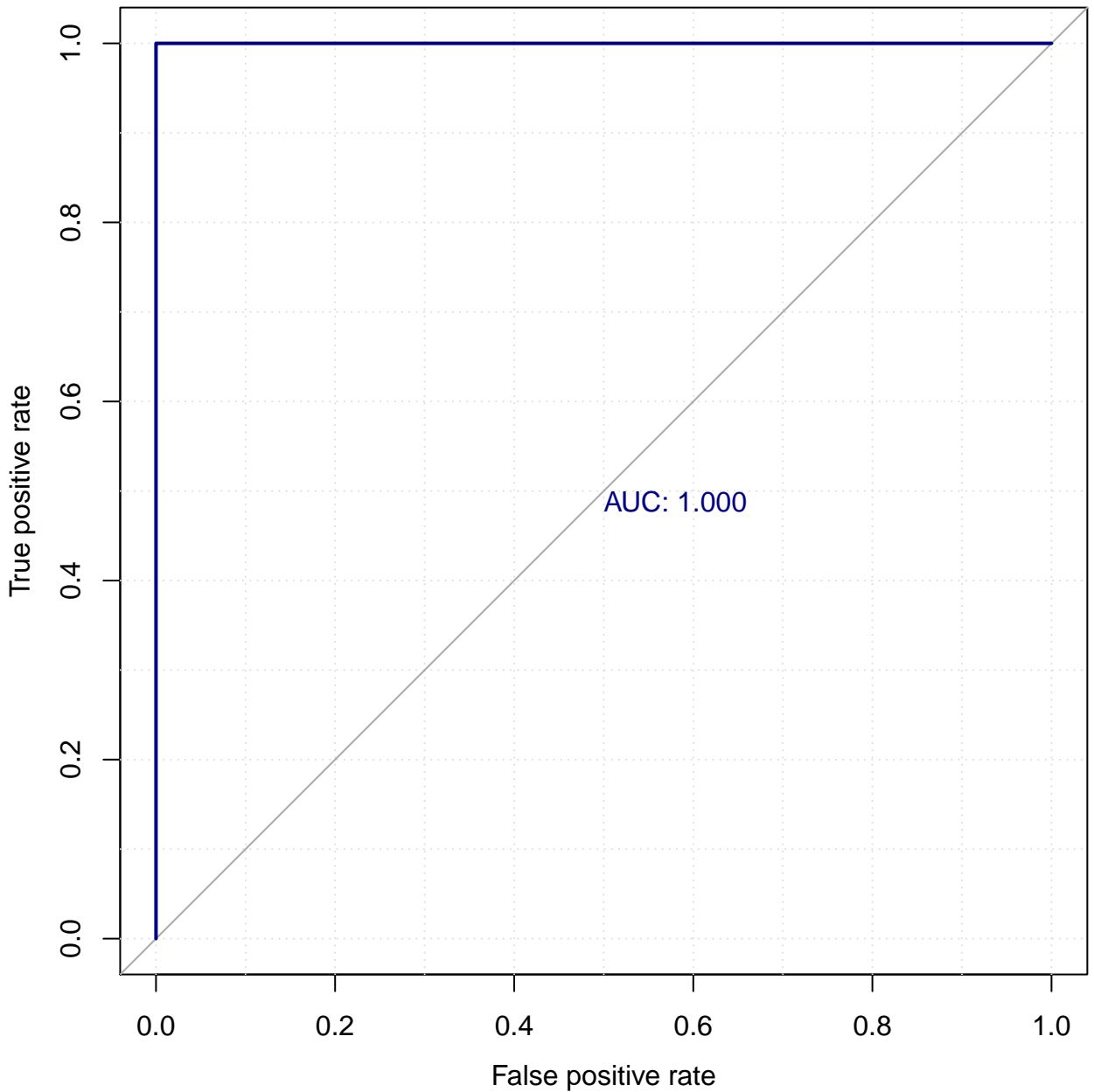

Supplement: Supplementary file 1 — Supplementary Information 1. [file 41598_2022_24687_MOESM1_ESM.zip › raw data/Metabolomics raw data/4.MetDiffAnalysis/EG.vs.CG/ROC_neg/Com_1478_neg_ROC.pdf]

# EG.vs.CG

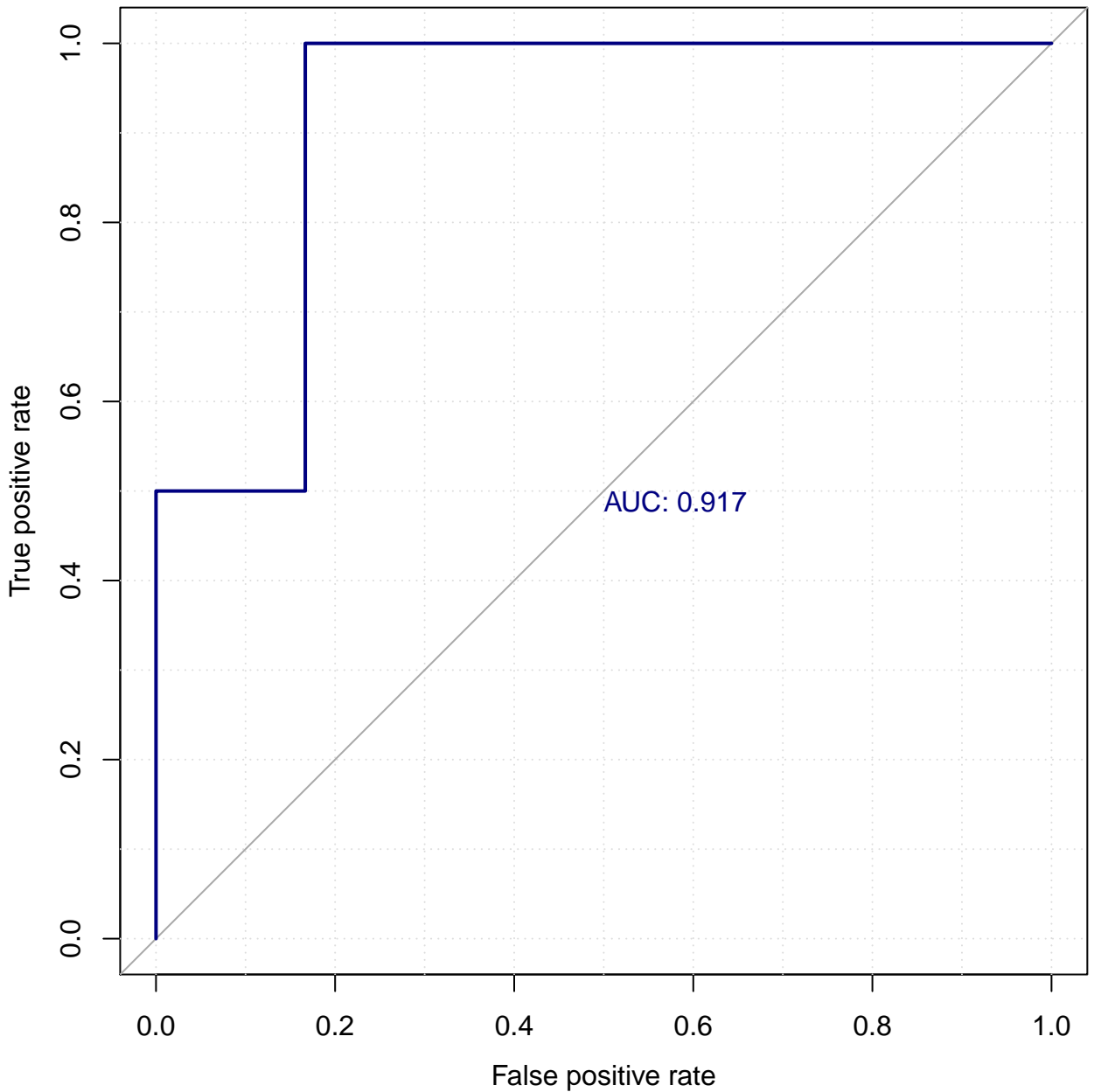

Supplement: Supplementary file 1 — Supplementary Information 1. [file 41598_2022_24687_MOESM1_ESM.zip › raw data/Metabolomics raw data/4.MetDiffAnalysis/EG.vs.CG/ROC_neg/Com_15503_neg_ROC.pdf]

# EG.vs.CG

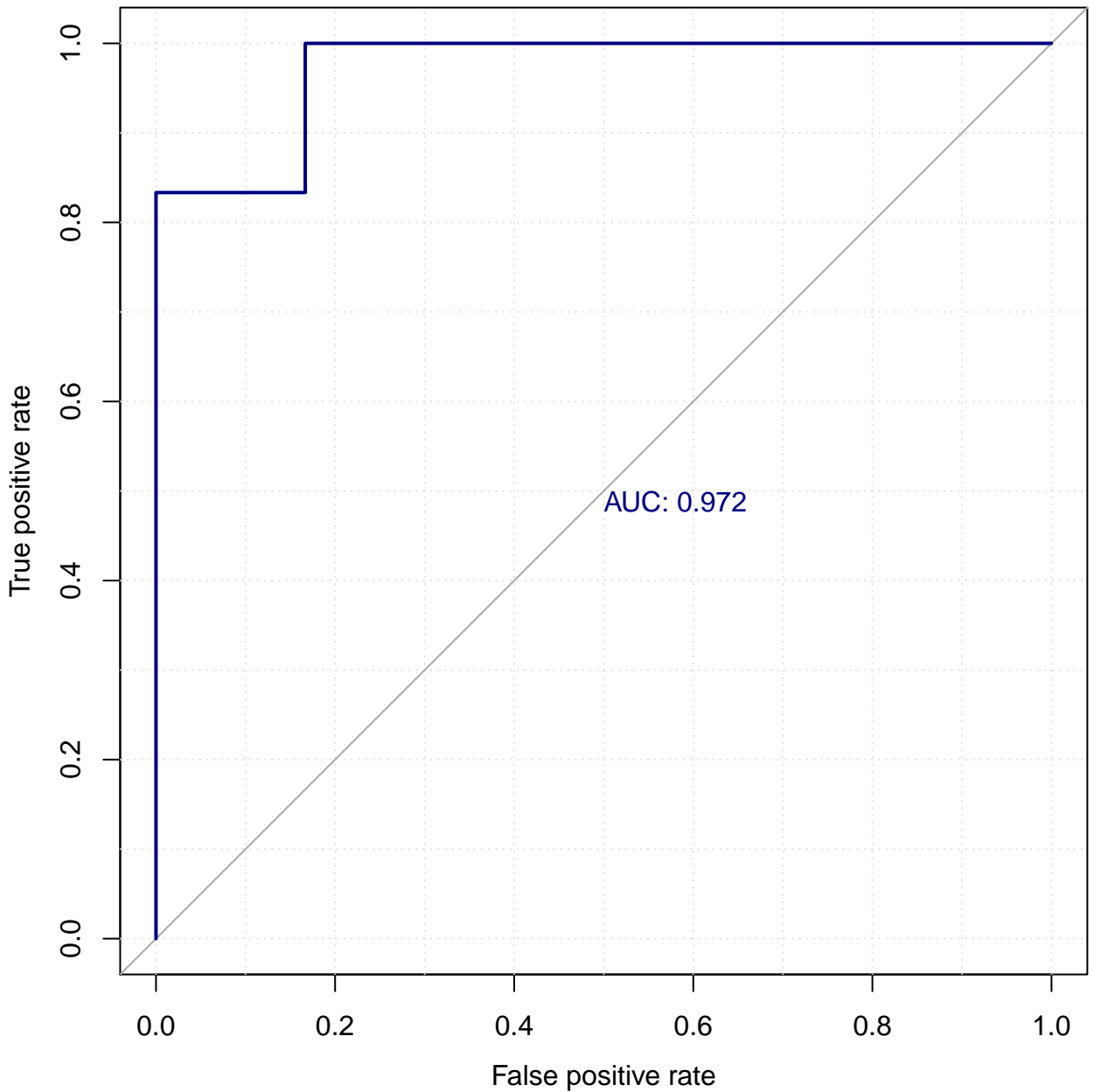

Supplement: Supplementary file 1 — Supplementary Information 1. [file 41598_2022_24687_MOESM1_ESM.zip › raw data/Metabolomics raw data/4.MetDiffAnalysis/EG.vs.CG/ROC_neg/Com_1608_neg_ROC.pdf]

# EG.vs.CG

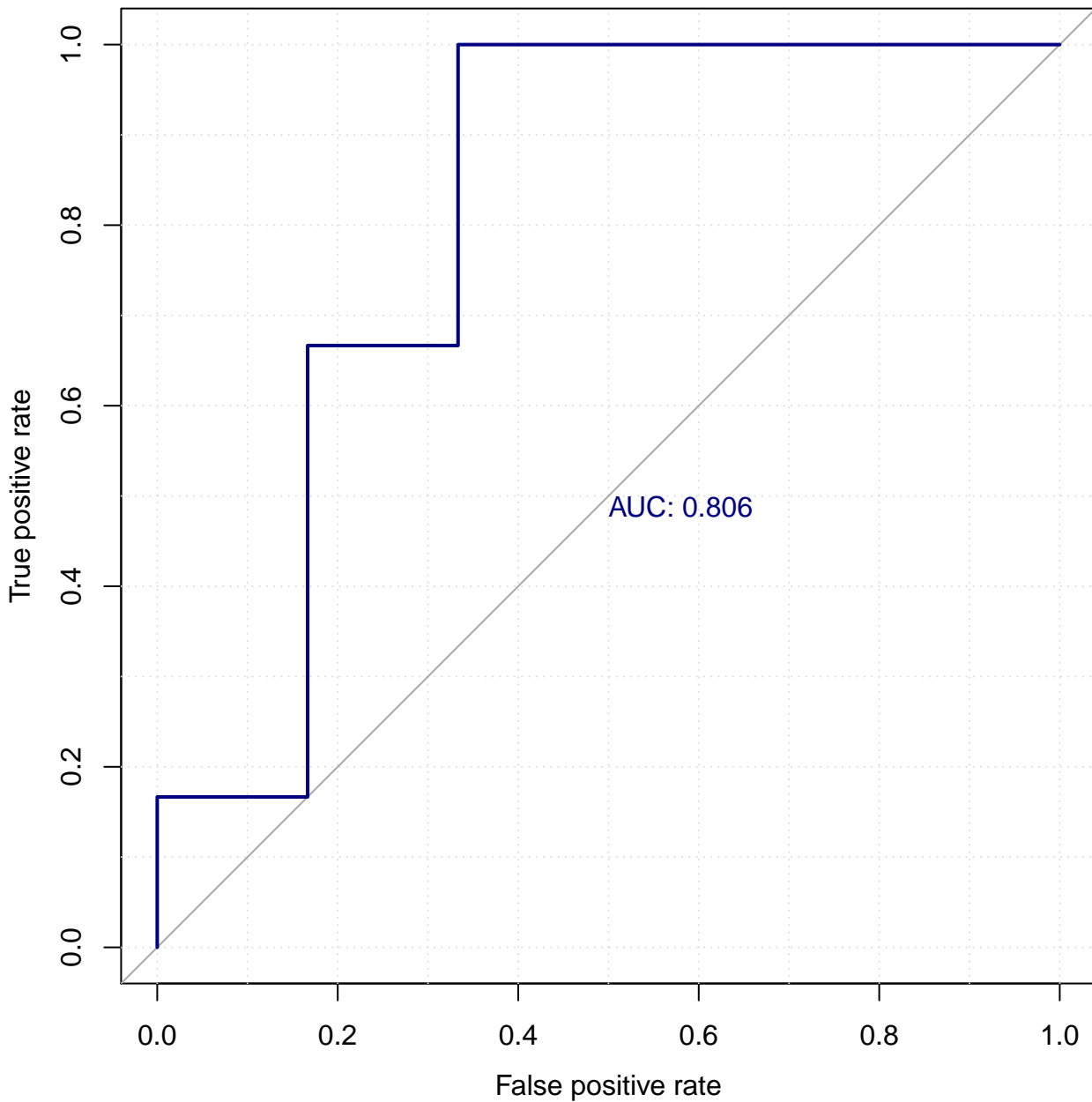

Supplement: Supplementary file 1 — Supplementary Information 1. [file 41598_2022_24687_MOESM1_ESM.zip › raw data/Metabolomics raw data/4.MetDiffAnalysis/EG.vs.CG/ROC_neg/Com_16443_neg_ROC.pdf]

# EG.vs.CG

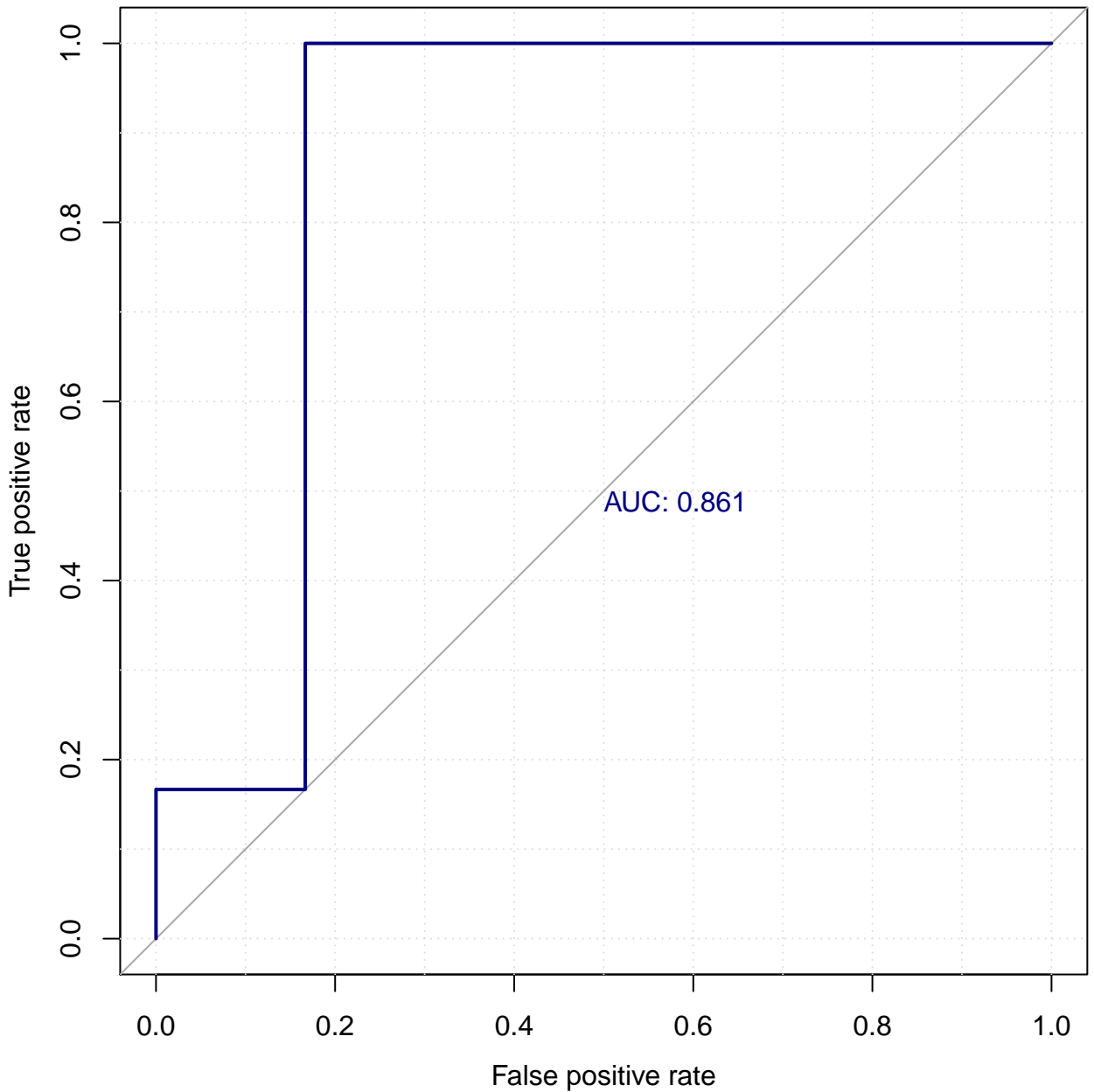

Supplement: Supplementary file 1 — Supplementary Information 1. [file 41598_2022_24687_MOESM1_ESM.zip › raw data/Metabolomics raw data/4.MetDiffAnalysis/EG.vs.CG/ROC_neg/Com_16570_neg_ROC.pdf]

# EG.vs.CG

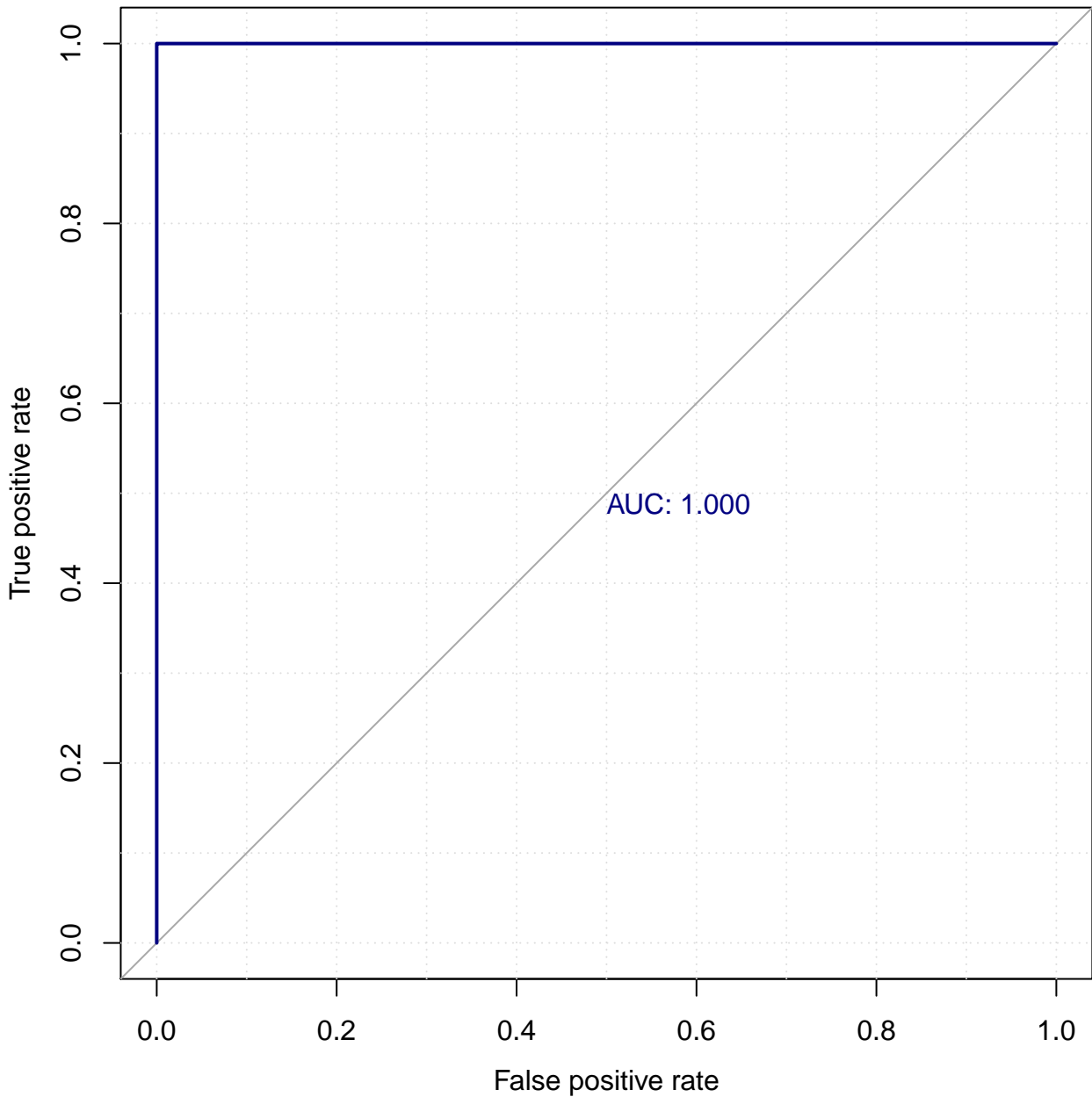

Supplement: Supplementary file 1 — Supplementary Information 1. [file 41598_2022_24687_MOESM1_ESM.zip › raw data/Metabolomics raw data/4.MetDiffAnalysis/EG.vs.CG/ROC_neg/Com_16591_neg_ROC.pdf]

# EG.vs.CG

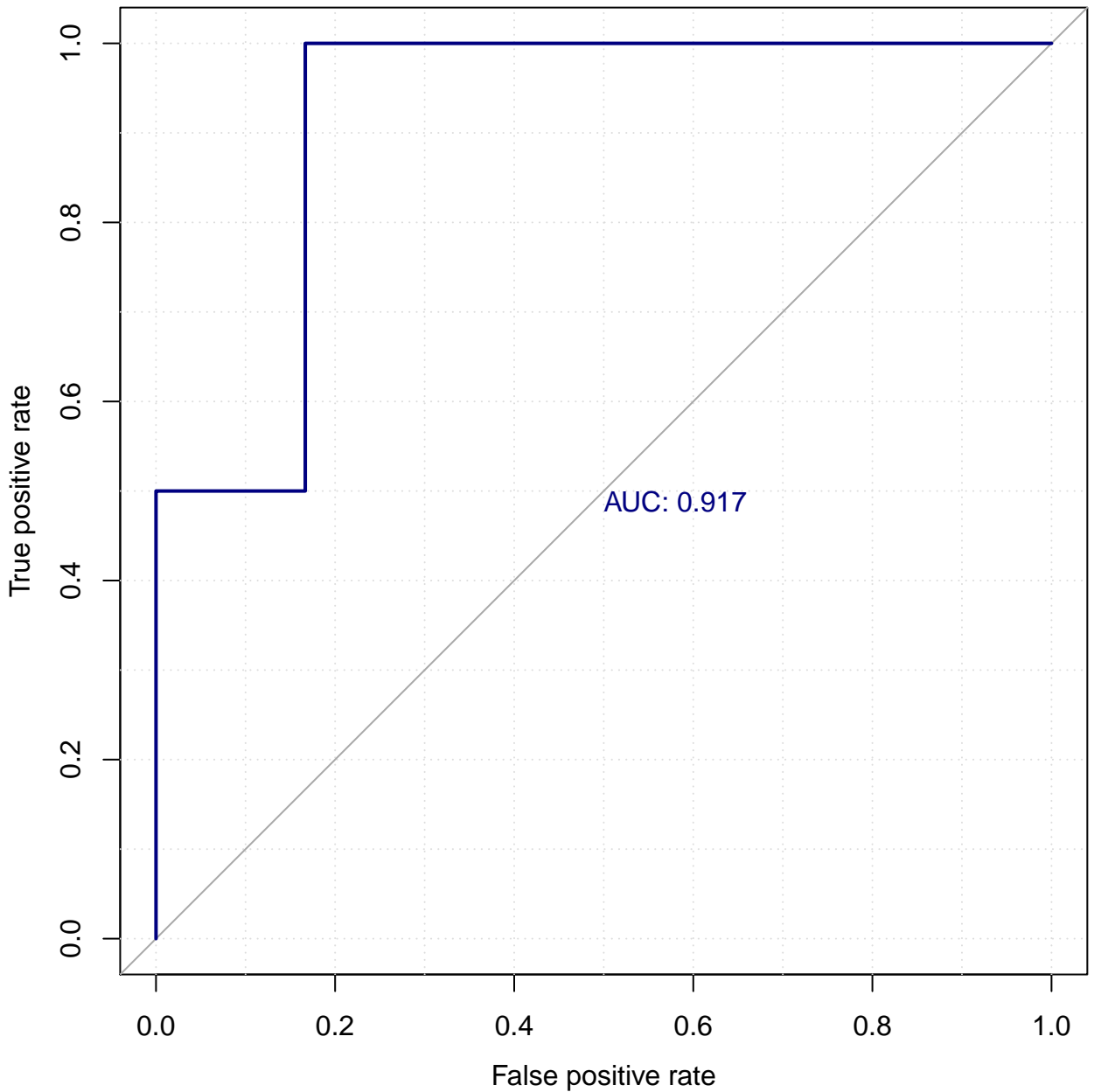

Supplement: Supplementary file 1 — Supplementary Information 1. [file 41598_2022_24687_MOESM1_ESM.zip › raw data/Metabolomics raw data/4.MetDiffAnalysis/EG.vs.CG/ROC_neg/Com_16734_neg_ROC.pdf]

# EG.vs.CG

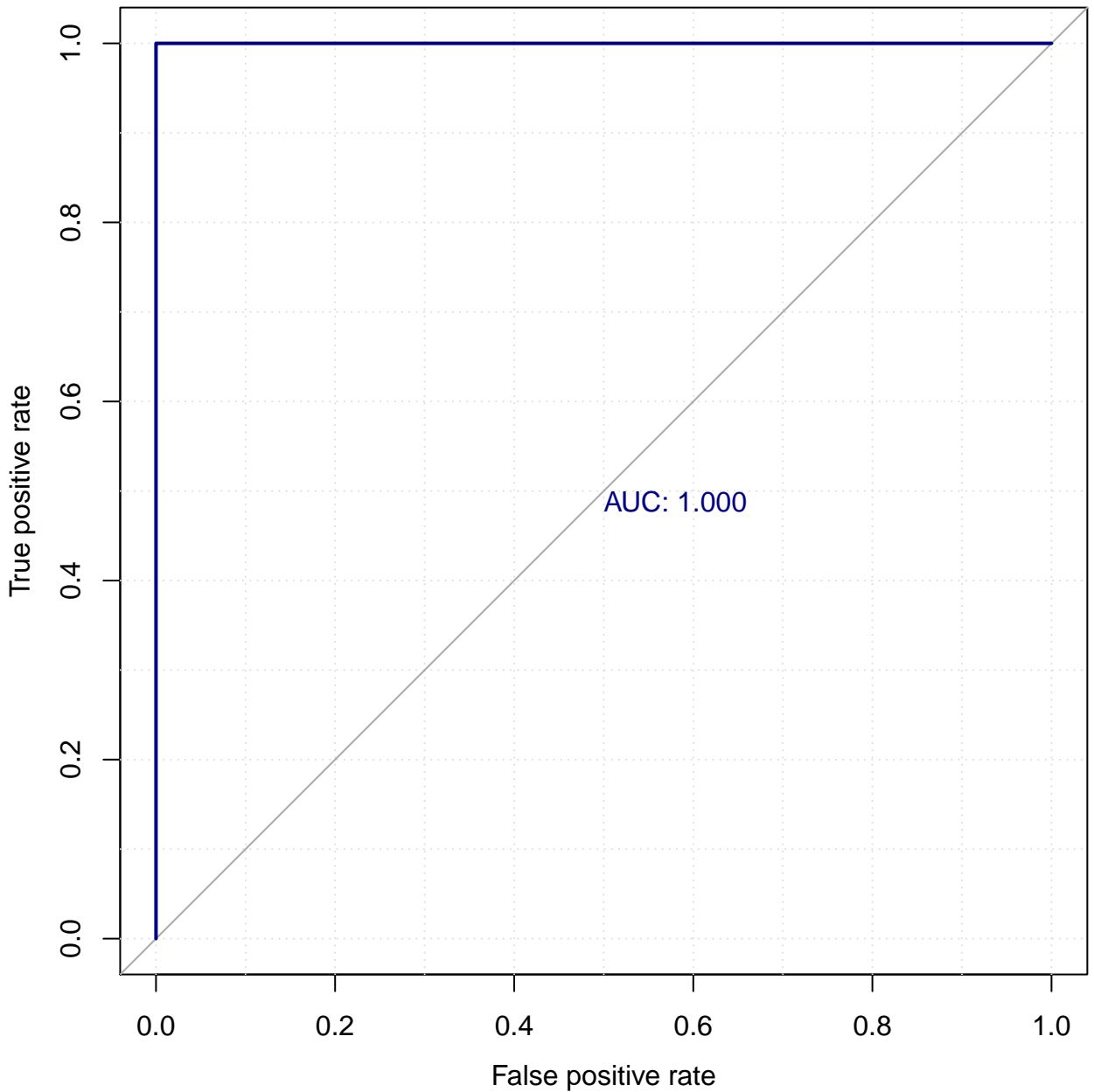

Supplement: Supplementary file 1 — Supplementary Information 1. [file 41598_2022_24687_MOESM1_ESM.zip › raw data/Metabolomics raw data/4.MetDiffAnalysis/EG.vs.CG/ROC_neg/Com_16820_neg_ROC.pdf]

# EG.vs.CG

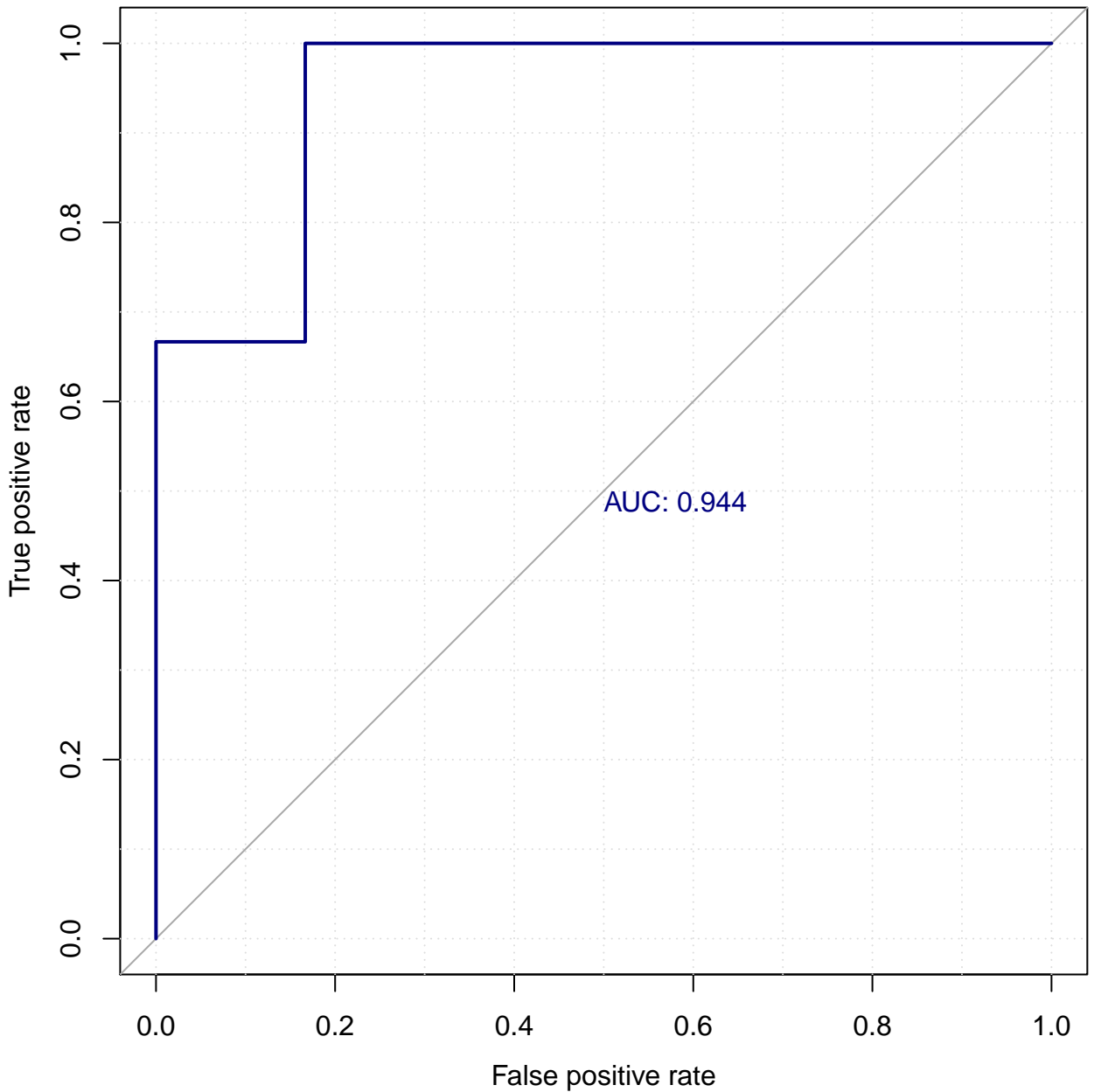

Supplement: Supplementary file 1 — Supplementary Information 1. [file 41598_2022_24687_MOESM1_ESM.zip › raw data/Metabolomics raw data/4.MetDiffAnalysis/EG.vs.CG/ROC_neg/Com_1687_neg_ROC.pdf]

# EG.vs.CG

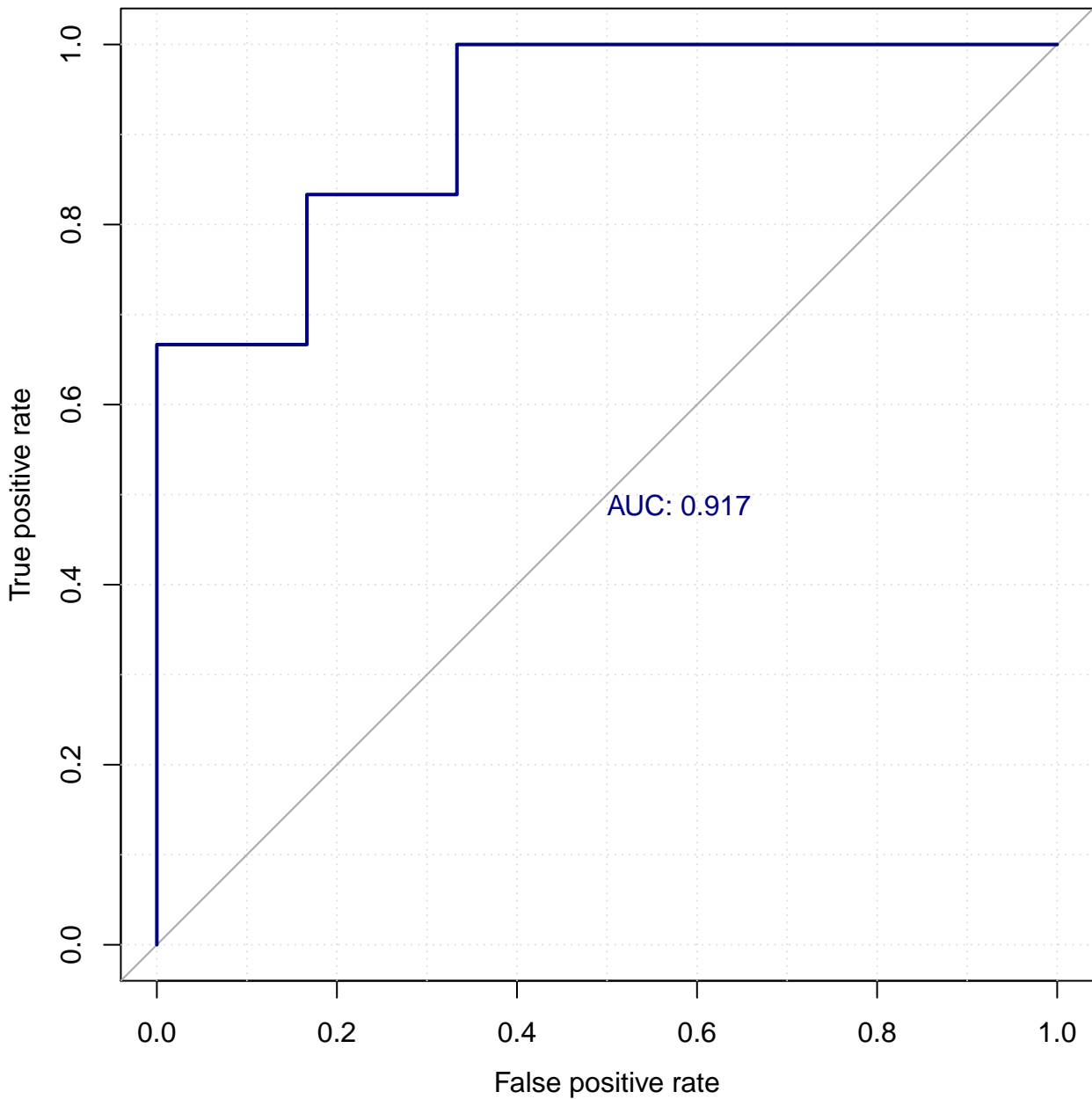

Supplement: Supplementary file 1 — Supplementary Information 1. [file 41598_2022_24687_MOESM1_ESM.zip › raw data/Metabolomics raw data/4.MetDiffAnalysis/EG.vs.CG/ROC_neg/Com_1698_neg_ROC.pdf]

# EG.vs.CG

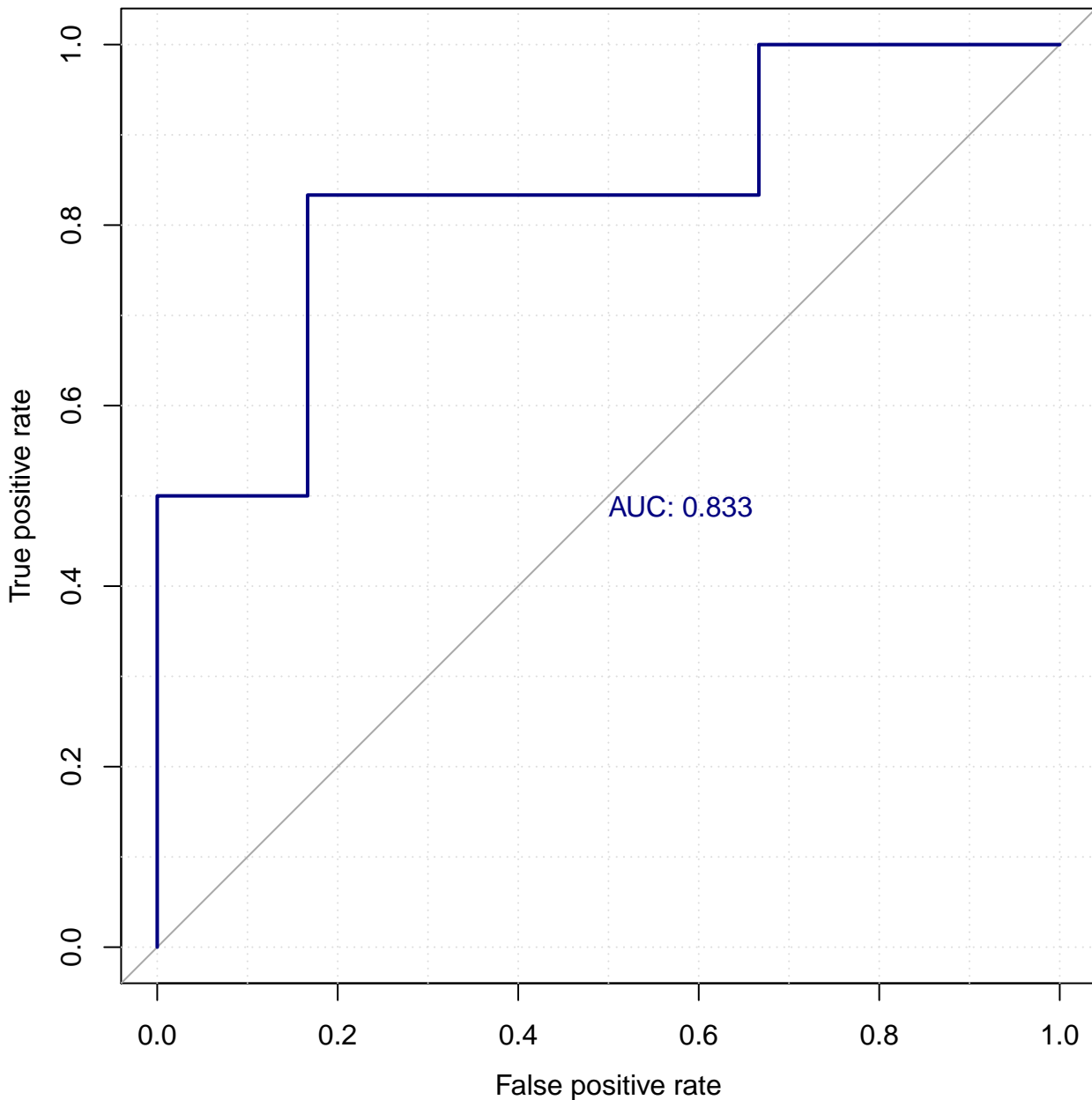

Supplement: Supplementary file 1 — Supplementary Information 1. [file 41598_2022_24687_MOESM1_ESM.zip › raw data/Metabolomics raw data/4.MetDiffAnalysis/EG.vs.CG/ROC_neg/Com_1728_neg_ROC.pdf]

# EG.vs.CG

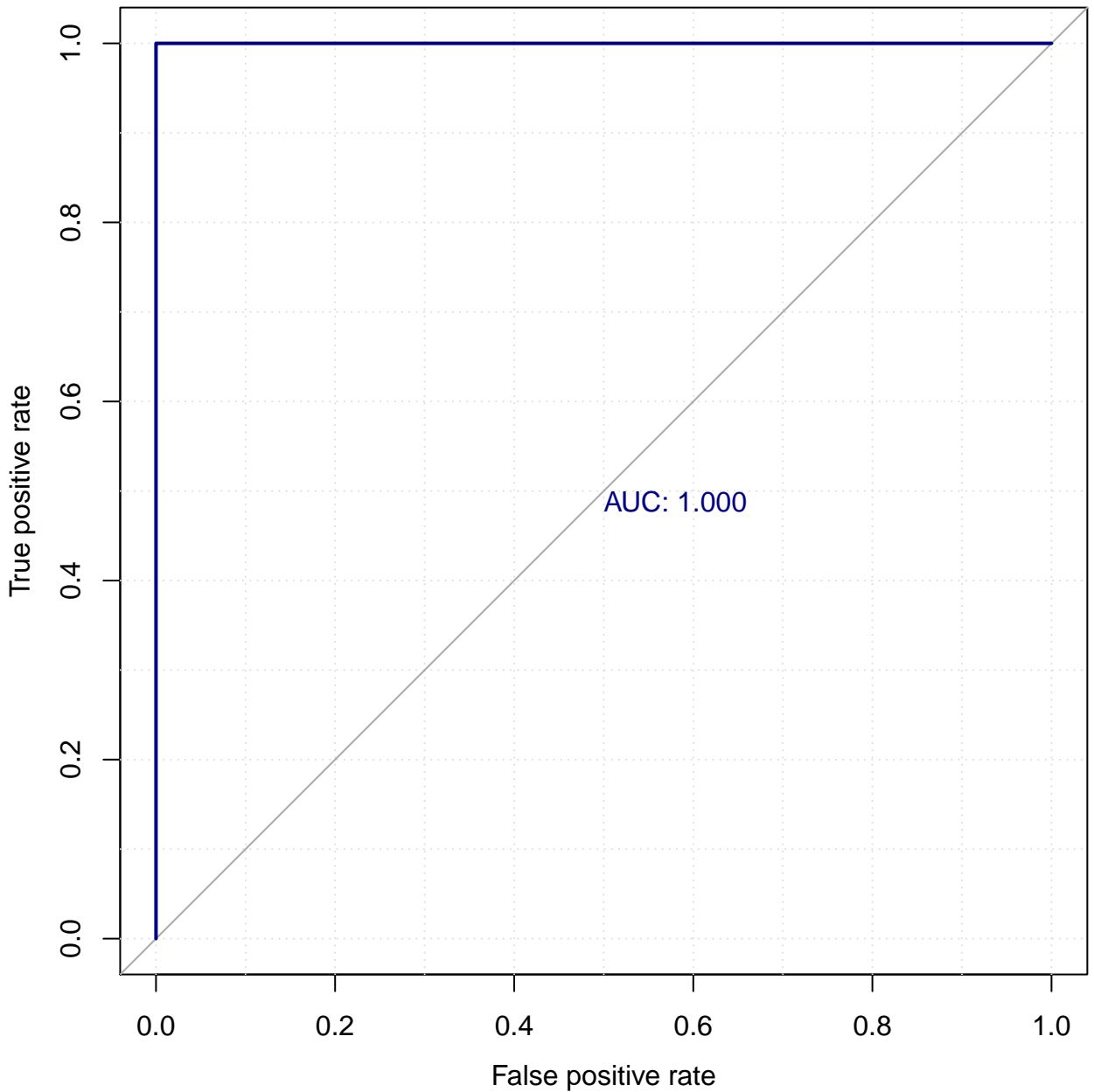

Supplement: Supplementary file 1 — Supplementary Information 1. [file 41598_2022_24687_MOESM1_ESM.zip › raw data/Metabolomics raw data/4.MetDiffAnalysis/EG.vs.CG/ROC_neg/Com_1781_neg_ROC.pdf]

# EG.vs.CG

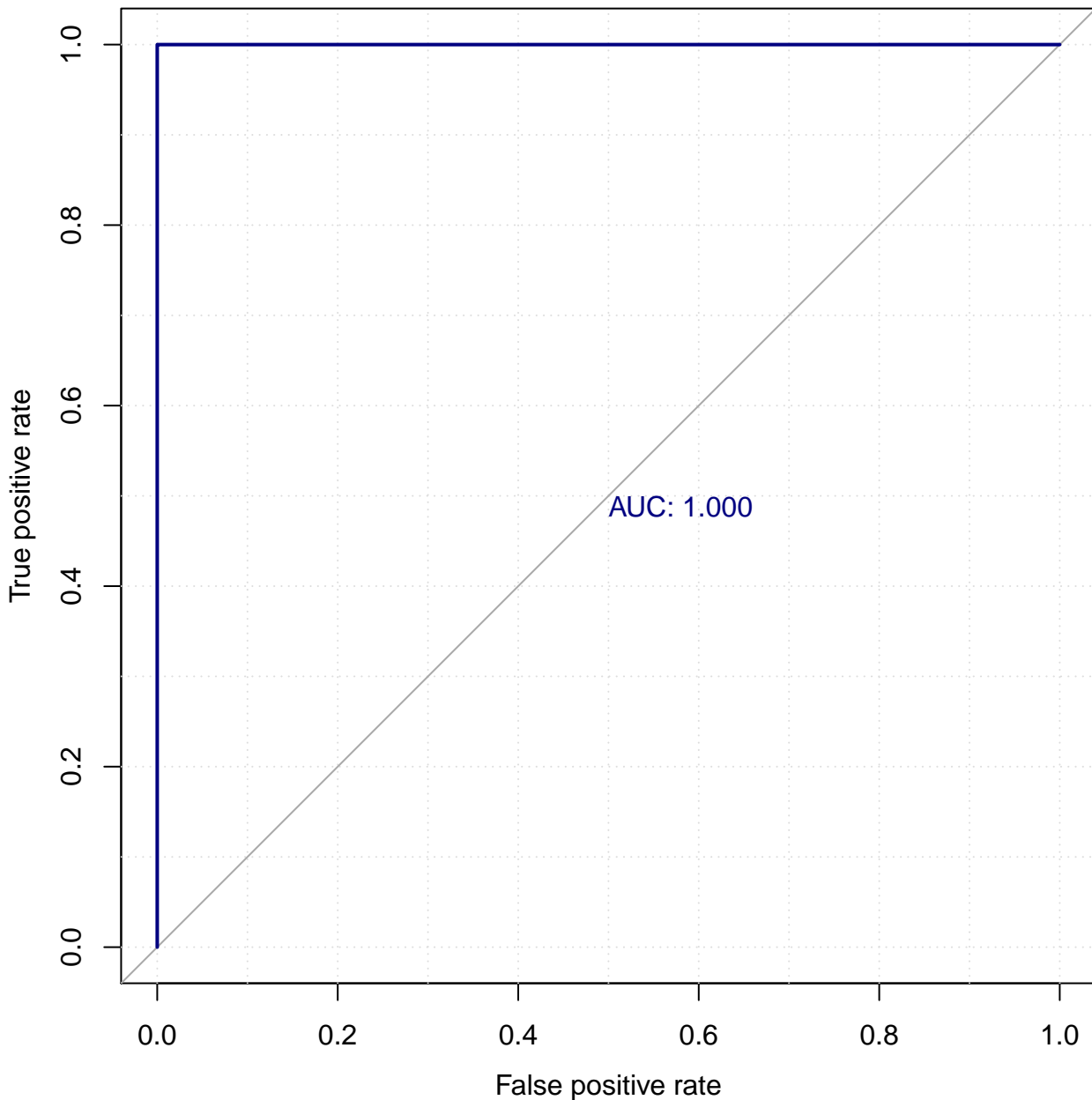

Supplement: Supplementary file 1 — Supplementary Information 1. [file 41598_2022_24687_MOESM1_ESM.zip › raw data/Metabolomics raw data/4.MetDiffAnalysis/EG.vs.CG/ROC_neg/Com_1828_neg_ROC.pdf]

# EG.vs.CG

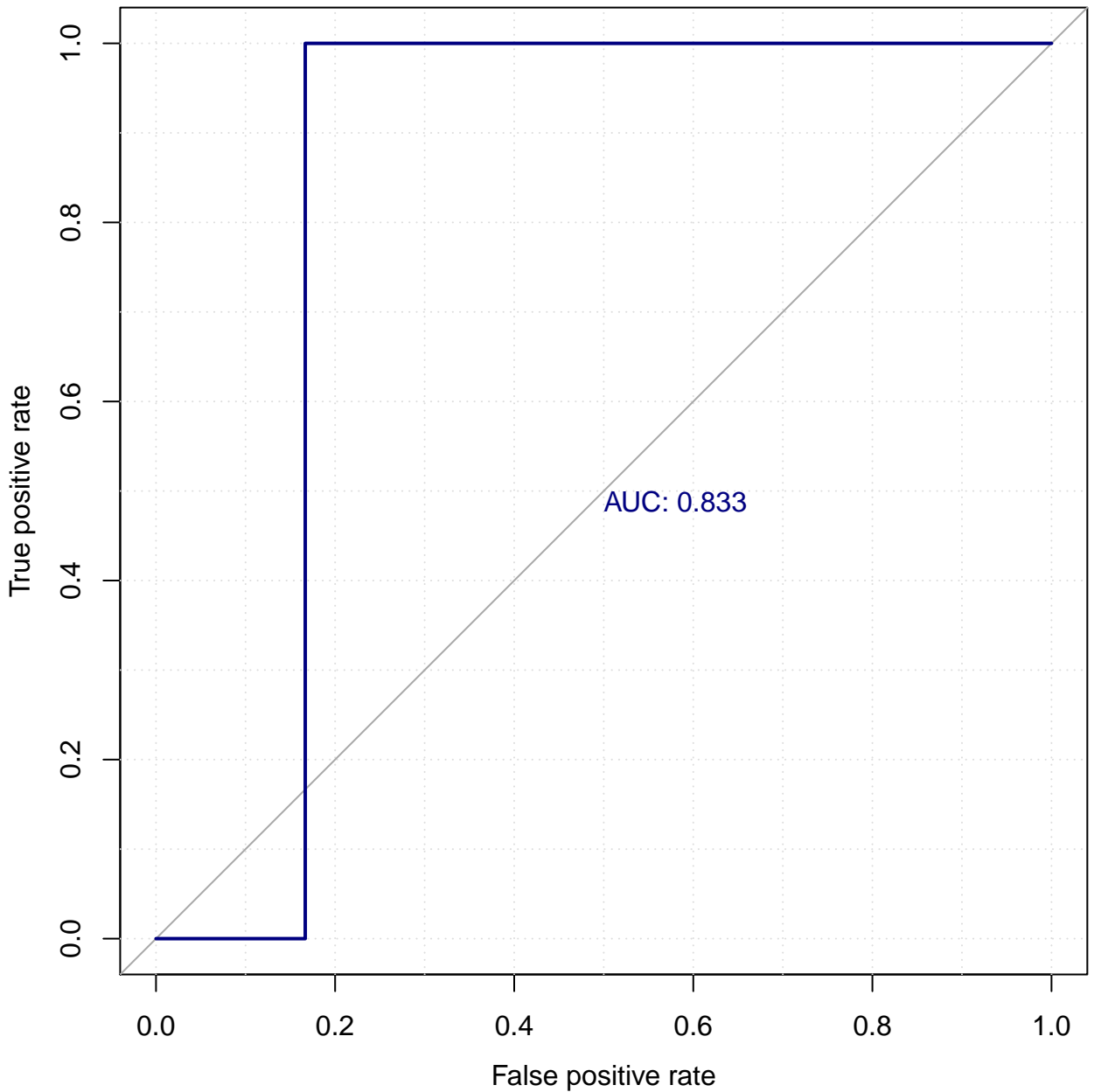

Supplement: Supplementary file 1 — Supplementary Information 1. [file 41598_2022_24687_MOESM1_ESM.zip › raw data/Metabolomics raw data/4.MetDiffAnalysis/EG.vs.CG/ROC_neg/Com_1897_neg_ROC.pdf]

# EG.vs.CG

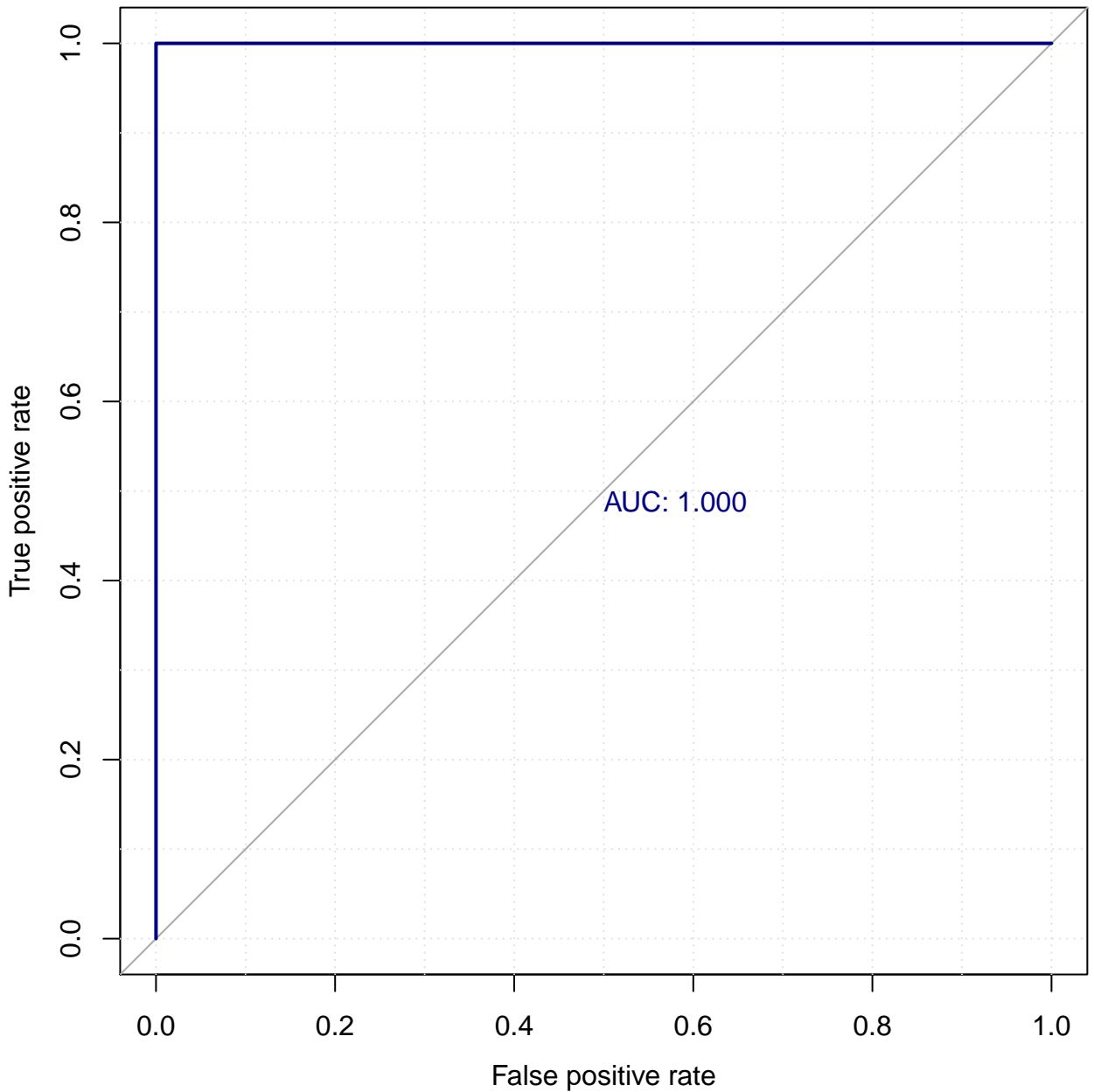

Supplement: Supplementary file 1 — Supplementary Information 1. [file 41598_2022_24687_MOESM1_ESM.zip › raw data/Metabolomics raw data/4.MetDiffAnalysis/EG.vs.CG/ROC_neg/Com_19_neg_ROC.pdf]

# EG.vs.CG

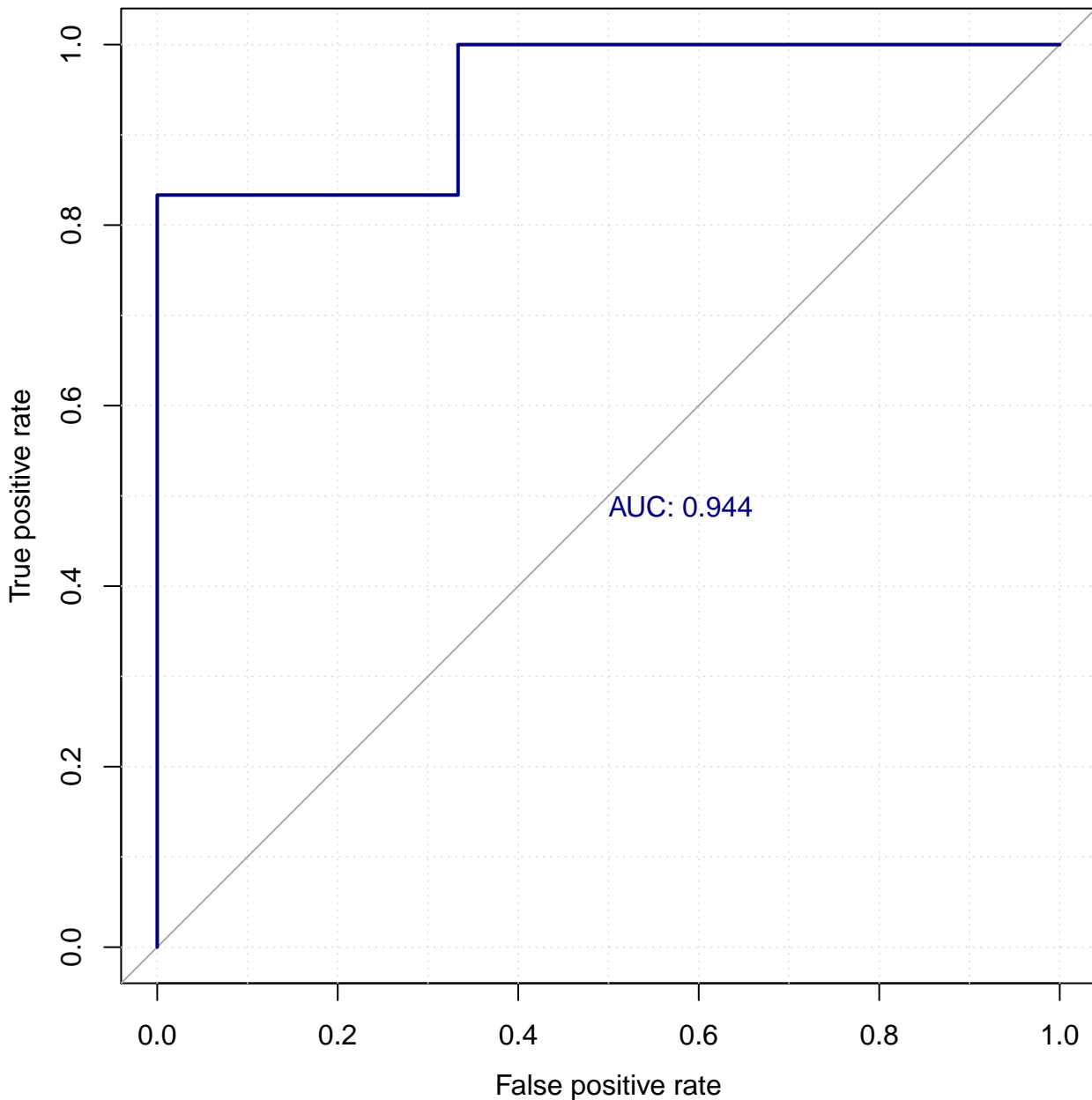

Supplement: Supplementary file 1 — Supplementary Information 1. [file 41598_2022_24687_MOESM1_ESM.zip › raw data/Metabolomics raw data/4.MetDiffAnalysis/EG.vs.CG/ROC_neg/Com_2124_neg_ROC.pdf]

# EG.vs.CG

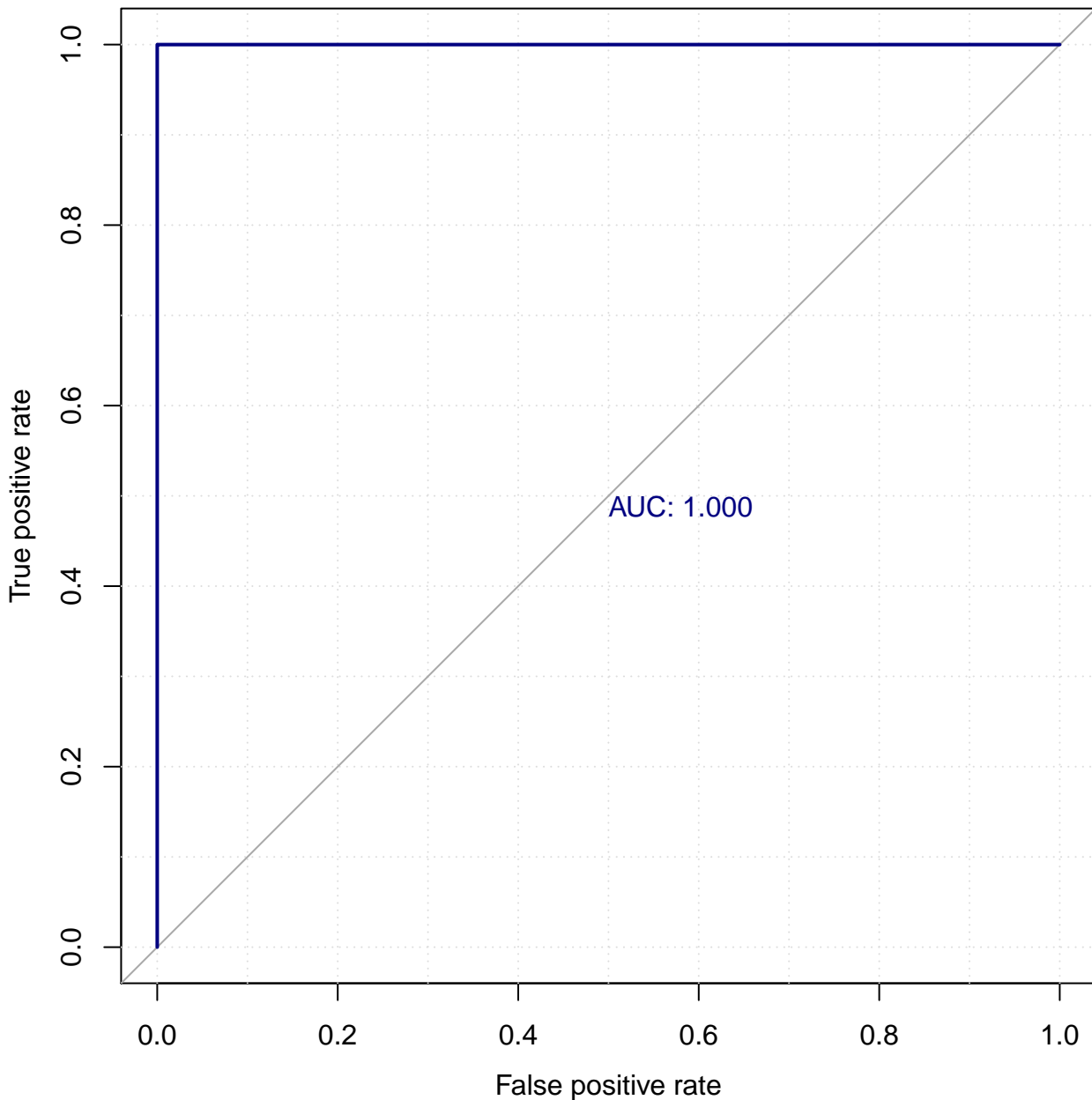

Supplement: Supplementary file 1 — Supplementary Information 1. [file 41598_2022_24687_MOESM1_ESM.zip › raw data/Metabolomics raw data/4.MetDiffAnalysis/EG.vs.CG/ROC_neg/Com_267_neg_ROC.pdf]

# EG.vs.CG

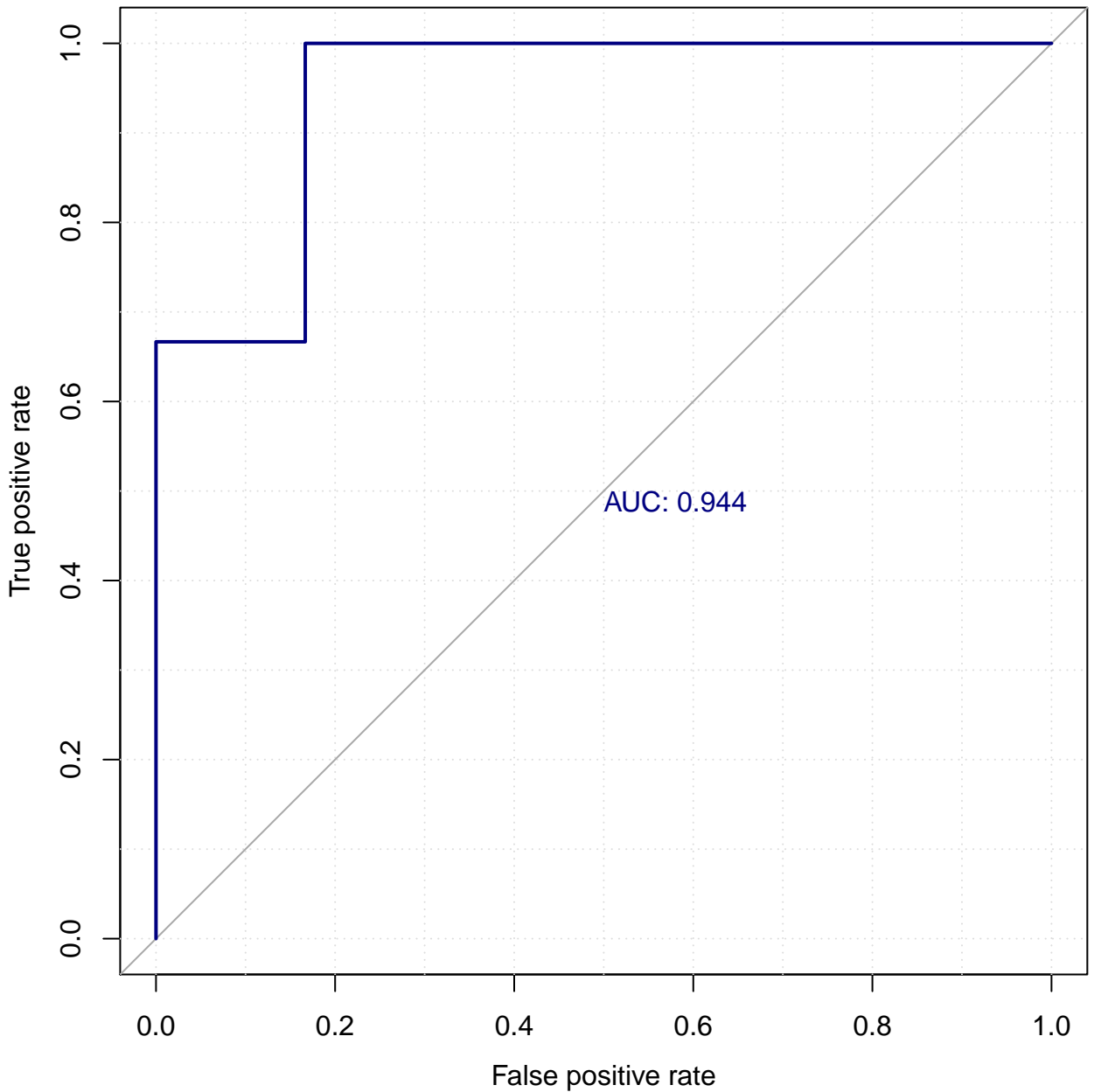

Supplement: Supplementary file 1 — Supplementary Information 1. [file 41598_2022_24687_MOESM1_ESM.zip › raw data/Metabolomics raw data/4.MetDiffAnalysis/EG.vs.CG/ROC_neg/Com_2693_neg_ROC.pdf]

# EG.vs.CG

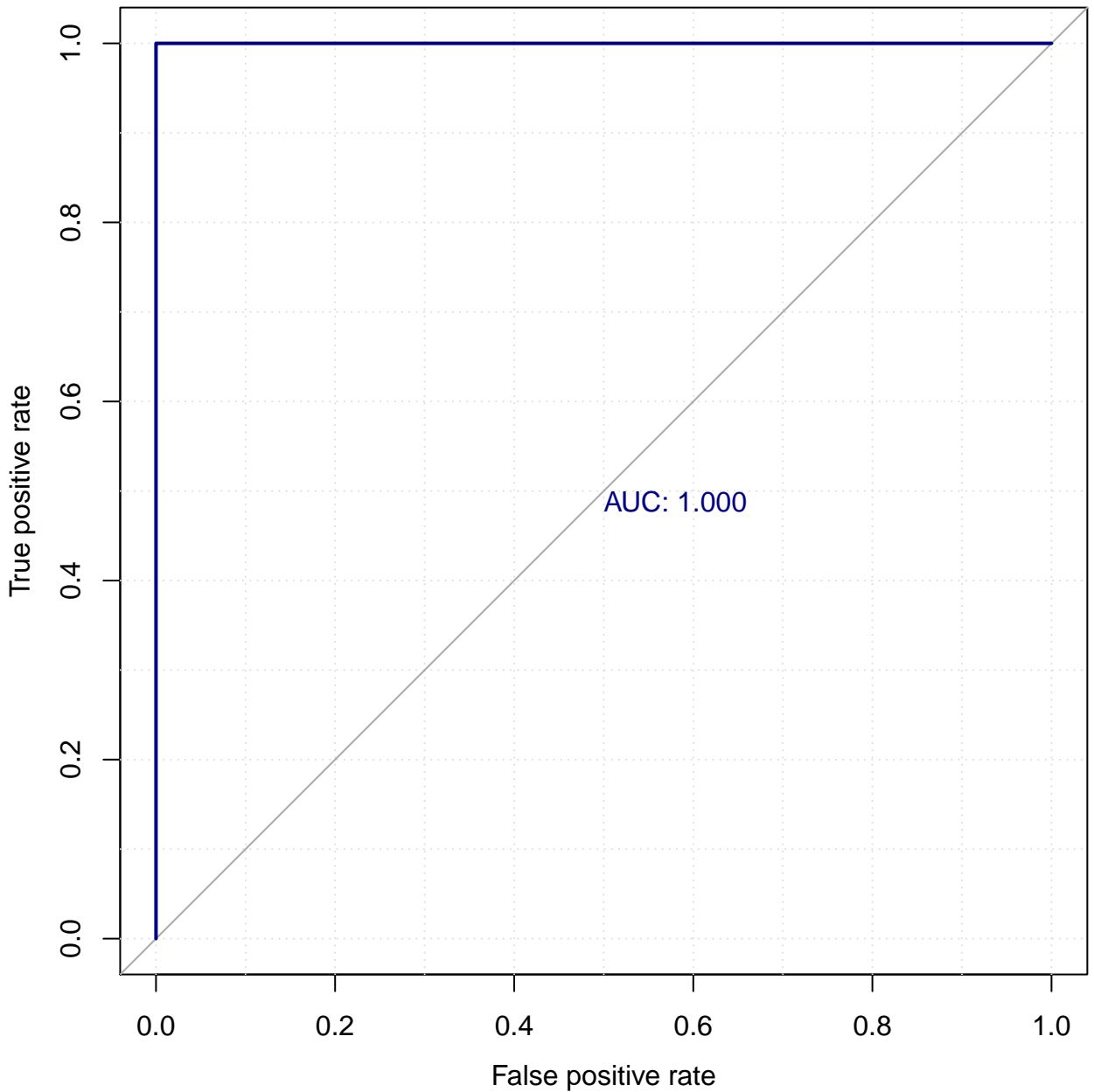

Supplement: Supplementary file 1 — Supplementary Information 1. [file 41598_2022_24687_MOESM1_ESM.zip › raw data/Metabolomics raw data/4.MetDiffAnalysis/EG.vs.CG/ROC_neg/Com_2705_neg_ROC.pdf]

# EG.vs.CG

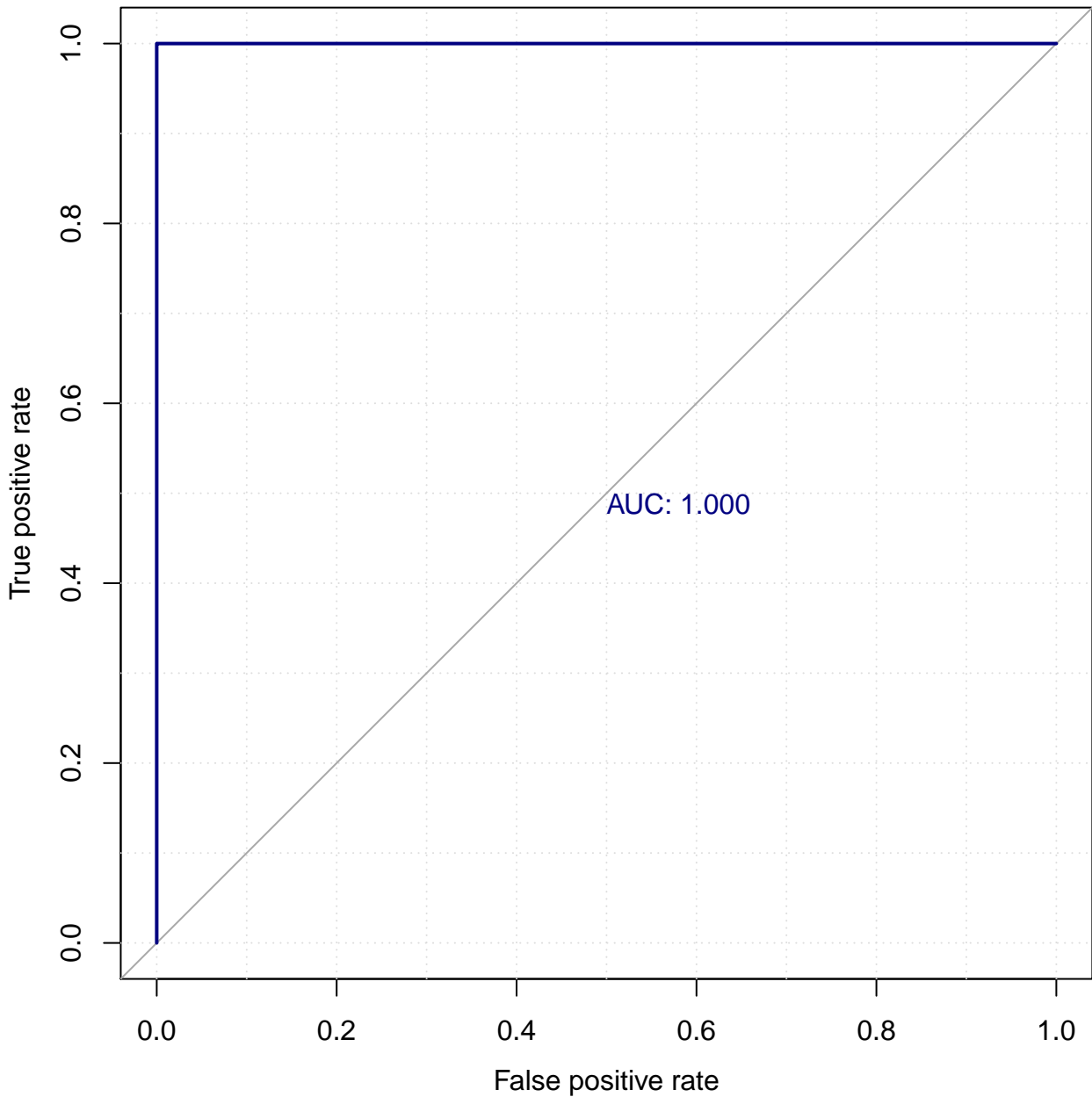

Supplement: Supplementary file 1 — Supplementary Information 1. [file 41598_2022_24687_MOESM1_ESM.zip › raw data/Metabolomics raw data/4.MetDiffAnalysis/EG.vs.CG/ROC_neg/Com_271_neg_ROC.pdf]

# EG.vs.CG

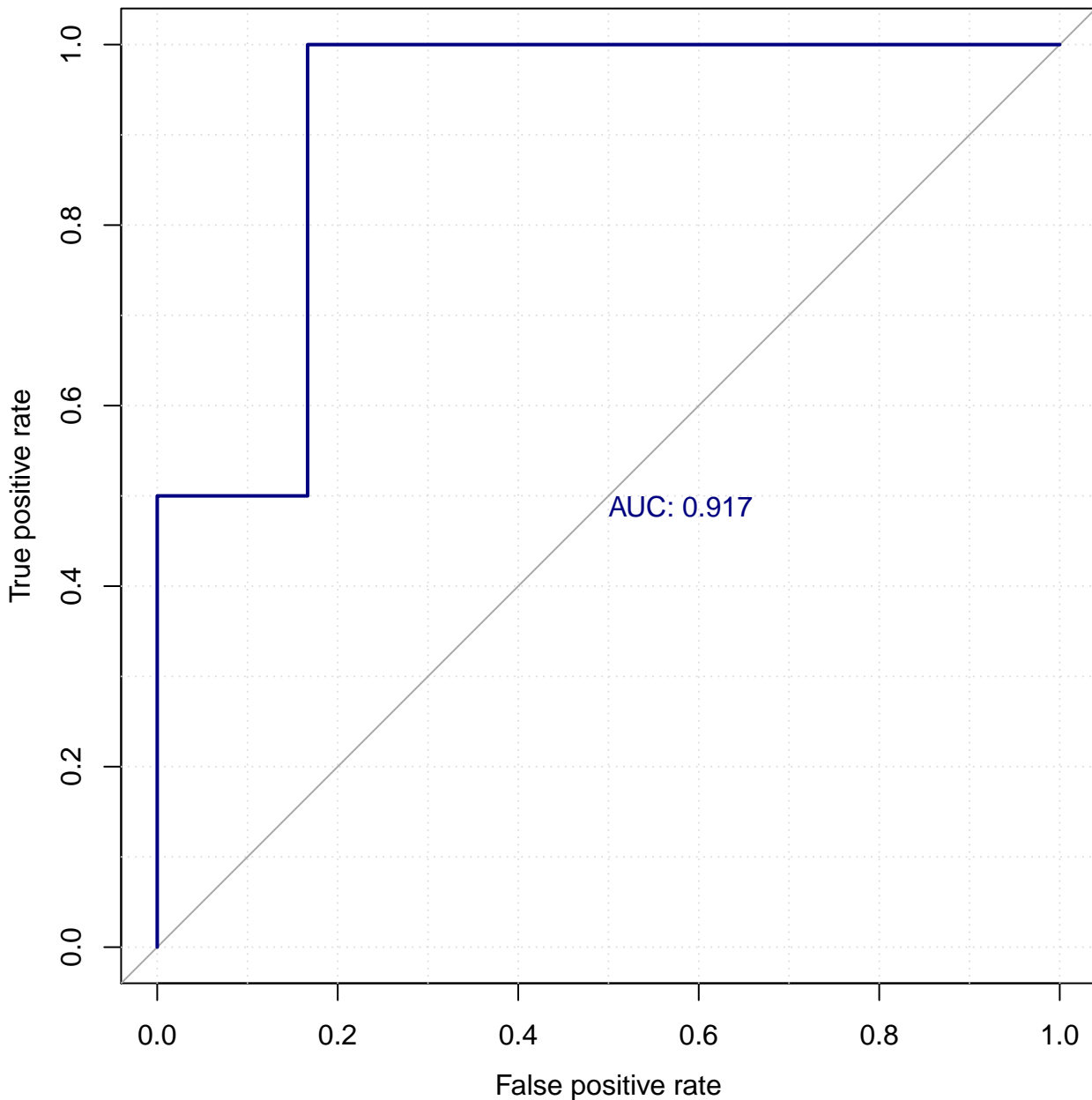

Supplement: Supplementary file 1 — Supplementary Information 1. [file 41598_2022_24687_MOESM1_ESM.zip › raw data/Metabolomics raw data/4.MetDiffAnalysis/EG.vs.CG/ROC_neg/Com_2974_neg_ROC.pdf]

# EG.vs.CG

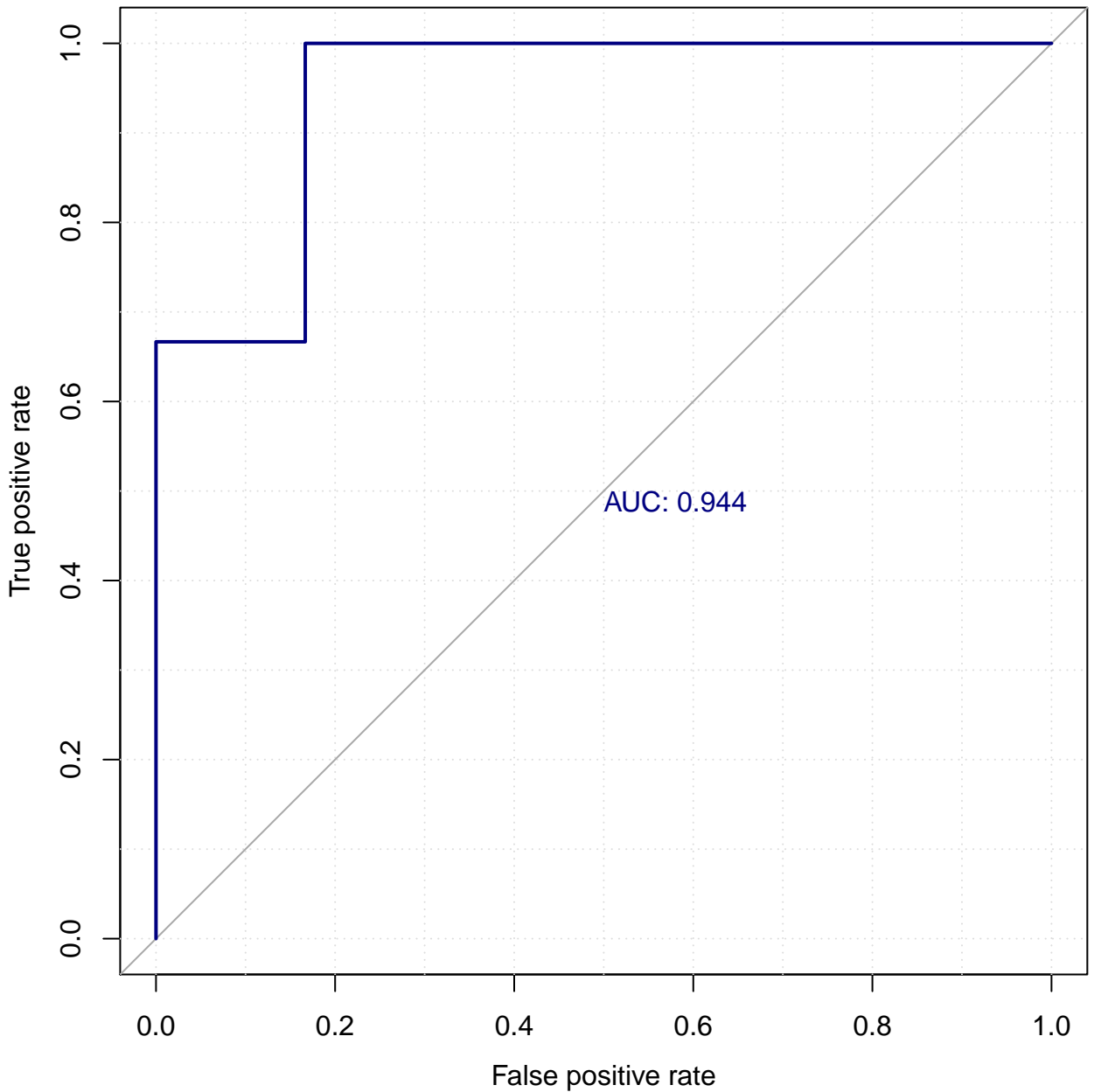

Supplement: Supplementary file 1 — Supplementary Information 1. [file 41598_2022_24687_MOESM1_ESM.zip › raw data/Metabolomics raw data/4.MetDiffAnalysis/EG.vs.CG/ROC_neg/Com_3046_neg_ROC.pdf]

# EG.vs.CG

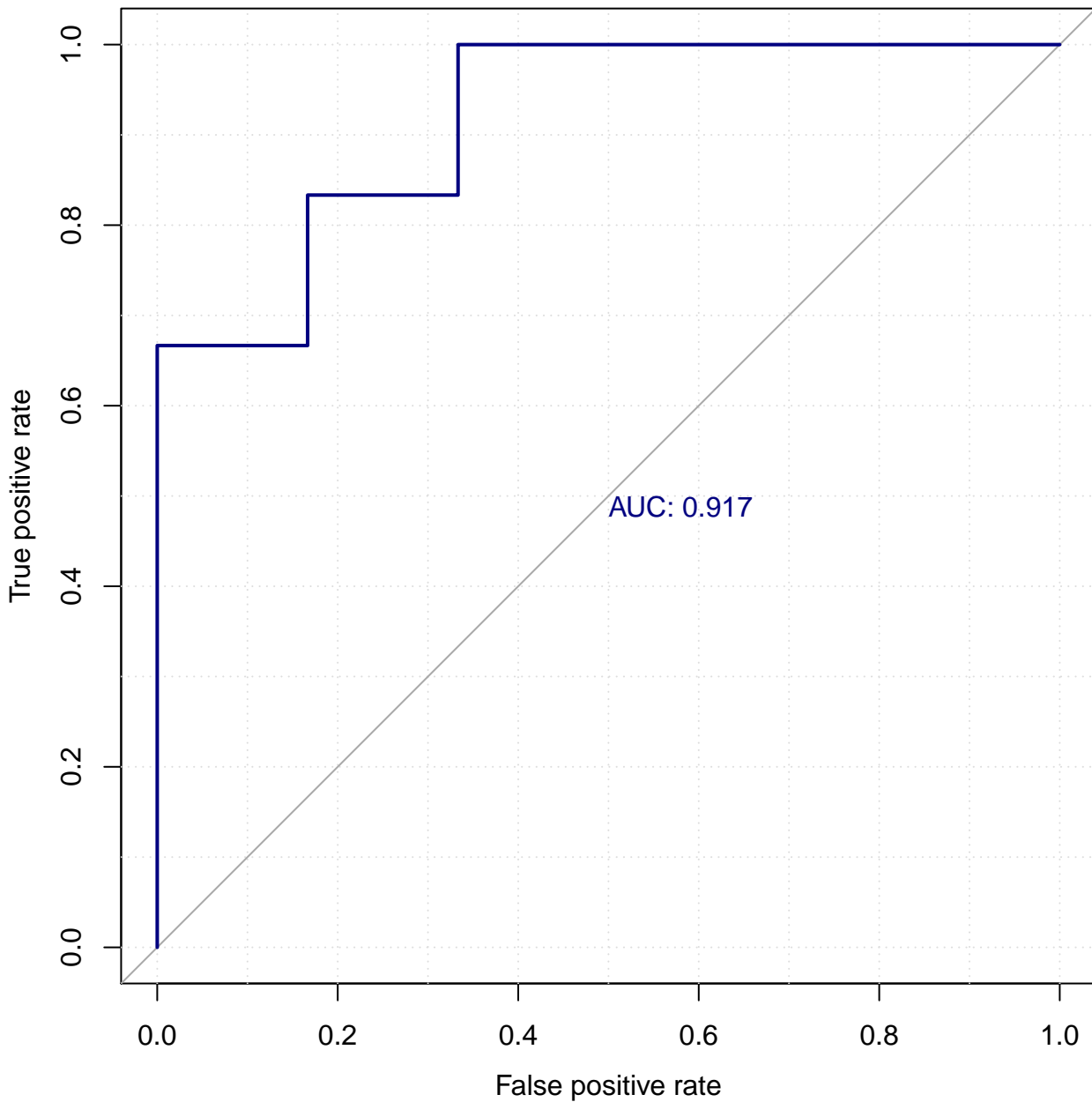

Supplement: Supplementary file 1 — Supplementary Information 1. [file 41598_2022_24687_MOESM1_ESM.zip › raw data/Metabolomics raw data/4.MetDiffAnalysis/EG.vs.CG/ROC_neg/Com_3147_neg_ROC.pdf]

# EG.vs.CG

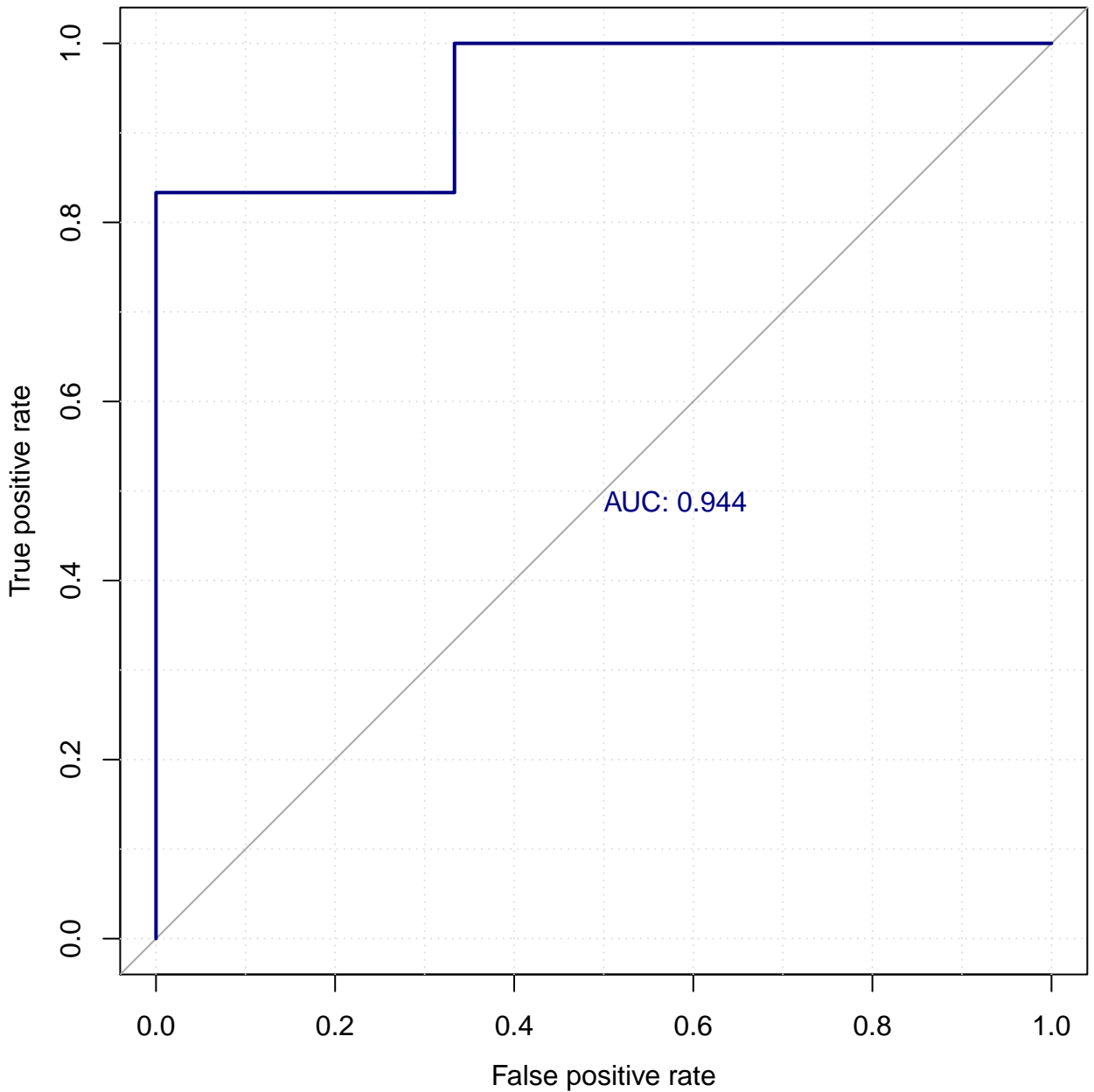

Supplement: Supplementary file 1 — Supplementary Information 1. [file 41598_2022_24687_MOESM1_ESM.zip › raw data/Metabolomics raw data/4.MetDiffAnalysis/EG.vs.CG/ROC_neg/Com_333_neg_ROC.pdf]

# EG.vs.CG

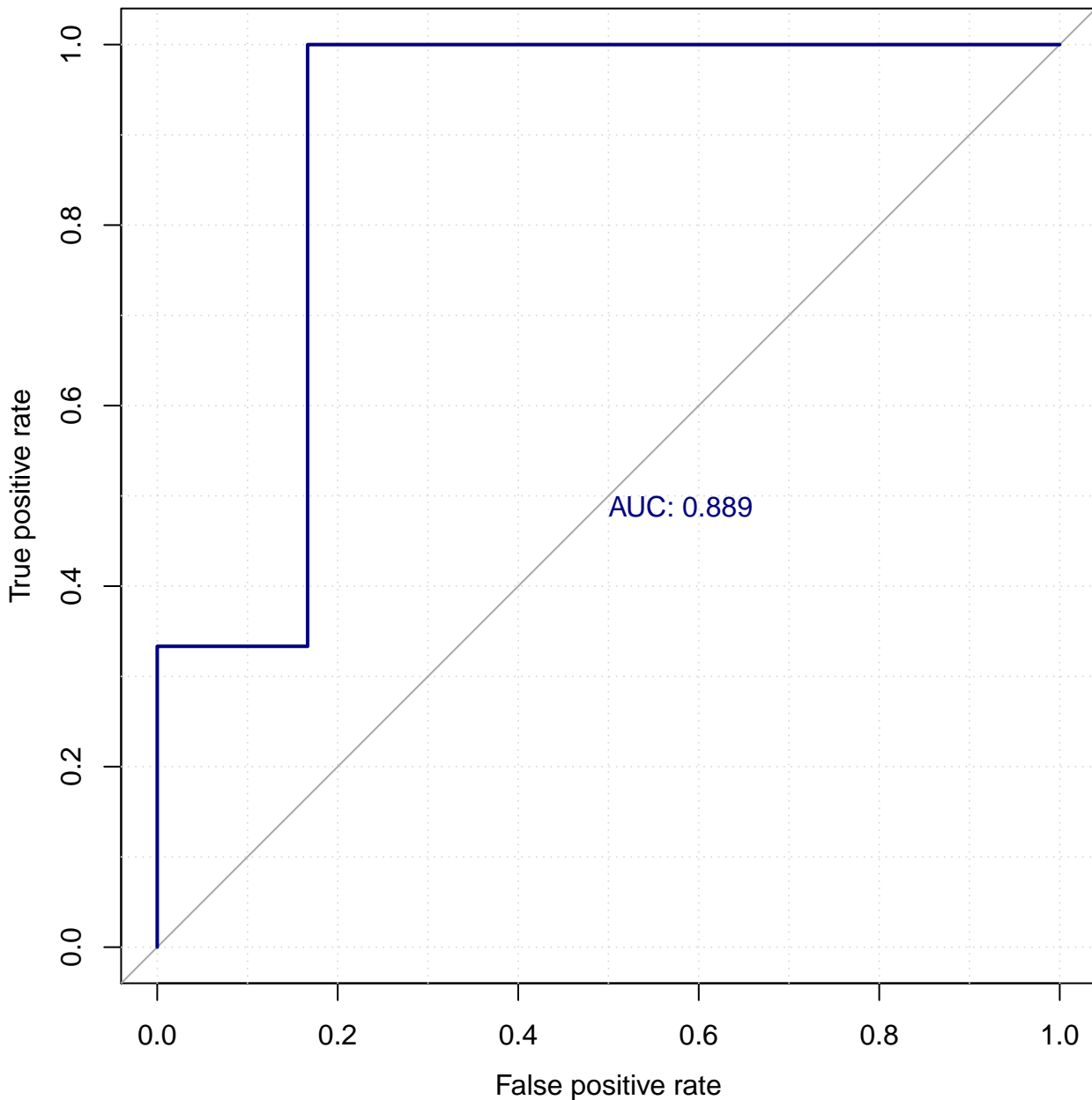

Supplement: Supplementary file 1 — Supplementary Information 1. [file 41598_2022_24687_MOESM1_ESM.zip › raw data/Metabolomics raw data/4.MetDiffAnalysis/EG.vs.CG/ROC_neg/Com_344_neg_ROC.pdf]

# EG.vs.CG

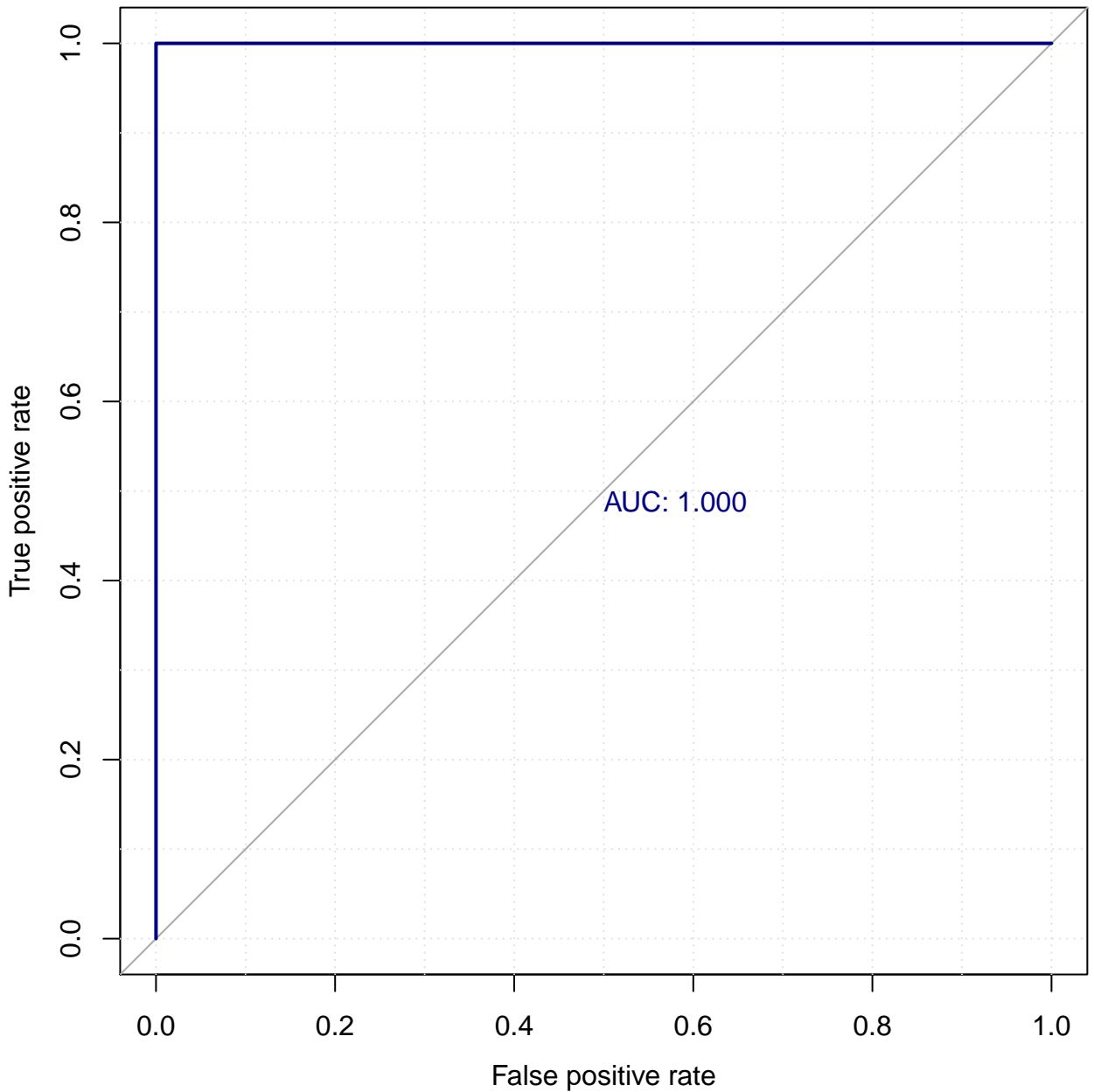

Supplement: Supplementary file 1 — Supplementary Information 1. [file 41598_2022_24687_MOESM1_ESM.zip › raw data/Metabolomics raw data/4.MetDiffAnalysis/EG.vs.CG/ROC_neg/Com_361_neg_ROC.pdf]
