# Supplementary figures and images for: Comparative multiomics analysis of cell physiological state after culture in a basket bioreactor (part 1 of 2)
Source: Sci Rep. 2022 Nov 23;12:20161. doi: 10.1038/s41598-022-24687-4 (PMC9686226; doi:10.1038/s41598-022-24687-4)

Pearson correlation between neg QC samples

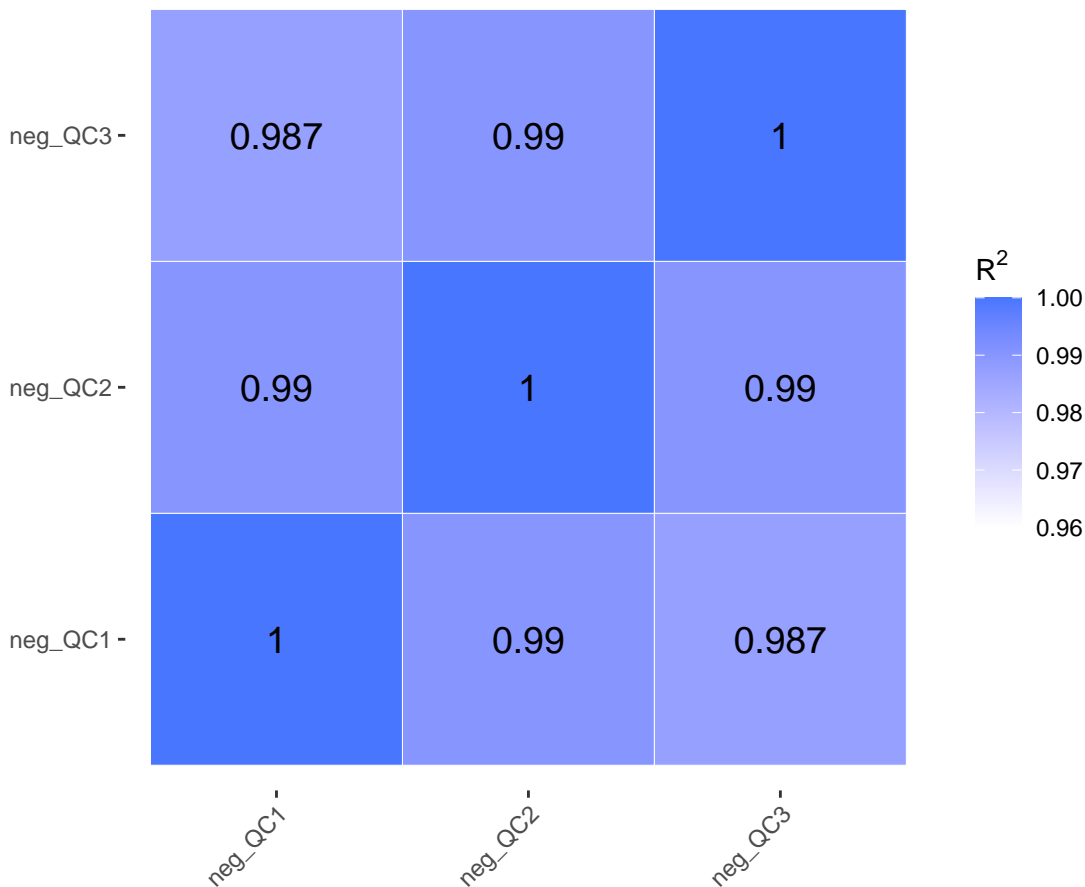

Supplement: Supplementary file 1 — Supplementary Information 1. [file 41598_2022_24687_MOESM1_ESM.zip › raw data/Metabolomics raw data/1.MetQuant-QC/Correlation/cor_pearson_neg.pdf]

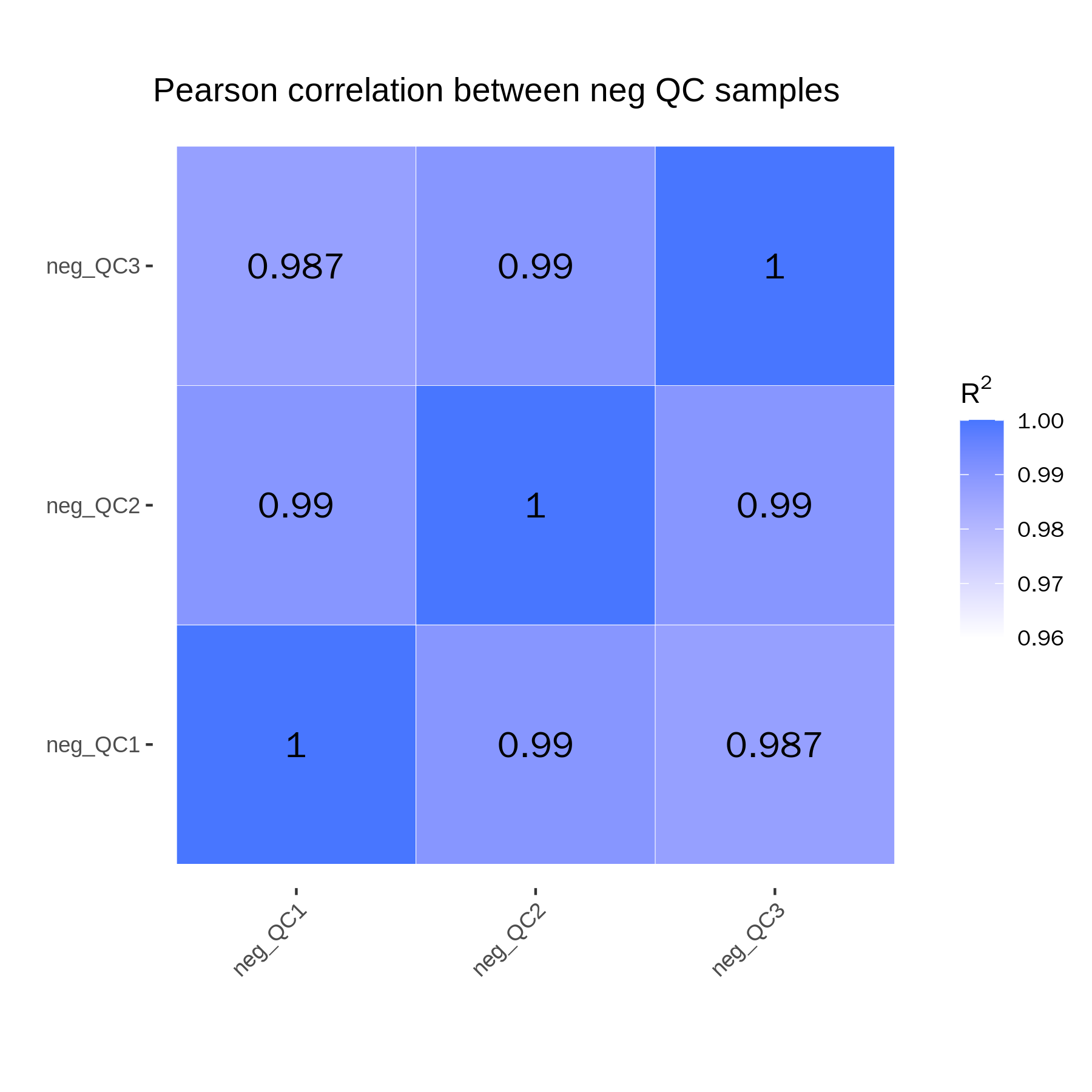

Supplement: Supplementary file 1 — Supplementary Information 1. [file 41598_2022_24687_MOESM1_ESM.zip › raw data/Metabolomics raw data/1.MetQuant-QC/Correlation/cor_pearson_neg.png]

Pearson correlation between pos QC samples

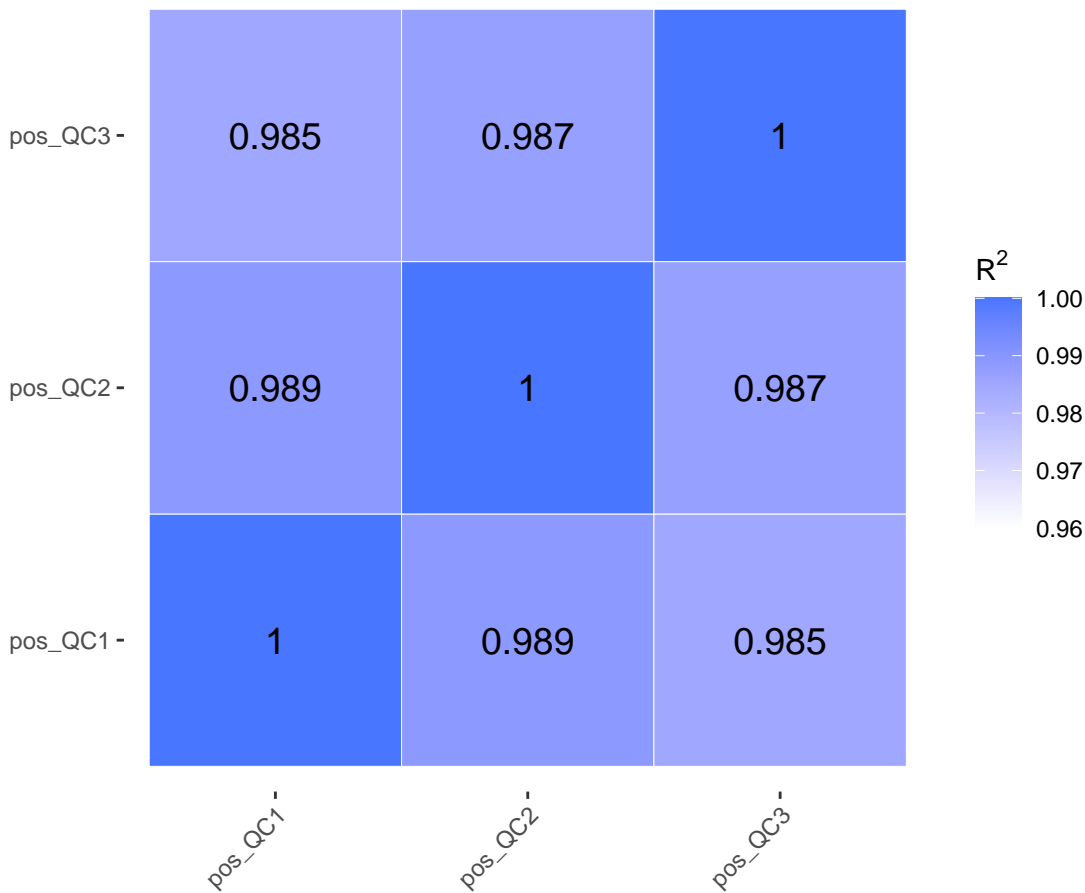

Supplement: Supplementary file 1 — Supplementary Information 1. [file 41598_2022_24687_MOESM1_ESM.zip › raw data/Metabolomics raw data/1.MetQuant-QC/Correlation/cor_pearson_pos.pdf]

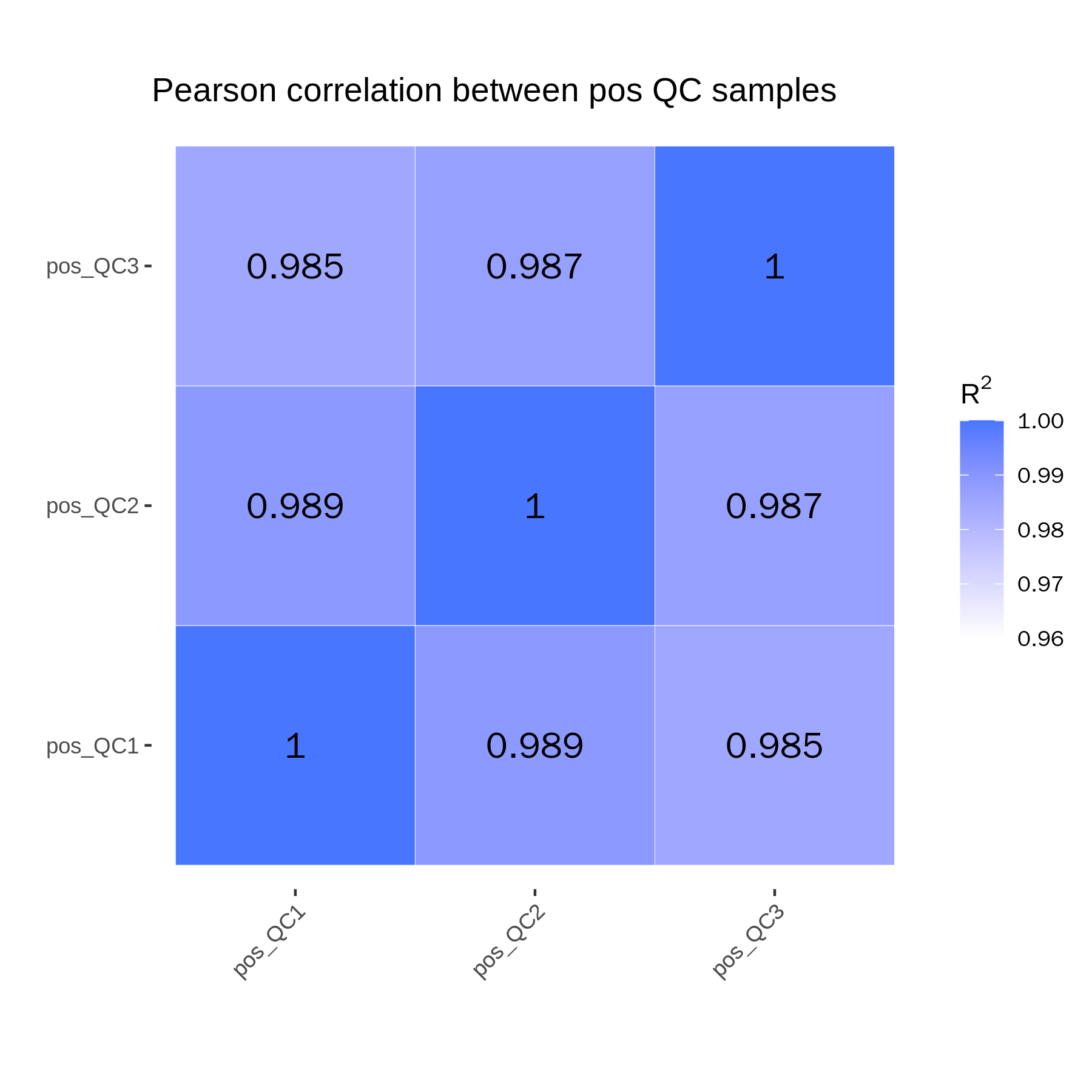

Supplement: Supplementary file 1 — Supplementary Information 1. [file 41598_2022_24687_MOESM1_ESM.zip › raw data/Metabolomics raw data/1.MetQuant-QC/Correlation/cor_pearson_pos.png]

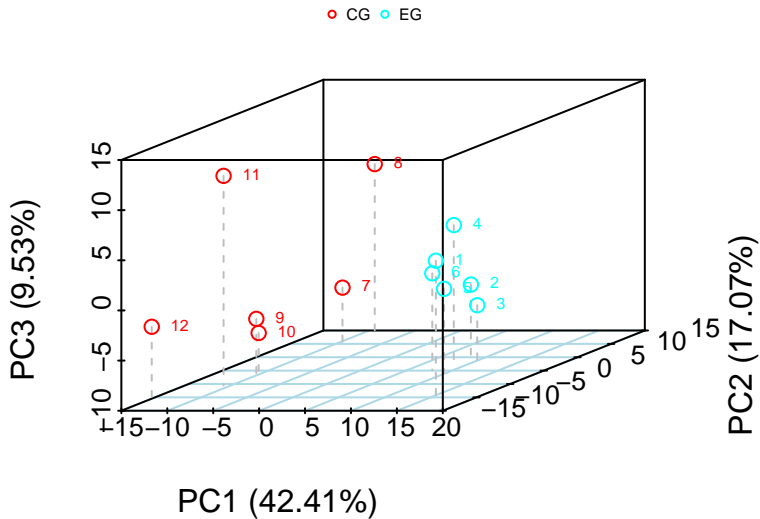

Supplement: Supplementary file 1 — Supplementary Information 1. [file 41598_2022_24687_MOESM1_ESM.zip › raw data/Metabolomics raw data/1.MetQuant-QC/Samples_neg-PCA.3D.pdf]

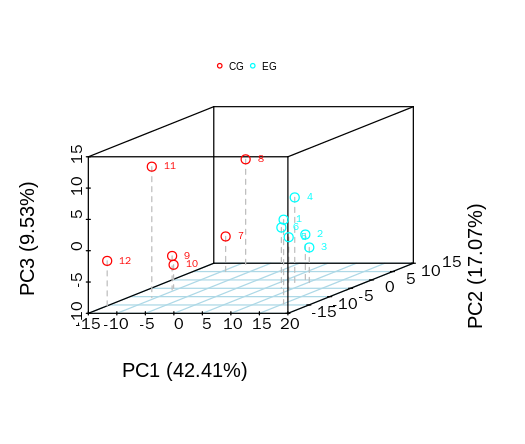

Supplement: Supplementary file 1 — Supplementary Information 1. [file 41598_2022_24687_MOESM1_ESM.zip › raw data/Metabolomics raw data/1.MetQuant-QC/Samples_neg-PCA.3D.png]

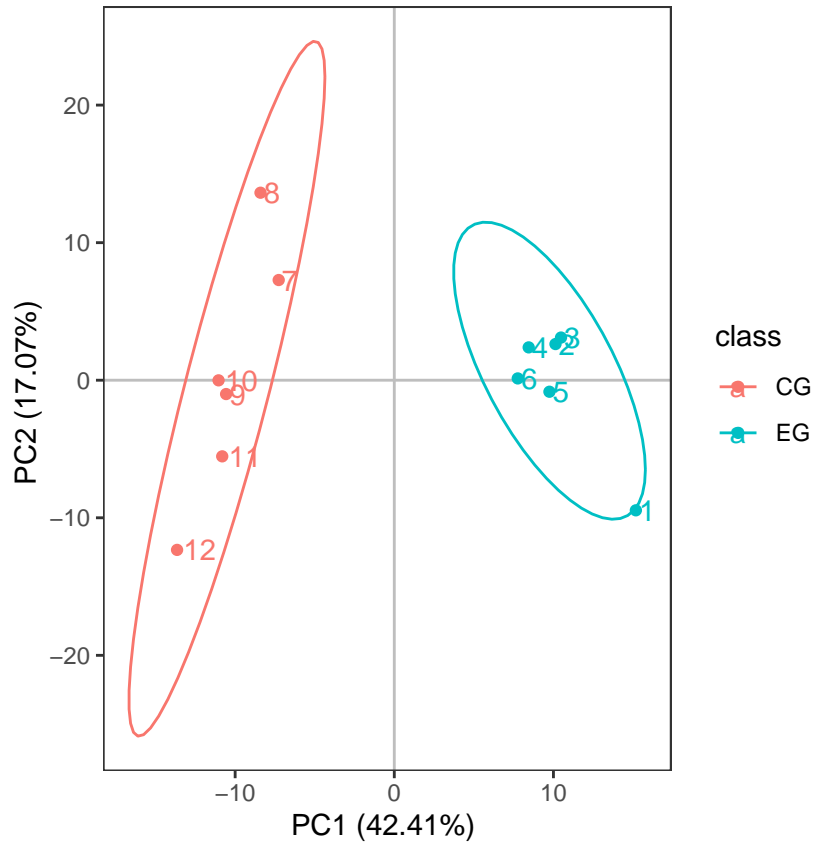

Supplement: Supplementary file 1 — Supplementary Information 1. [file 41598_2022_24687_MOESM1_ESM.zip › raw data/Metabolomics raw data/1.MetQuant-QC/Samples_neg-PCA.pdf]

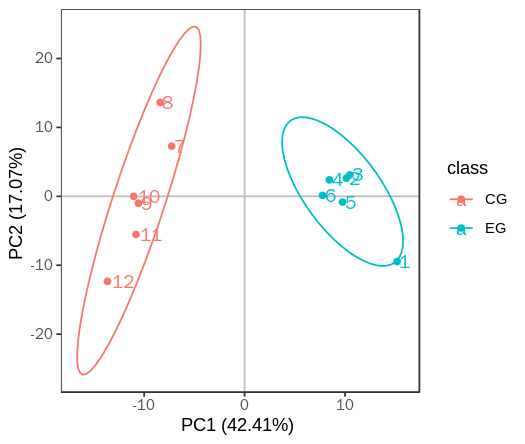

Supplement: Supplementary file 1 — Supplementary Information 1. [file 41598_2022_24687_MOESM1_ESM.zip › raw data/Metabolomics raw data/1.MetQuant-QC/Samples_neg-PCA.png]

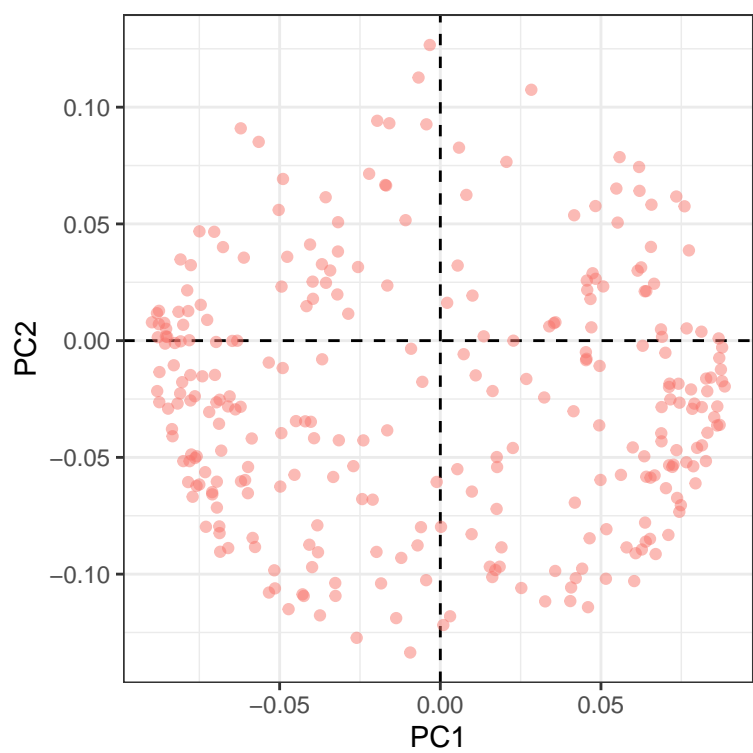

Supplement: Supplementary file 1 — Supplementary Information 1. [file 41598_2022_24687_MOESM1_ESM.zip › raw data/Metabolomics raw data/1.MetQuant-QC/Samples_neg-pcaloading.pdf]

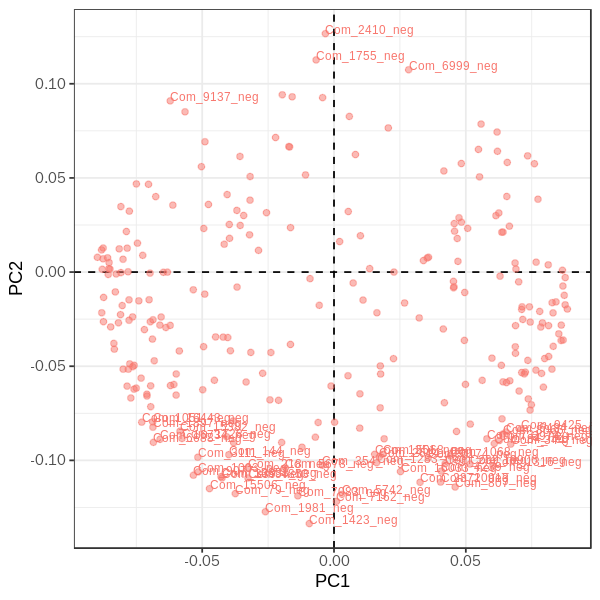

Supplement: Supplementary file 1 — Supplementary Information 1. [file 41598_2022_24687_MOESM1_ESM.zip › raw data/Metabolomics raw data/1.MetQuant-QC/Samples_neg-pcaloading.png]

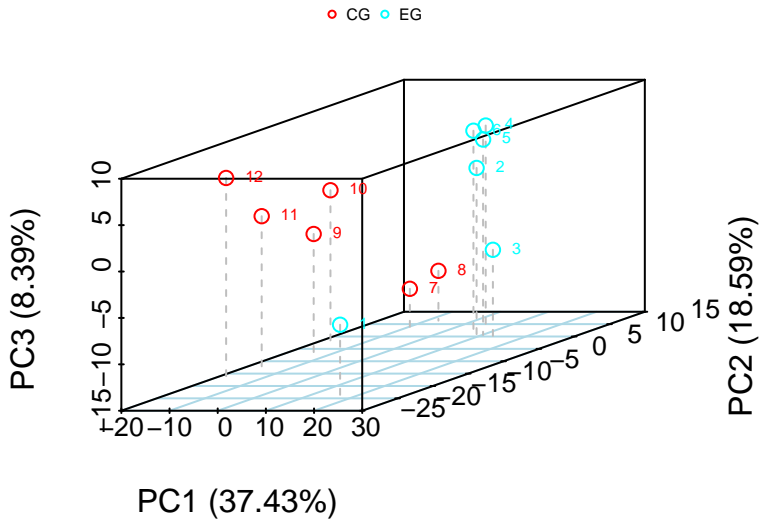

Supplement: Supplementary file 1 — Supplementary Information 1. [file 41598_2022_24687_MOESM1_ESM.zip › raw data/Metabolomics raw data/1.MetQuant-QC/Samples_pos-PCA.3D.pdf]

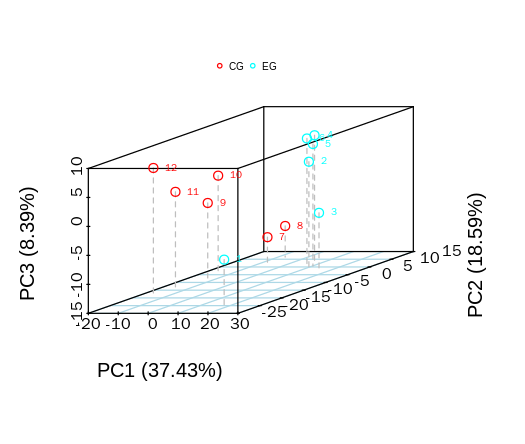

Supplement: Supplementary file 1 — Supplementary Information 1. [file 41598_2022_24687_MOESM1_ESM.zip › raw data/Metabolomics raw data/1.MetQuant-QC/Samples_pos-PCA.3D.png]

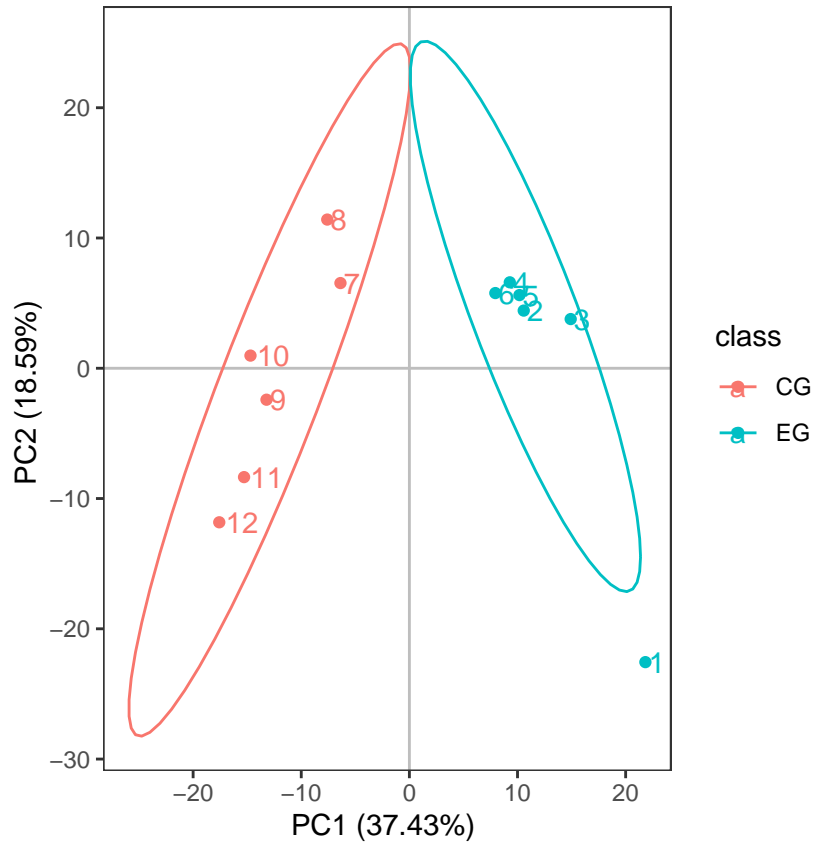

Supplement: Supplementary file 1 — Supplementary Information 1. [file 41598_2022_24687_MOESM1_ESM.zip › raw data/Metabolomics raw data/1.MetQuant-QC/Samples_pos-PCA.pdf]

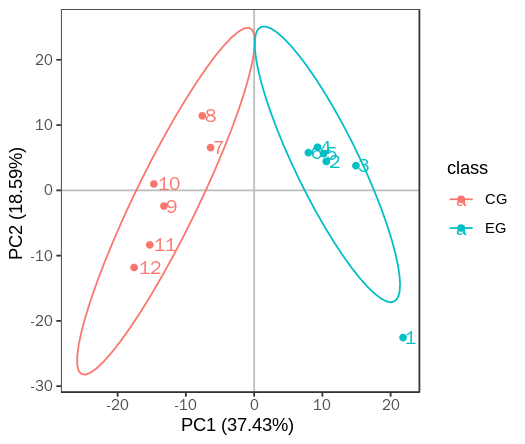

Supplement: Supplementary file 1 — Supplementary Information 1. [file 41598_2022_24687_MOESM1_ESM.zip › raw data/Metabolomics raw data/1.MetQuant-QC/Samples_pos-PCA.png]

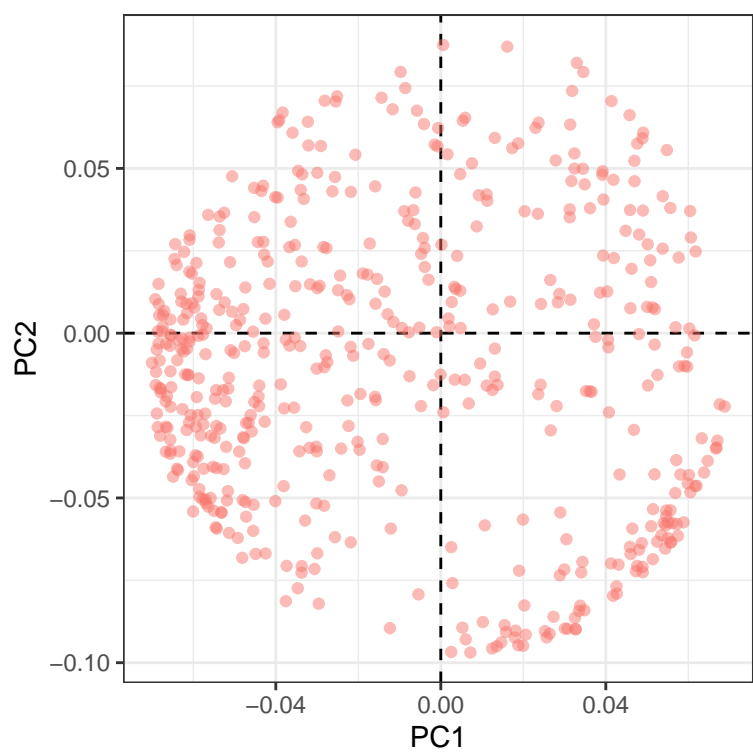

Supplement: Supplementary file 1 — Supplementary Information 1. [file 41598_2022_24687_MOESM1_ESM.zip › raw data/Metabolomics raw data/1.MetQuant-QC/Samples_pos-pcaloading.pdf]

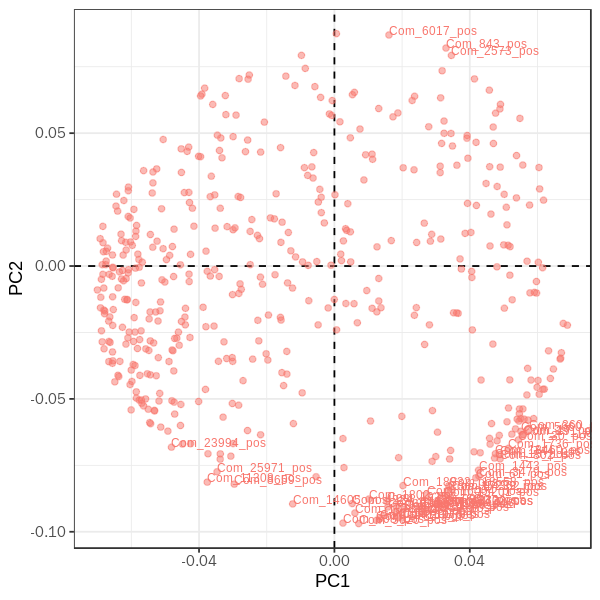

Supplement: Supplementary file 1 — Supplementary Information 1. [file 41598_2022_24687_MOESM1_ESM.zip › raw data/Metabolomics raw data/1.MetQuant-QC/Samples_pos-pcaloading.png]

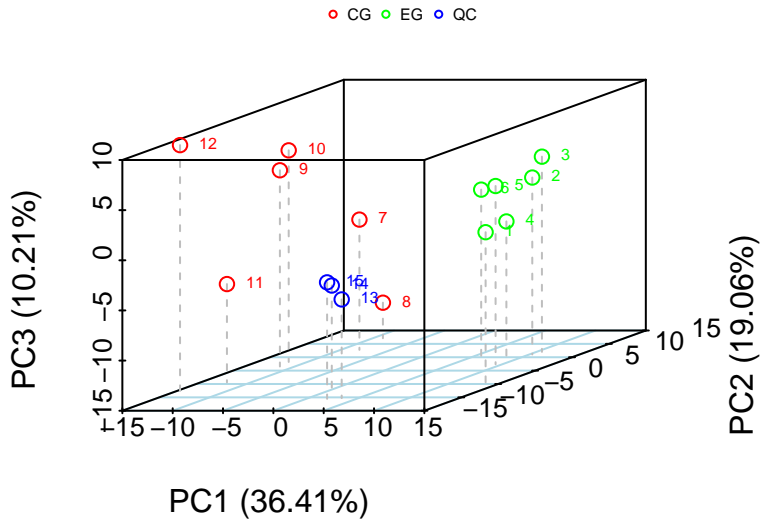

Supplement: Supplementary file 1 — Supplementary Information 1. [file 41598_2022_24687_MOESM1_ESM.zip › raw data/Metabolomics raw data/1.MetQuant-QC/Samples_QC_neg-PCA.3D.pdf]

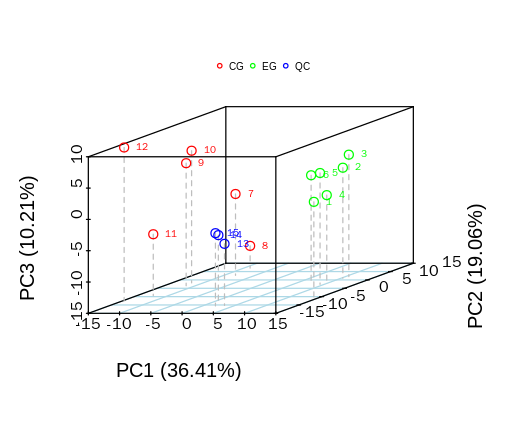

Supplement: Supplementary file 1 — Supplementary Information 1. [file 41598_2022_24687_MOESM1_ESM.zip › raw data/Metabolomics raw data/1.MetQuant-QC/Samples_QC_neg-PCA.3D.png]

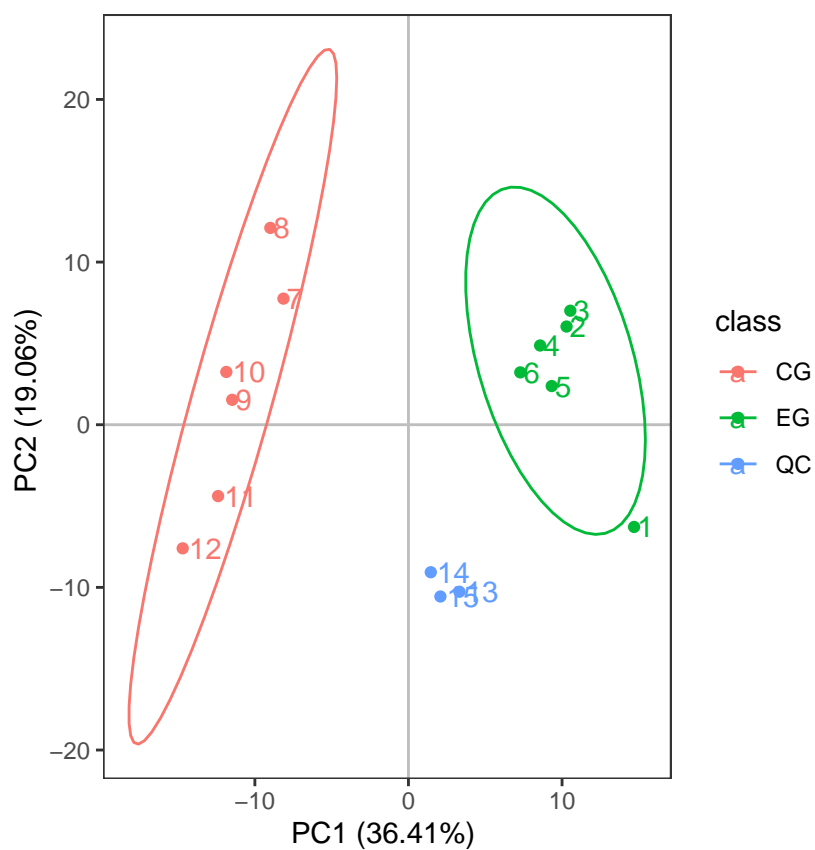

Supplement: Supplementary file 1 — Supplementary Information 1. [file 41598_2022_24687_MOESM1_ESM.zip › raw data/Metabolomics raw data/1.MetQuant-QC/Samples_QC_neg-PCA.pdf]

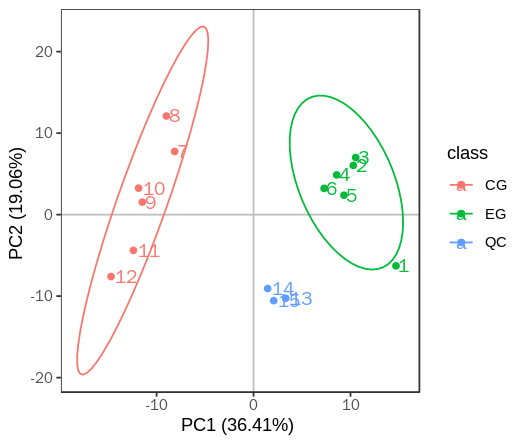

Supplement: Supplementary file 1 — Supplementary Information 1. [file 41598_2022_24687_MOESM1_ESM.zip › raw data/Metabolomics raw data/1.MetQuant-QC/Samples_QC_neg-PCA.png]

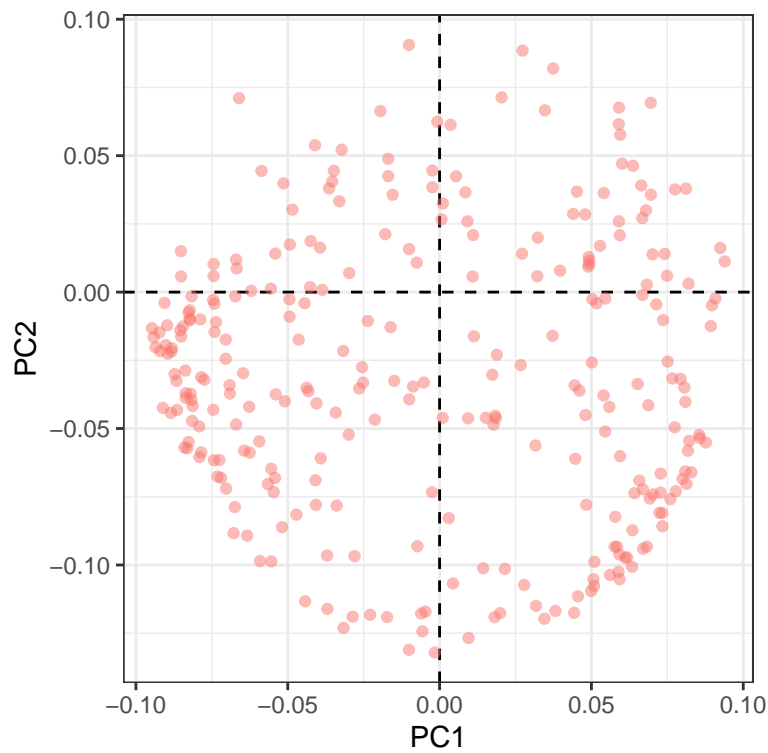

Supplement: Supplementary file 1 — Supplementary Information 1. [file 41598_2022_24687_MOESM1_ESM.zip › raw data/Metabolomics raw data/1.MetQuant-QC/Samples_QC_neg-pcaloading.pdf]

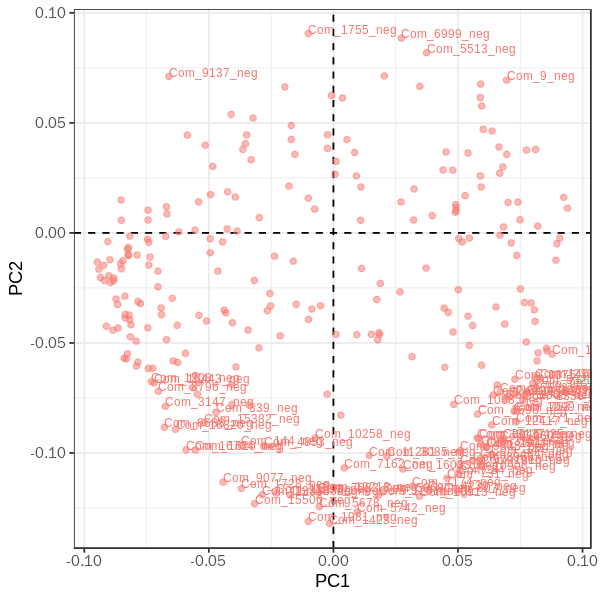

Supplement: Supplementary file 1 — Supplementary Information 1. [file 41598_2022_24687_MOESM1_ESM.zip › raw data/Metabolomics raw data/1.MetQuant-QC/Samples_QC_neg-pcaloading.png]

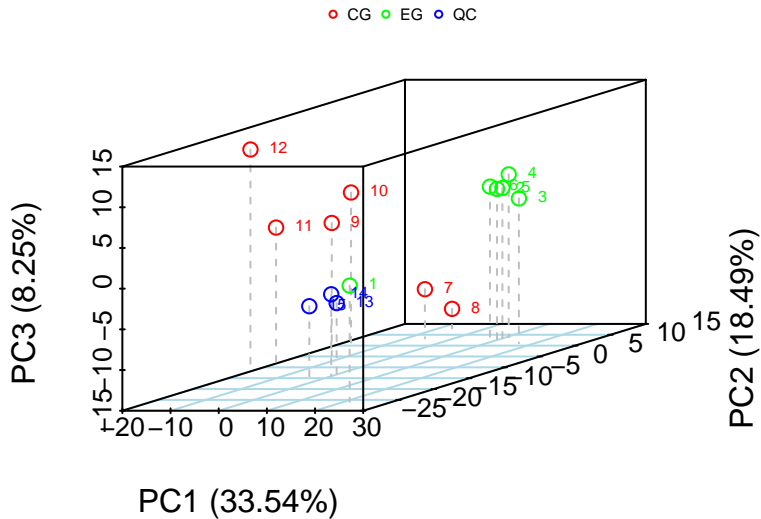

Supplement: Supplementary file 1 — Supplementary Information 1. [file 41598_2022_24687_MOESM1_ESM.zip › raw data/Metabolomics raw data/1.MetQuant-QC/Samples_QC_pos-PCA.3D.pdf]

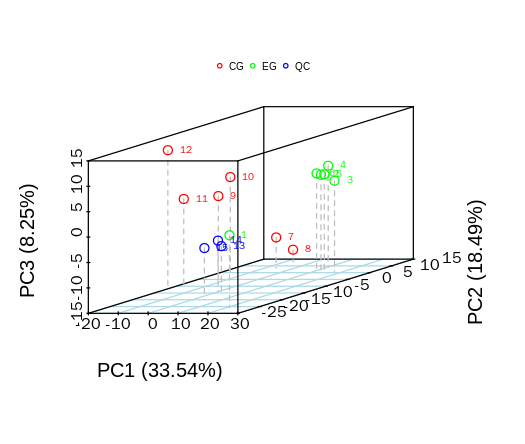

Supplement: Supplementary file 1 — Supplementary Information 1. [file 41598_2022_24687_MOESM1_ESM.zip › raw data/Metabolomics raw data/1.MetQuant-QC/Samples_QC_pos-PCA.3D.png]

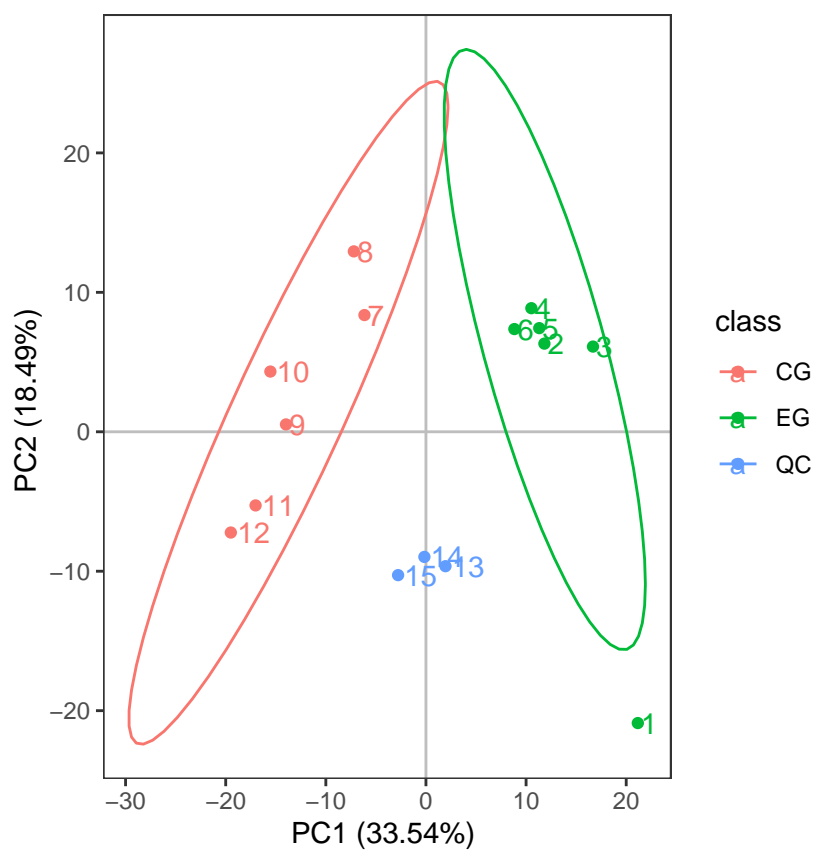

Supplement: Supplementary file 1 — Supplementary Information 1. [file 41598_2022_24687_MOESM1_ESM.zip › raw data/Metabolomics raw data/1.MetQuant-QC/Samples_QC_pos-PCA.pdf]

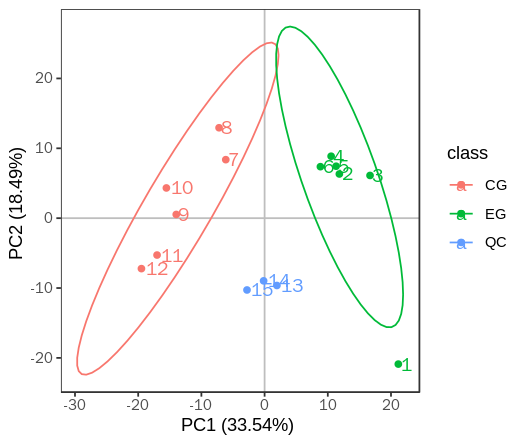

Supplement: Supplementary file 1 — Supplementary Information 1. [file 41598_2022_24687_MOESM1_ESM.zip › raw data/Metabolomics raw data/1.MetQuant-QC/Samples_QC_pos-PCA.png]

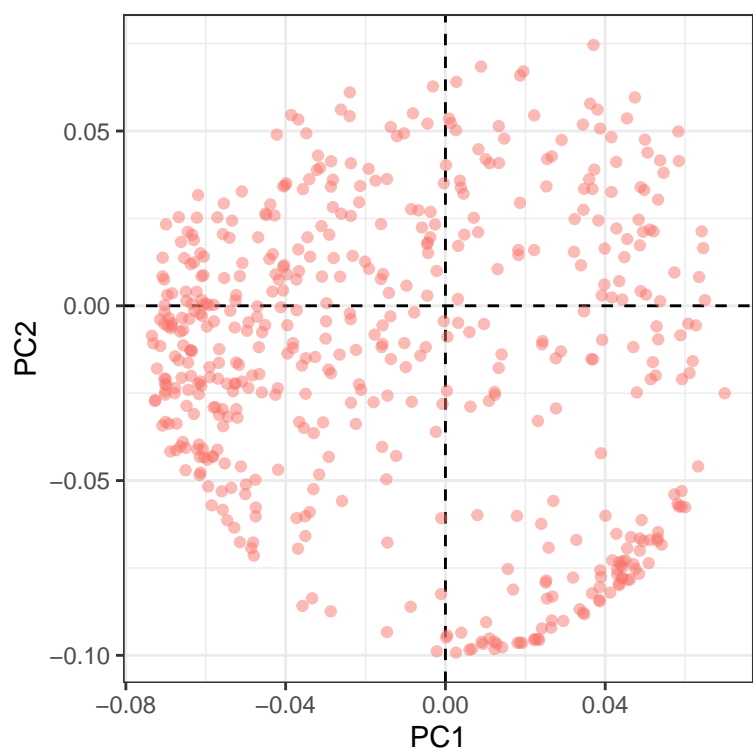

Supplement: Supplementary file 1 — Supplementary Information 1. [file 41598_2022_24687_MOESM1_ESM.zip › raw data/Metabolomics raw data/1.MetQuant-QC/Samples_QC_pos-pcaloading.pdf]

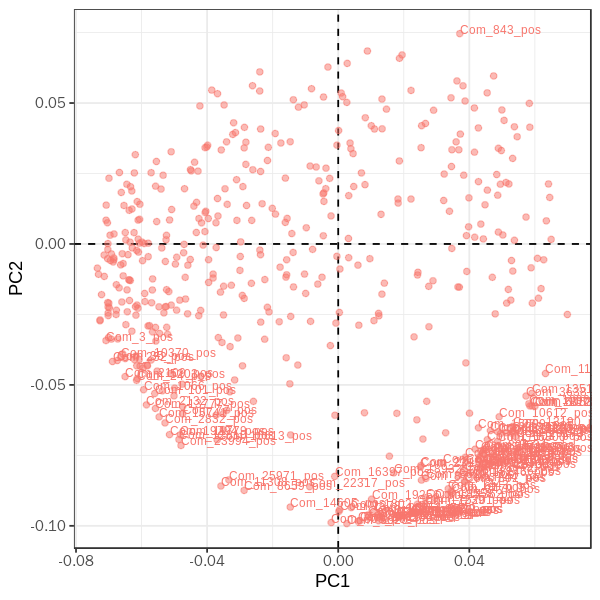

Supplement: Supplementary file 1 — Supplementary Information 1. [file 41598_2022_24687_MOESM1_ESM.zip › raw data/Metabolomics raw data/1.MetQuant-QC/Samples_QC_pos-pcaloading.png]

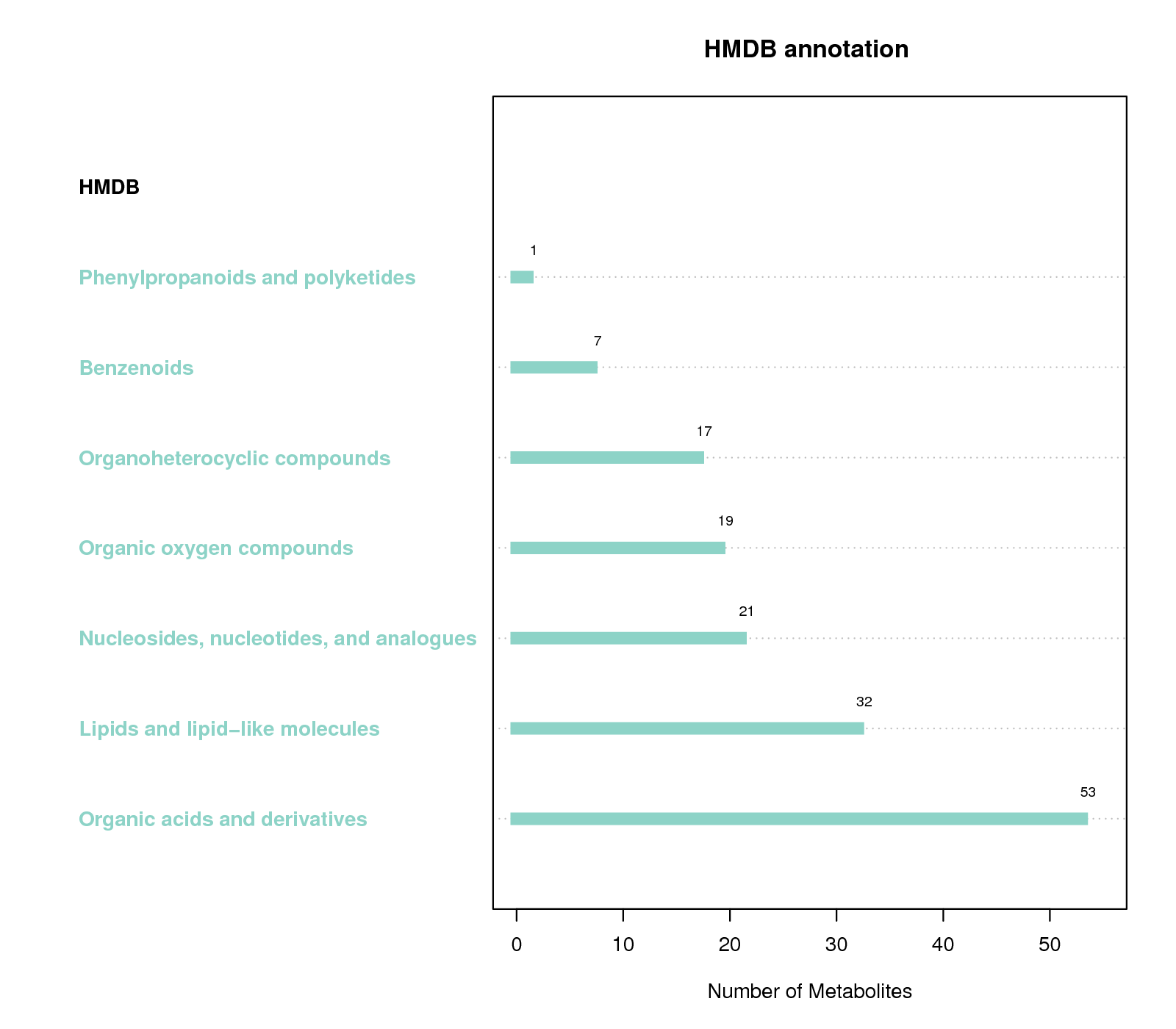

Supplement: Supplementary file 1 — Supplementary Information 1. [file 41598_2022_24687_MOESM1_ESM.zip › raw data/Metabolomics raw data/2.MetAnnotation/HMDB/meta_neg.HMDB.Anno.png]

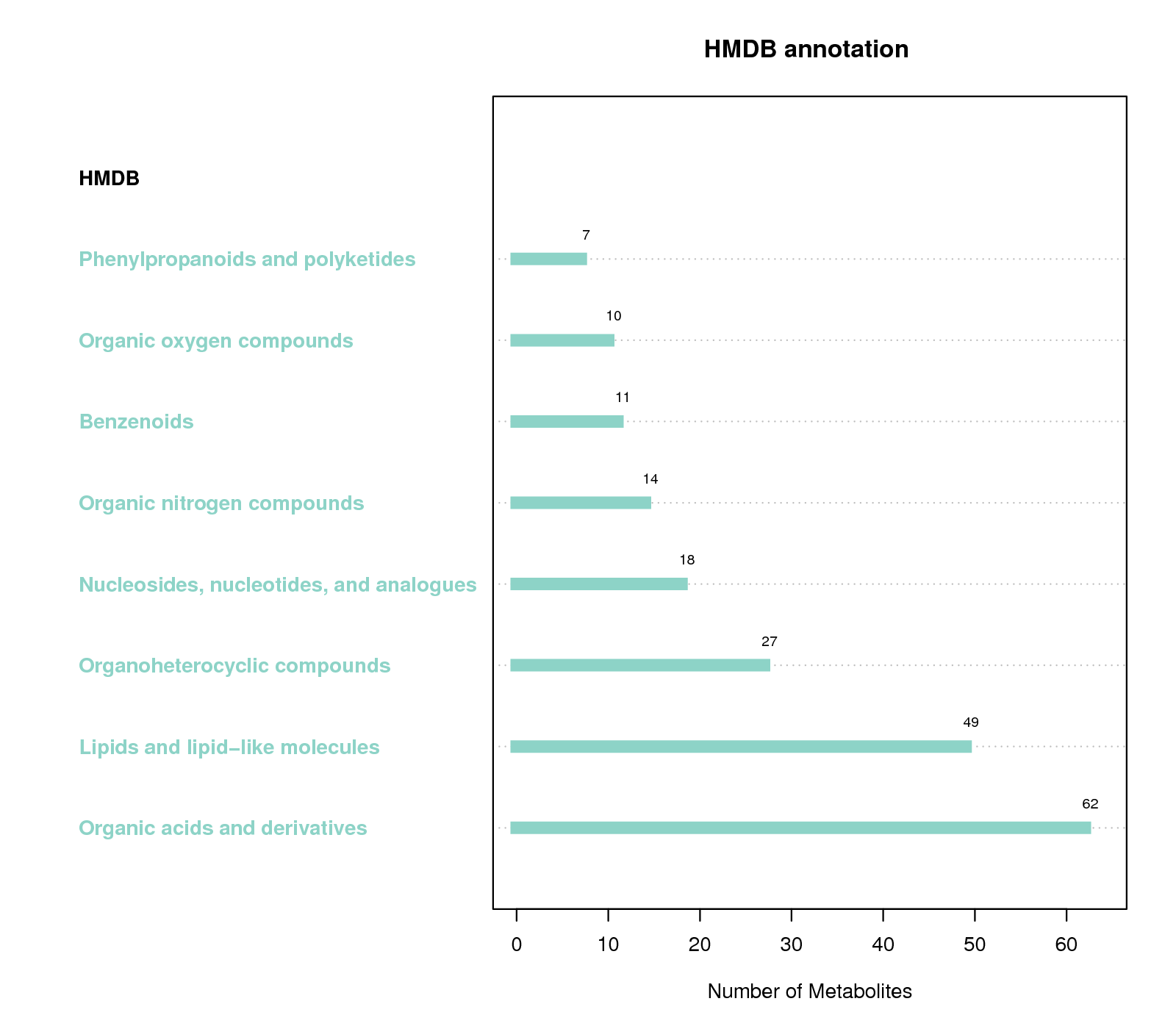

Supplement: Supplementary file 1 — Supplementary Information 1. [file 41598_2022_24687_MOESM1_ESM.zip › raw data/Metabolomics raw data/2.MetAnnotation/HMDB/meta_pos.HMDB.Anno.png]

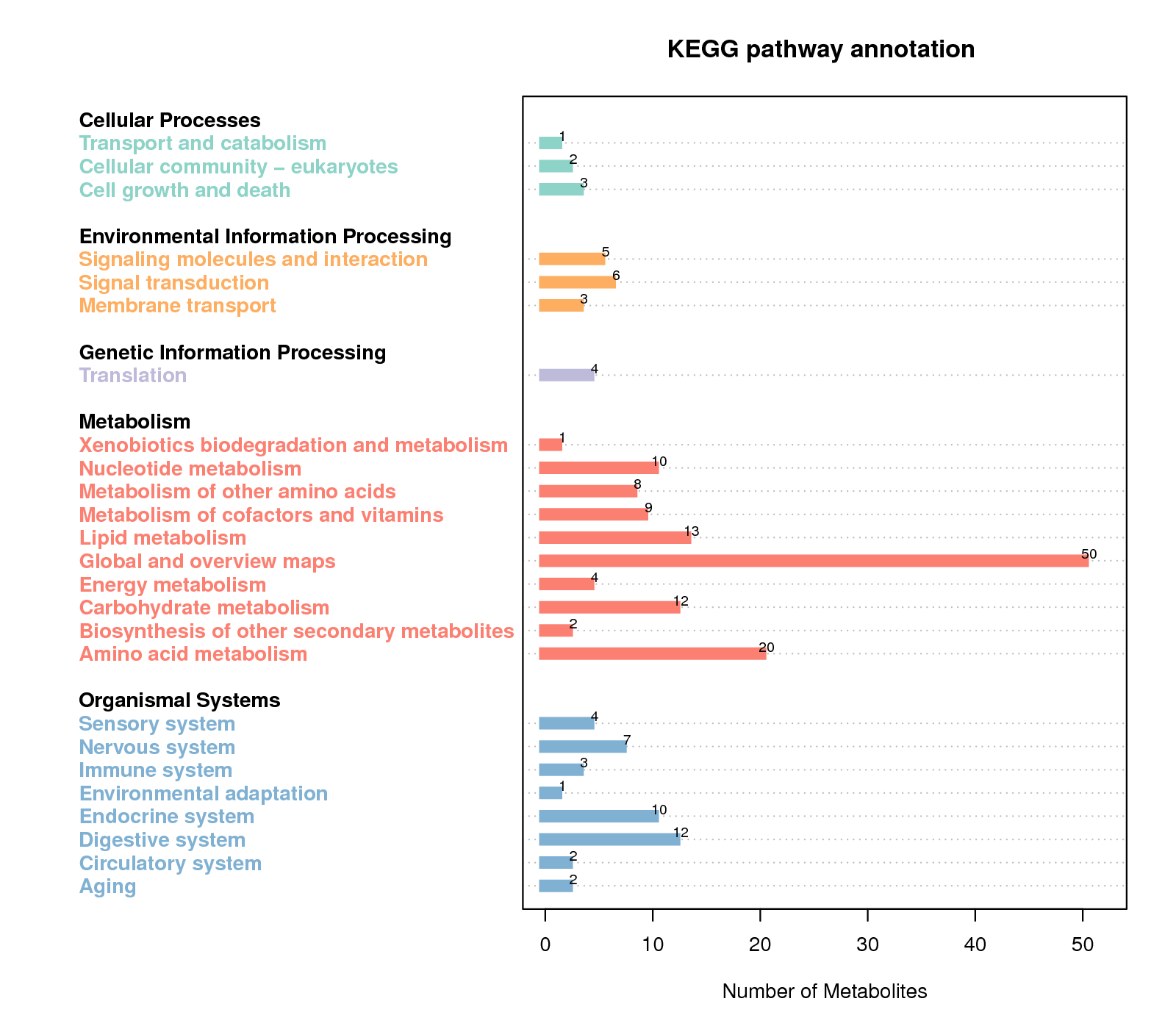

Supplement: Supplementary file 1 — Supplementary Information 1. [file 41598_2022_24687_MOESM1_ESM.zip › raw data/Metabolomics raw data/2.MetAnnotation/KEGG/meta_neg.KEGG.Anno.png]

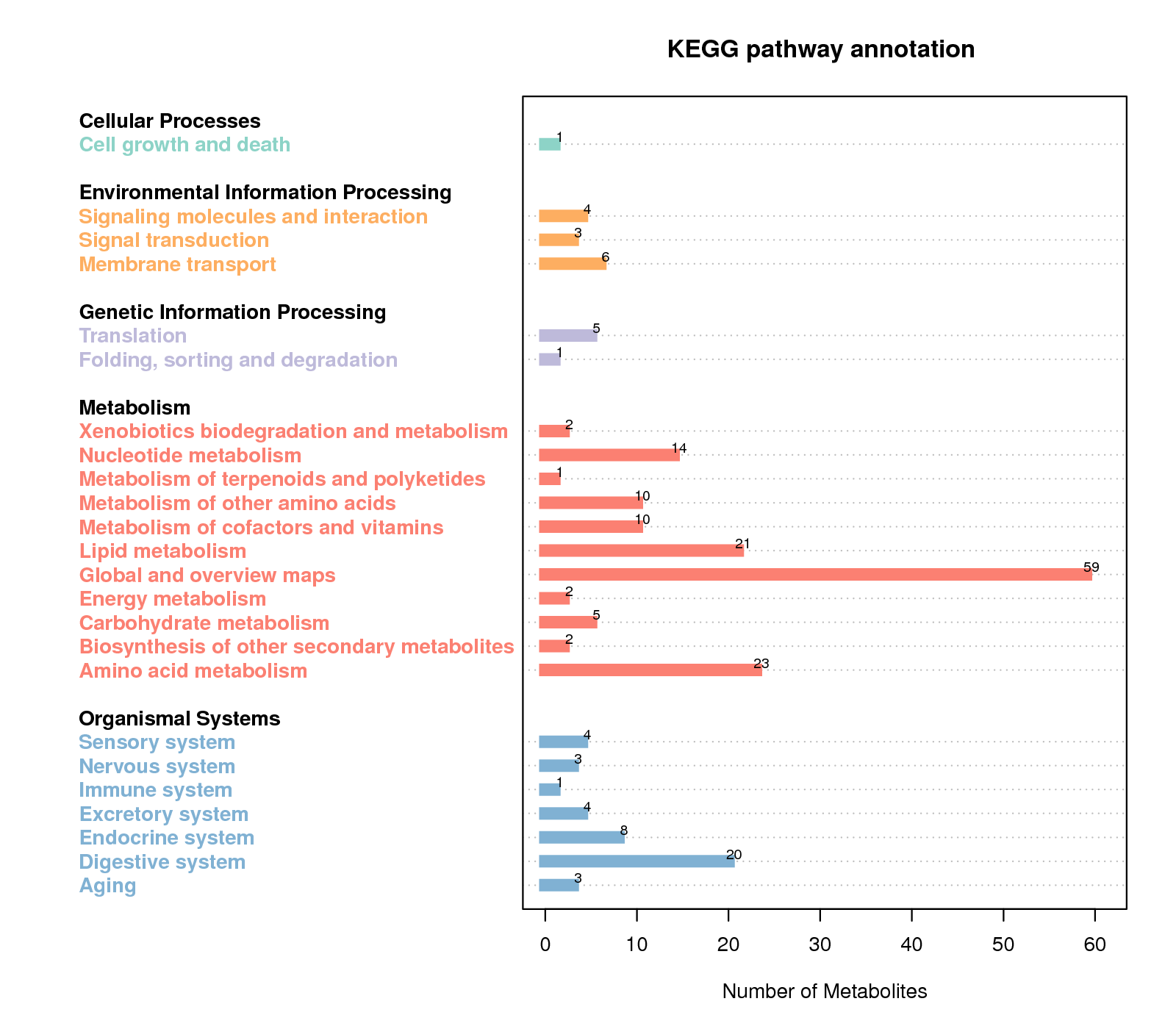

Supplement: Supplementary file 1 — Supplementary Information 1. [file 41598_2022_24687_MOESM1_ESM.zip › raw data/Metabolomics raw data/2.MetAnnotation/KEGG/meta_pos.KEGG.Anno.png]

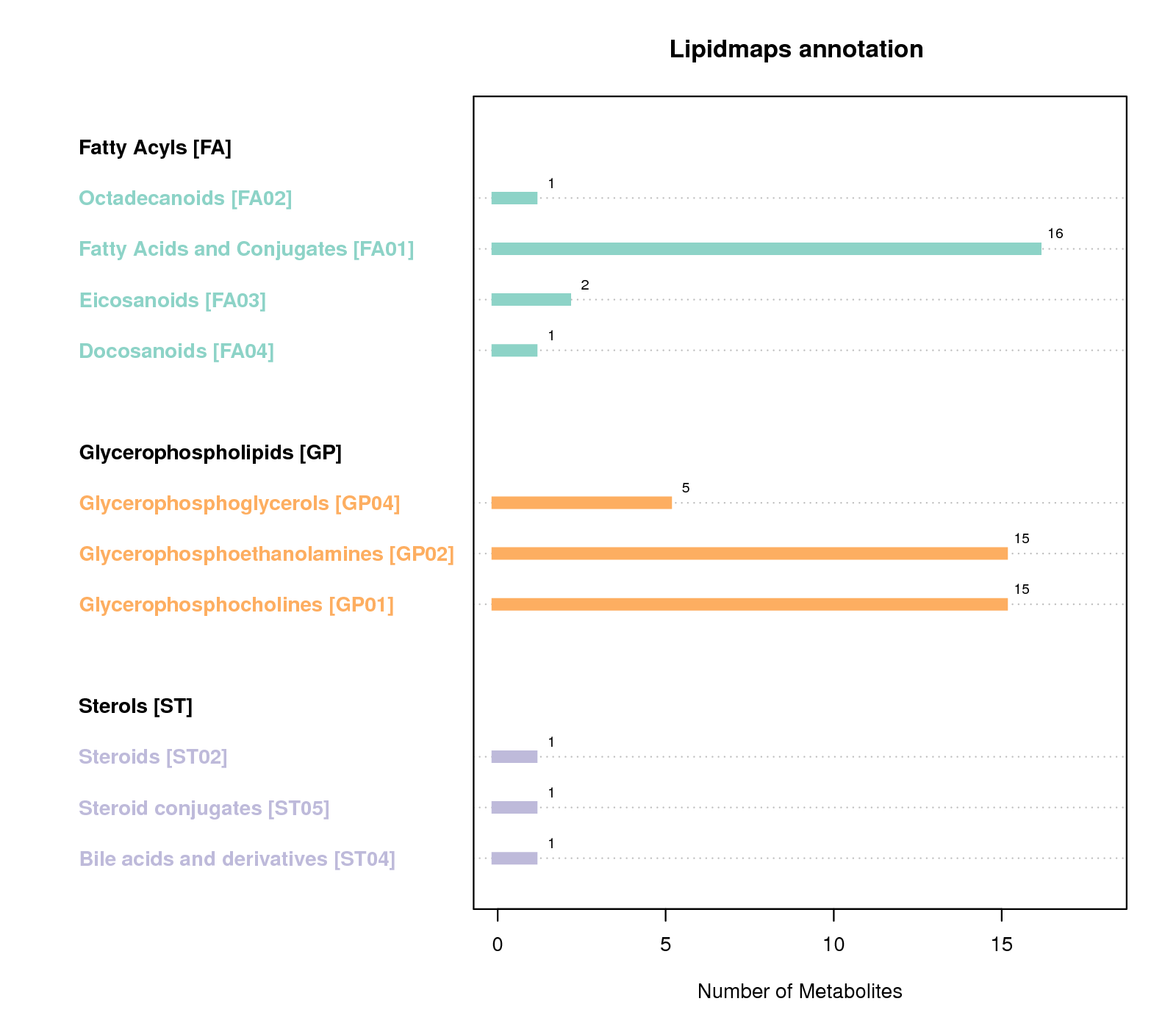

Supplement: Supplementary file 1 — Supplementary Information 1. [file 41598_2022_24687_MOESM1_ESM.zip › raw data/Metabolomics raw data/2.MetAnnotation/Lipidmaps/meta_neg.Lipidmaps.Anno.png]

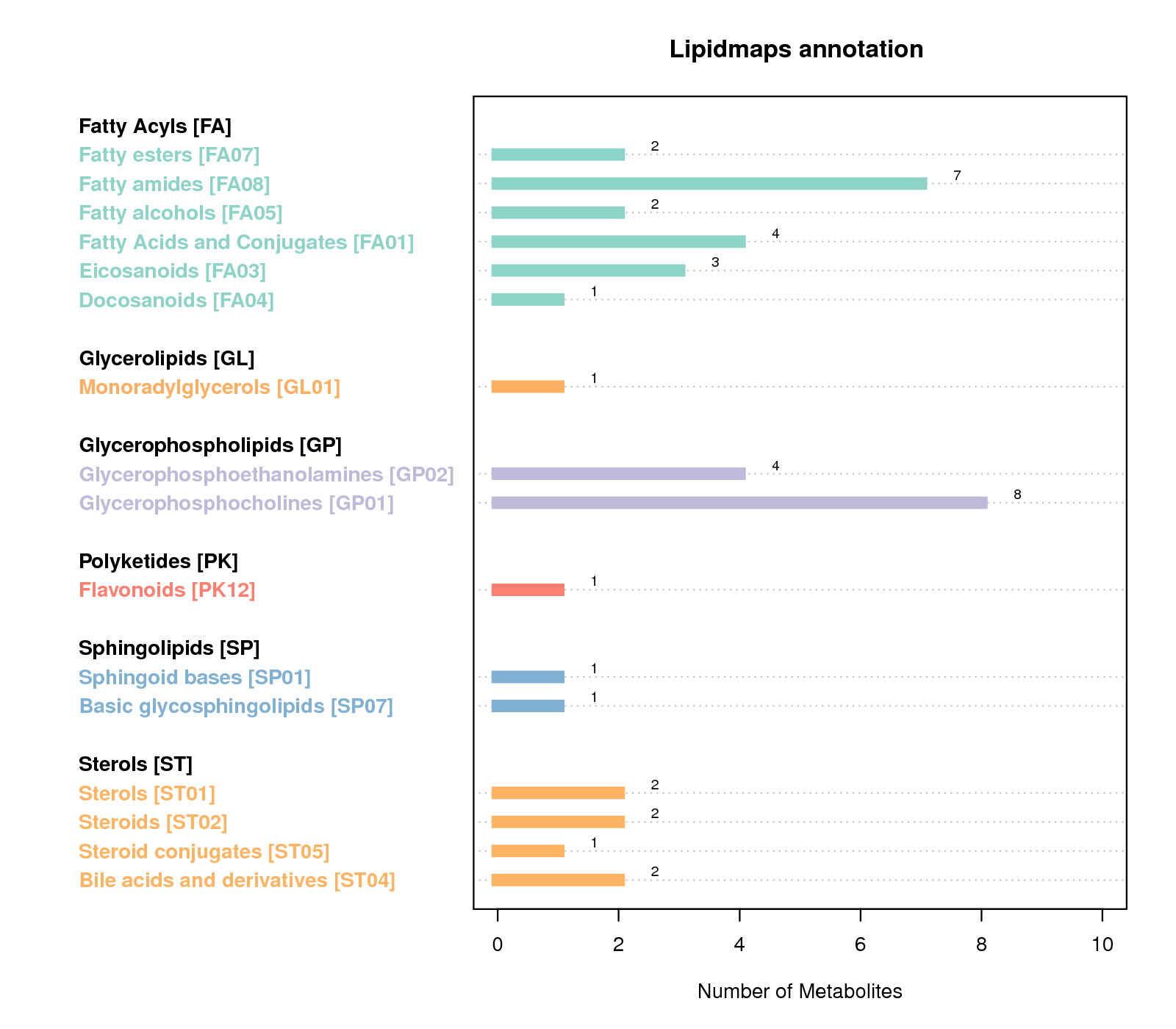

Supplement: Supplementary file 1 — Supplementary Information 1. [file 41598_2022_24687_MOESM1_ESM.zip › raw data/Metabolomics raw data/2.MetAnnotation/Lipidmaps/meta_pos.Lipidmaps.Anno.png]

# EG.vs.CG

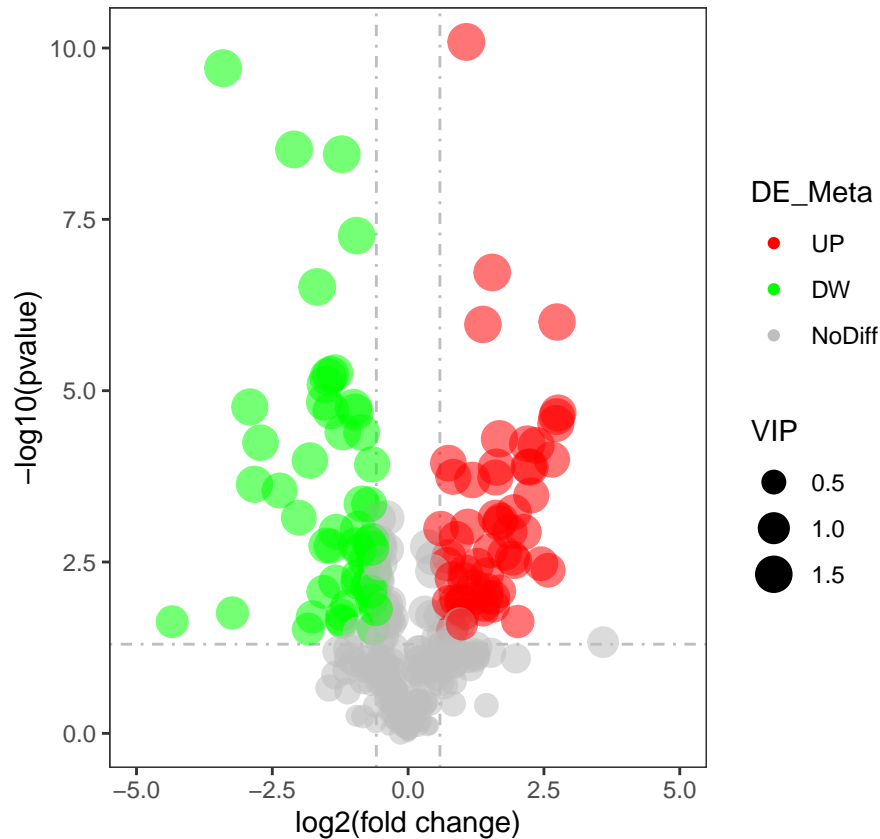

Supplement: Supplementary file 1 — Supplementary Information 1. [file 41598_2022_24687_MOESM1_ESM.zip › raw data/Metabolomics raw data/3.MetDiffScreening/EG.vs.CG/EG.vs.CG_neg.xls.volcano.pdf]

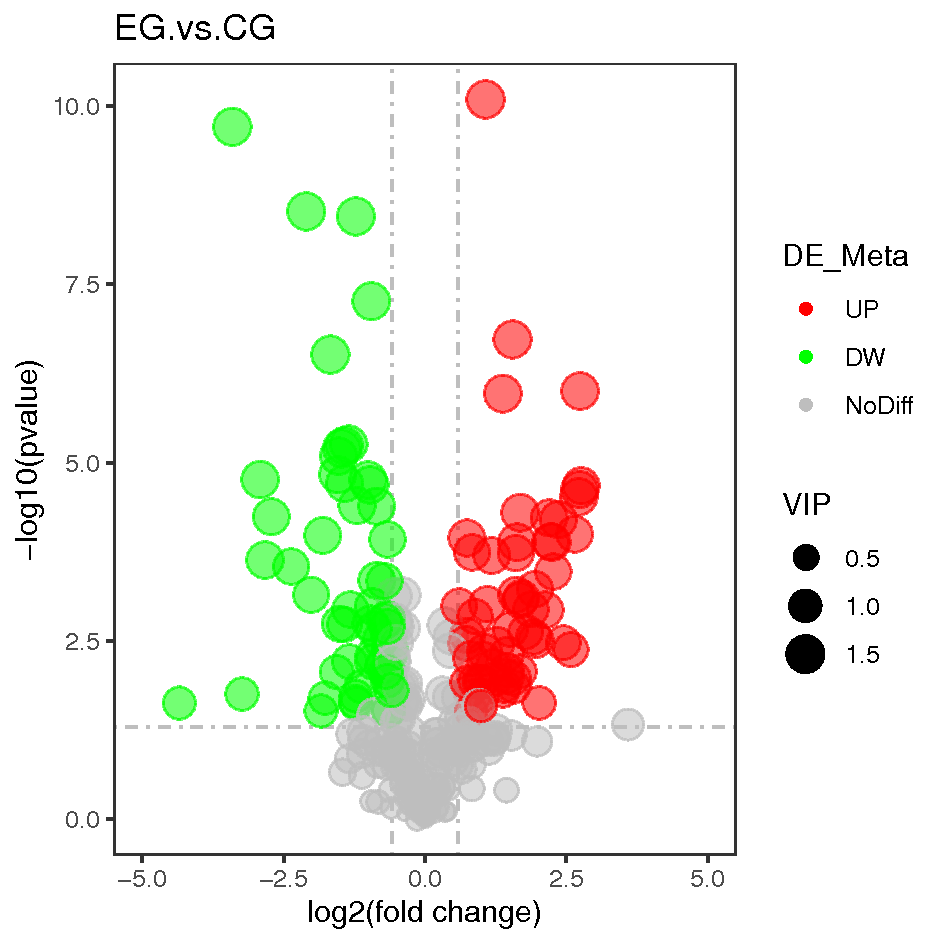

Supplement: Supplementary file 1 — Supplementary Information 1. [file 41598_2022_24687_MOESM1_ESM.zip › raw data/Metabolomics raw data/3.MetDiffScreening/EG.vs.CG/EG.vs.CG_neg.xls.volcano.png]

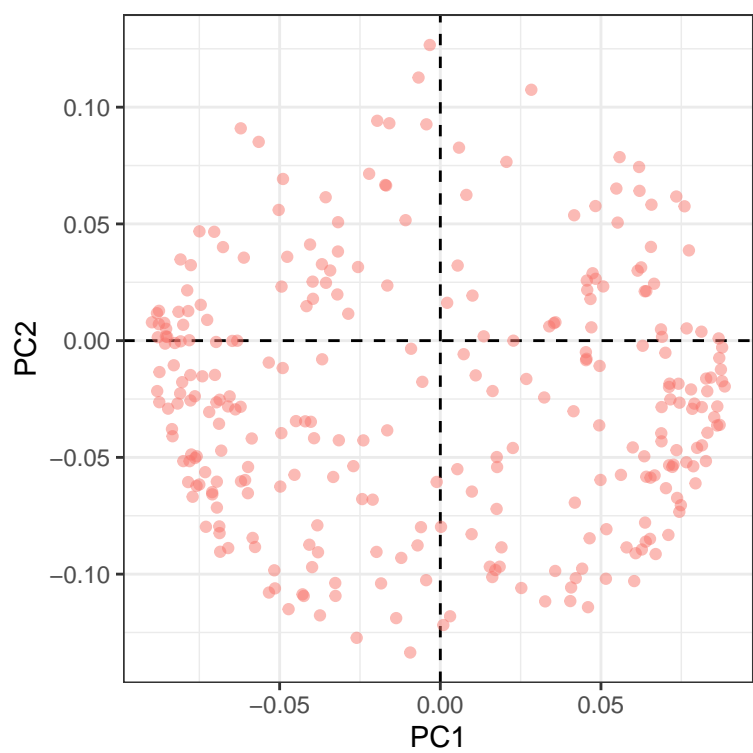

Supplement: Supplementary file 1 — Supplementary Information 1. [file 41598_2022_24687_MOESM1_ESM.zip › raw data/Metabolomics raw data/3.MetDiffScreening/EG.vs.CG/EG.vs.CG_neg_PCA-pcaloading.pdf]

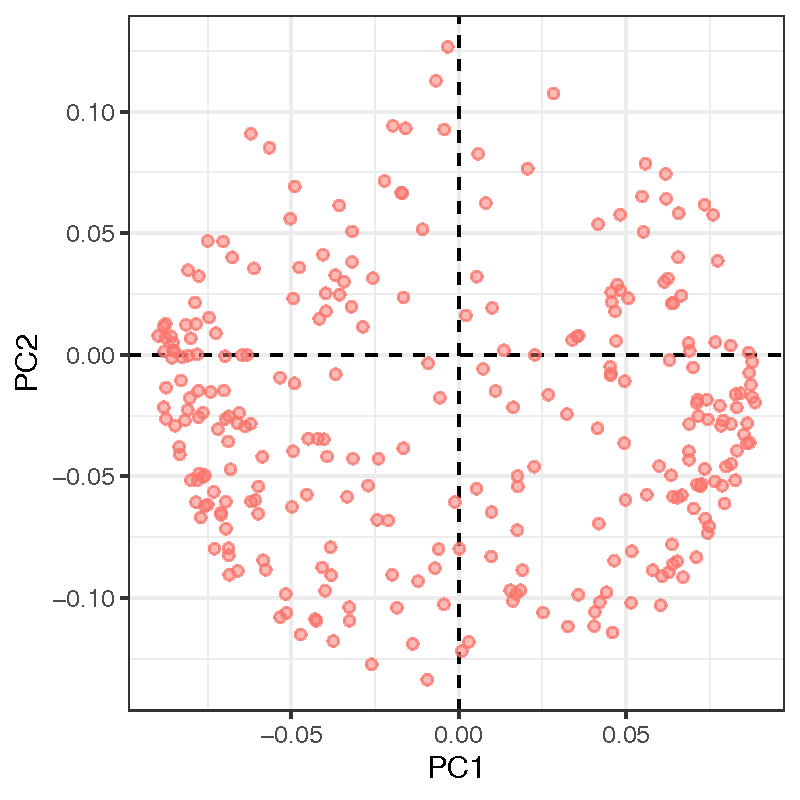

Supplement: Supplementary file 1 — Supplementary Information 1. [file 41598_2022_24687_MOESM1_ESM.zip › raw data/Metabolomics raw data/3.MetDiffScreening/EG.vs.CG/EG.vs.CG_neg_PCA-pcaloading.png]

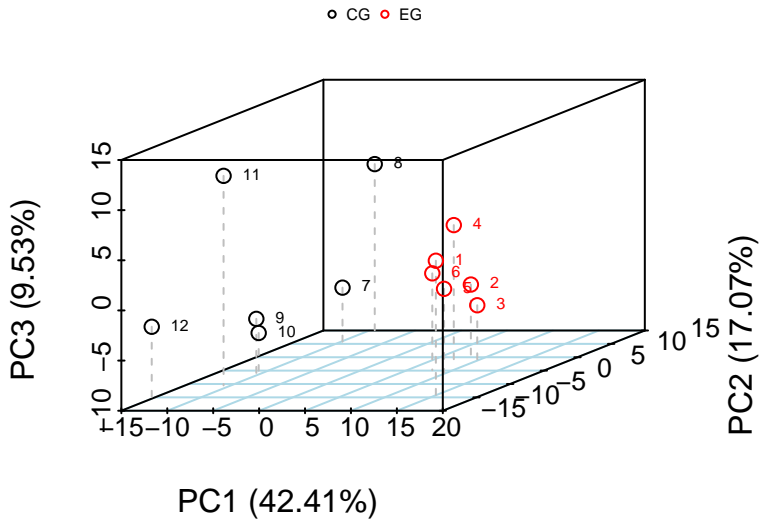

Supplement: Supplementary file 1 — Supplementary Information 1. [file 41598_2022_24687_MOESM1_ESM.zip › raw data/Metabolomics raw data/3.MetDiffScreening/EG.vs.CG/EG.vs.CG_neg_PCA.3D.pdf]

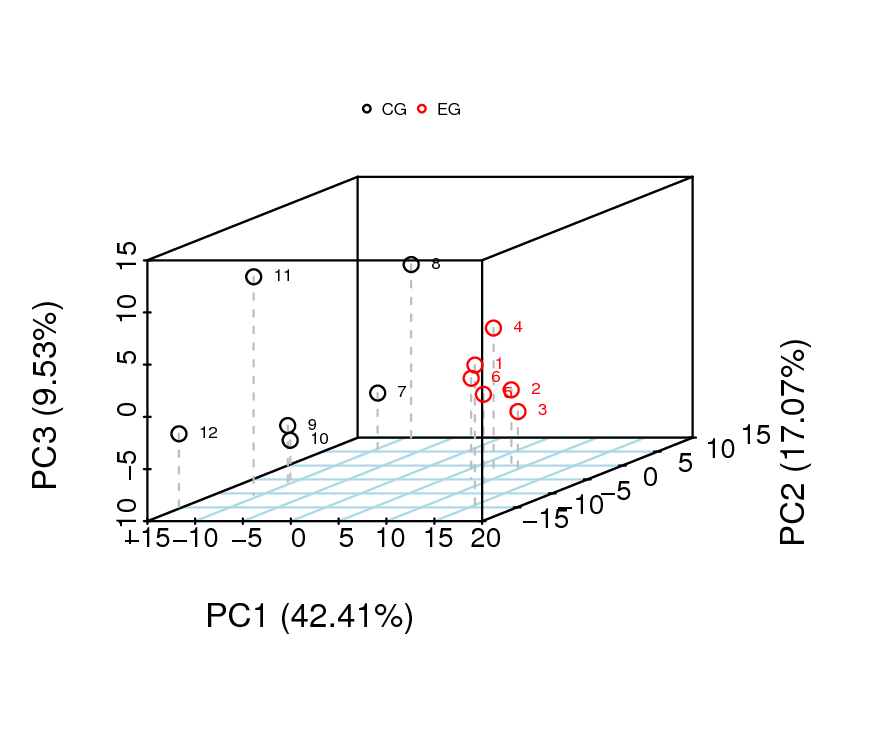

Supplement: Supplementary file 1 — Supplementary Information 1. [file 41598_2022_24687_MOESM1_ESM.zip › raw data/Metabolomics raw data/3.MetDiffScreening/EG.vs.CG/EG.vs.CG_neg_PCA.3D.png]

class    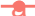 CG    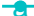 EG

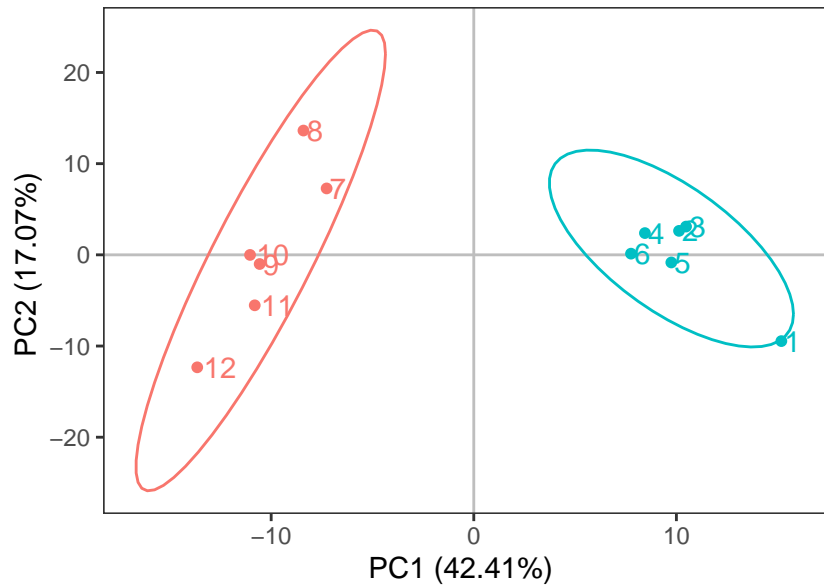

Supplement: Supplementary file 1 — Supplementary Information 1. [file 41598_2022_24687_MOESM1_ESM.zip › raw data/Metabolomics raw data/3.MetDiffScreening/EG.vs.CG/EG.vs.CG_neg_PCA.pdf]

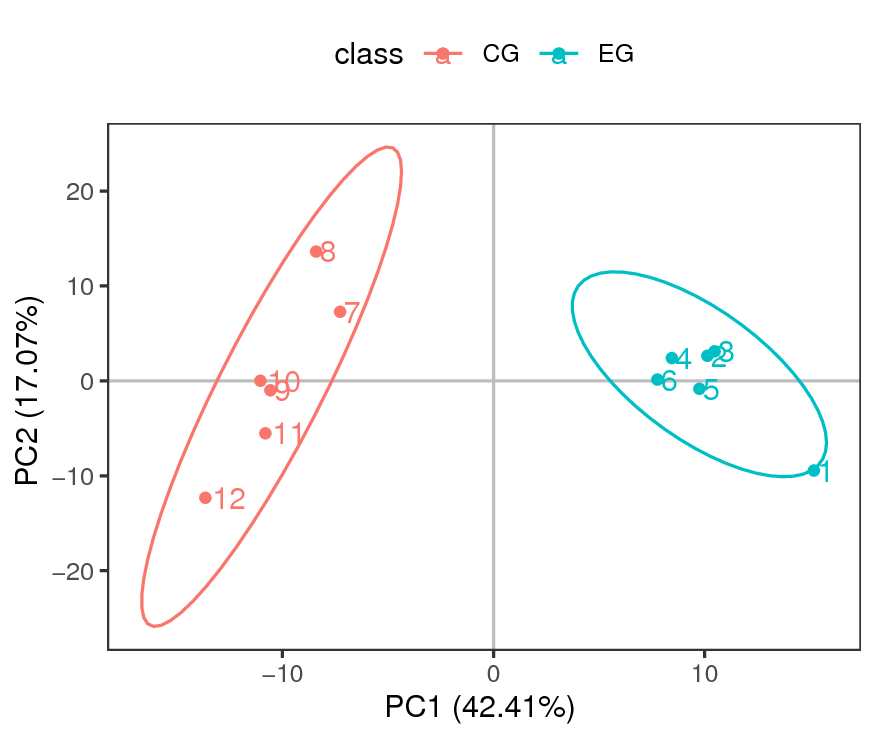

Supplement: Supplementary file 1 — Supplementary Information 1. [file 41598_2022_24687_MOESM1_ESM.zip › raw data/Metabolomics raw data/3.MetDiffScreening/EG.vs.CG/EG.vs.CG_neg_PCA.png]

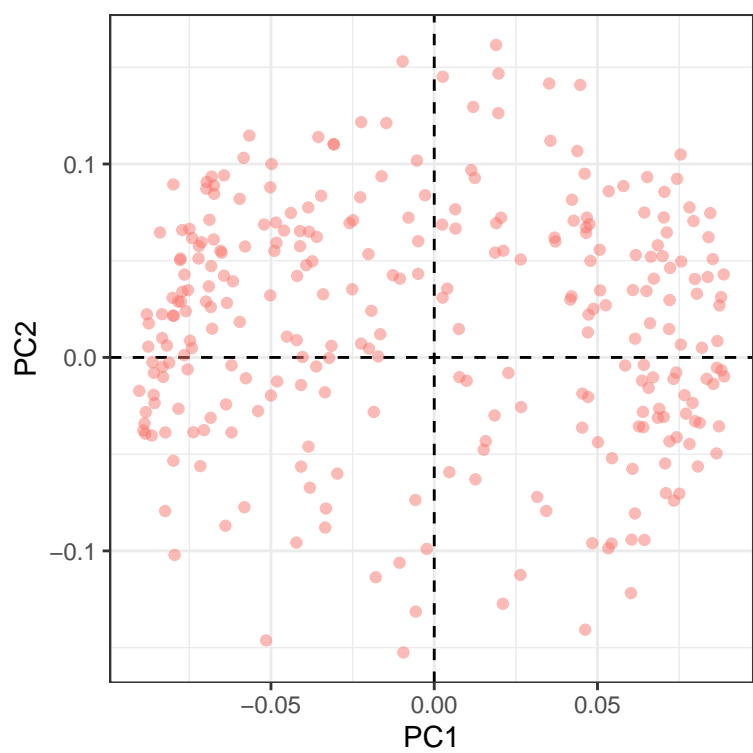

Supplement: Supplementary file 1 — Supplementary Information 1. [file 41598_2022_24687_MOESM1_ESM.zip › raw data/Metabolomics raw data/3.MetDiffScreening/EG.vs.CG/EG.vs.CG_neg_PLSDA-loading.pdf]

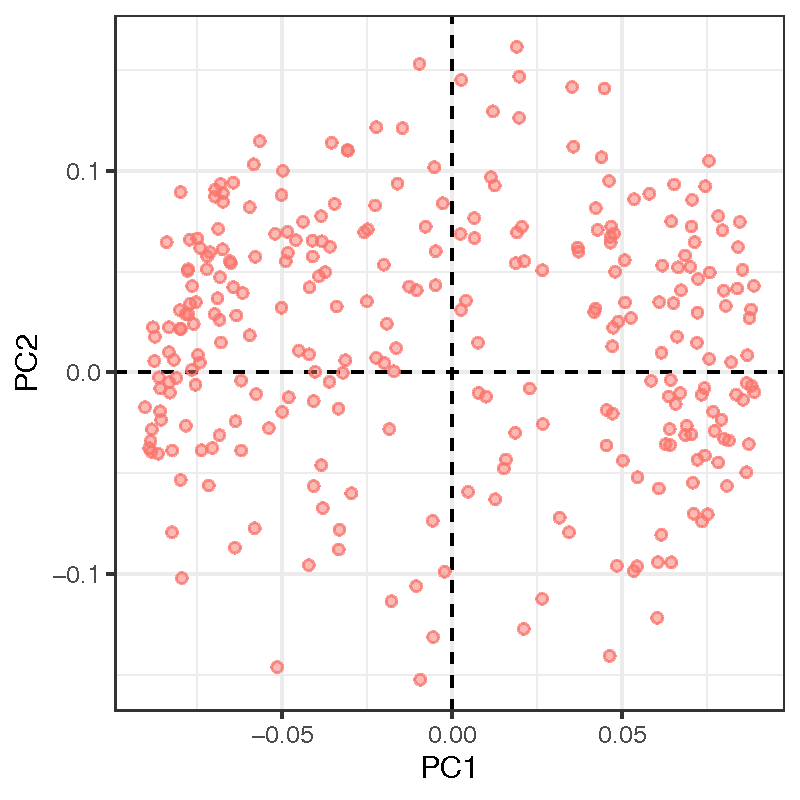

Supplement: Supplementary file 1 — Supplementary Information 1. [file 41598_2022_24687_MOESM1_ESM.zip › raw data/Metabolomics raw data/3.MetDiffScreening/EG.vs.CG/EG.vs.CG_neg_PLSDA-loading.png]

class CG EG

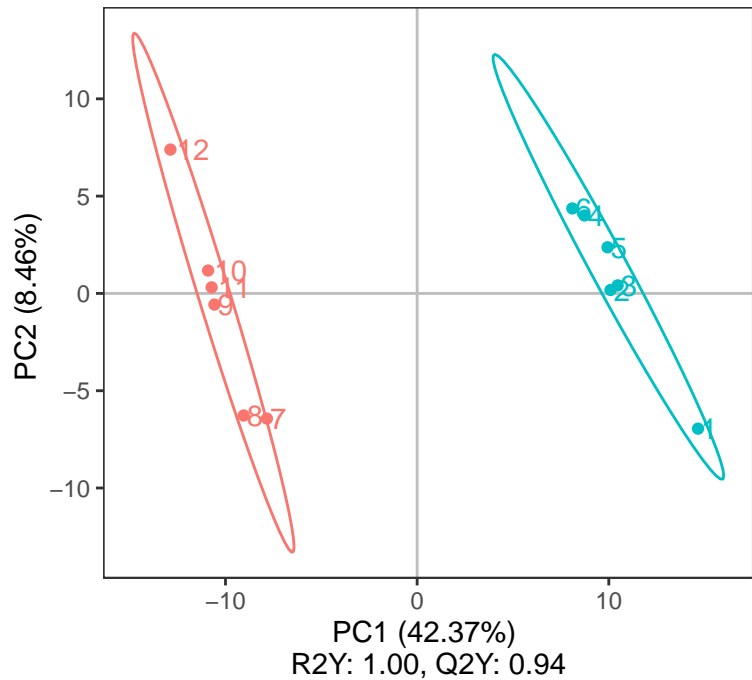

Supplement: Supplementary file 1 — Supplementary Information 1. [file 41598_2022_24687_MOESM1_ESM.zip › raw data/Metabolomics raw data/3.MetDiffScreening/EG.vs.CG/EG.vs.CG_neg_PLSDA-score.pdf]

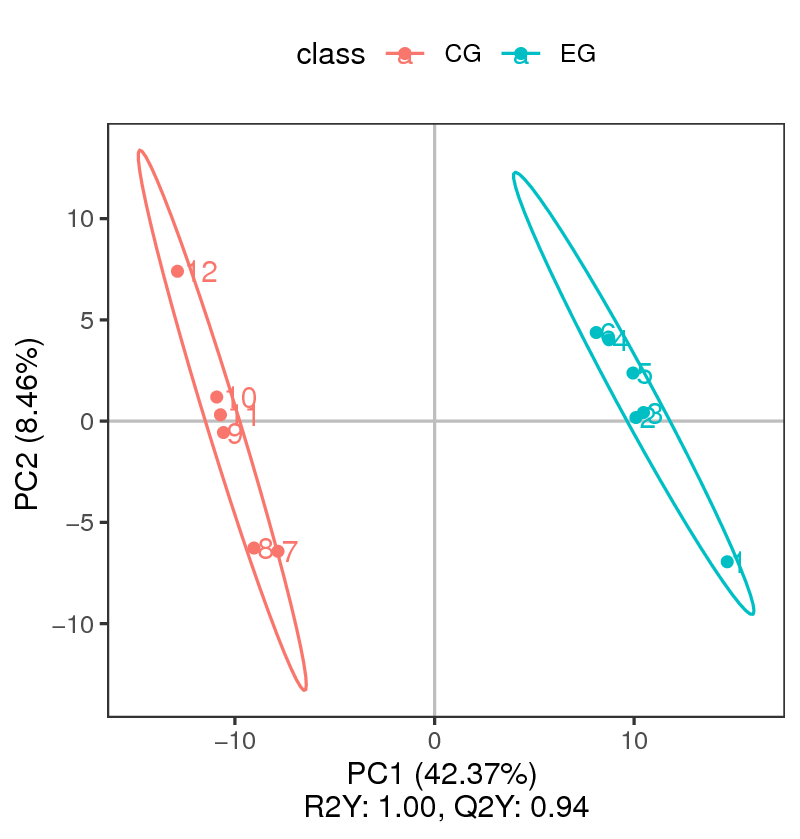

Supplement: Supplementary file 1 — Supplementary Information 1. [file 41598_2022_24687_MOESM1_ESM.zip › raw data/Metabolomics raw data/3.MetDiffScreening/EG.vs.CG/EG.vs.CG_neg_PLSDA-score.png]

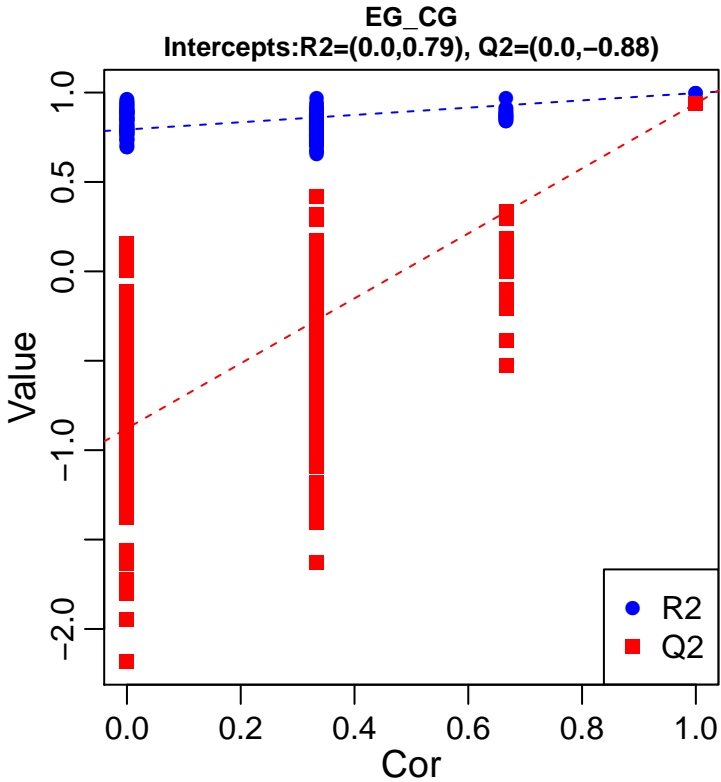

Supplement: Supplementary file 1 — Supplementary Information 1. [file 41598_2022_24687_MOESM1_ESM.zip › raw data/Metabolomics raw data/3.MetDiffScreening/EG.vs.CG/EG.vs.CG_neg_PLSDA-valid.pdf]

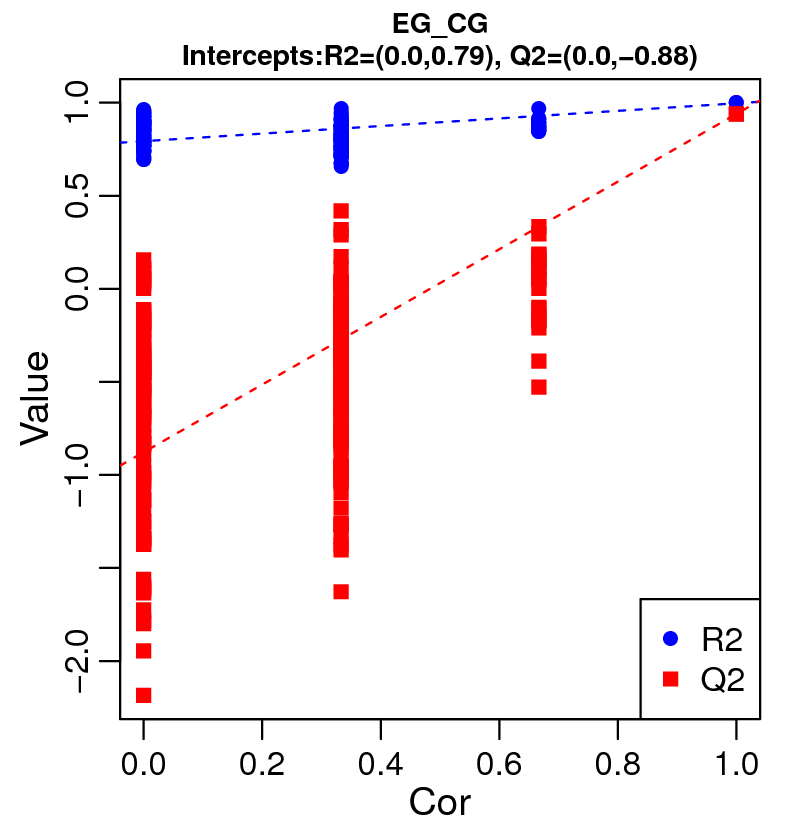

Supplement: Supplementary file 1 — Supplementary Information 1. [file 41598_2022_24687_MOESM1_ESM.zip › raw data/Metabolomics raw data/3.MetDiffScreening/EG.vs.CG/EG.vs.CG_neg_PLSDA-valid.png]

# EG.vs.CG

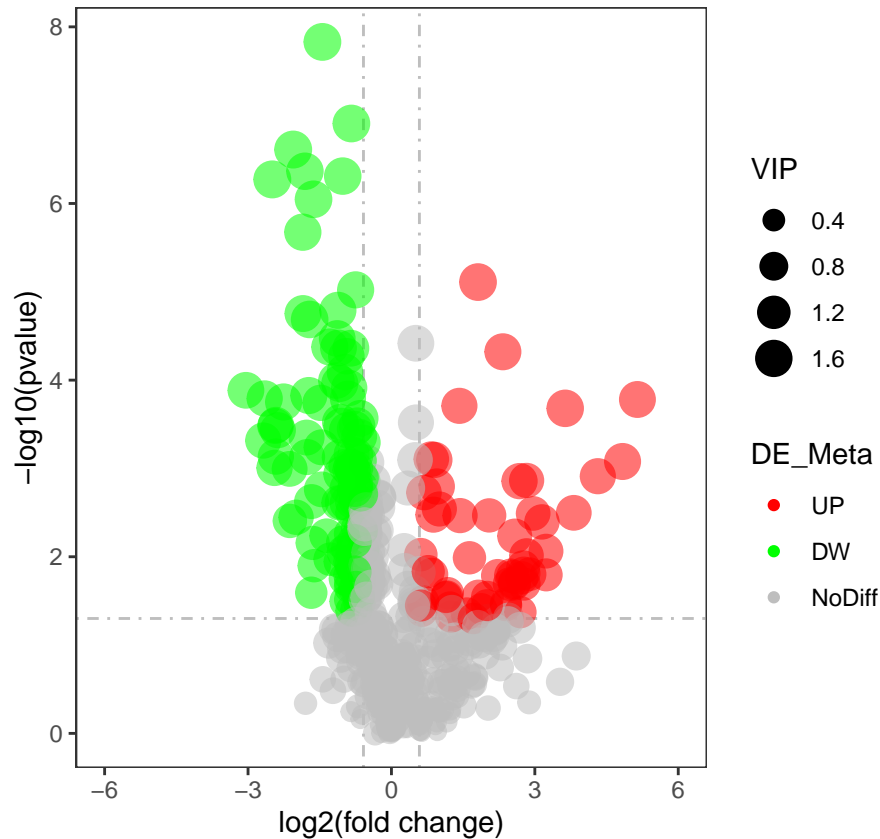

Supplement: Supplementary file 1 — Supplementary Information 1. [file 41598_2022_24687_MOESM1_ESM.zip › raw data/Metabolomics raw data/3.MetDiffScreening/EG.vs.CG/EG.vs.CG_pos.xls.volcano.pdf]

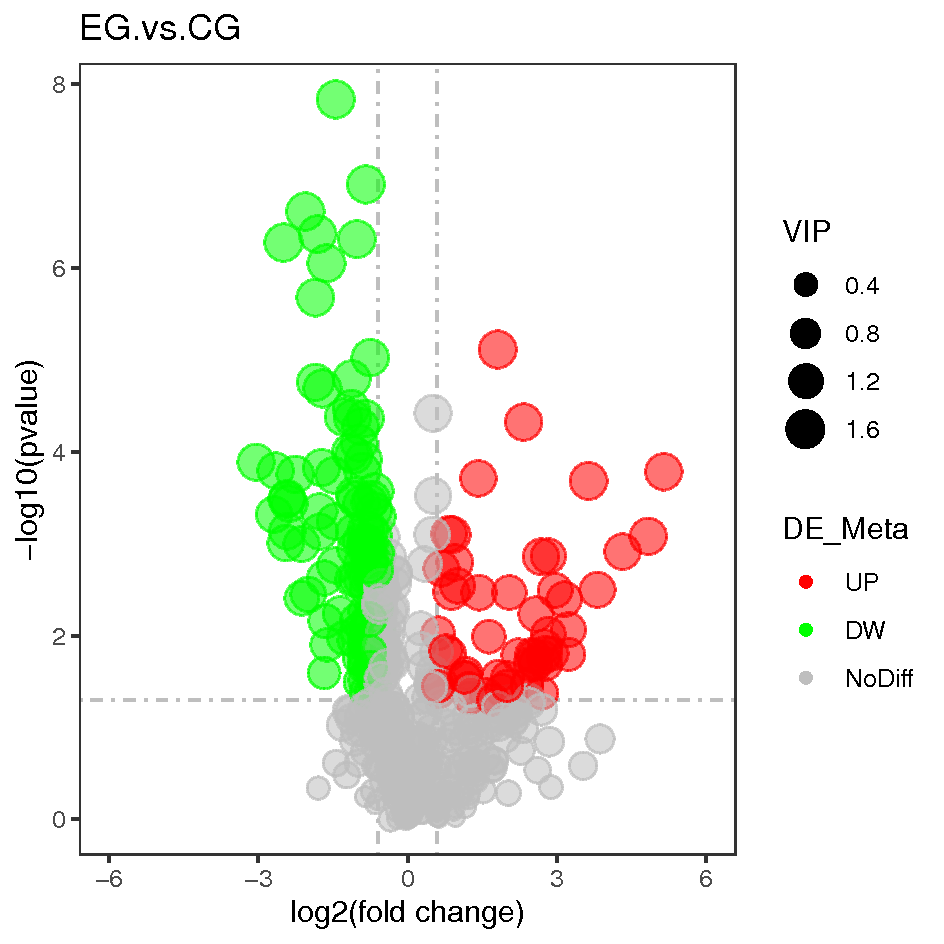

Supplement: Supplementary file 1 — Supplementary Information 1. [file 41598_2022_24687_MOESM1_ESM.zip › raw data/Metabolomics raw data/3.MetDiffScreening/EG.vs.CG/EG.vs.CG_pos.xls.volcano.png]

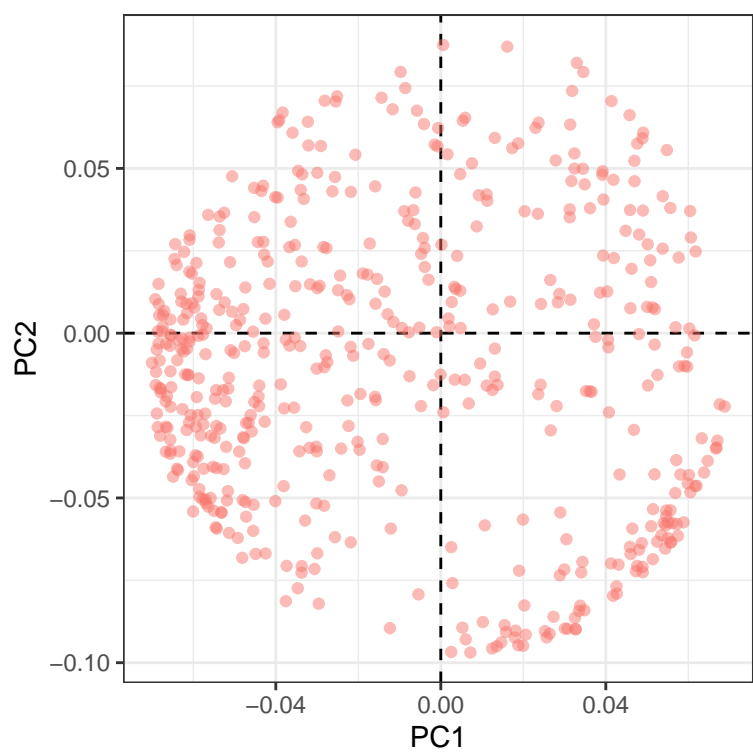

Supplement: Supplementary file 1 — Supplementary Information 1. [file 41598_2022_24687_MOESM1_ESM.zip › raw data/Metabolomics raw data/3.MetDiffScreening/EG.vs.CG/EG.vs.CG_pos_PCA-pcaloading.pdf]

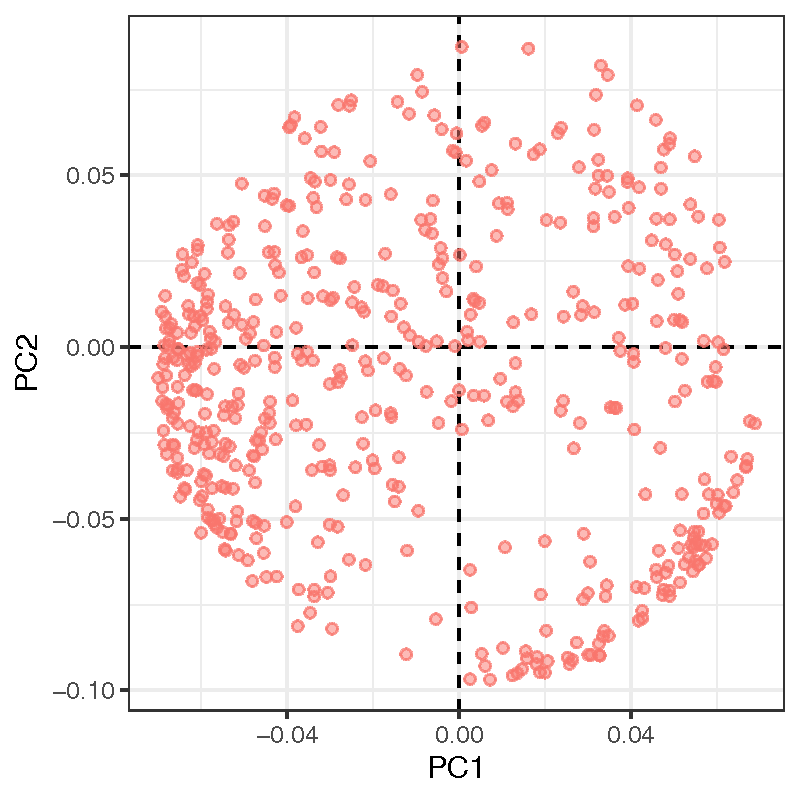

Supplement: Supplementary file 1 — Supplementary Information 1. [file 41598_2022_24687_MOESM1_ESM.zip › raw data/Metabolomics raw data/3.MetDiffScreening/EG.vs.CG/EG.vs.CG_pos_PCA-pcaloading.png]

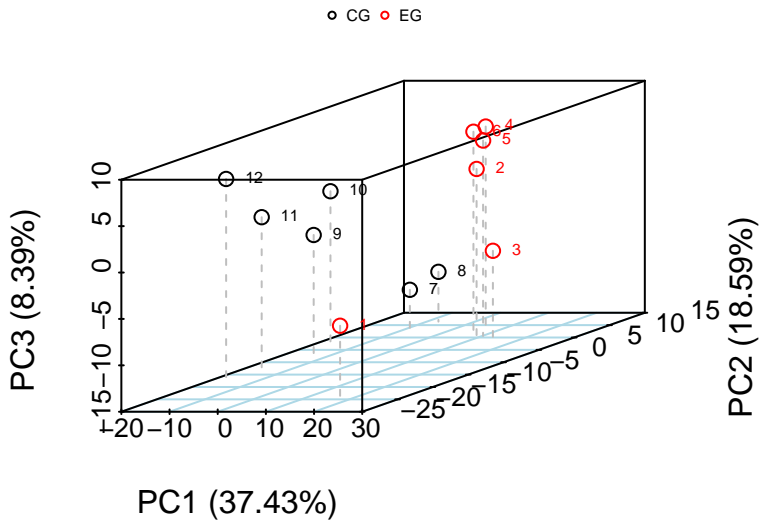

Supplement: Supplementary file 1 — Supplementary Information 1. [file 41598_2022_24687_MOESM1_ESM.zip › raw data/Metabolomics raw data/3.MetDiffScreening/EG.vs.CG/EG.vs.CG_pos_PCA.3D.pdf]

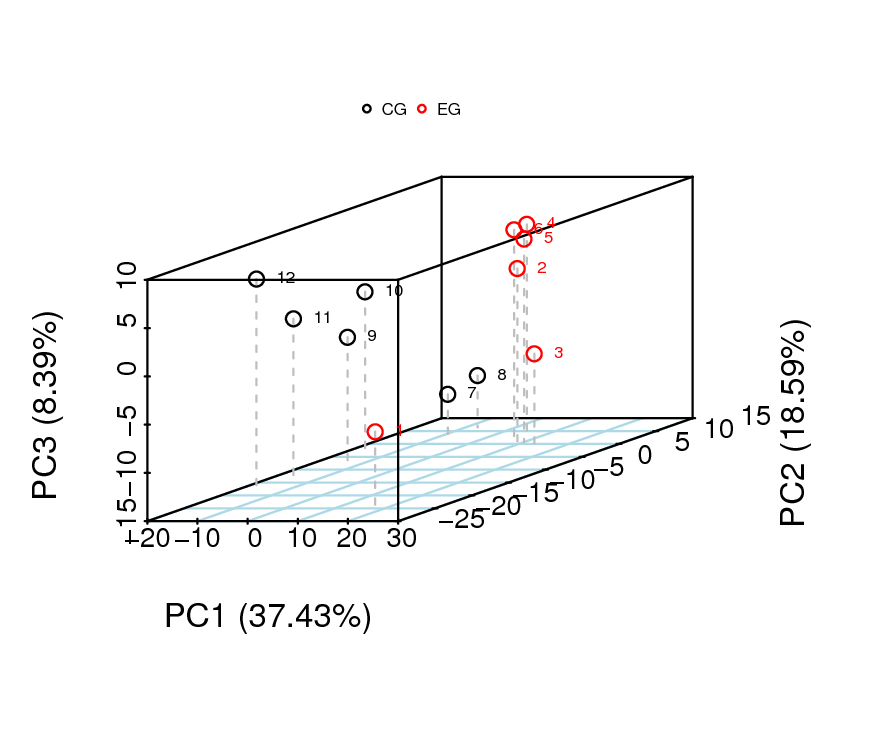

Supplement: Supplementary file 1 — Supplementary Information 1. [file 41598_2022_24687_MOESM1_ESM.zip › raw data/Metabolomics raw data/3.MetDiffScreening/EG.vs.CG/EG.vs.CG_pos_PCA.3D.png]

class    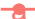 CG    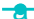 EG

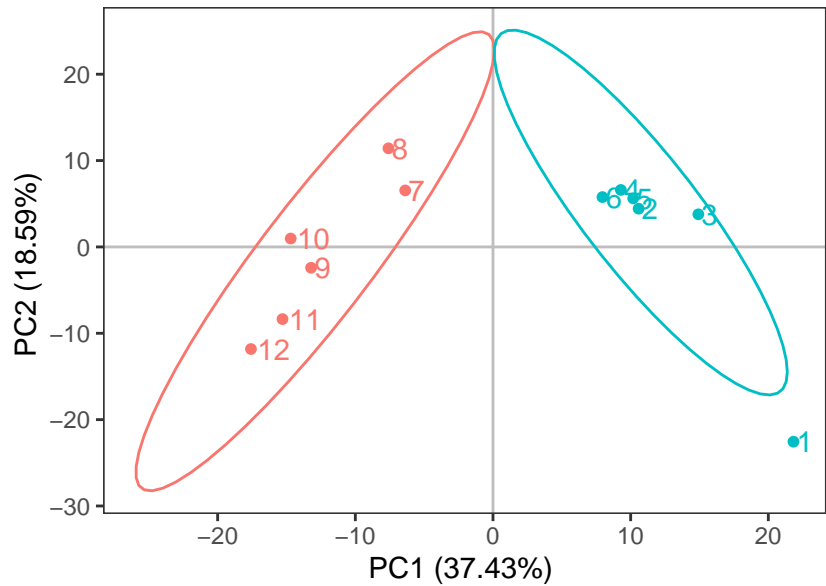

Supplement: Supplementary file 1 — Supplementary Information 1. [file 41598_2022_24687_MOESM1_ESM.zip › raw data/Metabolomics raw data/3.MetDiffScreening/EG.vs.CG/EG.vs.CG_pos_PCA.pdf]

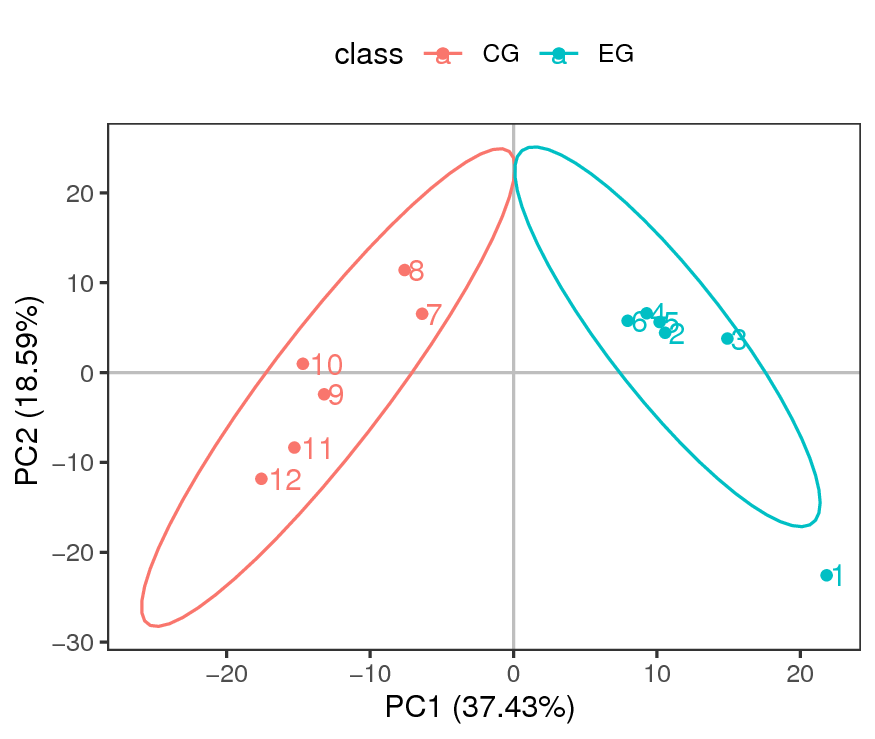

Supplement: Supplementary file 1 — Supplementary Information 1. [file 41598_2022_24687_MOESM1_ESM.zip › raw data/Metabolomics raw data/3.MetDiffScreening/EG.vs.CG/EG.vs.CG_pos_PCA.png]

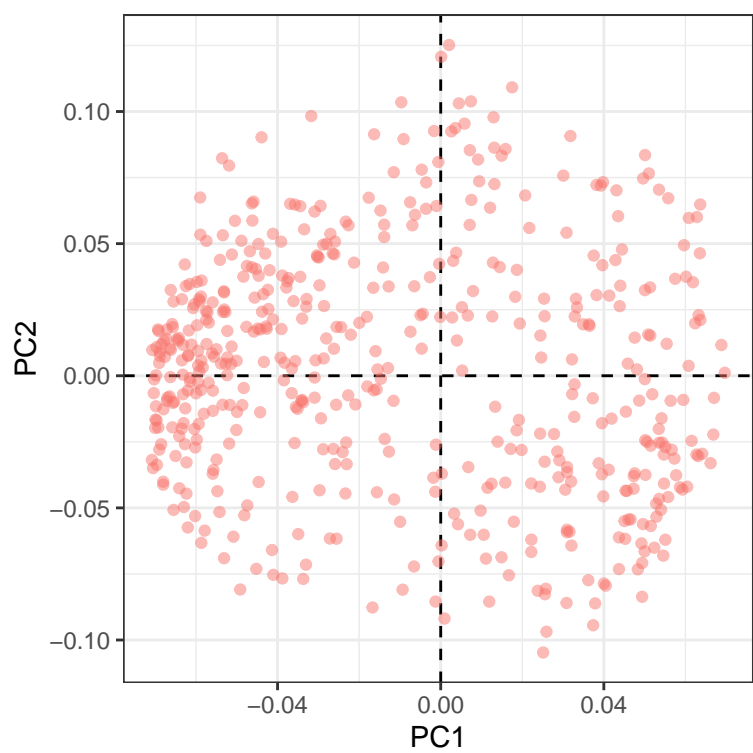

Supplement: Supplementary file 1 — Supplementary Information 1. [file 41598_2022_24687_MOESM1_ESM.zip › raw data/Metabolomics raw data/3.MetDiffScreening/EG.vs.CG/EG.vs.CG_pos_PLSDA-loading.pdf]

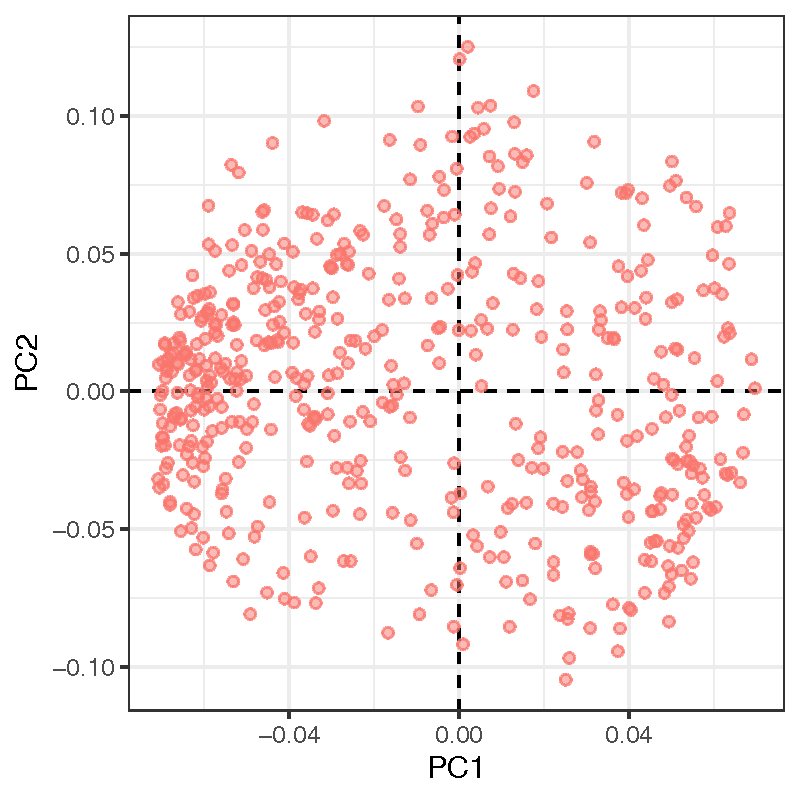

Supplement: Supplementary file 1 — Supplementary Information 1. [file 41598_2022_24687_MOESM1_ESM.zip › raw data/Metabolomics raw data/3.MetDiffScreening/EG.vs.CG/EG.vs.CG_pos_PLSDA-loading.png]

class   CG   EG

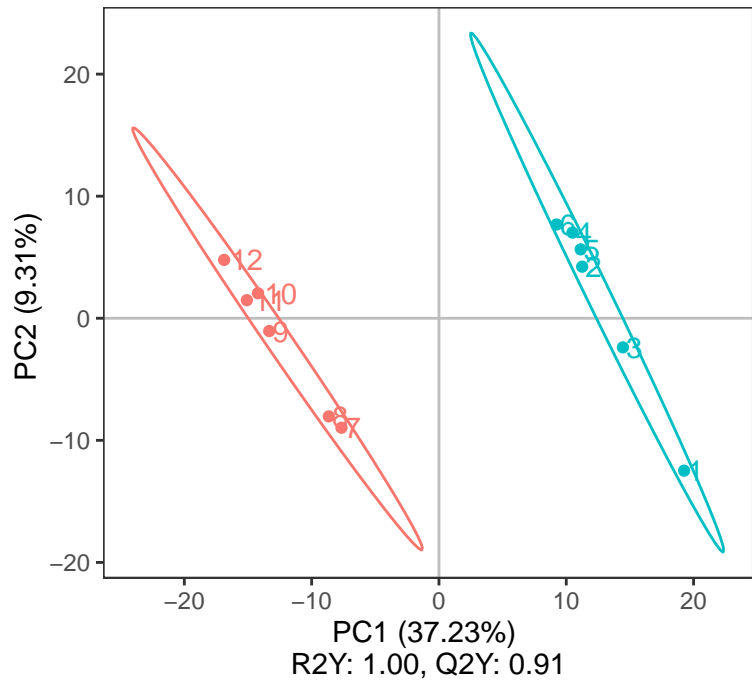

Supplement: Supplementary file 1 — Supplementary Information 1. [file 41598_2022_24687_MOESM1_ESM.zip › raw data/Metabolomics raw data/3.MetDiffScreening/EG.vs.CG/EG.vs.CG_pos_PLSDA-score.pdf]

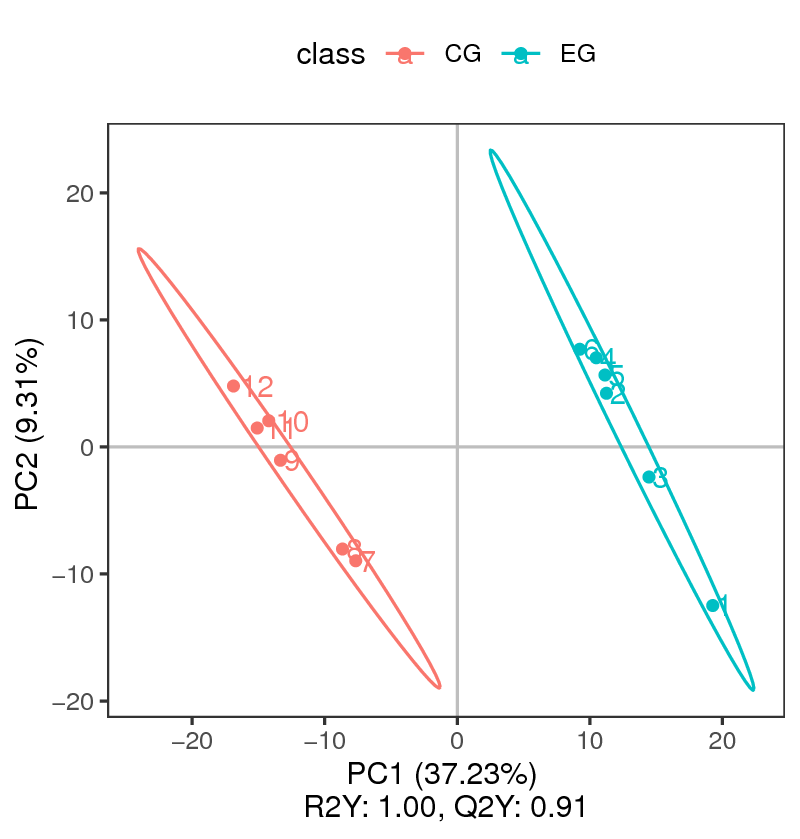

Supplement: Supplementary file 1 — Supplementary Information 1. [file 41598_2022_24687_MOESM1_ESM.zip › raw data/Metabolomics raw data/3.MetDiffScreening/EG.vs.CG/EG.vs.CG_pos_PLSDA-score.png]

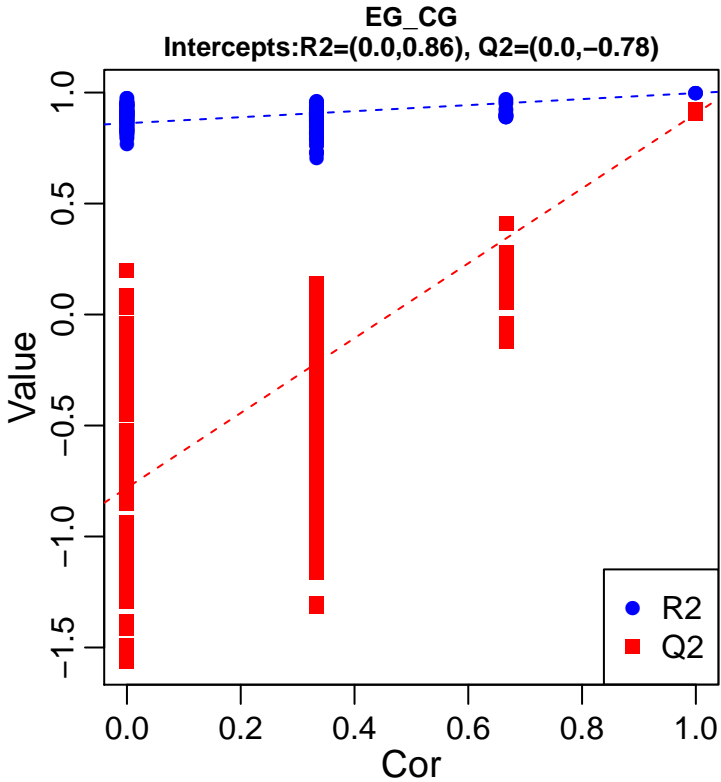

Supplement: Supplementary file 1 — Supplementary Information 1. [file 41598_2022_24687_MOESM1_ESM.zip › raw data/Metabolomics raw data/3.MetDiffScreening/EG.vs.CG/EG.vs.CG_pos_PLSDA-valid.pdf]

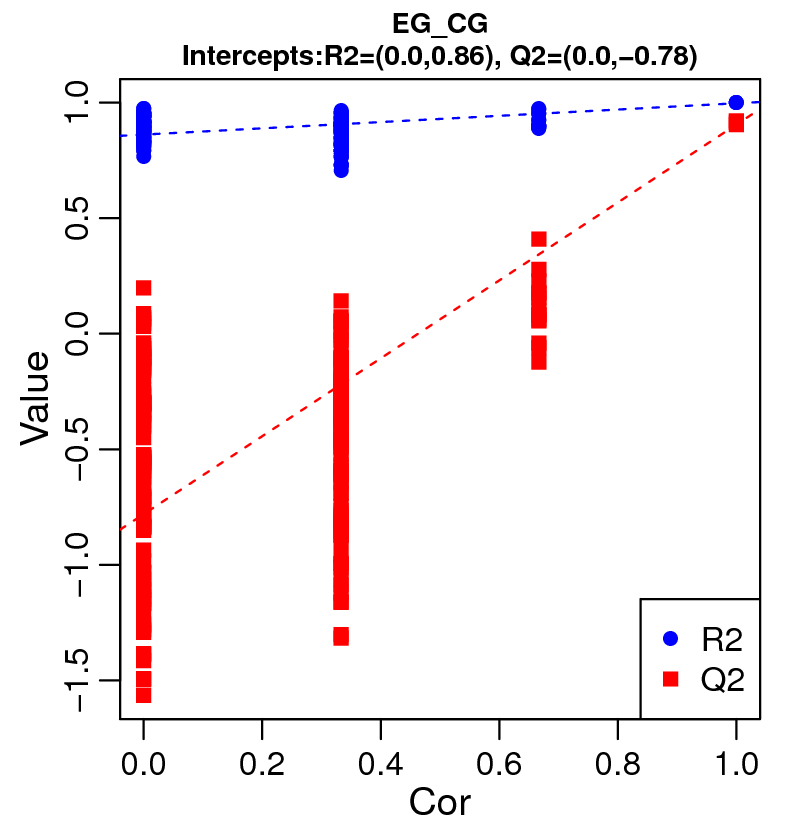

Supplement: Supplementary file 1 — Supplementary Information 1. [file 41598_2022_24687_MOESM1_ESM.zip › raw data/Metabolomics raw data/3.MetDiffScreening/EG.vs.CG/EG.vs.CG_pos_PLSDA-valid.png]

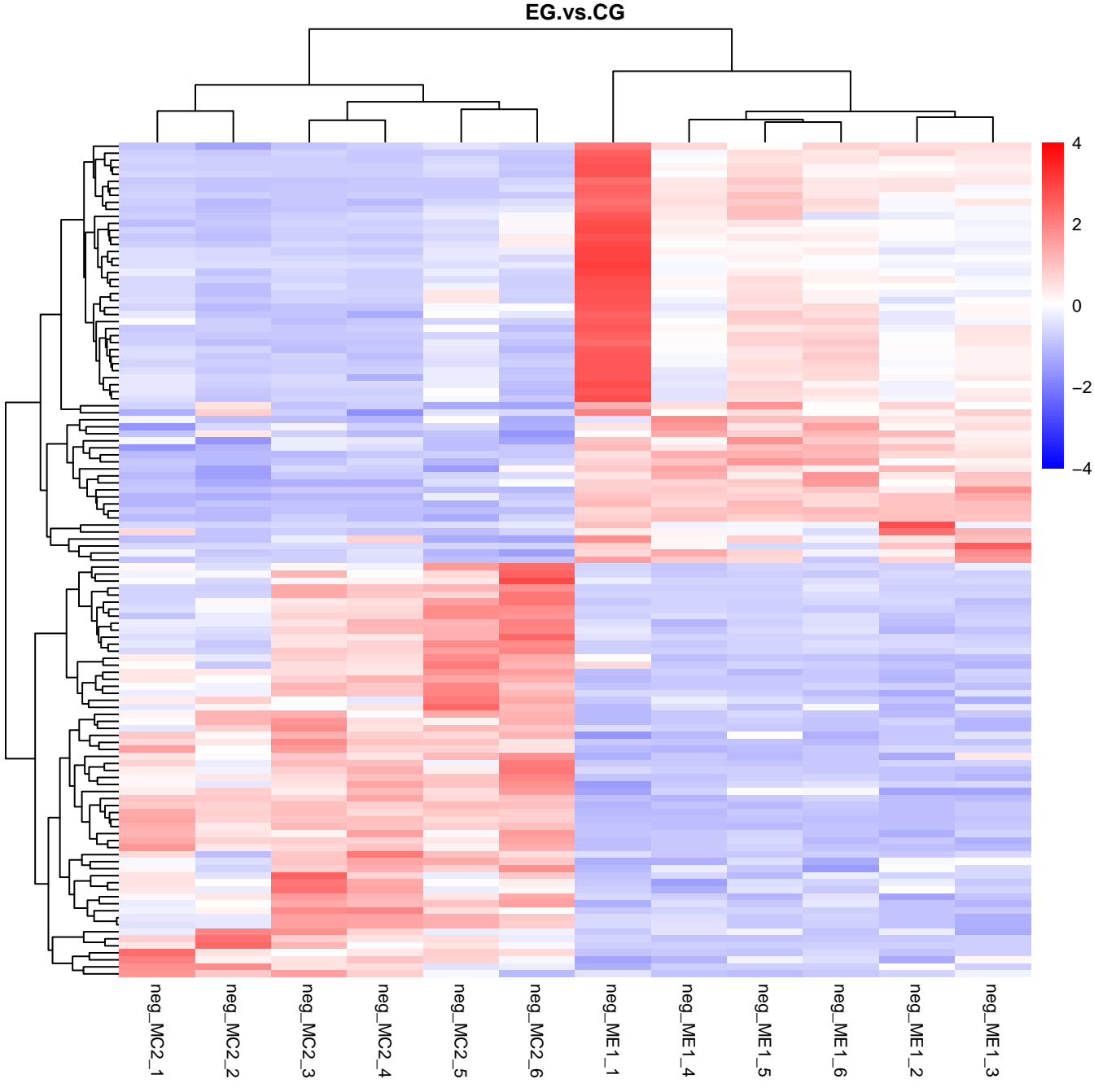

Supplement: Supplementary file 1 — Supplementary Information 1. [file 41598_2022_24687_MOESM1_ESM.zip › raw data/Metabolomics raw data/4.MetDiffAnalysis/EG.vs.CG/EG.vs.CG_neg_cluster_heatmap.pdf]

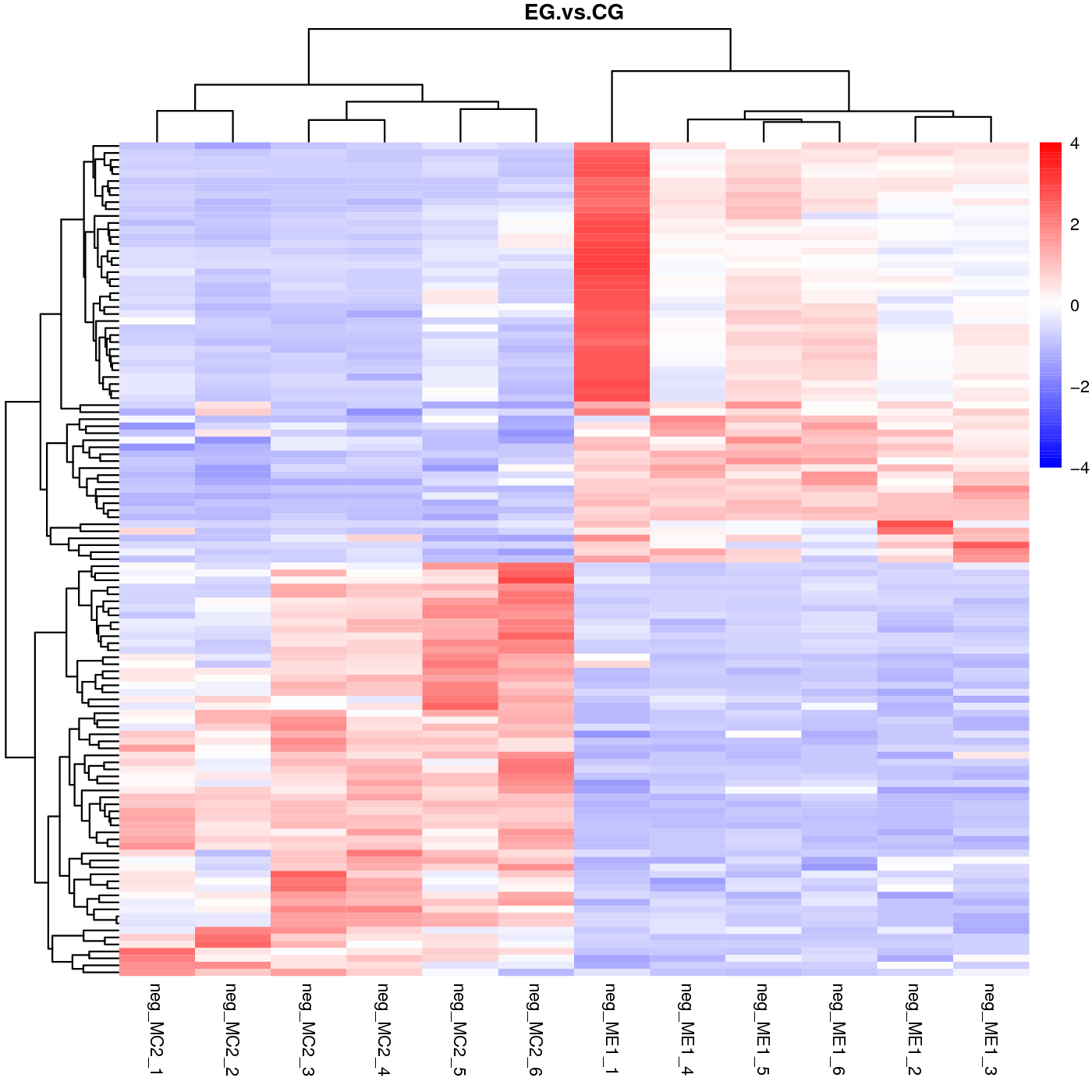

Supplement: Supplementary file 1 — Supplementary Information 1. [file 41598_2022_24687_MOESM1_ESM.zip › raw data/Metabolomics raw data/4.MetDiffAnalysis/EG.vs.CG/EG.vs.CG_neg_cluster_heatmap.png]

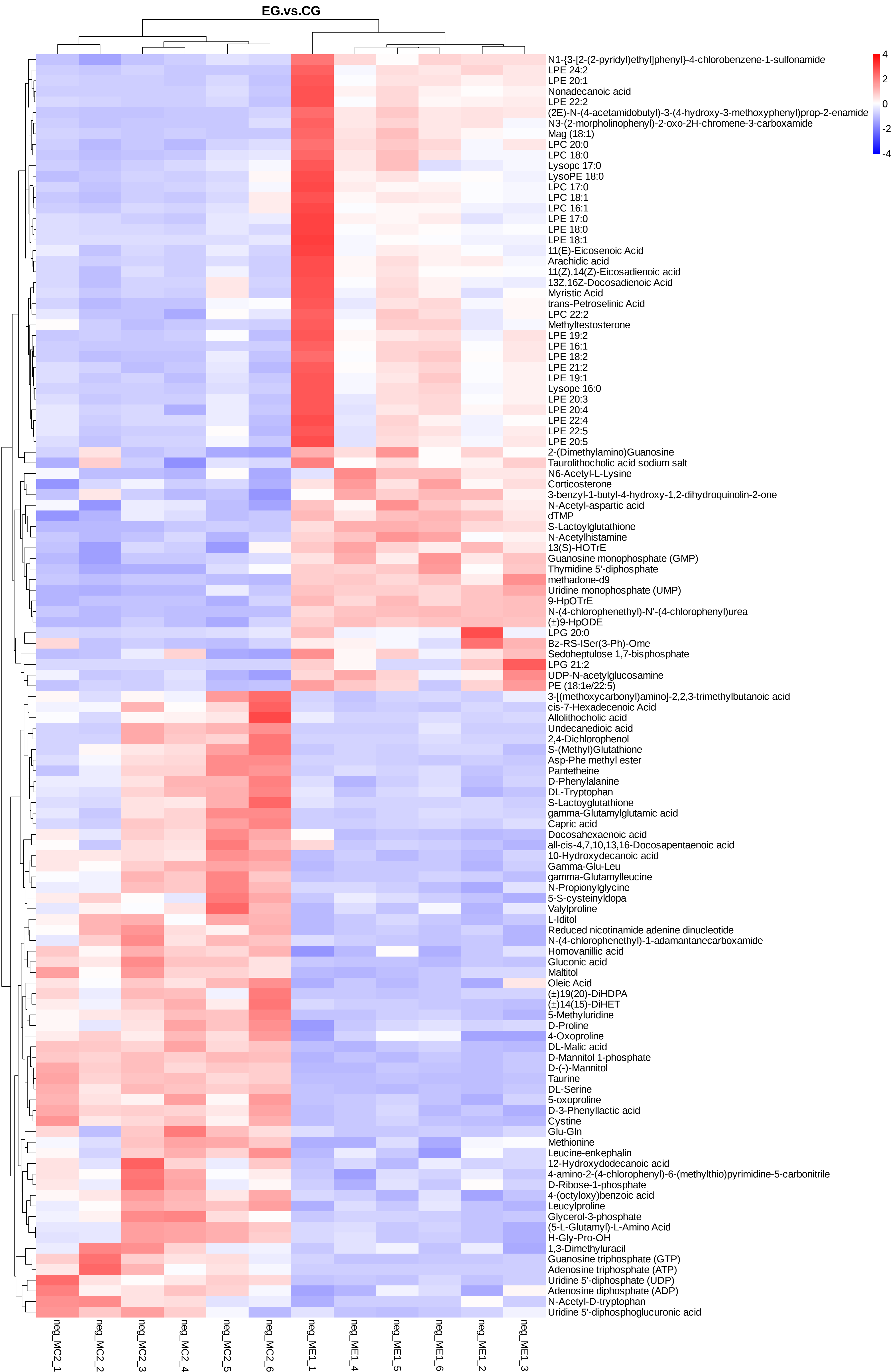

Supplement: Supplementary file 1 — Supplementary Information 1. [file 41598_2022_24687_MOESM1_ESM.zip › raw data/Metabolomics raw data/4.MetDiffAnalysis/EG.vs.CG/EG.vs.CG_neg_cluster_heatmap_detail.pdf]

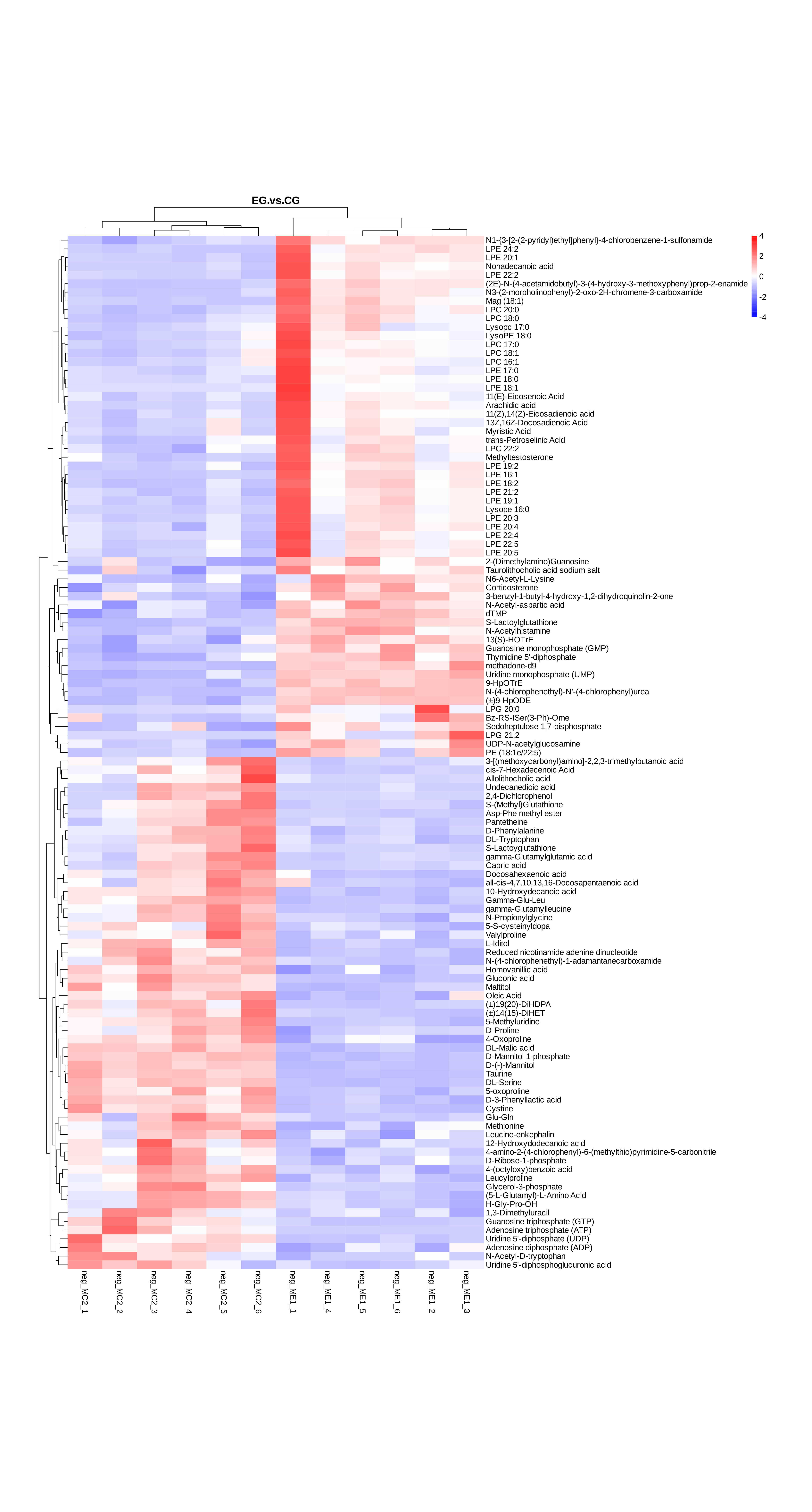

Supplement: Supplementary file 1 — Supplementary Information 1. [file 41598_2022_24687_MOESM1_ESM.zip › raw data/Metabolomics raw data/4.MetDiffAnalysis/EG.vs.CG/EG.vs.CG_neg_cluster_heatmap_detail.png]

EG.vs.CG

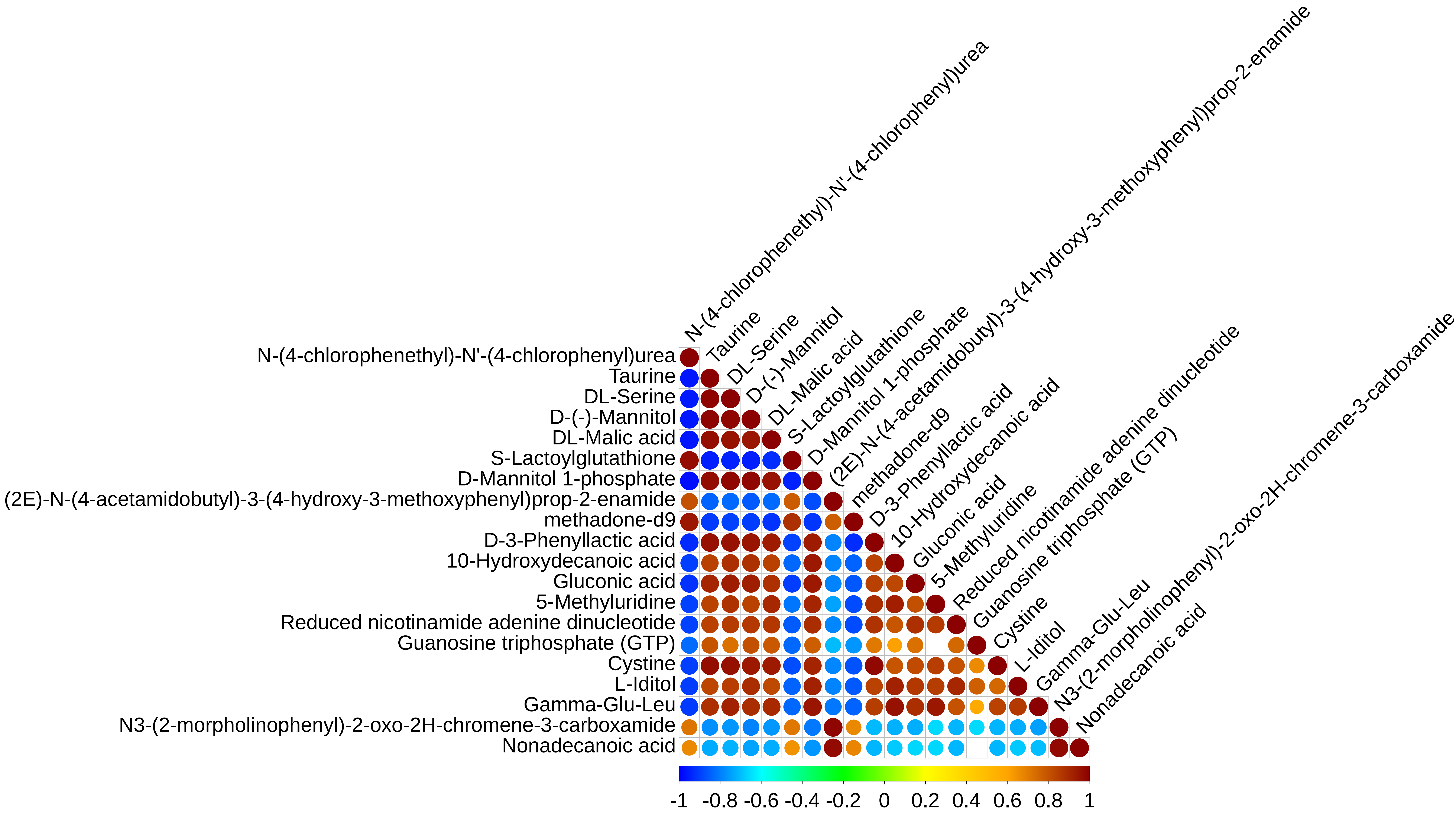

Supplement: Supplementary file 1 — Supplementary Information 1. [file 41598_2022_24687_MOESM1_ESM.zip › raw data/Metabolomics raw data/4.MetDiffAnalysis/EG.vs.CG/EG.vs.CG_neg_corr.pdf]

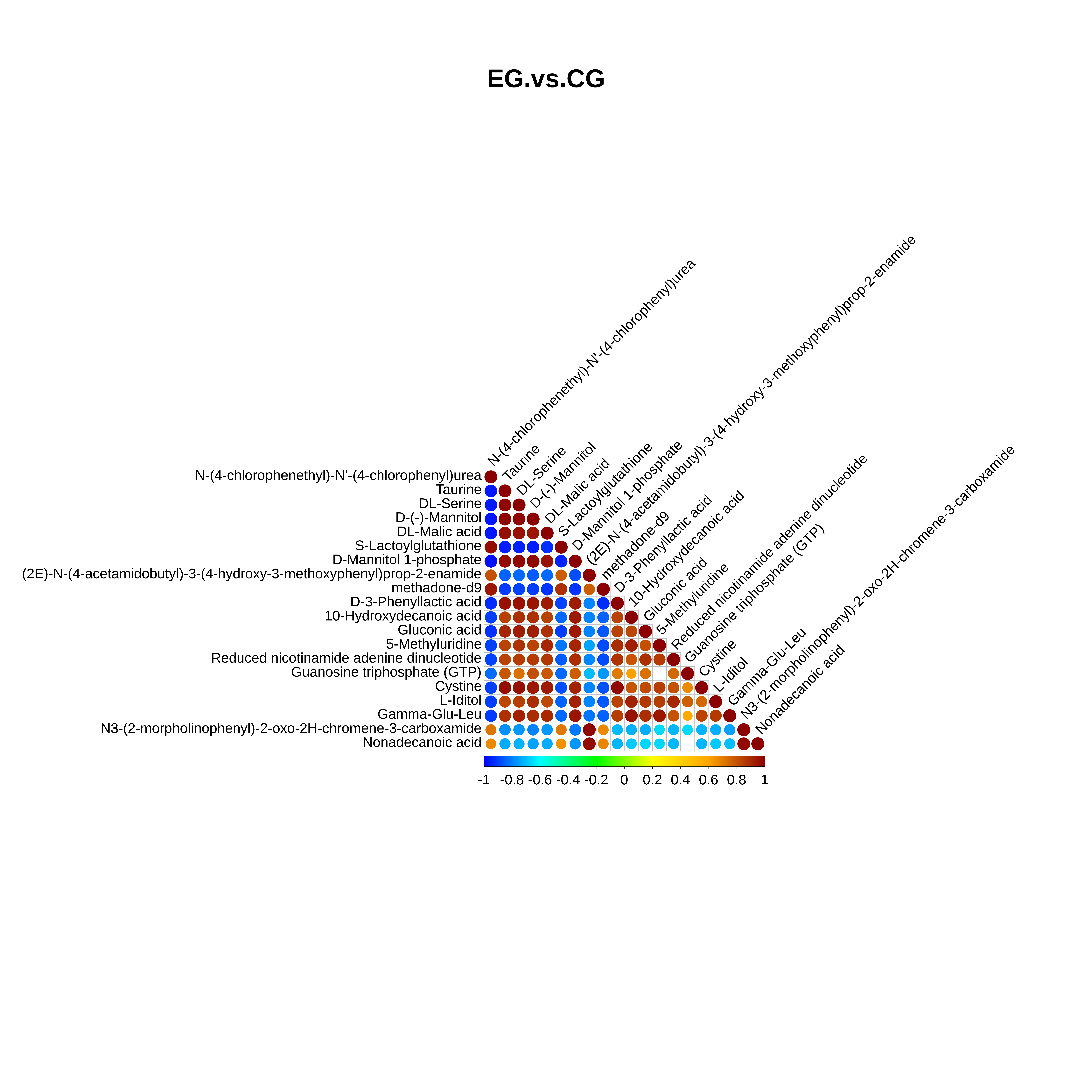

Supplement: Supplementary file 1 — Supplementary Information 1. [file 41598_2022_24687_MOESM1_ESM.zip › raw data/Metabolomics raw data/4.MetDiffAnalysis/EG.vs.CG/EG.vs.CG_neg_corr.png]

EG.vs.CG

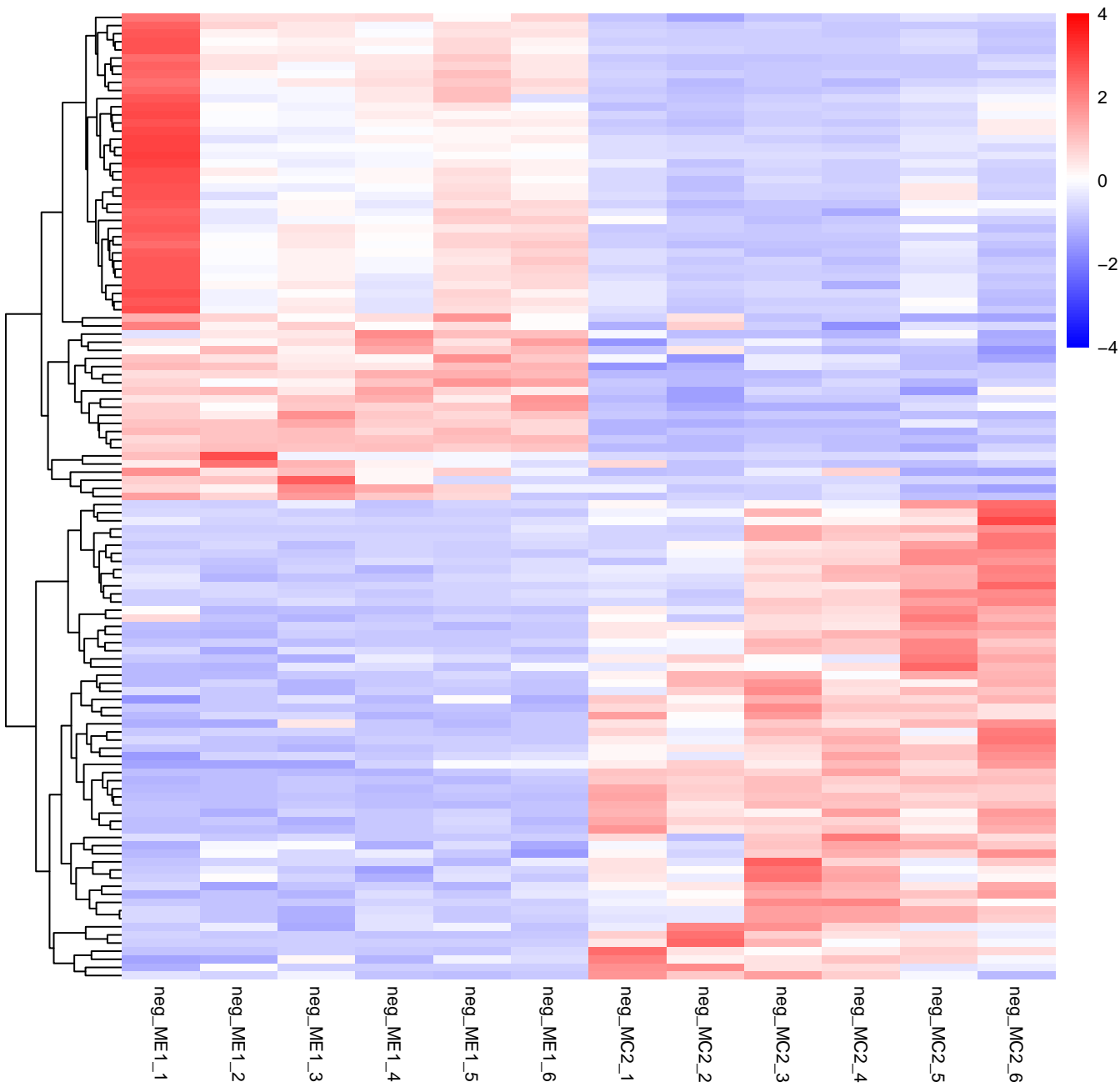

Supplement: Supplementary file 1 — Supplementary Information 1. [file 41598_2022_24687_MOESM1_ESM.zip › raw data/Metabolomics raw data/4.MetDiffAnalysis/EG.vs.CG/EG.vs.CG_neg_heatmap.pdf]

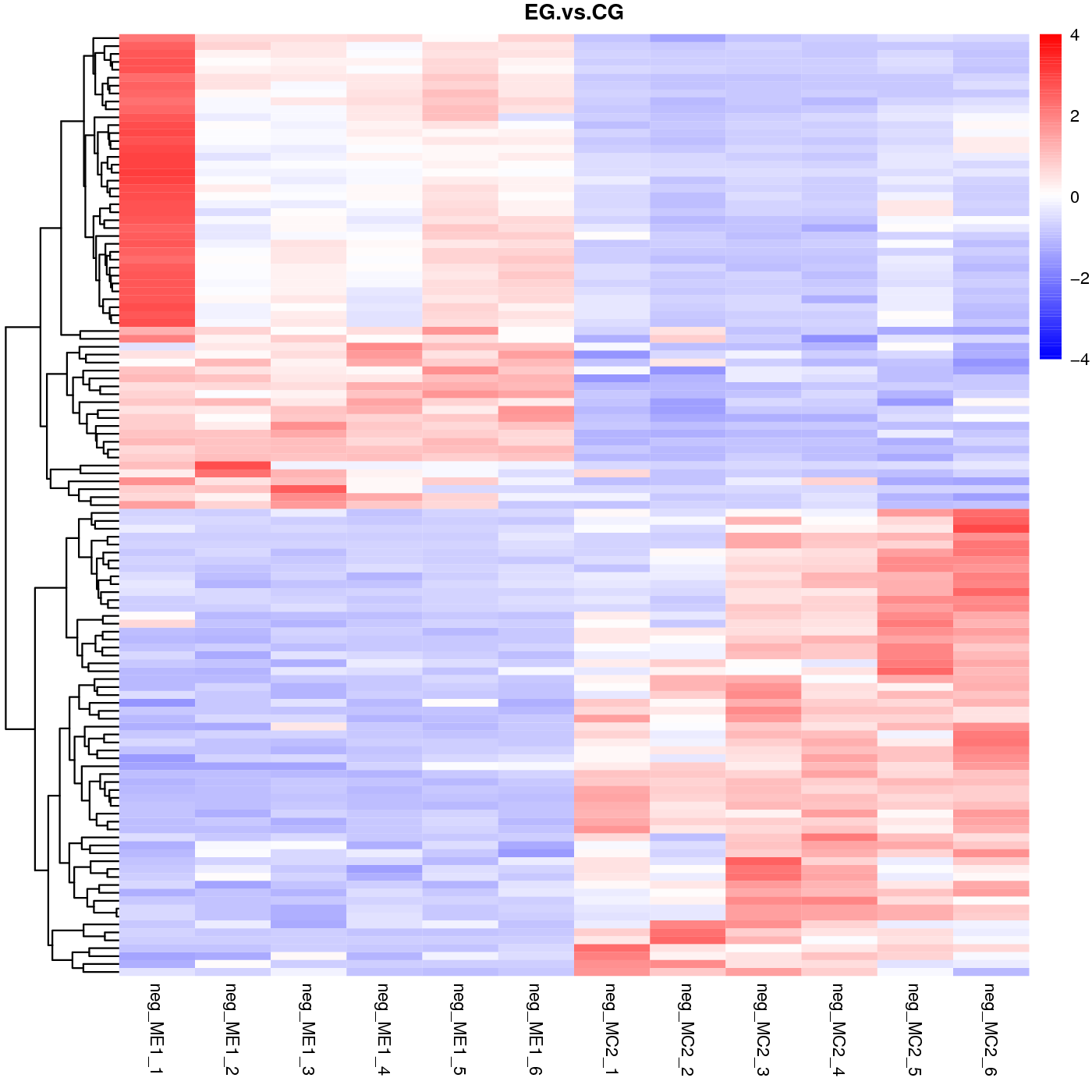

Supplement: Supplementary file 1 — Supplementary Information 1. [file 41598_2022_24687_MOESM1_ESM.zip › raw data/Metabolomics raw data/4.MetDiffAnalysis/EG.vs.CG/EG.vs.CG_neg_heatmap.png]

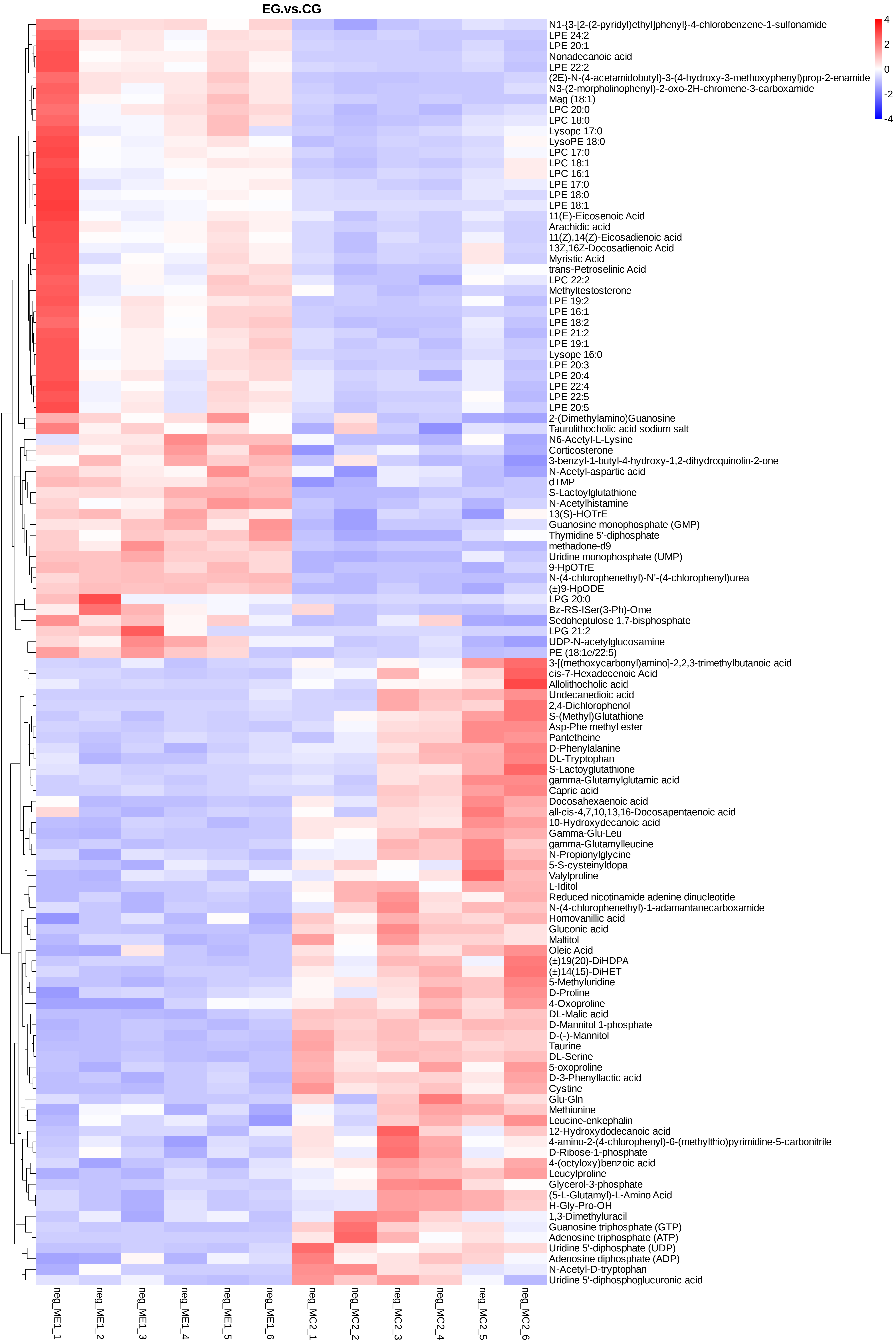

Supplement: Supplementary file 1 — Supplementary Information 1. [file 41598_2022_24687_MOESM1_ESM.zip › raw data/Metabolomics raw data/4.MetDiffAnalysis/EG.vs.CG/EG.vs.CG_neg_heatmap_detail.pdf]

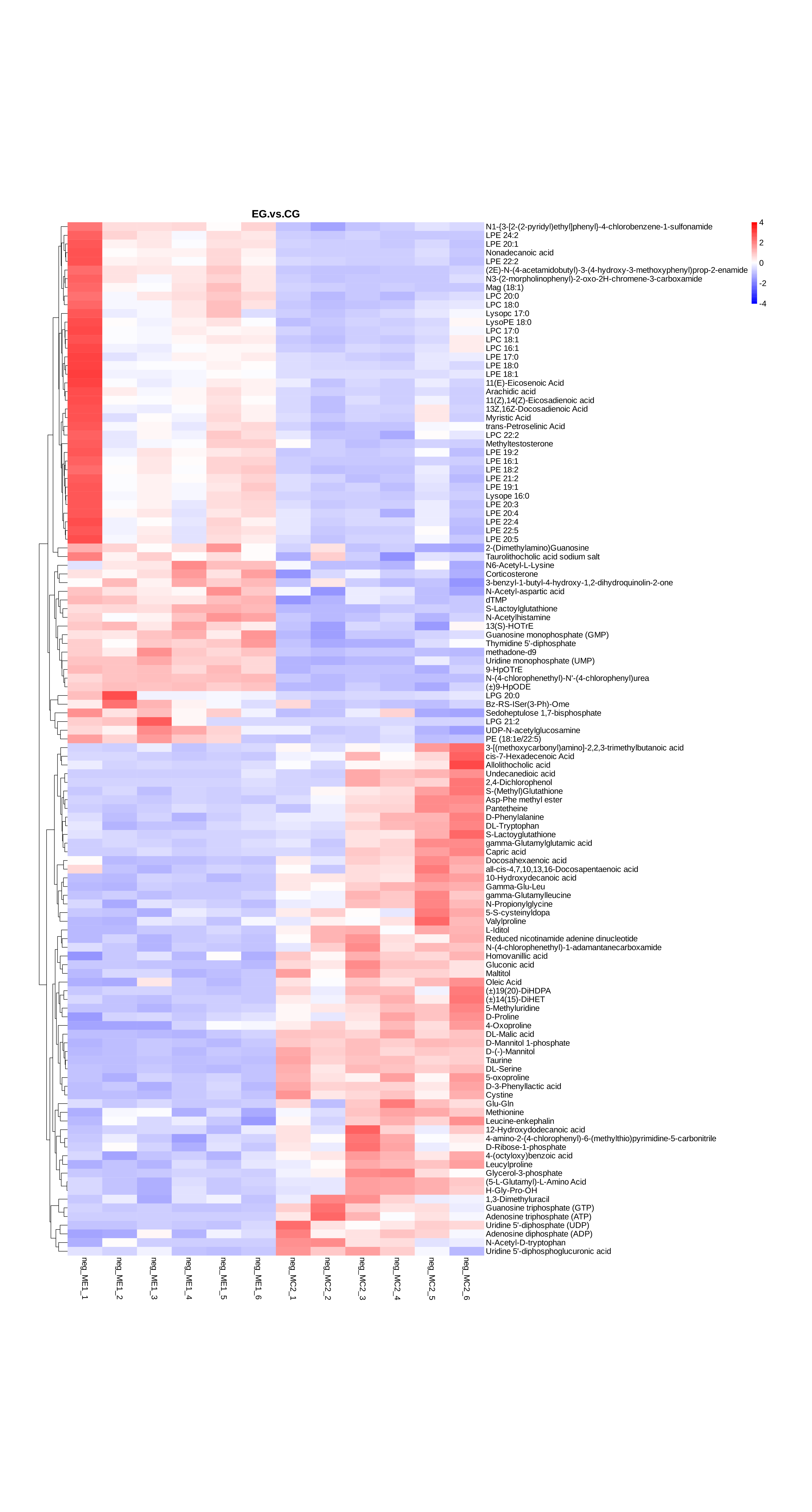

Supplement: Supplementary file 1 — Supplementary Information 1. [file 41598_2022_24687_MOESM1_ESM.zip › raw data/Metabolomics raw data/4.MetDiffAnalysis/EG.vs.CG/EG.vs.CG_neg_heatmap_detail.png]

## EG.vs.CG

Metabolites

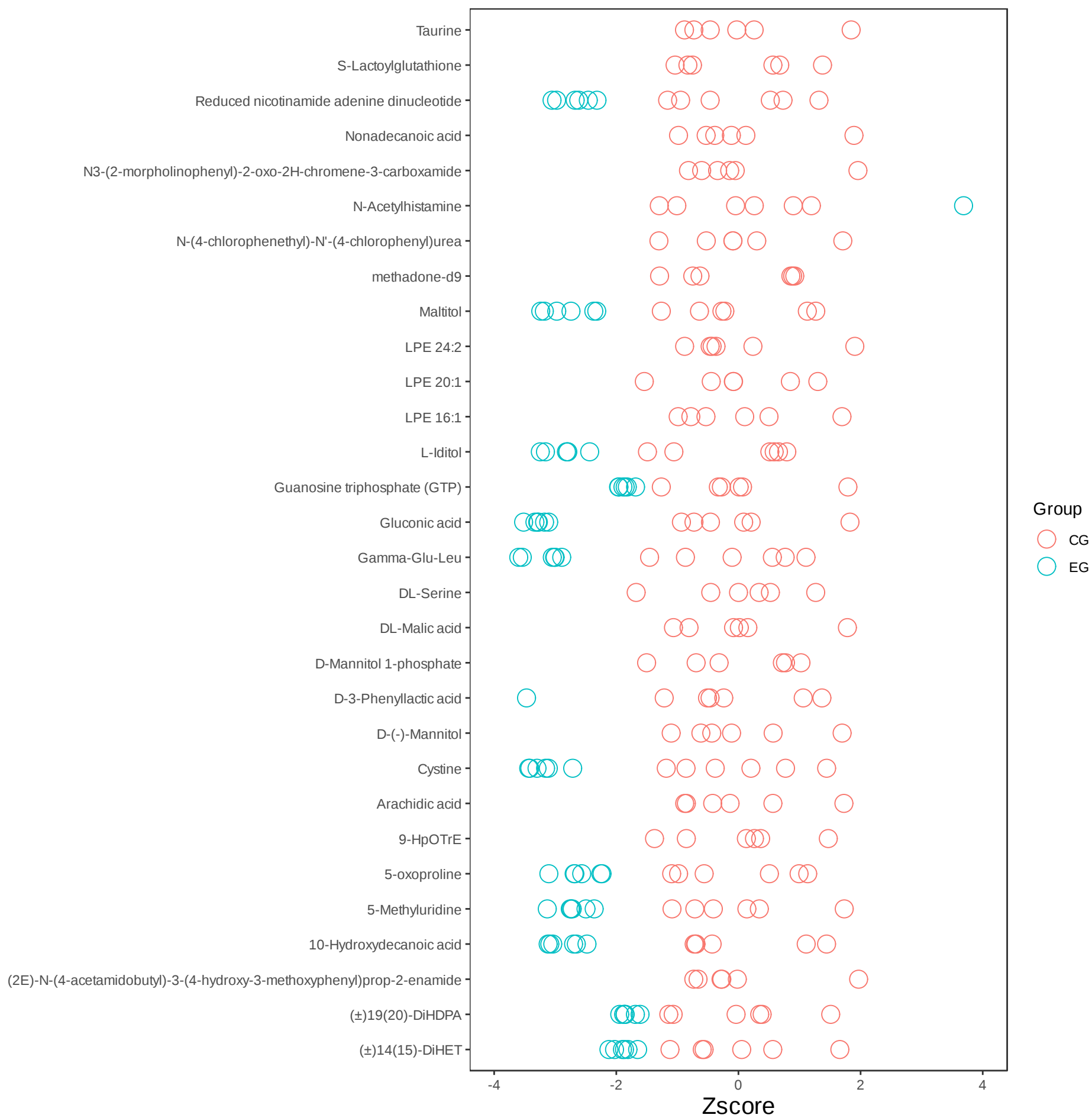

Supplement: Supplementary file 1 — Supplementary Information 1. [file 41598_2022_24687_MOESM1_ESM.zip › raw data/Metabolomics raw data/4.MetDiffAnalysis/EG.vs.CG/EG.vs.CG_neg_zscore.pdf]

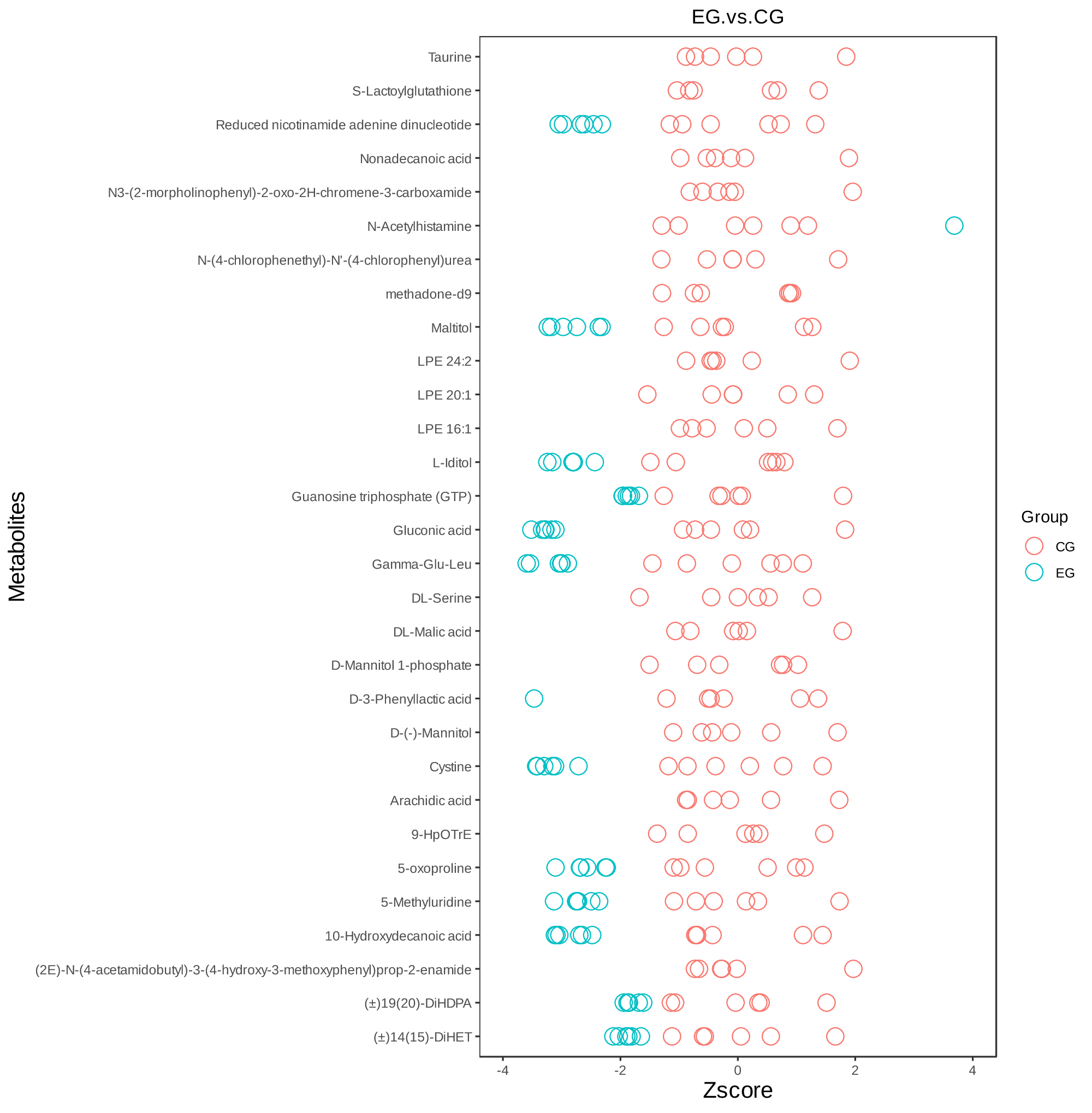

Supplement: Supplementary file 1 — Supplementary Information 1. [file 41598_2022_24687_MOESM1_ESM.zip › raw data/Metabolomics raw data/4.MetDiffAnalysis/EG.vs.CG/EG.vs.CG_neg_zscore.png]

EG.vs.CG

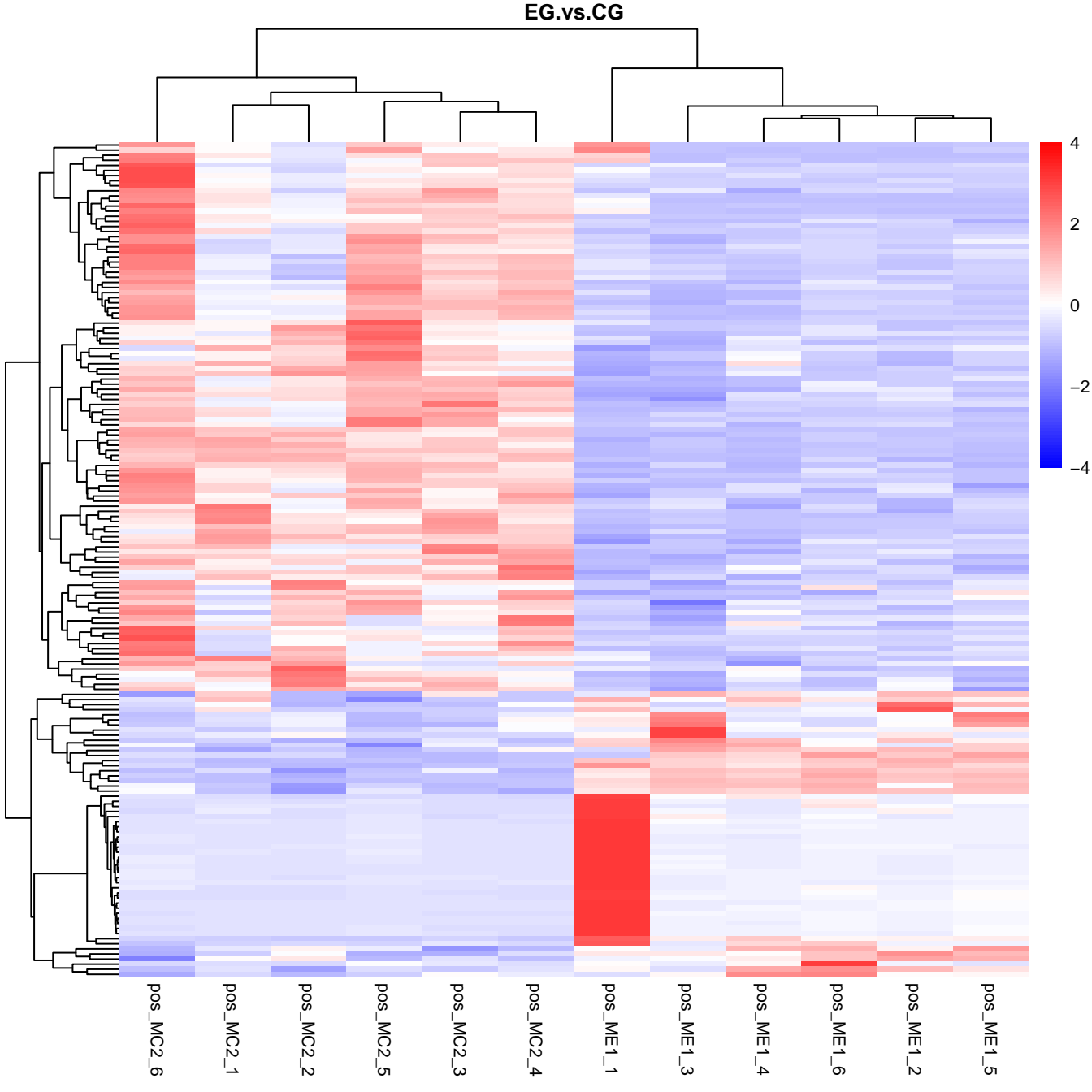

Supplement: Supplementary file 1 — Supplementary Information 1. [file 41598_2022_24687_MOESM1_ESM.zip › raw data/Metabolomics raw data/4.MetDiffAnalysis/EG.vs.CG/EG.vs.CG_pos_cluster_heatmap.pdf]

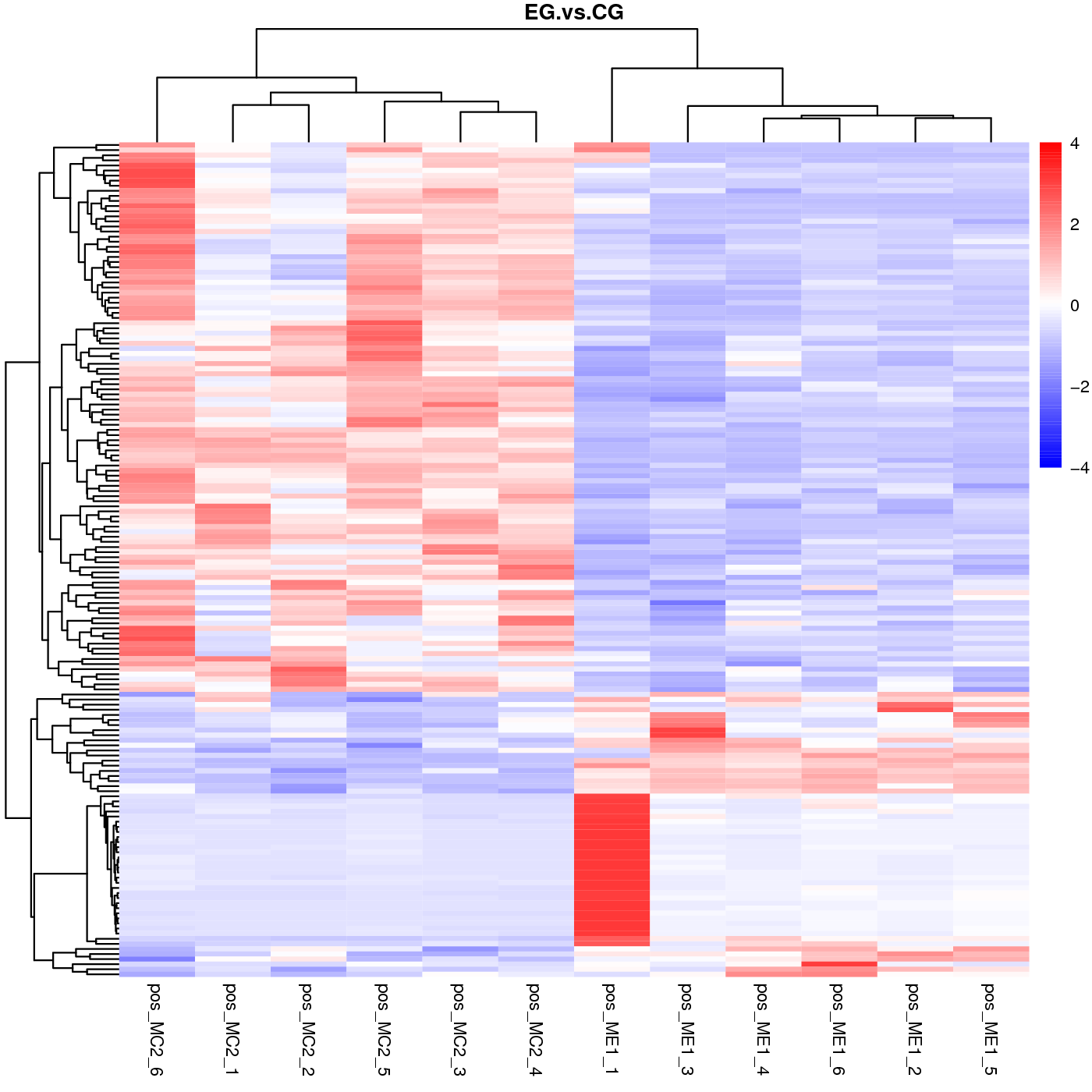

Supplement: Supplementary file 1 — Supplementary Information 1. [file 41598_2022_24687_MOESM1_ESM.zip › raw data/Metabolomics raw data/4.MetDiffAnalysis/EG.vs.CG/EG.vs.CG_pos_cluster_heatmap.png]

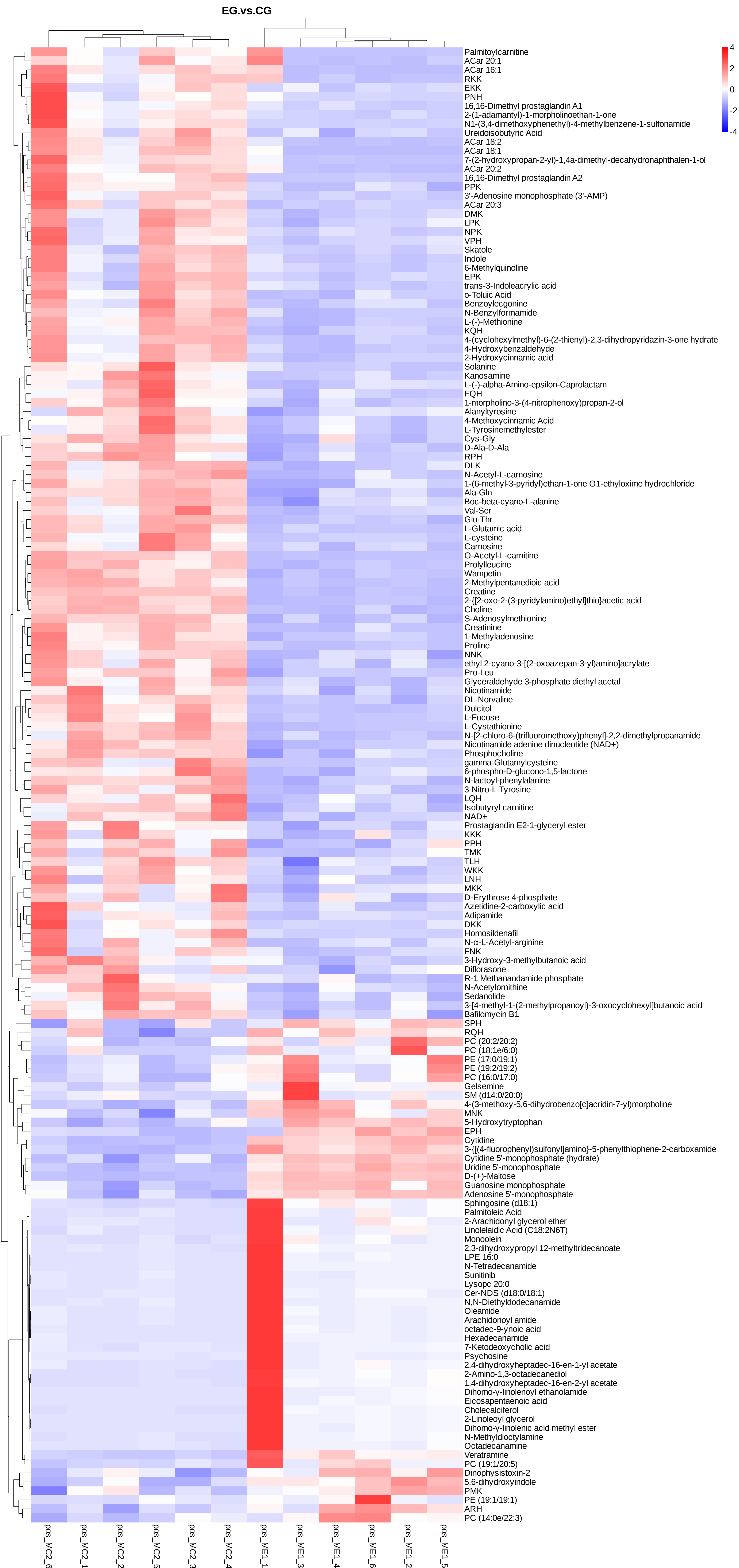

Supplement: Supplementary file 1 — Supplementary Information 1. [file 41598_2022_24687_MOESM1_ESM.zip › raw data/Metabolomics raw data/4.MetDiffAnalysis/EG.vs.CG/EG.vs.CG_pos_cluster_heatmap_detail.pdf]

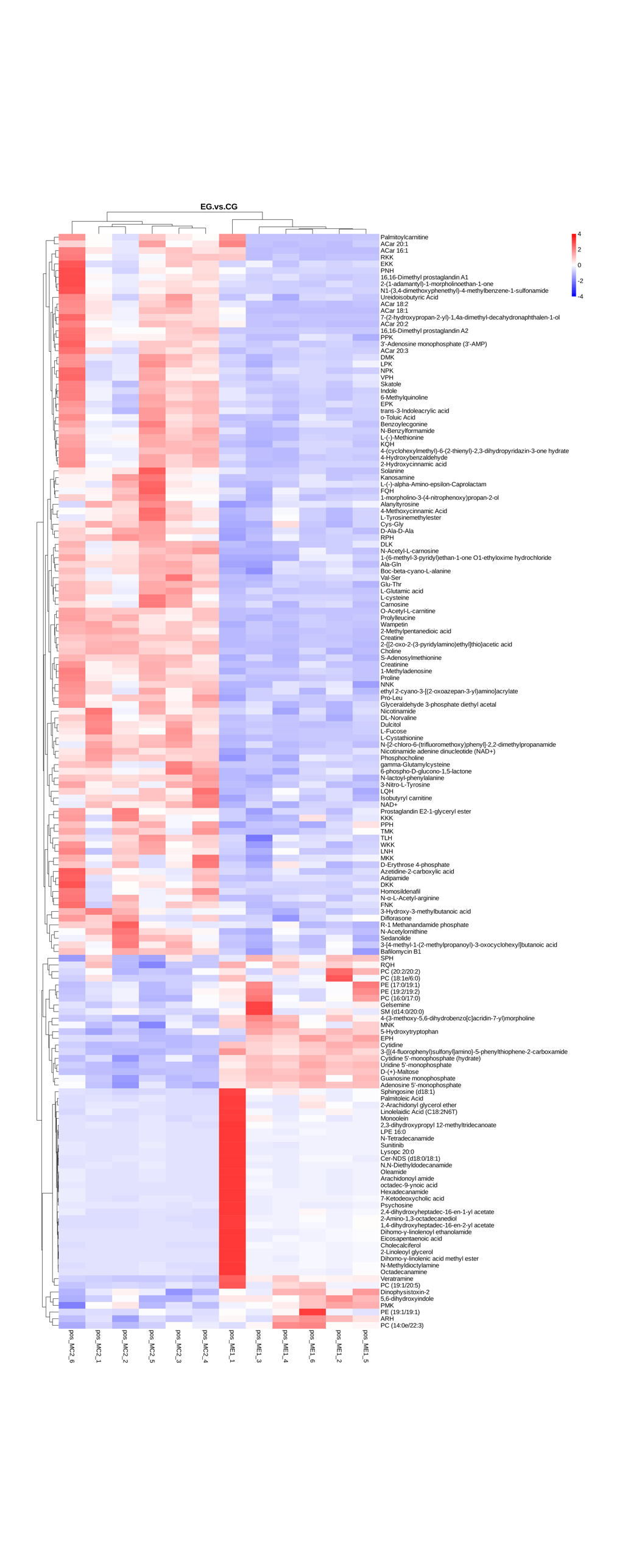

Supplement: Supplementary file 1 — Supplementary Information 1. [file 41598_2022_24687_MOESM1_ESM.zip › raw data/Metabolomics raw data/4.MetDiffAnalysis/EG.vs.CG/EG.vs.CG_pos_cluster_heatmap_detail.png]

## EG.vs.CG

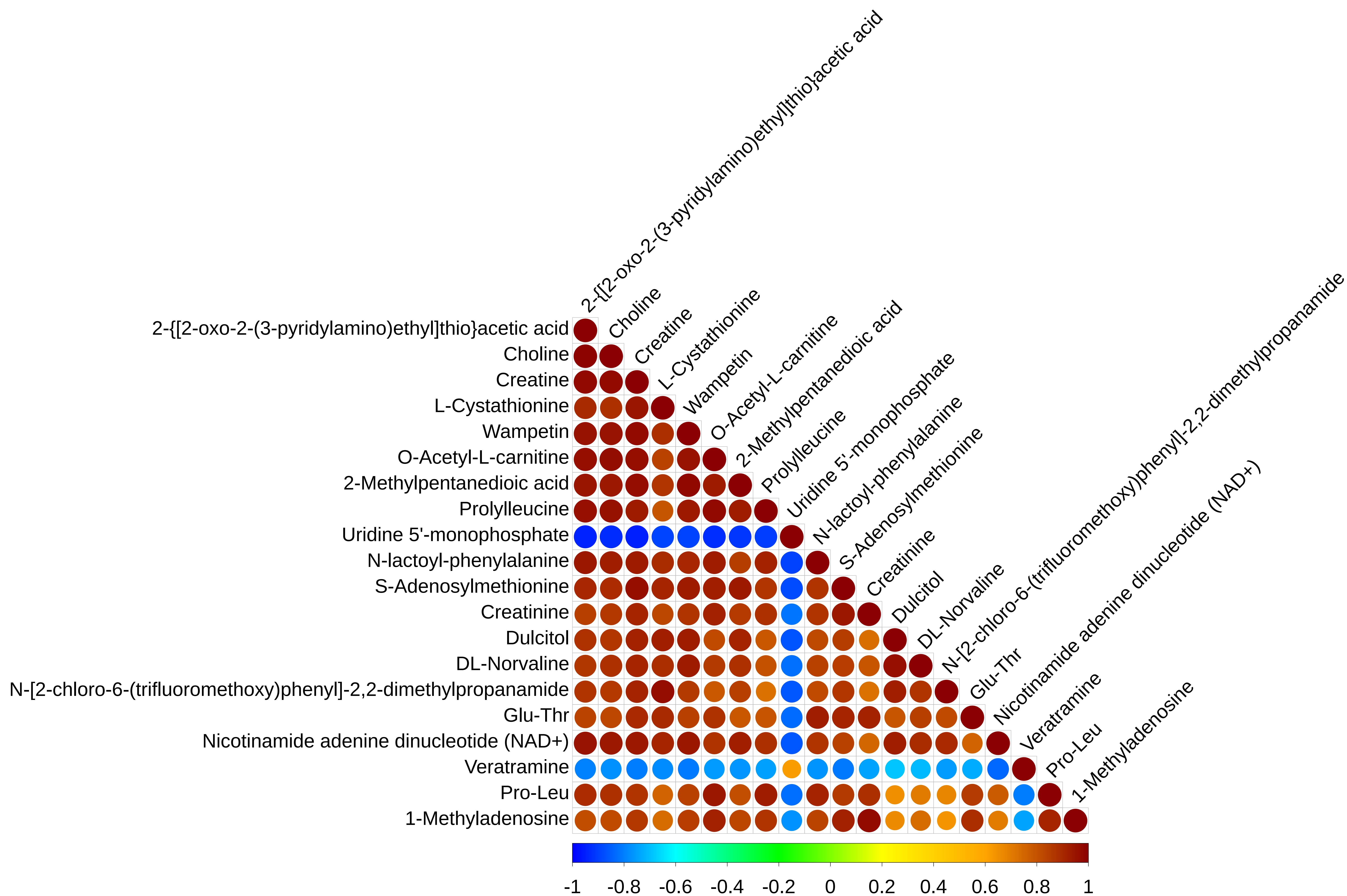

Supplement: Supplementary file 1 — Supplementary Information 1. [file 41598_2022_24687_MOESM1_ESM.zip › raw data/Metabolomics raw data/4.MetDiffAnalysis/EG.vs.CG/EG.vs.CG_pos_corr.pdf]

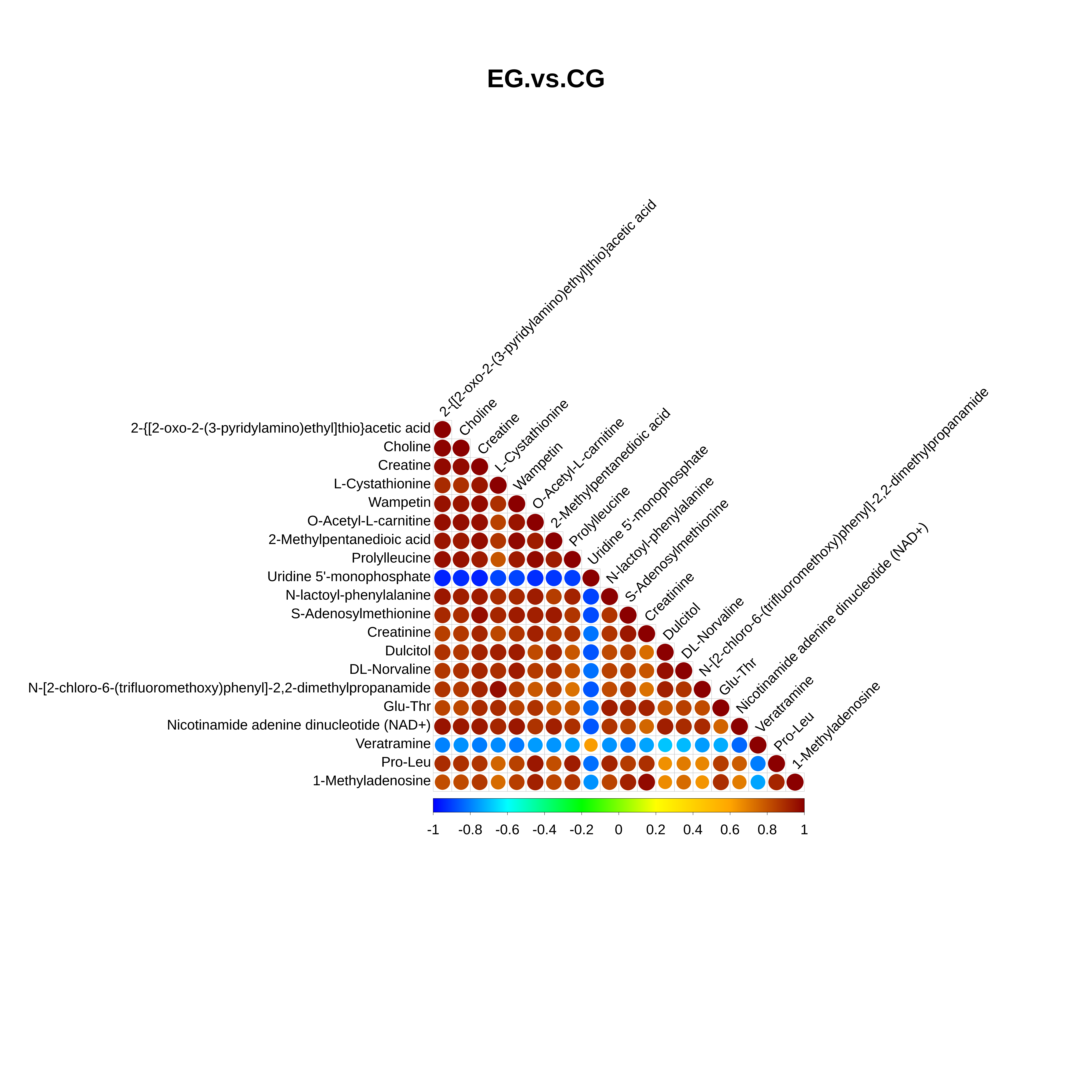

Supplement: Supplementary file 1 — Supplementary Information 1. [file 41598_2022_24687_MOESM1_ESM.zip › raw data/Metabolomics raw data/4.MetDiffAnalysis/EG.vs.CG/EG.vs.CG_pos_corr.png]

EG.vs.CG

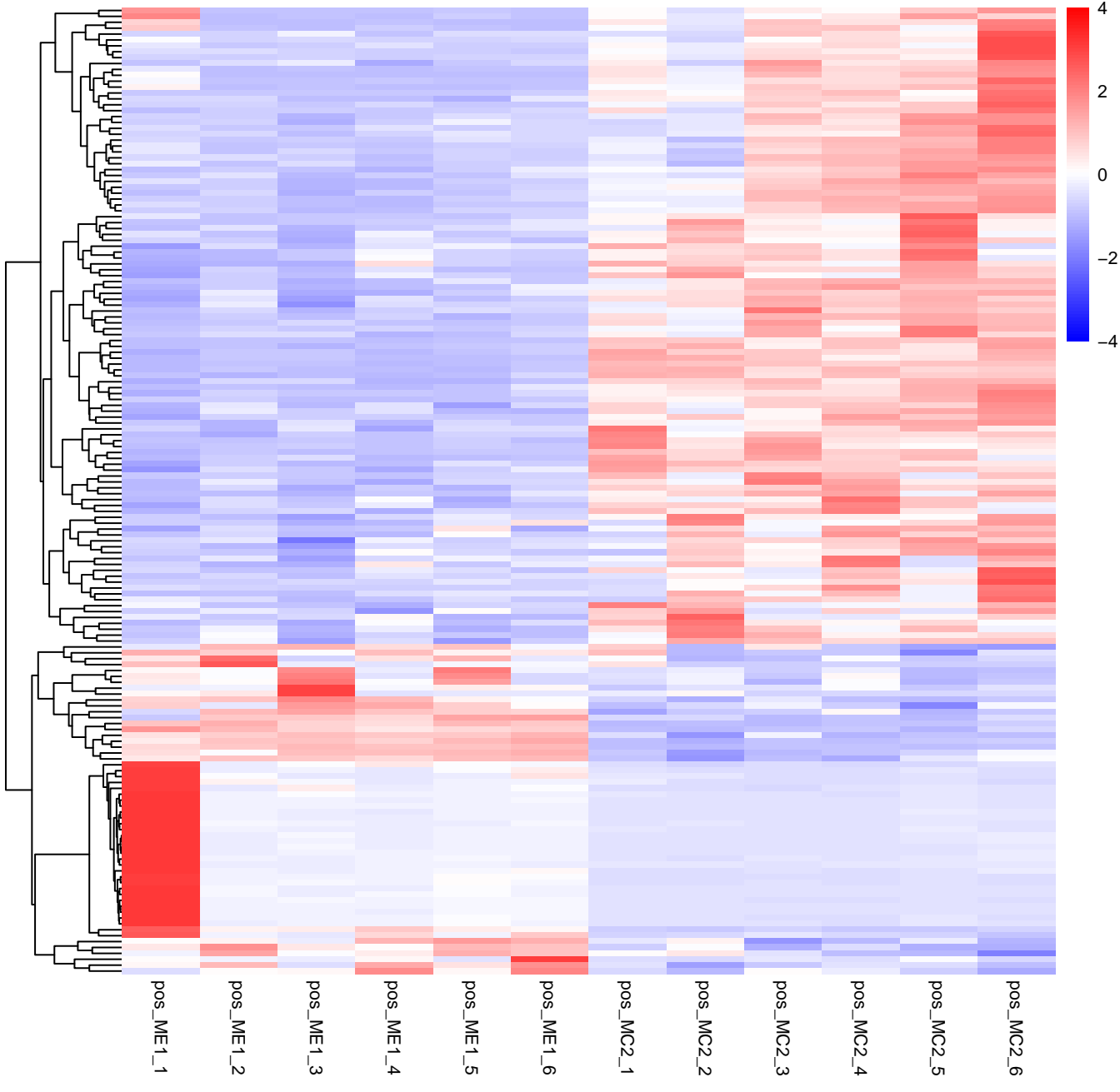

Supplement: Supplementary file 1 — Supplementary Information 1. [file 41598_2022_24687_MOESM1_ESM.zip › raw data/Metabolomics raw data/4.MetDiffAnalysis/EG.vs.CG/EG.vs.CG_pos_heatmap.pdf]

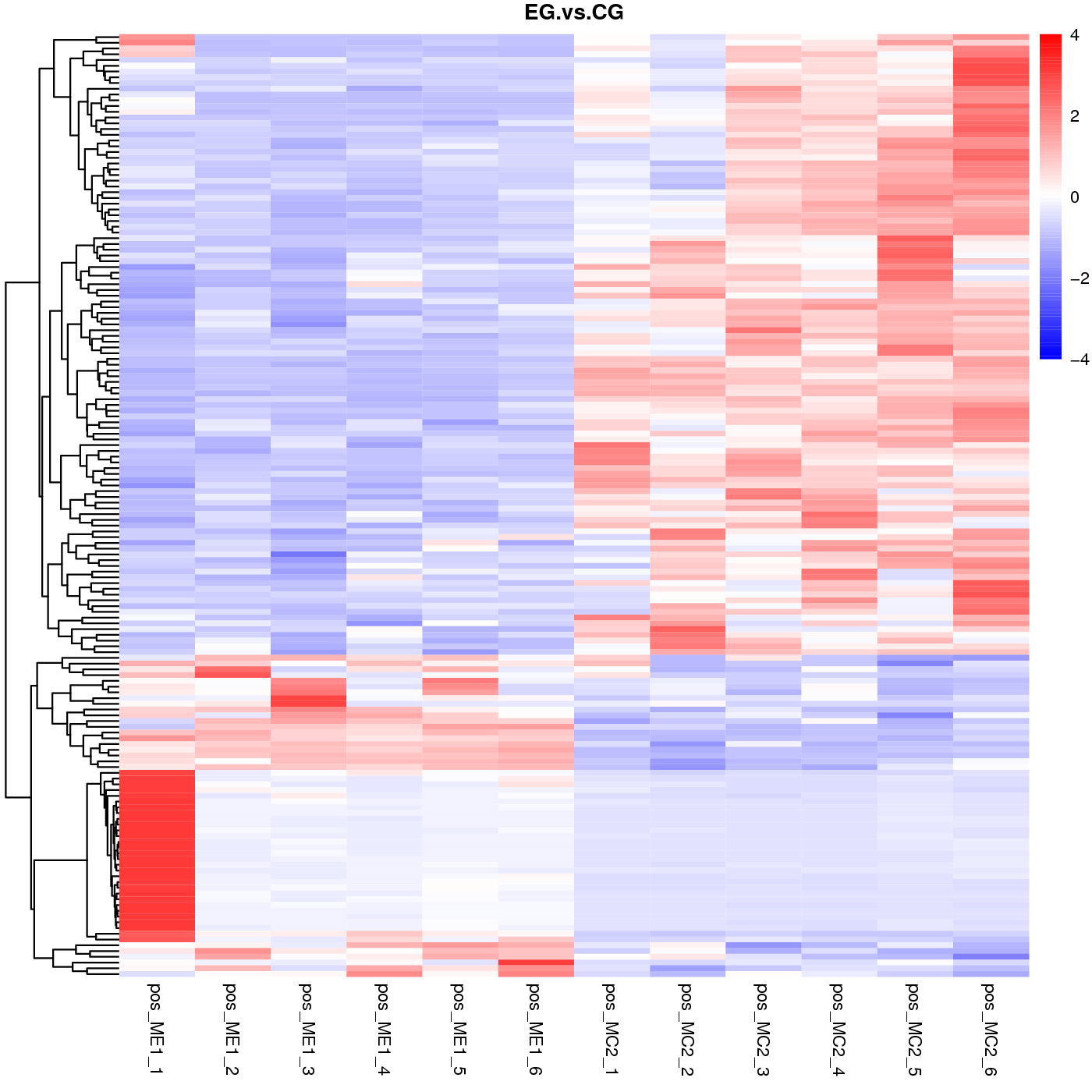

Supplement: Supplementary file 1 — Supplementary Information 1. [file 41598_2022_24687_MOESM1_ESM.zip › raw data/Metabolomics raw data/4.MetDiffAnalysis/EG.vs.CG/EG.vs.CG_pos_heatmap.png]

EG.vs.CG

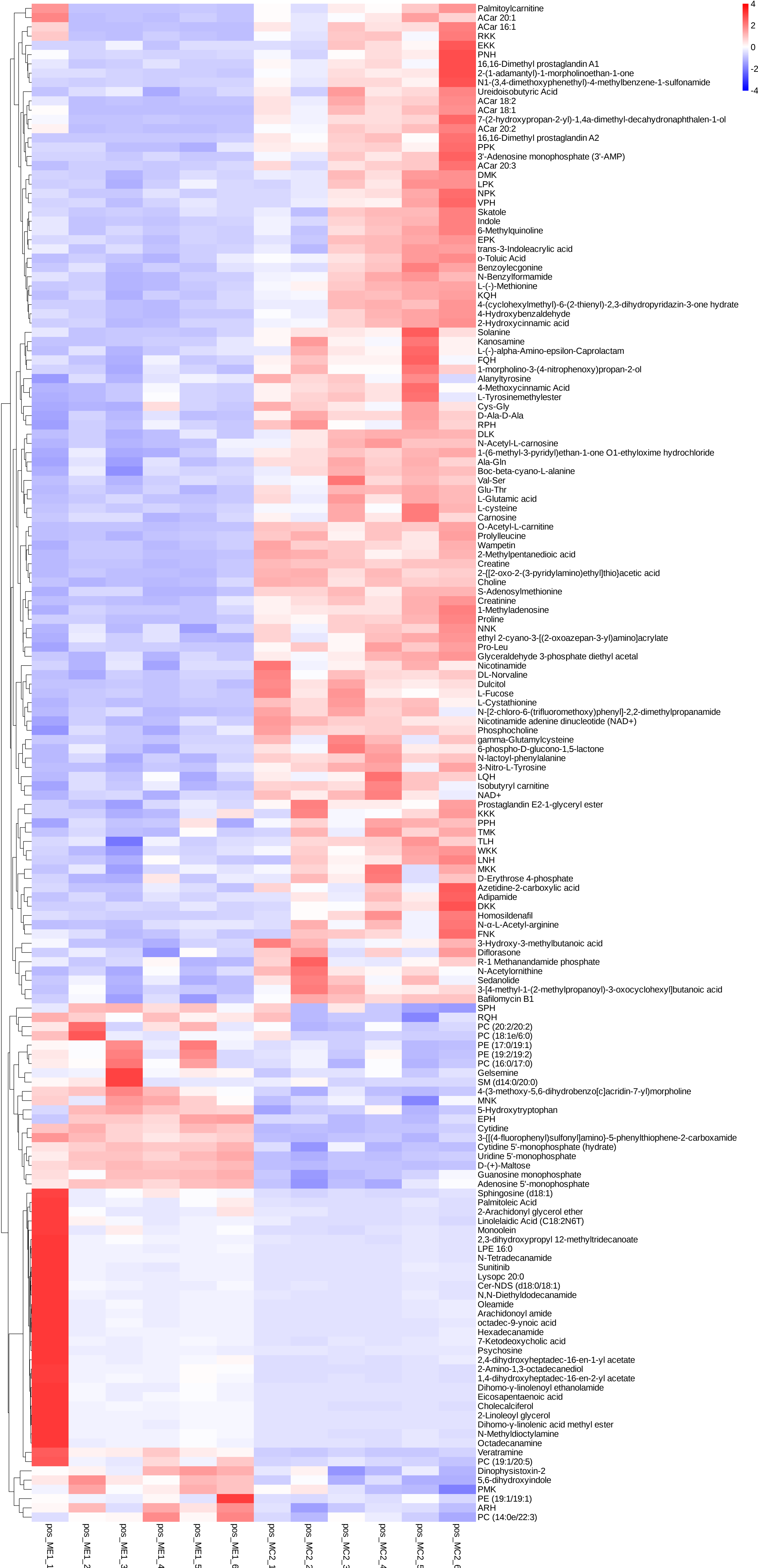

Supplement: Supplementary file 1 — Supplementary Information 1. [file 41598_2022_24687_MOESM1_ESM.zip › raw data/Metabolomics raw data/4.MetDiffAnalysis/EG.vs.CG/EG.vs.CG_pos_heatmap_detail.pdf]

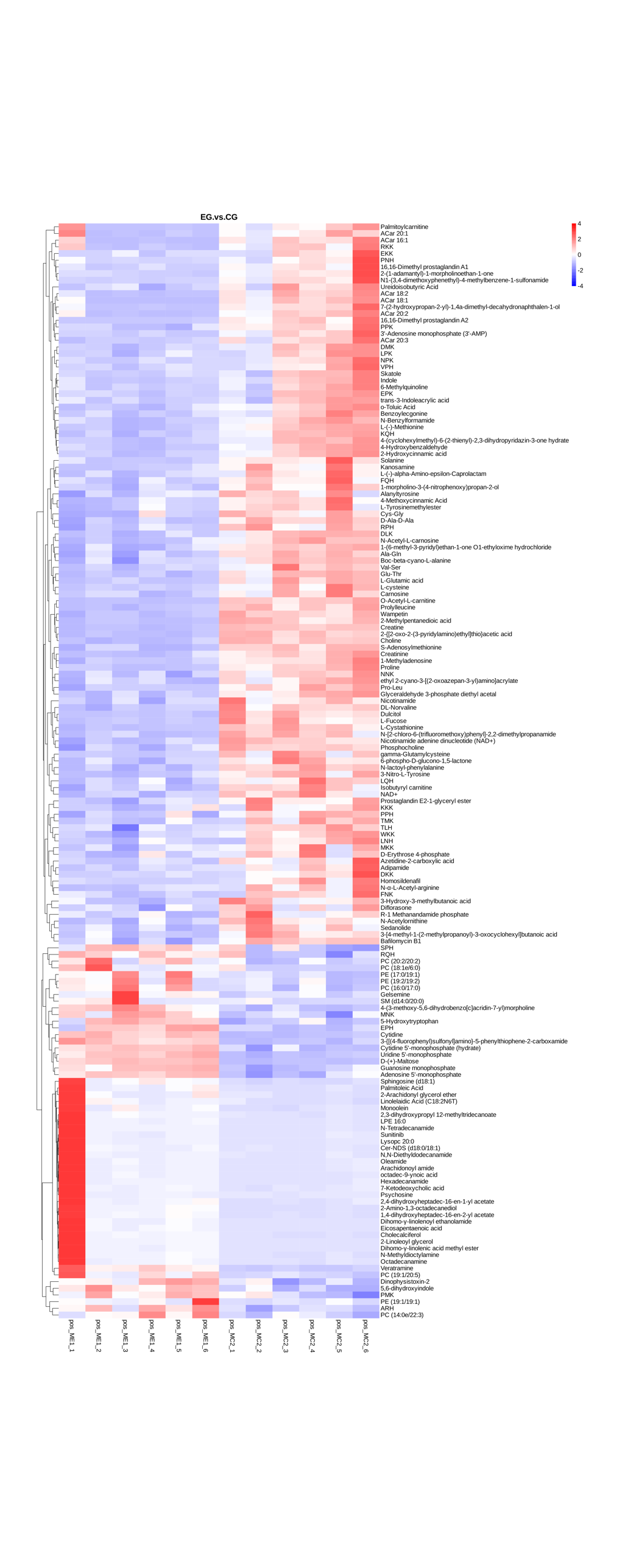

Supplement: Supplementary file 1 — Supplementary Information 1. [file 41598_2022_24687_MOESM1_ESM.zip › raw data/Metabolomics raw data/4.MetDiffAnalysis/EG.vs.CG/EG.vs.CG_pos_heatmap_detail.png]

## EG.vs.CG

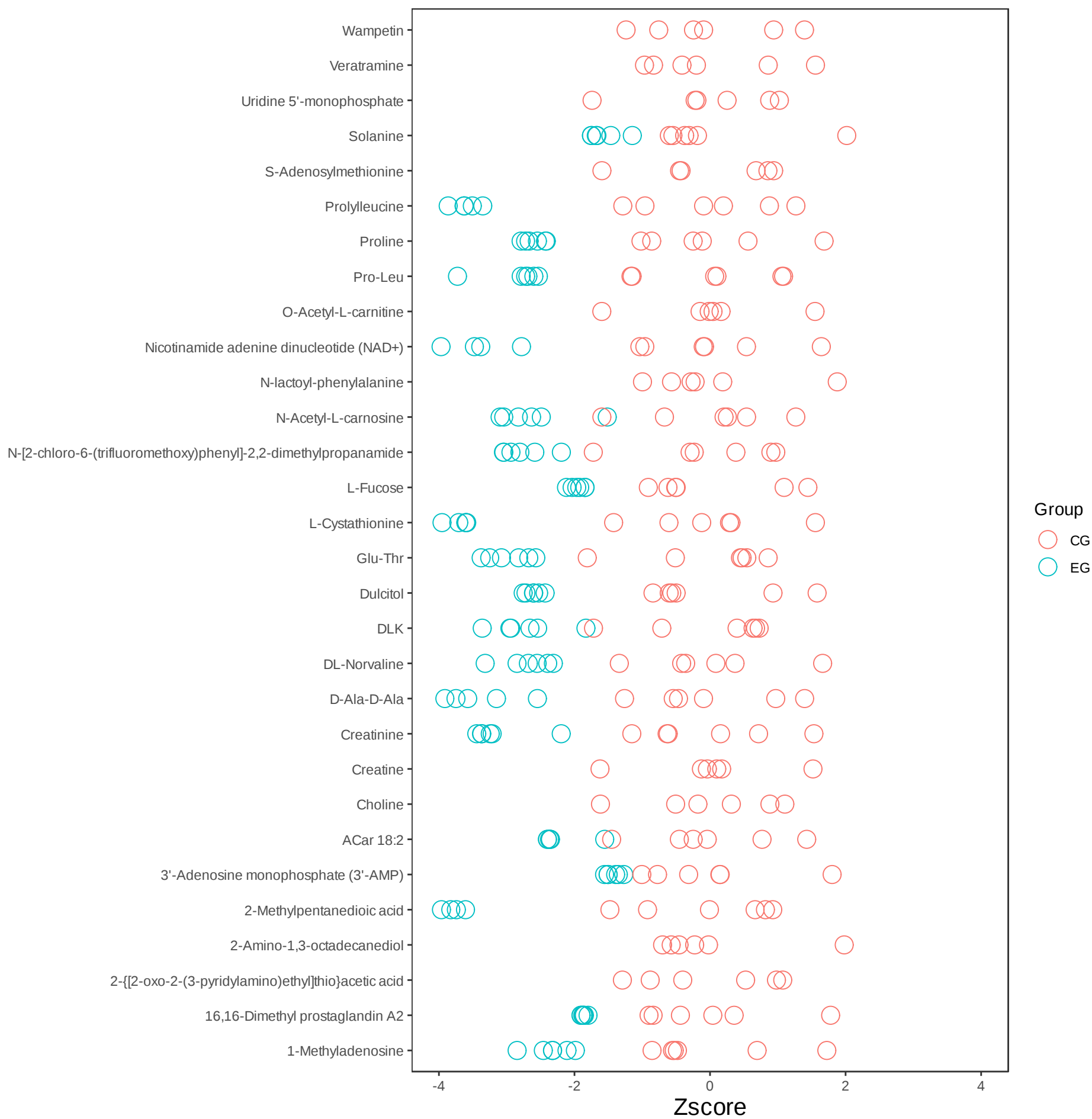

Supplement: Supplementary file 1 — Supplementary Information 1. [file 41598_2022_24687_MOESM1_ESM.zip › raw data/Metabolomics raw data/4.MetDiffAnalysis/EG.vs.CG/EG.vs.CG_pos_zscore.pdf]

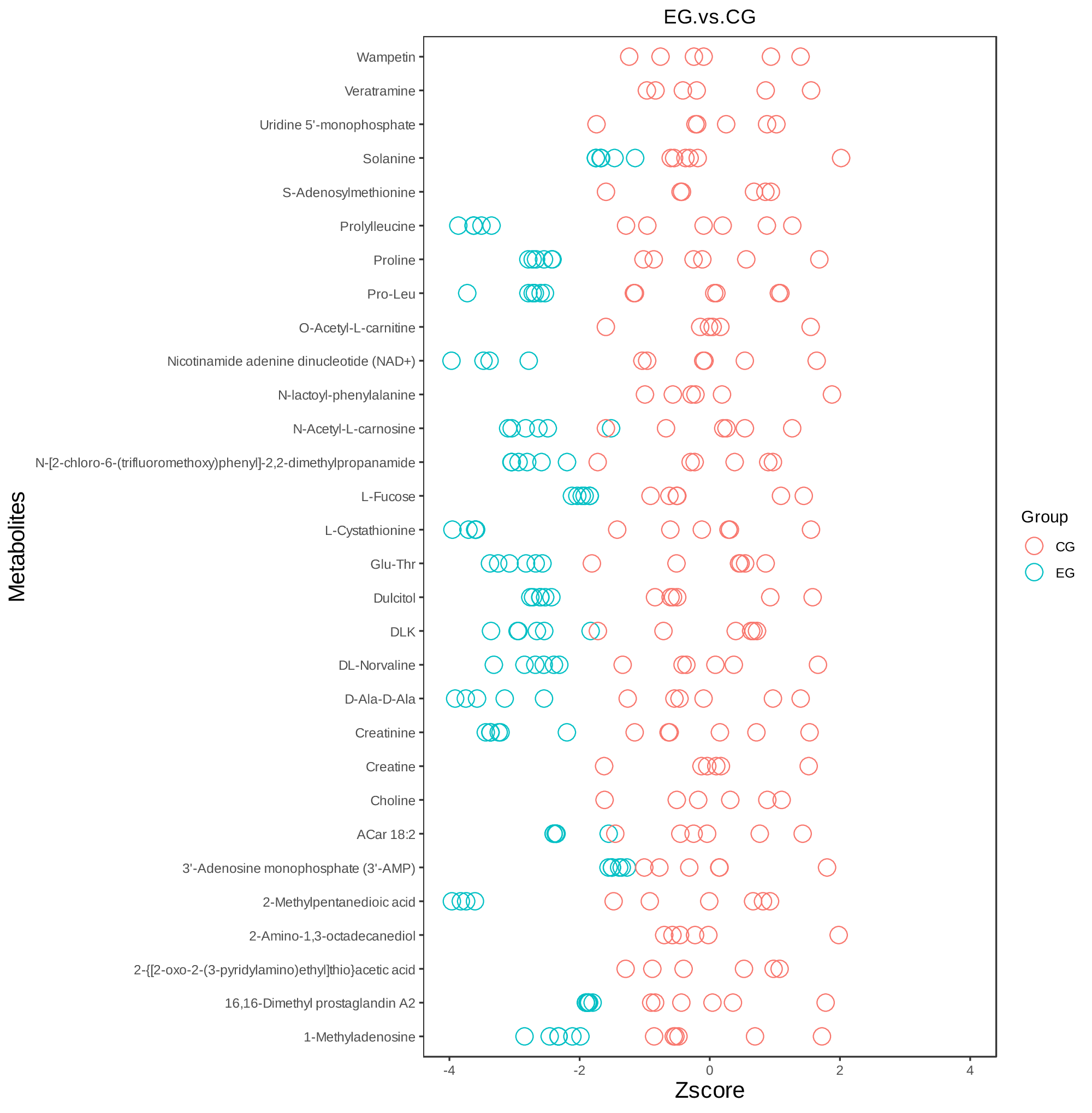

Supplement: Supplementary file 1 — Supplementary Information 1. [file 41598_2022_24687_MOESM1_ESM.zip › raw data/Metabolomics raw data/4.MetDiffAnalysis/EG.vs.CG/EG.vs.CG_pos_zscore.png]

# EG.vs.CG

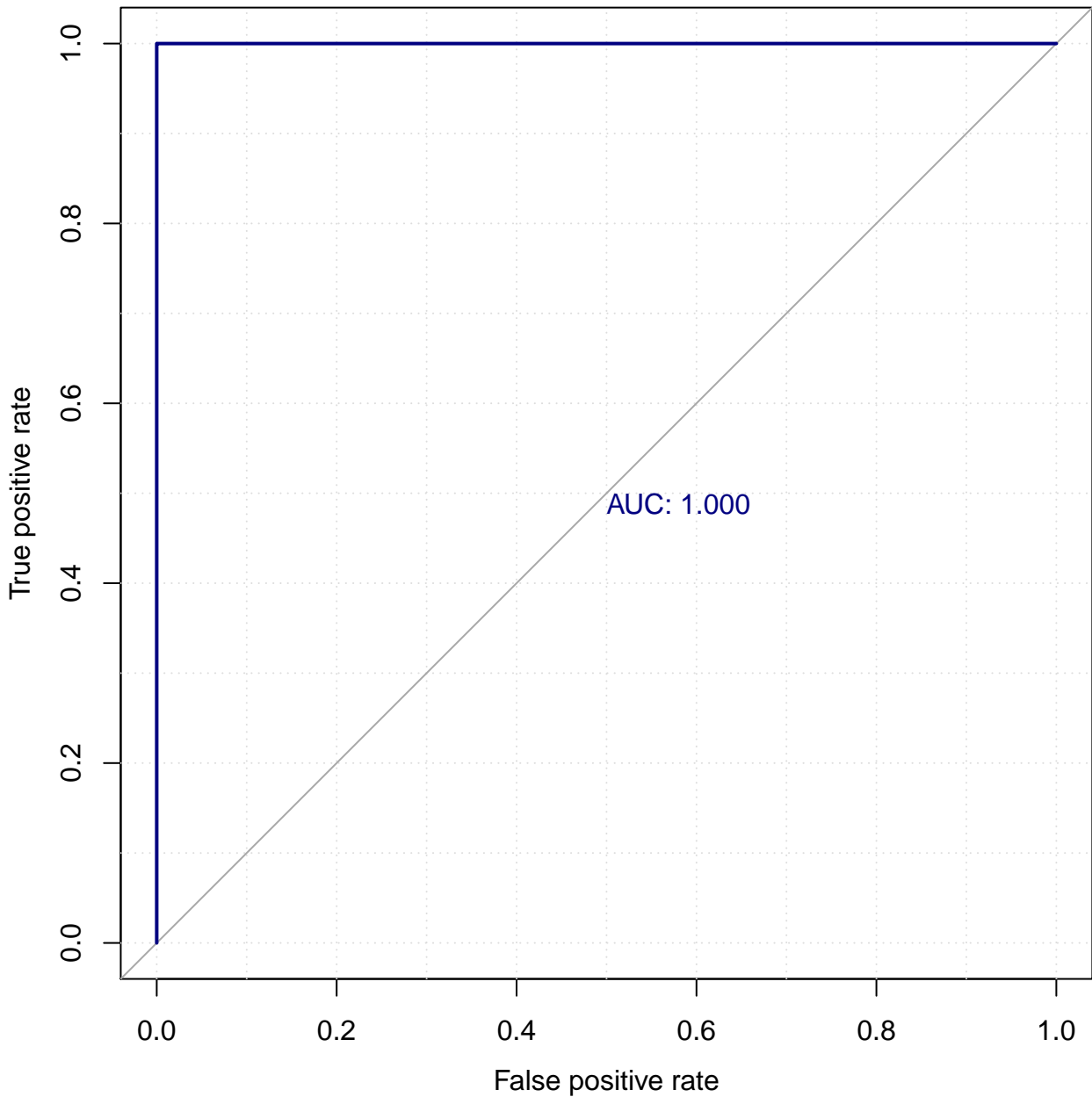

Supplement: Supplementary file 1 — Supplementary Information 1. [file 41598_2022_24687_MOESM1_ESM.zip › raw data/Metabolomics raw data/4.MetDiffAnalysis/EG.vs.CG/ROC_neg/Com_100_neg_ROC.pdf]

# EG.vs.CG

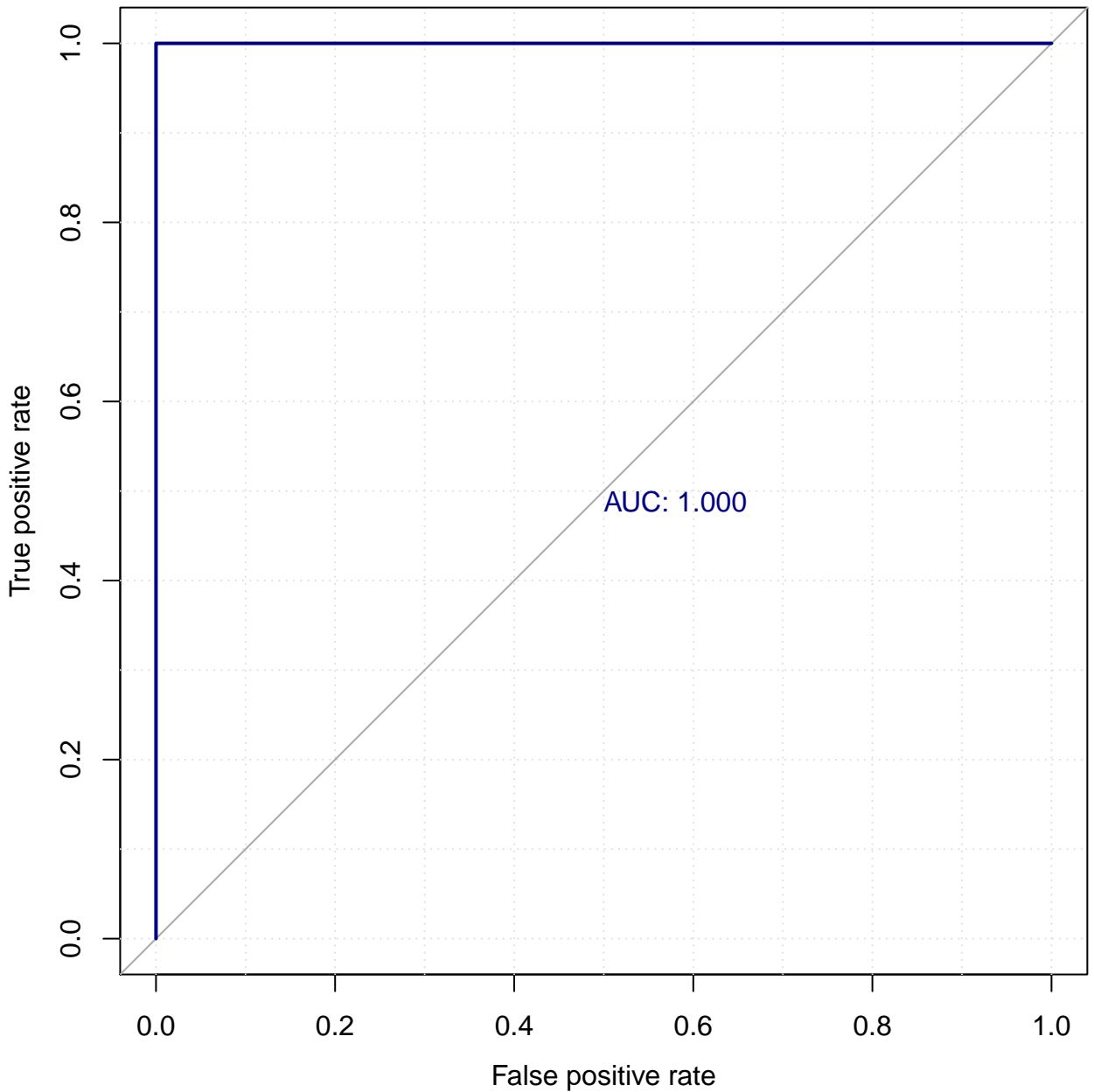

Supplement: Supplementary file 1 — Supplementary Information 1. [file 41598_2022_24687_MOESM1_ESM.zip › raw data/Metabolomics raw data/4.MetDiffAnalysis/EG.vs.CG/ROC_neg/Com_10399_neg_ROC.pdf]

# EG.vs.CG

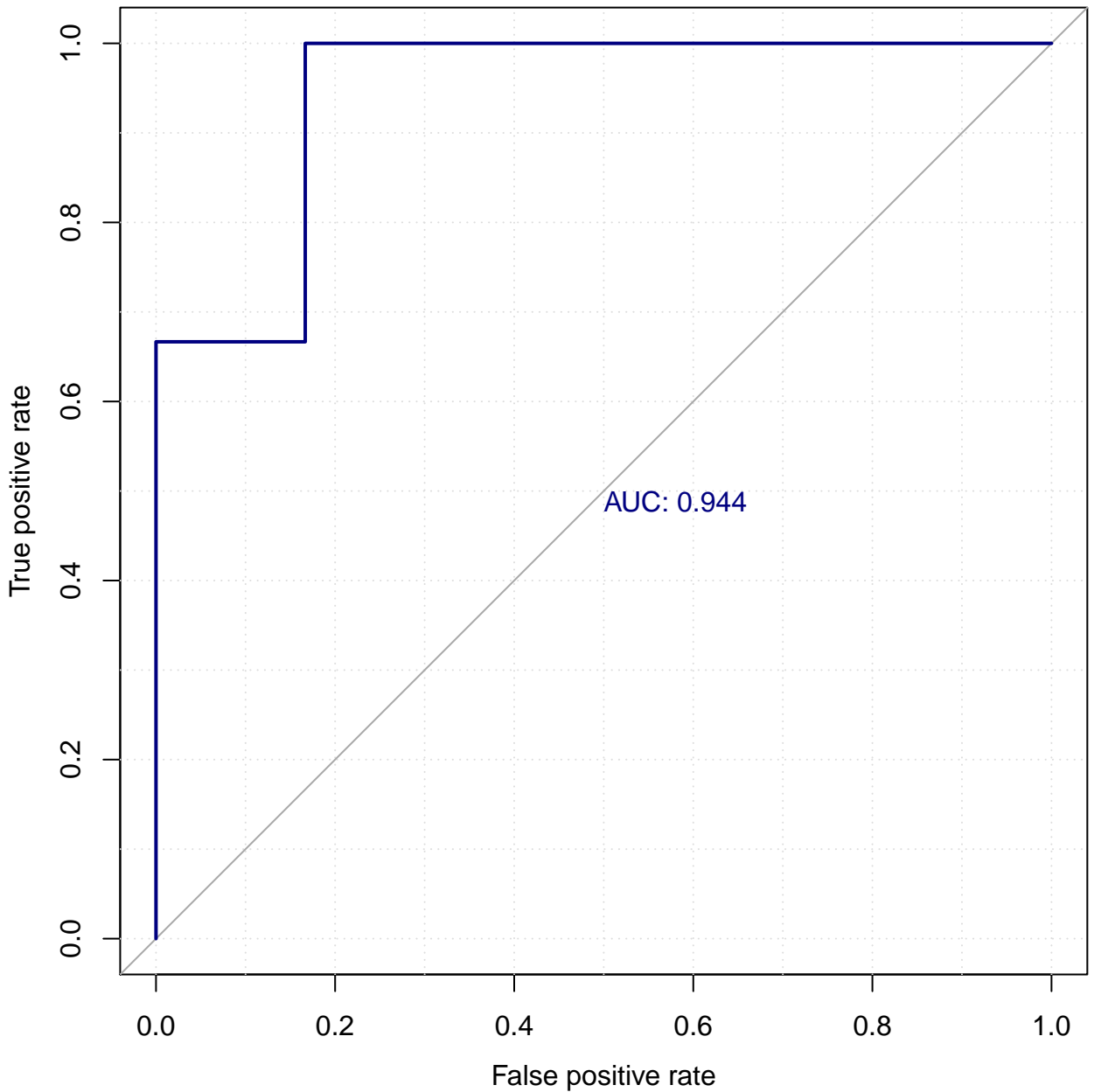

Supplement: Supplementary file 1 — Supplementary Information 1. [file 41598_2022_24687_MOESM1_ESM.zip › raw data/Metabolomics raw data/4.MetDiffAnalysis/EG.vs.CG/ROC_neg/Com_10502_neg_ROC.pdf]

# EG.vs.CG

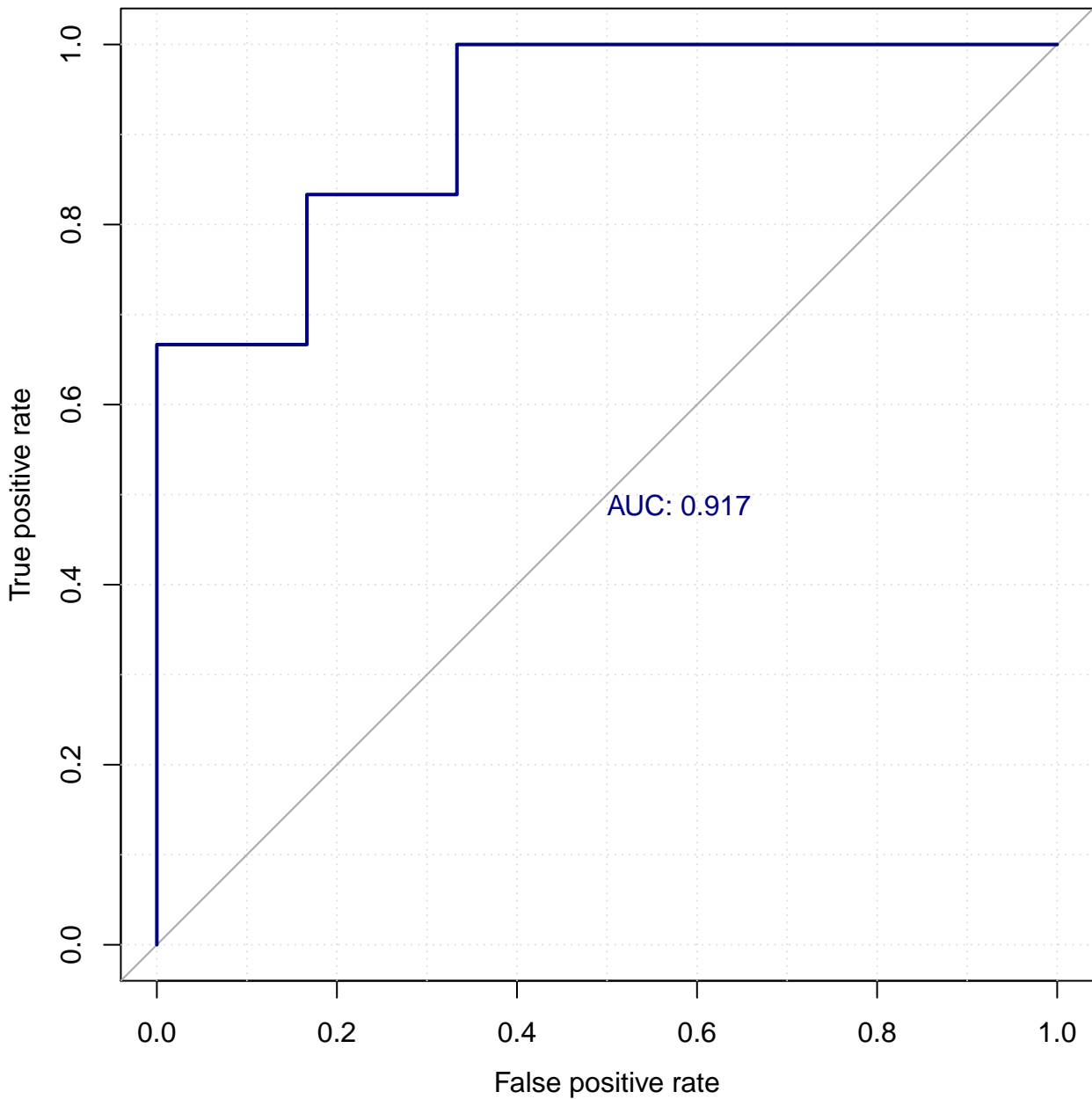

Supplement: Supplementary file 1 — Supplementary Information 1. [file 41598_2022_24687_MOESM1_ESM.zip › raw data/Metabolomics raw data/4.MetDiffAnalysis/EG.vs.CG/ROC_neg/Com_1051_neg_ROC.pdf]

# EG.vs.CG

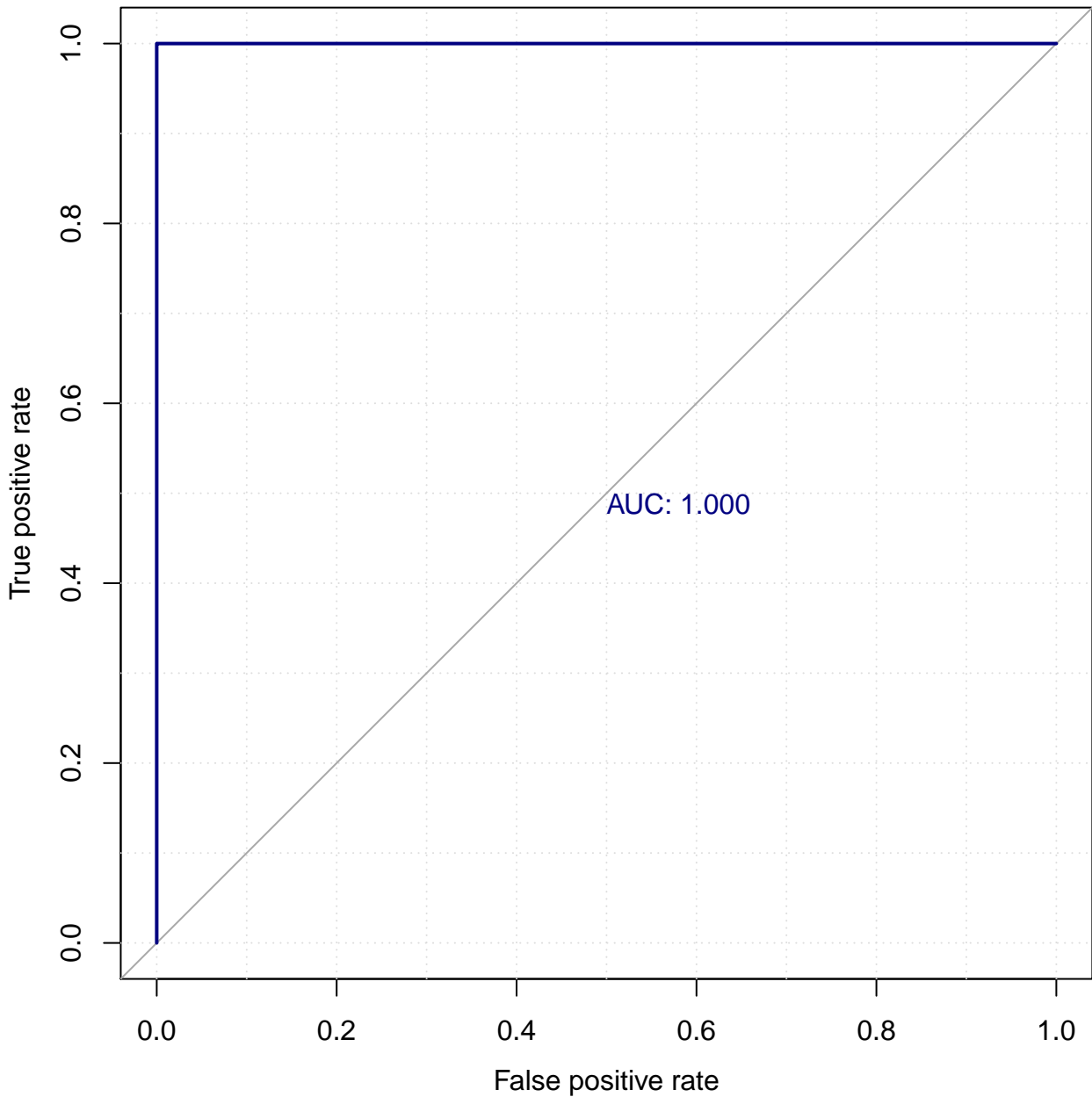

Supplement: Supplementary file 1 — Supplementary Information 1. [file 41598_2022_24687_MOESM1_ESM.zip › raw data/Metabolomics raw data/4.MetDiffAnalysis/EG.vs.CG/ROC_neg/Com_1054_neg_ROC.pdf]

# EG.vs.CG

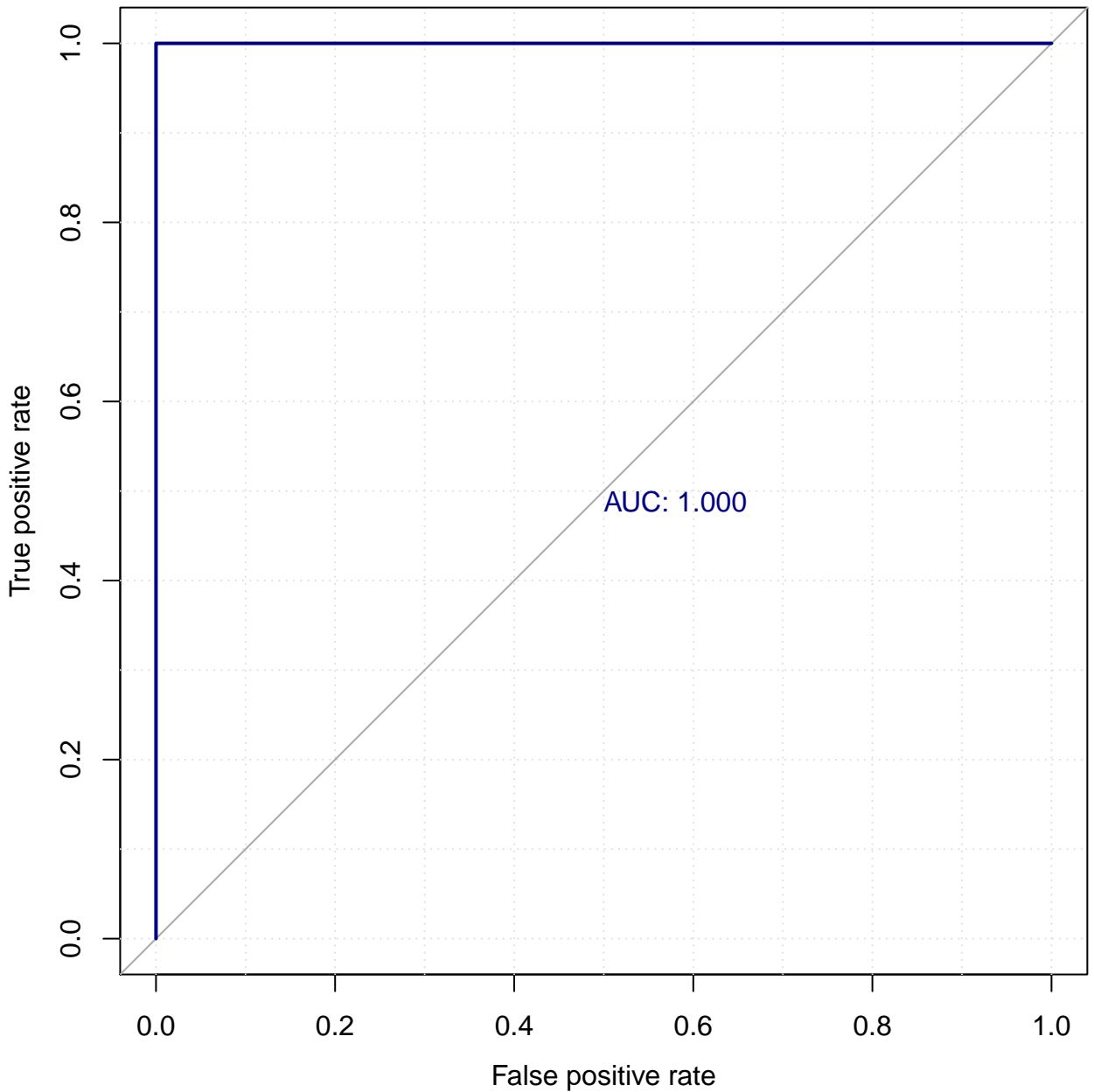

Supplement: Supplementary file 1 — Supplementary Information 1. [file 41598_2022_24687_MOESM1_ESM.zip › raw data/Metabolomics raw data/4.MetDiffAnalysis/EG.vs.CG/ROC_neg/Com_10641_neg_ROC.pdf]

# EG.vs.CG

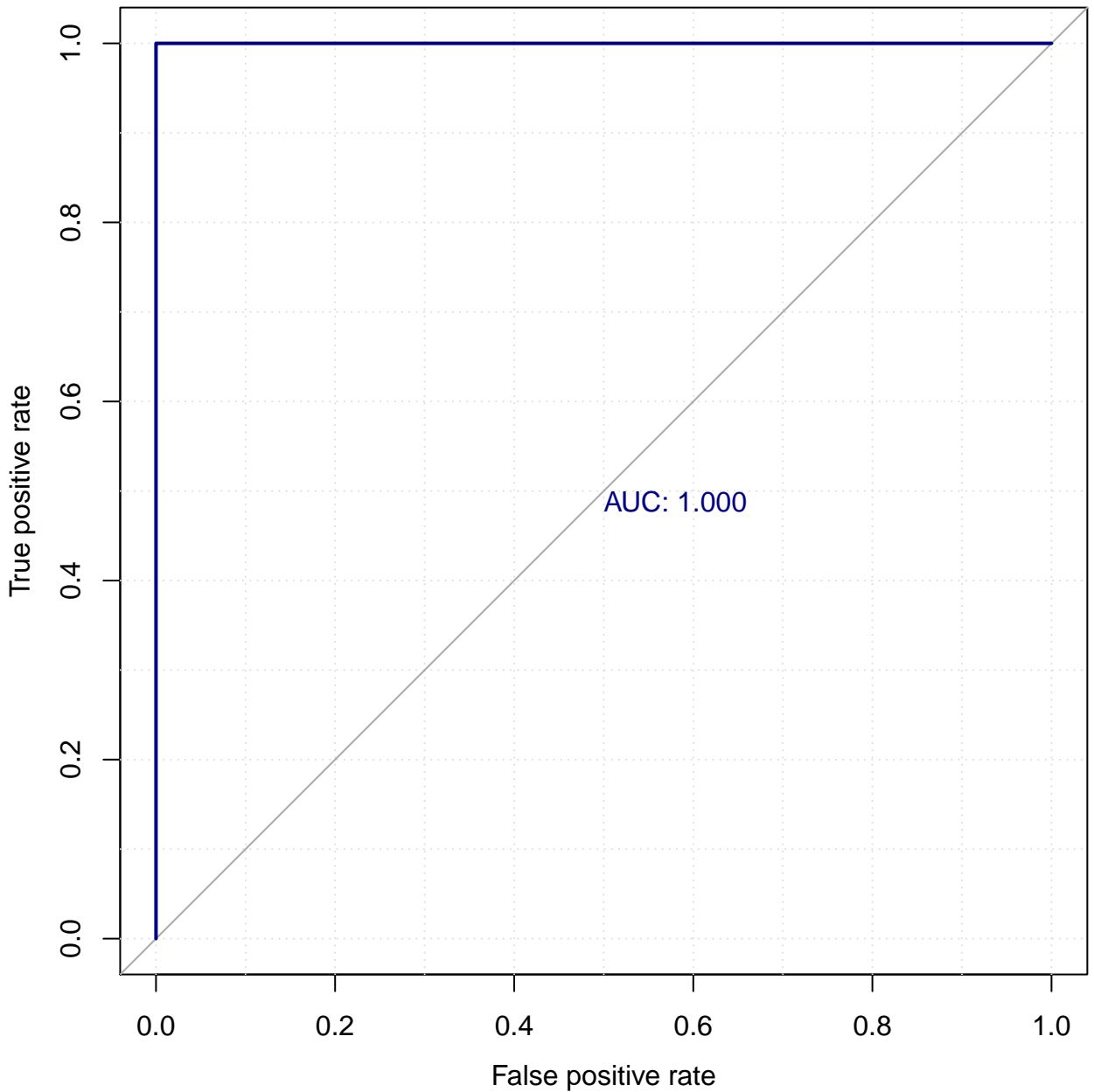

Supplement: Supplementary file 1 — Supplementary Information 1. [file 41598_2022_24687_MOESM1_ESM.zip › raw data/Metabolomics raw data/4.MetDiffAnalysis/EG.vs.CG/ROC_neg/Com_10859_neg_ROC.pdf]

# EG.vs.CG

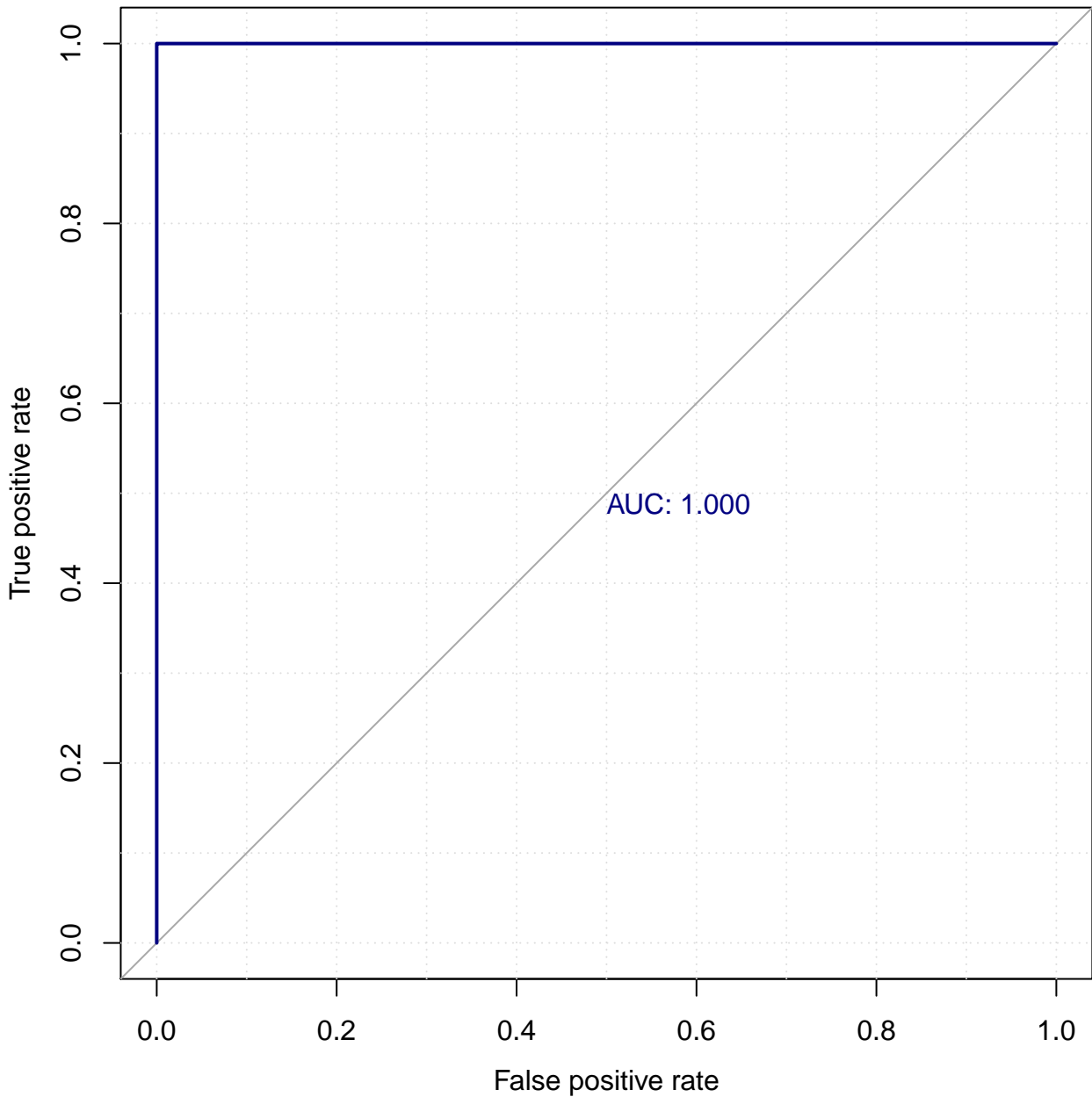

Supplement: Supplementary file 1 — Supplementary Information 1. [file 41598_2022_24687_MOESM1_ESM.zip › raw data/Metabolomics raw data/4.MetDiffAnalysis/EG.vs.CG/ROC_neg/Com_1090_neg_ROC.pdf]

# EG.vs.CG

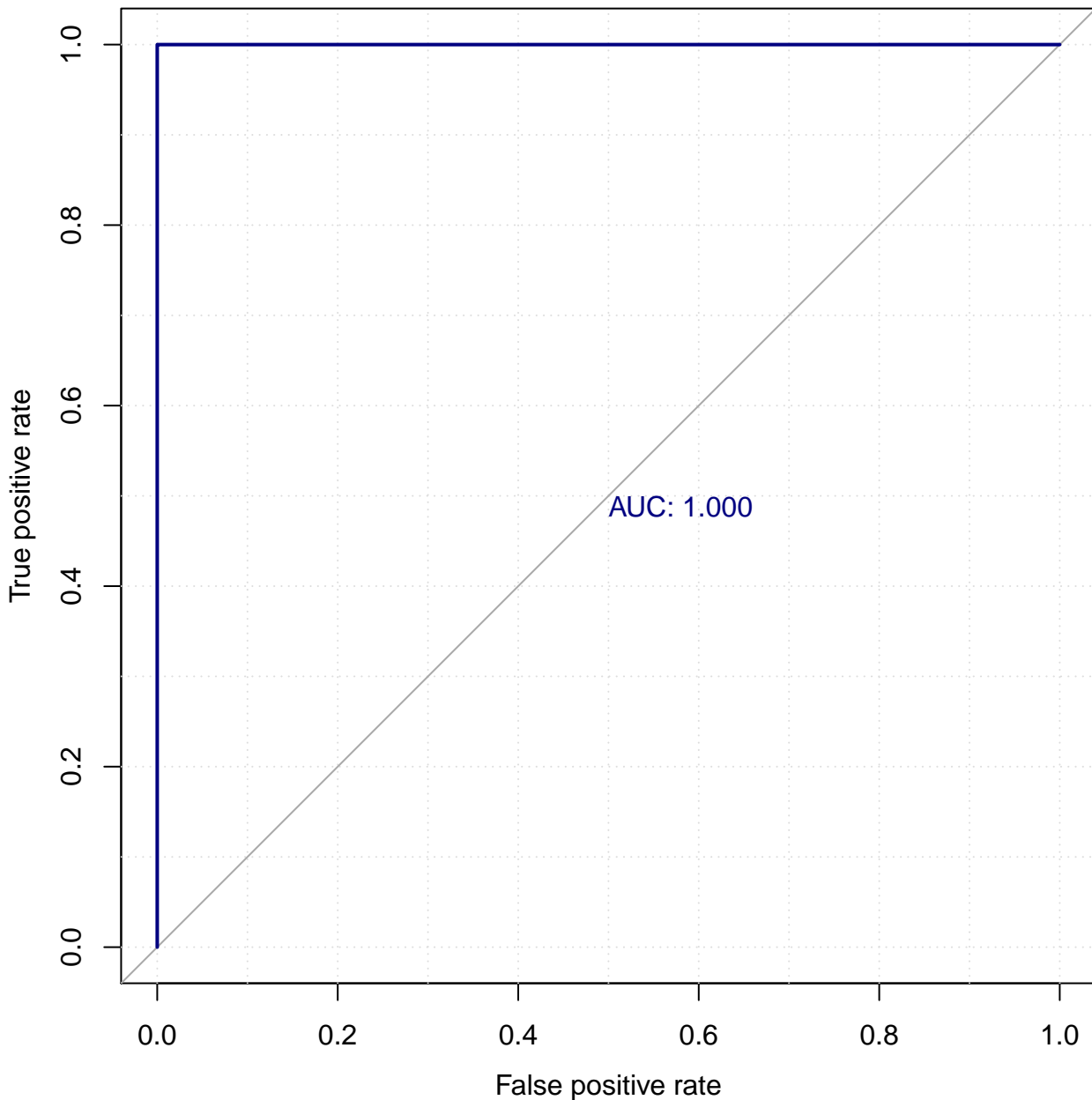

Supplement: Supplementary file 1 — Supplementary Information 1. [file 41598_2022_24687_MOESM1_ESM.zip › raw data/Metabolomics raw data/4.MetDiffAnalysis/EG.vs.CG/ROC_neg/Com_1099_neg_ROC.pdf]

# EG.vs.CG

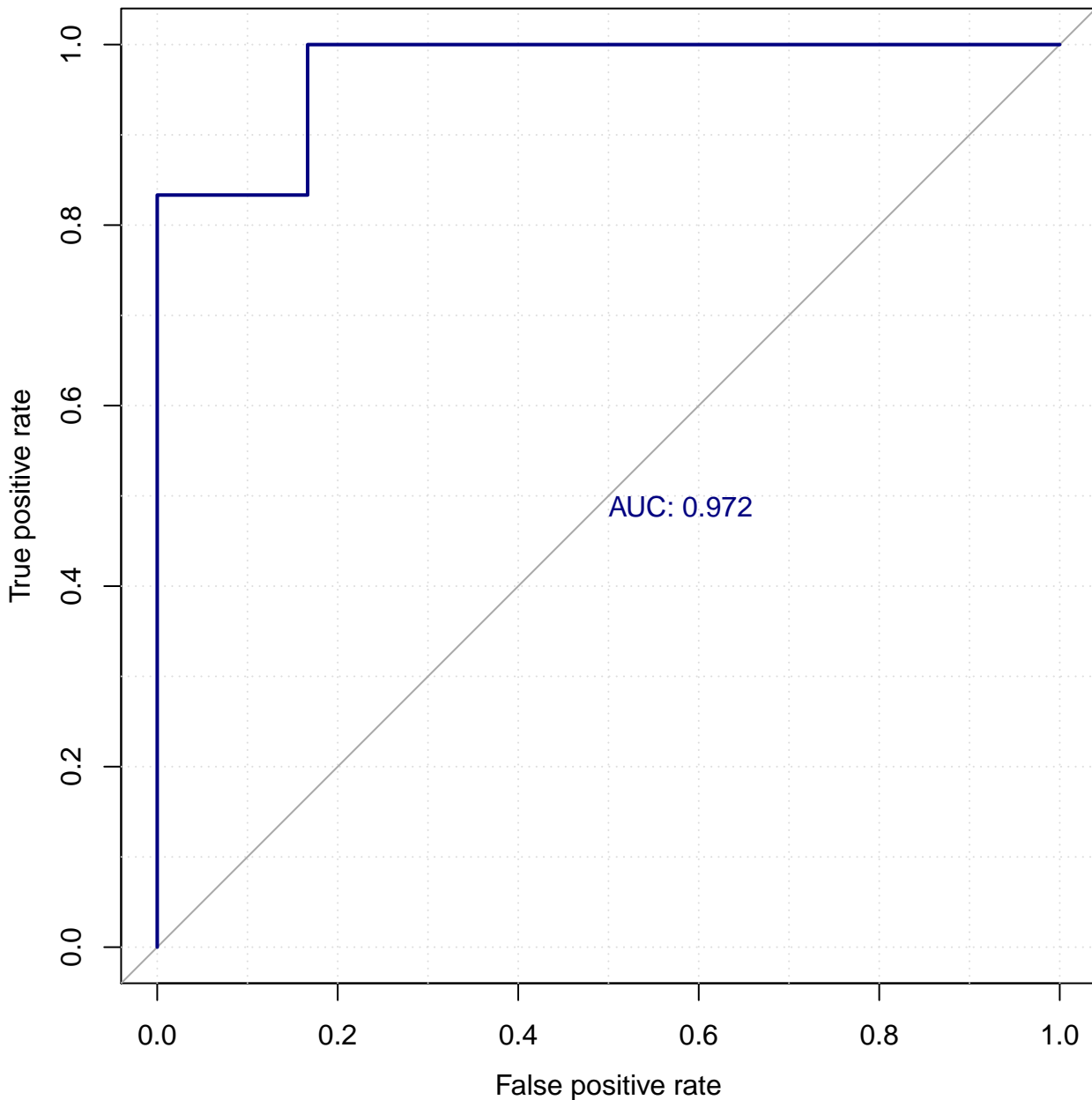

Supplement: Supplementary file 1 — Supplementary Information 1. [file 41598_2022_24687_MOESM1_ESM.zip › raw data/Metabolomics raw data/4.MetDiffAnalysis/EG.vs.CG/ROC_neg/Com_11057_neg_ROC.pdf]

# EG.vs.CG

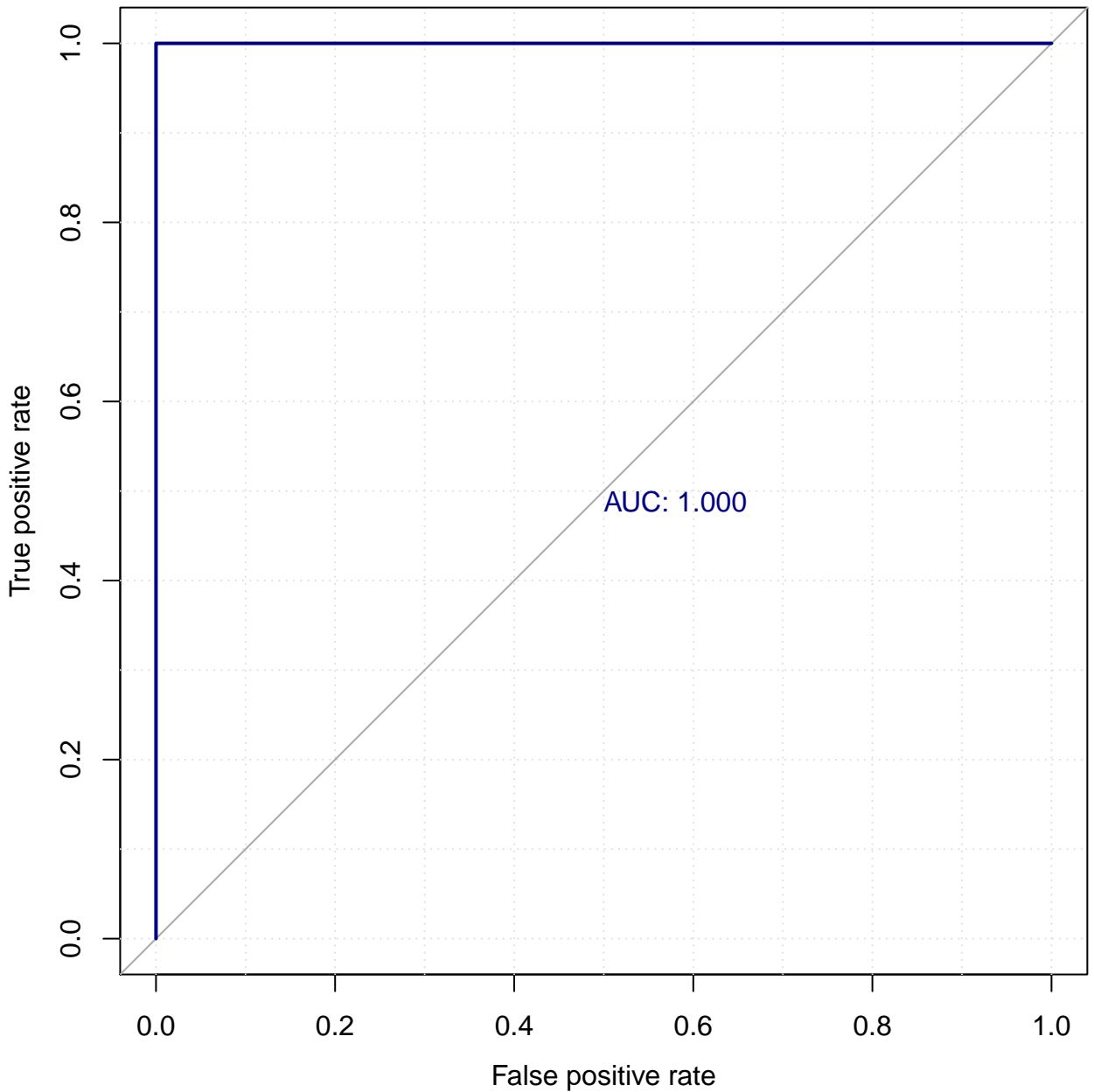

Supplement: Supplementary file 1 — Supplementary Information 1. [file 41598_2022_24687_MOESM1_ESM.zip › raw data/Metabolomics raw data/4.MetDiffAnalysis/EG.vs.CG/ROC_neg/Com_11269_neg_ROC.pdf]

# EG.vs.CG

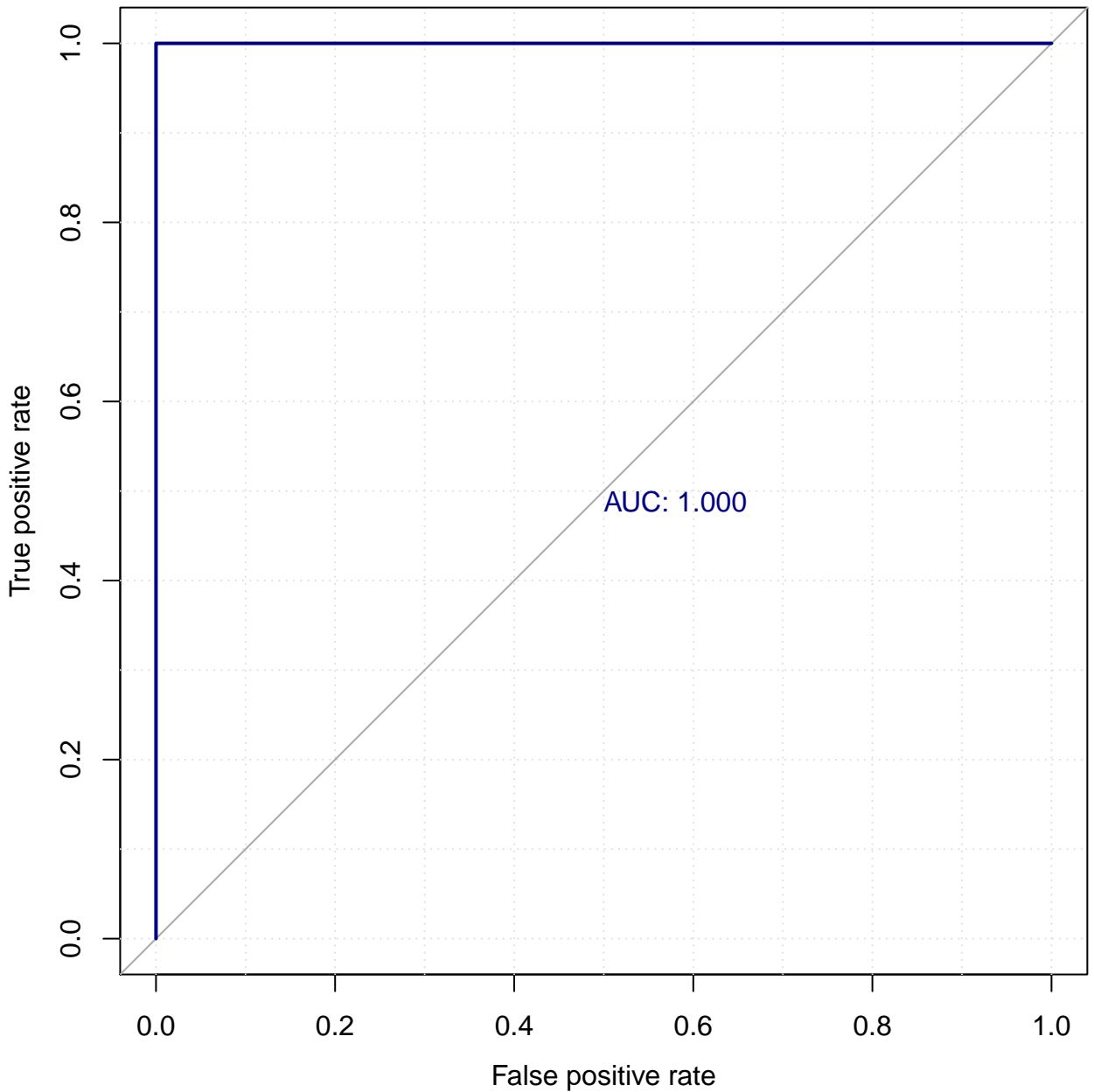

Supplement: Supplementary file 1 — Supplementary Information 1. [file 41598_2022_24687_MOESM1_ESM.zip › raw data/Metabolomics raw data/4.MetDiffAnalysis/EG.vs.CG/ROC_neg/Com_1170_neg_ROC.pdf]

# EG.vs.CG

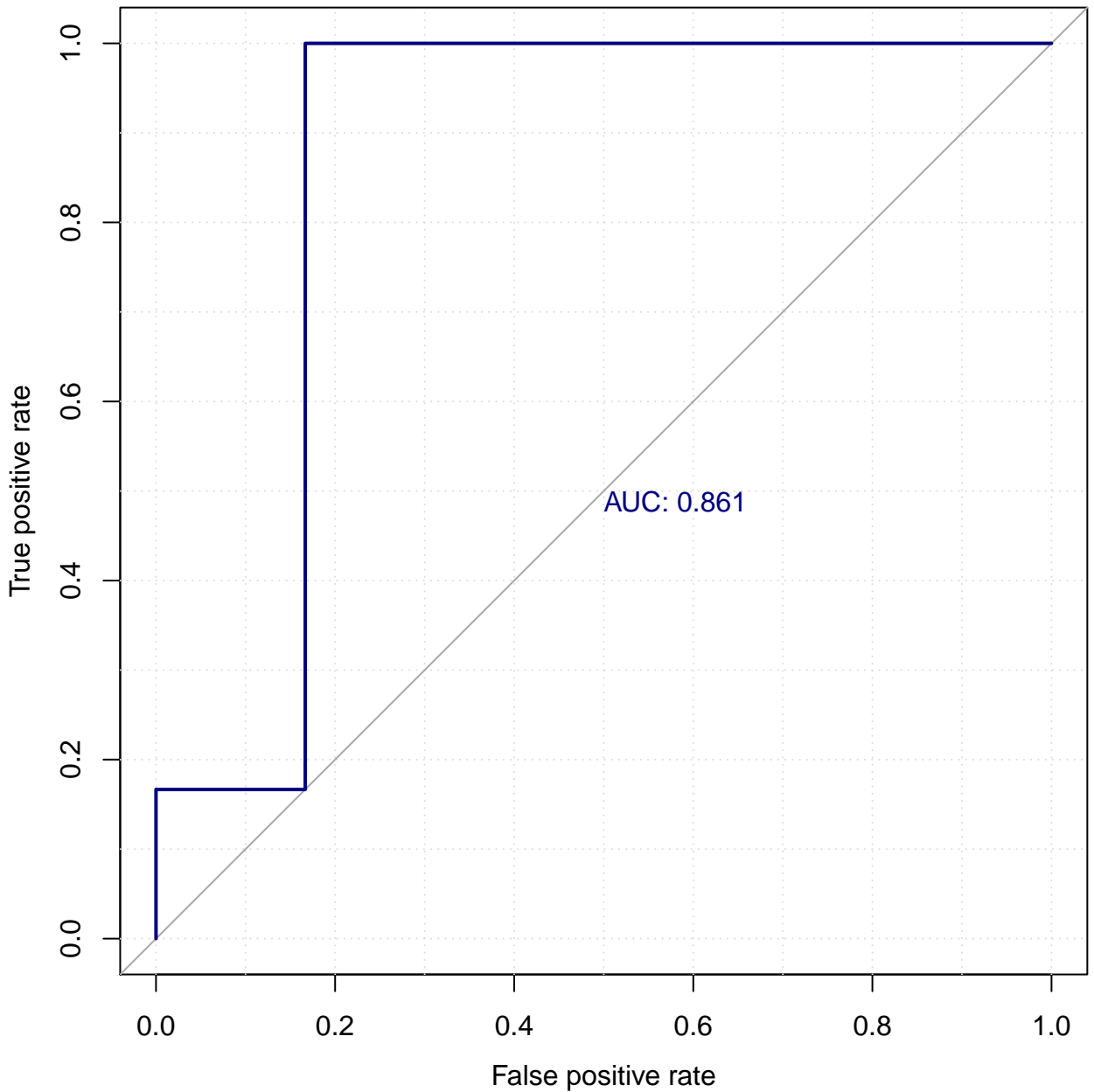

Supplement: Supplementary file 1 — Supplementary Information 1. [file 41598_2022_24687_MOESM1_ESM.zip › raw data/Metabolomics raw data/4.MetDiffAnalysis/EG.vs.CG/ROC_neg/Com_1208_neg_ROC.pdf]

# EG.vs.CG

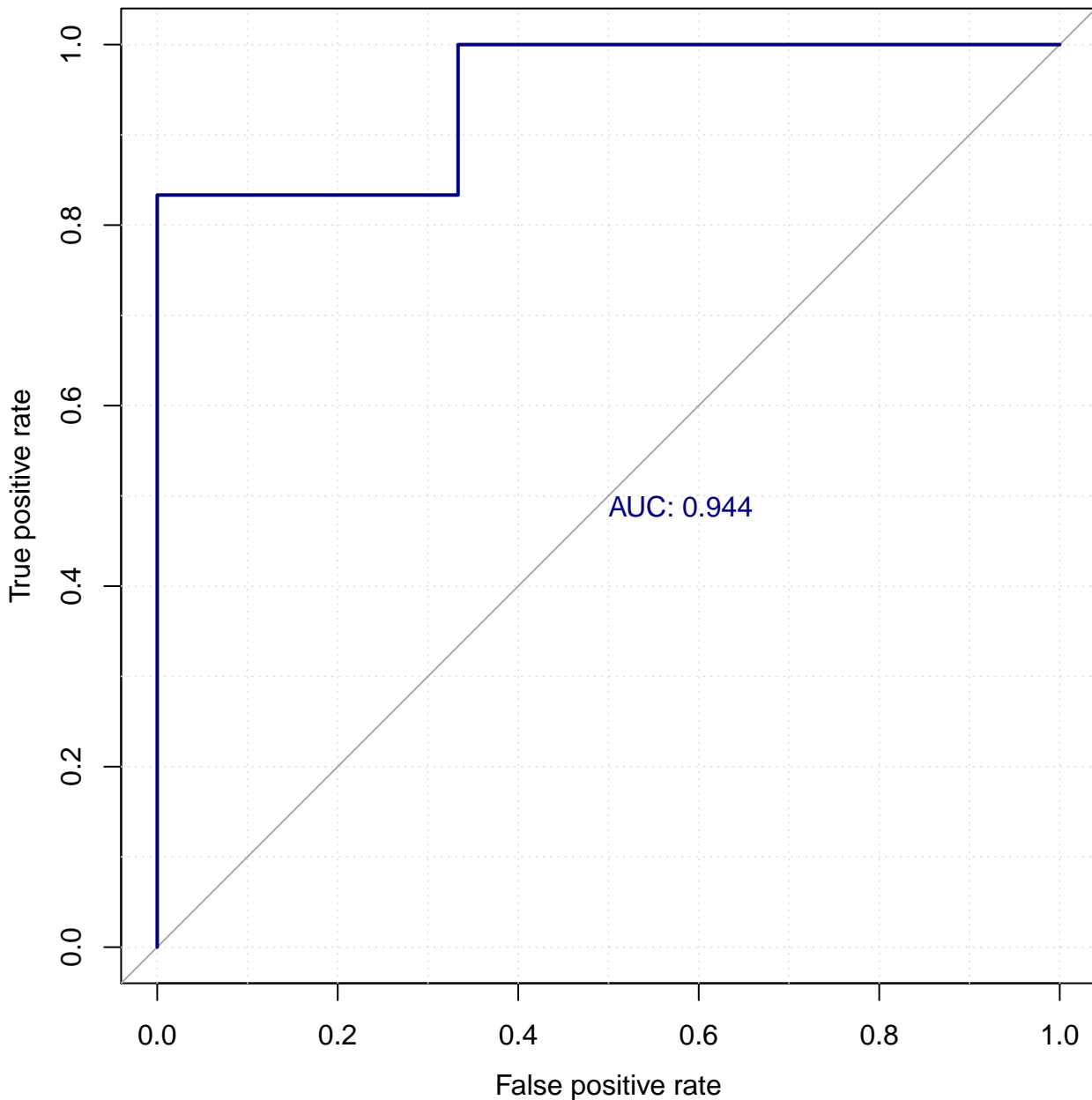

Supplement: Supplementary file 1 — Supplementary Information 1. [file 41598_2022_24687_MOESM1_ESM.zip › raw data/Metabolomics raw data/4.MetDiffAnalysis/EG.vs.CG/ROC_neg/Com_12417_neg_ROC.pdf]
