# Supplementary material for: Comparative multiomics analysis of cell physiological state after culture in a basket bioreactor
Source: Sci Rep. 2022 Nov 23;12:20161. doi: 10.1038/s41598-022-24687-4 (PMC9686226; doi:10.1038/s41598-022-24687-4)
Supplement: Supplementary file 2 — Supplementary Information 2. [file 41598_2022_24687_MOESM2_ESM.docx]

**Supporting Information**

**For**

Comparative Multiomics Analysis of Cell Physiological State after Culture in a Basket Bioreactor

Shouzhi Yu ^1,†^, Miaomiao Guo ^1,†^, Yadan Zhang ^1,†^, Cunpei Bo ^1^,Hongyang Liang ^1^,

Hui Wang ^1*^, Xiaoming Yang ^1,2,*^

^1^Beijing Institute of Biological Products Company Limited, Beijing, China

^2^China National Biotec Group Company Limited, Beijing, China

^†^These authors contributed equally

*Correspondence: wanghui_bsy@163.com (H.W.), yangxiaoming@sinopharm.com (X.Y.)


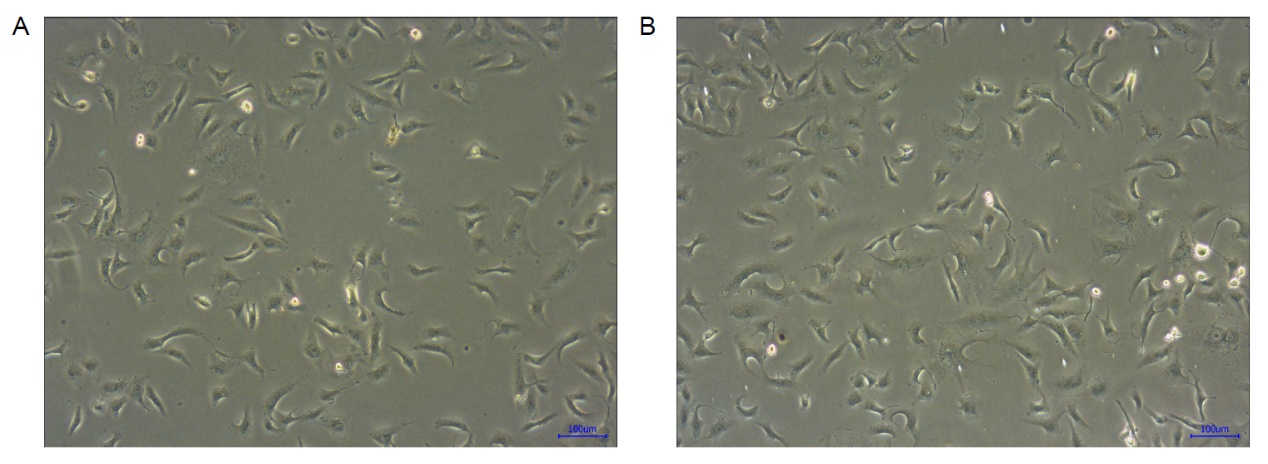


Figure S1. Microscopy of Cell Senescence Assay（A）cells cultured in square flask；（B）cells cultured in a basket bioreactor

**Table S1 GO enrichment analysis of differential genes in transcriptomic analysis ^1^**

| Category | Description | GeneRatio | pvalue | geneName |
| --- | --- | --- | --- | --- |
| BP | tRNA aminoacylation for protein translation | 5/86 | 0.000167 | GARS1/IARS1/WARS1/CARS1/MARS1 |
| BP | amino acid activation | 5/86 | 0.000211 | GARS1/IARS1/WARS1/CARS1/MARS1 |
| BP | tRNA aminoacylation | 5/86 | 0.000211 | GARS1/IARS1/WARS1/CARS1/MARS1 |
| BP | carboxylic acid metabolic process | 8/86 | 0.000285 | ASNS/MTHFD2/GARS1/MSMO1/IARS1/WARS1/CARS1/MARS1 |
| BP | organic acid metabolic process | 8/86 | 0.0003 | ASNS/MTHFD2/GARS1/MSMO1/IARS1/WARS1/CARS1/MARS1 |
| BP | oxoacid metabolic process | 8/86 | 0.0003 | ASNS/MTHFD2/GARS1/MSMO1/IARS1/WARS1/CARS1/MARS1 |
| BP | cellular amino acid metabolic process | 6/86 | 0.000328 | ASNS/GARS1/IARS1/WARS1/CARS1/MARS1 |

^1^ASNS: asparagine synthetase; MTHFD2: Methenyltetrahydrofolate cyclohydrolase; MSMO1: methylsterol monooxygenase 1.

**Table S2 The transcription levels of several genes involved in cell growth increased^1^**

| gene_name | log2FoldChange | pvalue | padj | gene_length |
| --- | --- | --- | --- | --- |
| AREG | 2.54 | 2.85E-08 | 1.05E-05 | 1271 |
| CCND1 | 1.48 | 3.65E-06 | 5.32E-04 | 4245 |
| EREG | 2.54 | 2.01E-05 | 2.00E-03 | 4860 |
| MYC | 1.36 | 4.50E-05 | 3.75E-03 | 2997 |

^1^log2FoldChange: Ratio of gene expression levels in the treatment group to the control group, then logarithm based on 2; P value: p-value of significance test; padj: P value corrected by multiple hypothesis testing. gene_length: Gene length, the sum of all exon non-overlapping regions from gene initiation to termination.

**Table S3 Screening results of protein difference analysis**

| Compared Samples^1^ | Num. of Total Quant.^2^ | regulated Type^3^ | fold-change^4^>1.2 | fold-change>1.3 | fold-change>1.5 | fold-change>2.0 |
| --- | --- | --- | --- | --- | --- | --- |
| EG.vs.CG | 5736 | up-regulated | 951 | 741 | 390 | 88 |
|  |  | down-regulated | 699 | 567 | 354 | 134 |

^1^Compared Samples: Comparison of the sample pairs, the former compared to the latter; ^2^Num.of Total Quant.: Proteins identified together in the two groups of samples; ^3^regulated type: protein regulation type; ^4^fold-change: difference multiple thresholds. EG: the samples of basket bioreactor. CG: the samples of control.

**Table S4 Some up-regulated proteins in BP term of Go enrichment**

| Protein^1^ | Description^2^ | Gene | EG.vs.CG FC | EG.vs.CG  P value^3^ | EG.vs.CG  UP. DOWN | Biological Process Description (GO_ID^4^) |
| --- | --- | --- | --- | --- | --- | --- |
| A0A0D9RKU0 | DNA replication licensing factor MCM3 | MCM3 | 2.13 | 2.41E-05 | up | Biological Process: DNA replication (GO:0006260) |
| A0A0D9RQN8 | DNA replication licensing factor MCM4 | MCM4 | 2.03 | 6.29E-03 | up | Biological Process: DNA replication (GO:0006260) |
| A0A0D9RLR8 | Proliferating cell nuclear antigen | PCNA | 2.14 | 1.33E-03 | up | Biological Process: regulation of DNA replication (GO:0006275) |
| A0A0D9RSM3 | Methenyltetrahydrofolate cyclohydrolase | MTHFD2 | 2.16 | 3.87E-03 | up | Biological Process: folic acid-containing compound biosynthetic process (GO:0009396) |
| A0A0D9RWI0 | Replication factor C subunit 1 | RFC1 | 3.11 | 1.24E-02 | up | Biological Process: DNA replication (GO:0006260) |

^1^Protein: protein ID; ^2^Description: Description of the protein; ^3^P value: P value of enrichment analysis; ^4^GO_ID: GO identification number.

**Table S5 Some up-regulated proteins in cellular component organization term of Go enrichment**

| Protein | Description | Gene | EG.vs.CG FC | EG.vs.CG  P value | EG.vs.CG UP. DOWN | GO_Class^1^ | GO_Term^2^ |
| --- | --- | --- | --- | --- | --- | --- | --- |
| A0A0D9RFT6 | Structural maintenance of chromosomes protein | SMC2 | 2.46 | 6.14E-04 | up | BP | cellular component organization  (GO:0016043) |
| A0A0D9RJF7 | Structural maintenance of chromosomes protein 1A | SMC1A | 2.57 | 1.97E-02 | up |  |  |
| A0A0D9RZ00 | BTB domain-containing protein | / | 2.14 | 3.64E-02 | up |  |  |

^1^GO_Class: GO Category (MF, CC, BP); ^2^GO_Term: Description of GO identification number function.

**Table S6.** Screening results of differential metabolites^1^

| Compared Samples | Num. of Total Ident. | Num. of Total Sig. | Num. of Sig. Up | Num. of Sig. down |
| --- | --- | --- | --- | --- |
| EG.vs.CG. pos | 509 | 164 | 56 | 108 |
| EG.vs.CG. neg | 285 | 119 | 60 | 59 |

^1^VIP value represents the contribution of metabolites to grouping; FC is the ratio of the mean values of all biological replicates of each metabolite in the comparison group; P value is calculated by T-test [4], indicating the significant level of difference. The threshold was set as VIP > 1.0, FC > 1.5 or FC < 0.667 and P value < 0.05.

**Table S7** The detail information of some differential metabolites

| ID | Name | KEGG_pathway | log2FC | Pvalue | VIP | Up.Down |
| --- | --- | --- | --- | --- | --- | --- |
| Com_1480_pos | Cytidine | Metabolic pathways | 3.64 | 2.09E-04 | 1.61 | up |
| Com_6063_pos | Cholecalciferol | Metabolic pathways | 3.15 | 4.03E-03 | 1.38 | up |
| Com_572_pos | Uridine 5'-monophosphate | Metabolic pathways | 1.81 | 7.76E-06 | 1.63 | up |
| Com_218_pos | Guanosine monophosphate | Metabolic pathways | 0.90 | 8.00E-04 | 1.48 | up |
| Com_5126_pos | Psychosine | Sphingolipid metabolism | 2.70 | 4.24E-02 | 1.09 | up |
| Com_11397_pos | Palmitoleic Acid | Fatty acid biosynthesis | 2.50 | 1.62E-02 | 1.20 | up |
| Com_13_pos | Adenosine 5'-monophosphate | cAMP signaling pathway | 0.88 | 3.34E-03 | 1.46 | up |
